# Umpolung of an Aliphatic Ketone to a Magnesium Ketone‐1,2‐diide Complex with Vicinal Dianionic Charge

**DOI:** 10.1002/anie.202204472

**Published:** 2022-07-11

**Authors:** Stuart Burnett, Connor Bourne, Alexandra M. Z. Slawin, Tanja van Mourik, Andreas Stasch

**Affiliations:** ^1^ EaStCHEM School of Chemistry University of St Andrews North Haugh St Andrews KY16 9ST UK

**Keywords:** Dianions, Low Oxidation States, Magnesium, Reductions, Umpolung

## Abstract

The new β‐diketimine ^*i*PrDip^nacnacH, HC(*i*PrCNDip)_2_H, Dip=2,6‐*i*Pr_2_‐C_6_H_3_, was converted to the magnesium(I) complex [{(^*i*PrDip^nacnac)Mg}_2_] and reaction with 2‐adamantanone (OAd) afforded the ketone‐1,2‐diide complex [{(^*i*PrDip^nacnac)Mg}_2_(μ‐OAd)]. The complex contains the first stable dianion of an aliphatic ketone with an electropositive metal and shows an OAd^2−^ unit with long C−O bond and pyramidal carbon centre. DFT studies reveal an anionic charge on both neighbouring C and O atoms. Reductions of aliphatic ketones with magnesium(I) complexes show that these likely proceed via highly reactive dianions and afforded a 1 : 1 mixture of an alkoxide and an enolate when an enolisable ketone was used, and rapid CH activations reactions, e.g., of stabilising ligand moieties, when non‐enolisable ketones were employed.

## Graphical abstract

The magnesium(I) complex of a new β‐diketiminate ligand formed with 2‐adamantanone a magnesium ketone‐1,2‐diide complex with vicinal dianionic charge. The reactivity of aliphatic ketone reductions is explained via ketone‐1,2‐diide intermediates including facile CH activations.

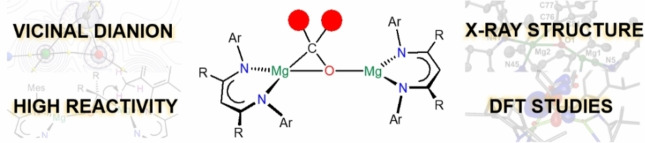

## Introduction

Carbonyl compounds such as ketones have their reactivity dominated by the electrophilic character of the carbonyl carbon atom. Umpolung of carbonyl compounds to gain some nucleophilic character at the carbon centre to undergo valuable C−C bond forming reactions can be achieved via a wide variety of stoichiometric and catalytic approaches in organic synthesis.^[1]^ The electrophilic character of ketones can also be altered by reduction via electron addition to the ketone π‐system. Reactions of ketones with strong reducing agents such as electropositive metals have been studied for well over 100 years[^2^, ^3^] and have, for example, led to isolable complexes of highly coloured ketyl radical anions and ketone dianions, e.g., with alkali metals,^[4]^ and a variety of uses in organic synthesis,^[5]^ for example in pinacol coupling reactions^[6]^ and the McMurry reaction.^[7]^ Electrochemically, ketones such as benzophenone show a reversible reduction to the radical anion and an irreversible to quasi‐reversible one to the dianion as determined from cyclic voltammetry.^[8]^ For example, [K(OCPh_2_)] in THF shows a second reduction wave at −3.28 V (vs Fc/Fc^+^) to the dianion^[4a]^ and highlights that very strongly reducing conditions are required to afford dianions.[^9^, ^10^] In reactions with alkali metals, Geier, Grützmacher and co‐workers were able to study molecular structures of sodium complexes of Ph_2_CO^2−^ ions with donor ligands.^[9]^ The structures show the planarity around the central carbon atom and suggest the wider delocalisation of the charge in the dianion via the phenyl groups. DFT calculations on the model complex [Na_2_(OCPh_2_)] afforded an NPA charge of +0.38 on the central carbon atom and thus it had been concluded that these species cannot be regarded as vicinal dianions. In addition, some lanthanoid complexes^[11]^ and transition metal complexes of groups 4–6^[12]^ form complexes with significantly reduced ketone fragments, largely for aromatic ketones. The Ln examples show dianionic ketone fragments with planar carbonyl centres similar to those for alkali metal complexes, and the transition metal examples show significantly more pyramidalization at the carbonyl unit in a metallaoxirane ring with more covalency and interaction of a metal *d*‐orbital with the R_2_CO π*‐orbital.^[13]^ In general, the majority of studies have been undertaken with aromatic ketones and there has been a discussion whether dianions of aliphatic ketones can be accessed at all for organic synthesis or if some observed reactivity is purely based on radical disproportionation chemistry of ketyl anions.^[14]^ More generally in anion chemistry, the formation of di‐ or even polyanionic species can suffer from stability issues such as Coulomb explosion.^[15]^ Anionic units in close proximity typically require stabilising factors such as coordination to metal cations and delocalisation of the charge via functional groups including aryl substituents.^[16]^ Despite this, organometallics with nucleophilic groups in close proximity have been forthcoming,[^16^, ^17^] but are rare when compared to monofunctional organometallics.

Dimagnesium(I) compounds of general formula LMgMgL, where L is an anionic ligand such as a β‐diketiminate, have shown to act as very strong molecular reducing agents,^[18]^ for example in reversible double reduction of selected alkenes and alkynes,^[19]^ related reduction of phosphaalkyne,^[20]^ and systems that could reduce CO.^[21]^ Their strength was also demonstrated in the facile reduction of fullerene C_60_ to its hexaanion, a substrate with six equidistant reduction waves and a final *E*_1/2_(C_60_^5−/6−^) of −3.28 V (vs Fc/Fc^+^).^[22]^ In reactions with ketones, a purple‐blue ketyl radical complex was previously structurally characterised from the reaction of LMgMgL with benzophenone after complexation with DMAP (4‐dimethylaminopyridine),^[23]^ and with related esters C−O bond scission to Mg acyl complexes has been observed.^[24]^ Very recently, low valent magnesium chemistry was extended to β‐diketiminate magnesium(0) complexes and this further demonstrates the suitability of this ligand class with strongly reducing Mg species.^[25]^ Here we report the synthesis and characterisation of a magnesium complex of an aliphatic ketone‐1,2‐diide, i.e. a vicinal alkoxide methanide, and associated reactivity via ketone dianions.

## Results and Discussion

**Scheme 1:**
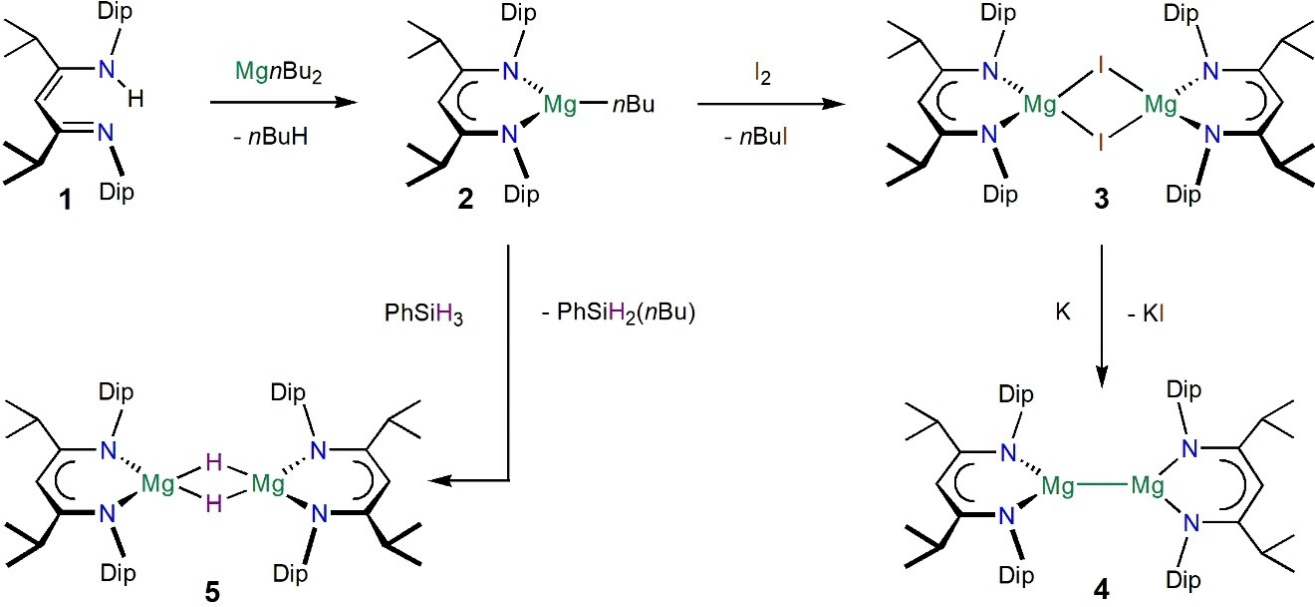
Synthesis of complexes **2**–**5**. Isolated yields: **2** (79 %), **3** (70 %), **4** (60 %), **5** (37 %) and higher in situ yields.

We have prepared the new β‐diketimine^[26]^ proligand ^*i*PrDip^nacnacH, HC(*i*PrCNDip)_2_H, Dip=2,6‐*i*Pr_2_‐C_6_H_3_, **1**, in a one pot condensation reaction, with the aim of introducing a highly robust ligand backbone unit to this ligand class.^[27]^ Conversion of **1** via [(^*i*PrDip^nacnac)Mg*n*Bu] **2**, and [{(^*i*PrDip^nacnac)MgI}_2_] **3**, followed by reduction with potassium metal, afforded the magnesium(I) complex [{(^*i*PrDip^nacnac)Mg}_2_] **4**, in a similar manner to related syntheses, see scheme 1.^[28]^ [(^*i*PrDip^nacnac)Mg*n*Bu] **2**, is also readily converted to [{(^*i*PrDip^nacnac)MgH}_2_] **5** (Scheme 1).[^28b^, ^28c^, ^29^] Compounds **1**–**5** show expected spectroscopic properties.^[27]^

**Scheme 2:**
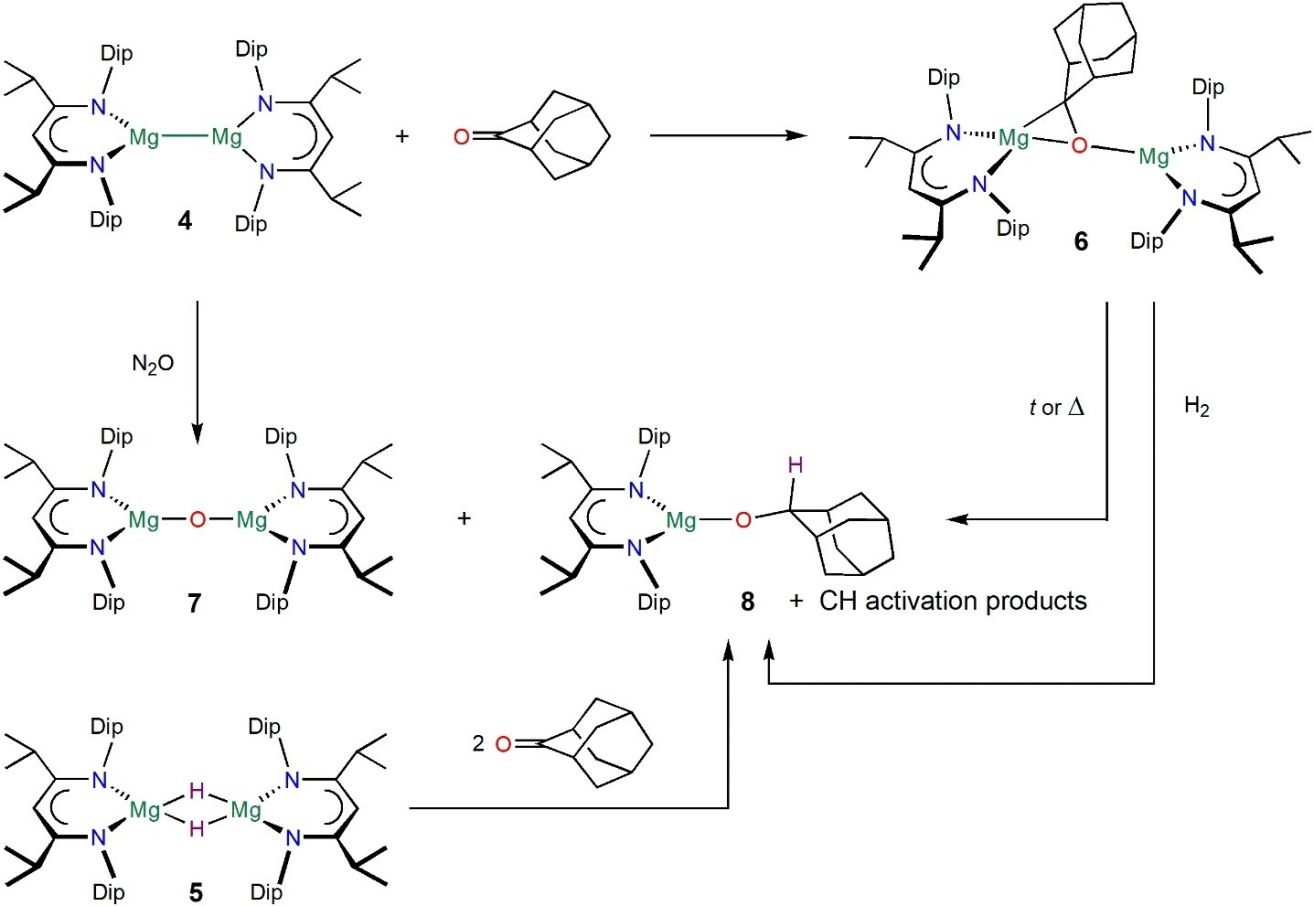
Synthesis and decomposition of complex **6**, synthesis of complexes **7** and **8**. Isolated yields: **6** (65 %, quantitative in situ yield), **7** (39 %), **8** (31 %) and higher in situ yields.

**Figure 1:**
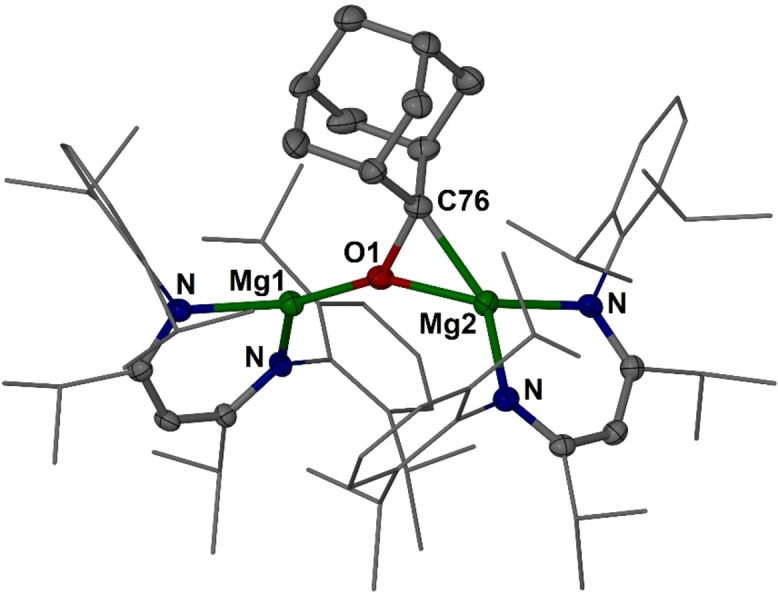
Molecular structure (30 % thermal ellipsoid) of complex **6** ⋅ C_6_H_6_. Hydrogen atoms, solvent molecule and minor disordered parts omitted for clarity. Dip and *i*Pr groups shown as wireframe. Selected bond lengths [Å] and angles [°]: Mg1−O1 1.884(3), Mg2−O1 2.024(3), Mg2−C76 2.106(5), O1−C76 1.539(5); C76−O1−Mg1 138.1(2), Mg1−O1−Mg2 150.09(16); sum of angles around C76 excluding Mg2: 327.9(11)°.

With the aim of preparing a magnesium complex of a ketone dianion, reactions of [{(^*i*PrDip^nacnac)Mg}_2_] **4**, with benzophenone in a 1 : 1 ratio led to highly coloured purple reaction mixtures, similar to the synthesis of a purple‐blue magnesium ketyl radical,^[23]^ but no pure complex was isolated. Attention turned to aliphatic ketones including 2‐adamantanone (OAd) due to its rigid carbon framework which renders it a non‐enolisable ketone.^[30]^ The reaction of [{(^*i*PrDip^nacnac)Mg}_2_] **4**, with one equivalent of 2‐adamantanone at room temperature rapidly and cleanly afforded the reduced ketone complex [{(^*i*PrDip^nacnac)Mg}_2_(μ‐OAd)] **6**, as yellow crystals, see Scheme 2 and Figure 1.

In the molecular structure of [{(^*i*PrDip^nacnac)Mg}_2_(μ‐OAd)] ⋅ C_6_H_6_, **6** ⋅ C_6_H_6_, (Figure 1),^[27]^ some outer groups show poor ordering, but the central section is well ordered. In **6**, the dianionic OAd^2−^ unit bridges the two LMg^+^ fragments in a μ‐κ^1^ *O* : η^2^ *O*,*C* fashion with an Mg2−C76 bond lengths of 2.106(5) Å, and a shorter Mg−O bond for Mg1 (1.884(3) Å) than for Mg2 (2.024(3) Å). C76 shows a pyramidal geometry in the dianionic OCAd^2−^ unit with an angle sum of 327.9(11)° (ideal 328.4°). The coordination environment around C76, considering the σ‐bonded carbon substituents and coordination to Mg2 but excluding O1, is nearly planar with an angle sum of 358.4(10)° (ideal 360°). The Mg−O−Mg angle in **6** (150.09(16)°) is quite obtuse and approaching a linear geometry. Related to this, the C−O bond length (1.539(5) Å) is very long, and significantly longer than other crystallographically characterised reduced ketone units in complexes of electropositive metals.[^9^, ^10^, ^11^, ^12^] For example, alkali metal complexes of dianionic aromatic ketones show C−O bond length of ca. 1.38–1.41 Å.[^9^, ^10^] There is, however, one reported osmium formaldehyde complex, [Os(η^2^‐CH_2_O)(CO)_2_(PPh_3_)_2_] ⋅ H_2_O, with a C−O bond length of 1.584(11) Å and the authors suggest that the significant lengthening could be due to incipient C−O bond rupture to metal oxide and metal carbene fragments.^[31]^ ^1^H and ^13^C{^1^H} NMR spectra of **6** show that the unsymmetric complex environment is retained in solution with two resonances for the ligand backbone CH units, and four different ^13^C{^1^H} NMR resonances for ligand *C*N units, likely due to interlocking of the large substituents in **6**. The ^13^C{^1^H} NMR resonance of the anionic carbon is found at *δ*=98.8 ppm.

**Figure 2:**
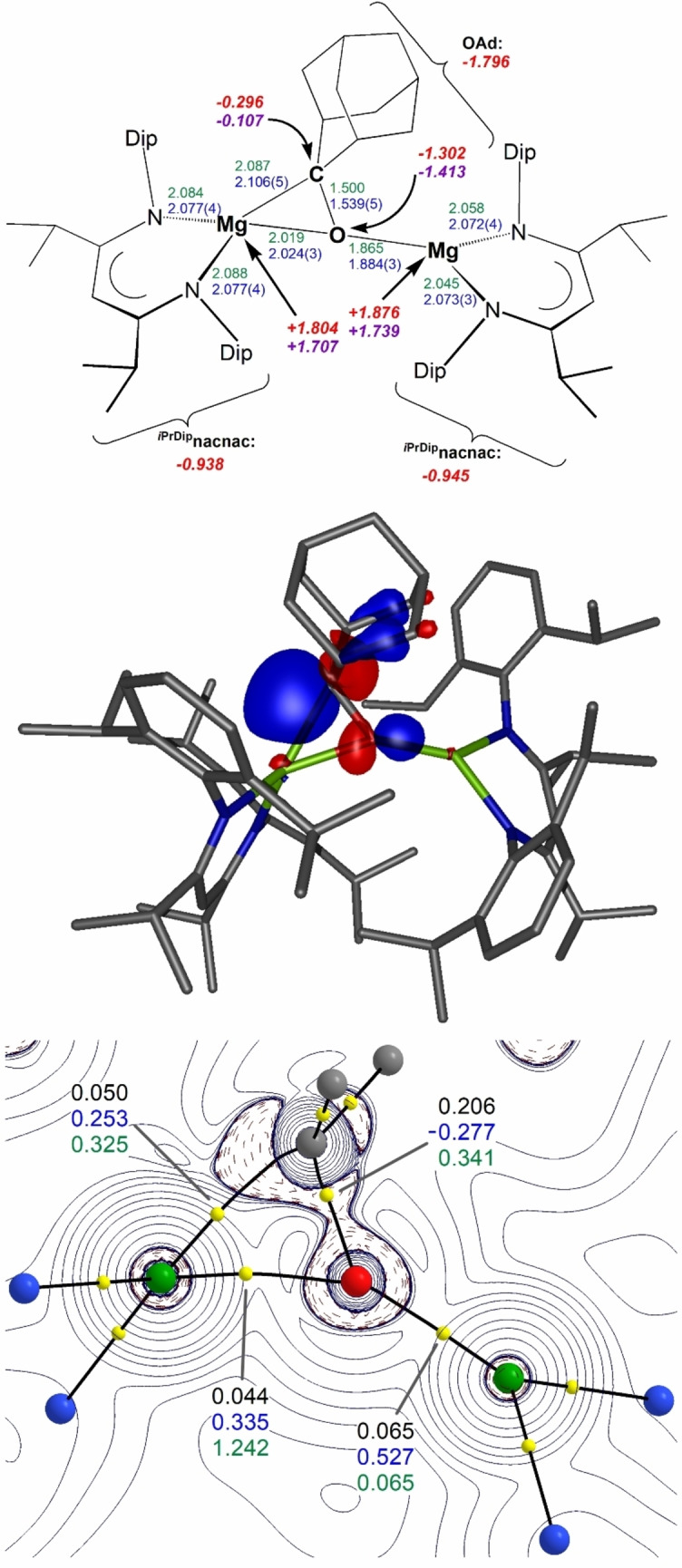
DFT studies (M06/def2‐TZVP+D3) of complex **6**. Top: Selected bond lengths from DFT optimisation (green) and X‐ray diffraction (blue) in Å, and calculated charges from NPA (red) and QTAIM (purple) analysis; Middle: HOMO (−4.24 eV) of complex **6** (isovalue 0.06); Bottom: QTAIM contour plots of the Laplacian of electron density (solid lines positive, dashed lines negative) through the MgCO plane showing only the core atoms (Mg green, O red, N blue, C grey), bond paths (black) and bond critical points (yellow). Values for the electron density, *ρ*(*r*) (black), Laplacian, ∇^*2*^ *ρ*(*r*) (blue), and bond ellipticity, *ϵ* (green), are given for key bond critical points.

To the best of our knowledge, complex **6** is the first isolated example of a dianionic aliphatic ketone complex of an *s*‐block metal, or of any other significantly electropositive metal, and thus likely the only one with a negative charge on the former ketone carbon centre. A DFT study at the M06/def2‐TZVP level of theory with D3 dispersion addition^[27]^ of complex **6** reproduced the geometry from X‐ray crystallography well with similar key bond lengths, see Figure 2, top. The contribution of dispersion to the stability of the complex has been found to be sizeable.^[27]^ The largest deviation is found for the C−O bond length which is only ca. 2.6 % shorter. The HOMO of complex **6** (Figure 2, middle) contains the Mg−C σ‐bonding interaction with some C−O π* contribution. Calculated charges from NPA of −0.296 for C and −1.302 for O in the ketone‐1,2‐diide unit and an overall charge of −1.80 for the reduced adamantanone fragment support the formulation of a dianionic system with vicinal anionic charge on the CO unit. For comparison, a calculated NPA charge for the carbon centre of +0.38 in the model complex [Na_2_(OCPh_2_)] highlights the significant difference between both systems (Δ_q_>0.6).^[9]^ Determined charges from QTAIM calculations of −0.107 for C and −1.413 for O distribute the charge slightly differently, but still provide a vicinal dianionic charge with a partial negative charge on the carbon atom. A contour map of the Laplacian of the electron density from QTAIM studies (Figure 2, bottom) shows the charge depletion (solid lines) and accumulation (dashed lines) in the MgCO plane including values for the electron density, *ρ*(*r*), Laplacian, ∇^*2*^ *ρ*(*r*), and bond ellipticity, *ϵ*, for key bond critical points that highlight the nature of the electron‐rich CO fragment. A Wiberg bond index (WBI) of 0.861 for the C−O bond shows the reduction of the bond order and the WBI of 0.110 for the Mg−C interaction is larger than those found for the Mg−O/N interactions.

Complex **6** is thermally sensitive and slowly decomposes in hydrocarbon solution to a mixture of products, including the magnesium oxide complex [{(^*i*PrDip^nacnac)Mg}_2_O] **7**, and by implication decomposition products of the C_10_H_14_ adamantane‐2‐ylidene carbene,^[32]^ the alkoxide complex [(^*i*PrDip^nacnac)Mg(OAdH)] **8**, and other products, including C−H activation products, possibly from a Dip methyl group as judged by ^1^H NMR spectroscopy and 2D NMR experiments, as the source of the hydrogen atom in complex **8**. Decompositions at relatively low temperature, e.g., room temperature, show a high percentage of formation of **8** and a putative CH activation product, whereas high temperature decomposition reactions increasingly produce more C−O cleavage product **7** and by implication hydrocarbon by‐products,^[32]^ likely for entropic reasons. The molecular structure, the bonding situation and charge distribution in **6** appears to be suitably set up for degradation into the MgOMg species **7** and the adamantane‐2‐ylidene carbene fragment. Storage of solid samples of **6** at room temperature in a glove box also leads to some degradation and samples are best stored in a glove box freezer. [{(^*i*PrDip^nacnac)Mg}_2_O] **7**, and its THF‐adduct [{(^*i*PrDip^nacnac)Mg(THF)}_2_O], were independently prepared by the reaction of [{(^*i*PrDip^nacnac)Mg}_2_] **4**, with N_2_O (scheme 2) as in a previous example,^[33]^ and [(^*i*PrDip^nacnac)Mg(OAdH)] **8** was independently obtained from reactions of [{(^*i*PrDip^nacnac)MgH}_2_] **5**, with 2‐adamantanone, OAd (scheme 2), or from [(^*i*PrDip^nacnac)Mg*n*Bu] **2** with 2‐adamantanol, HOAdH.^[27]^

Complex **6** contains a nucleophilic carbon centre from the direct ketone Umpolung, but the ketone‐1,2‐diide unit is also expected to be extremely basic, highly reducing, and is sterically extremely protected (see the space‐filling models in Figure S107). Complex **6** reacts with hydrogen at room temperature (1 bar) within minutes to hours to finally form [(^*i*PrDip^nacnac)Mg(OAdH)] **8**, see scheme 2, and unknown by‐products. It is tempting to suggest that the reaction proceeds via a proposed hydride bridged intermediate [(^*i*PrDip^nacnac)Mg(μ‐OAdH)(μ‐H)Mg(^*i*PrDip^nacnac)], c.f. the reactivity of a reduced diphenylethylene magnesium complex,^[19]^ but no formation of magnesium hydride **5** was observed. Likely for steric reasons, complex **6** did not react at room temperature in deuterated benzene with iodomethane, bis(pinacolato)diboron, 2,2,6,6‐tetramethylpiperidine or N_2_O, respectively, and with longer reaction times or higher temperatures decomposition of **6** became dominant. Preliminary reactivity studies towards organic electrophiles often afforded product mixtures including the formation of **8** from hydrogen abstraction.

**Scheme 3:**
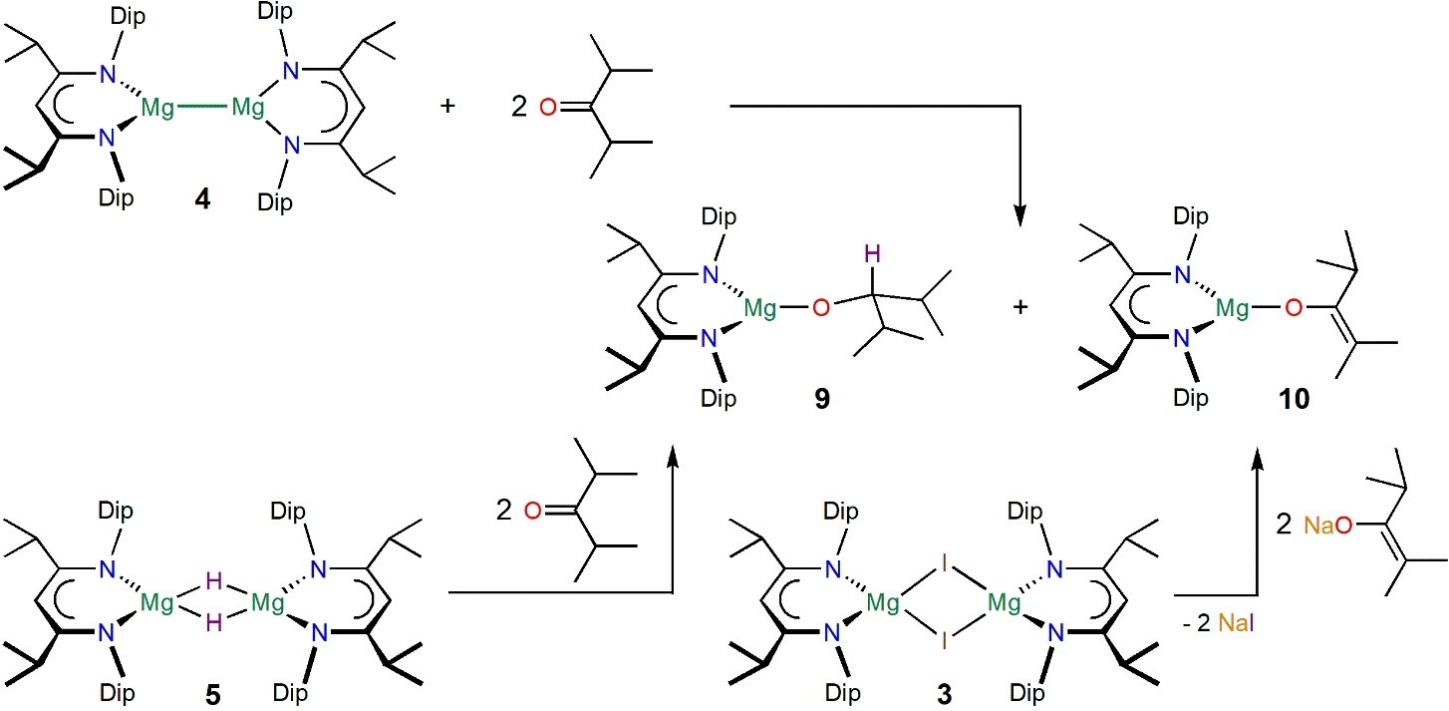
Synthesis of complexes **9** and **10**. Isolated yields: **9** (39 %), **10** (36 %) and higher in situ yields.

In studies with other aliphatic ketones, the reaction of [{(^*i*PrDip^nacnac)Mg}_2_] **4**, with enolisable diisopropyl ketone (2,4‐dimethyl‐3‐pentanone) proceeded at room temperature in a preferred 1 : 2 ratio and afforded an approximate 1 : 1 mixture of the alkoxide [(^*i*PrDip^nacnac)Mg(OCH*i*Pr_2_)] **9** and the enolate [(^*i*PrDip^nacnac)Mg{OC(=CMe_2_)*i*Pr}] **10**, scheme 3. The similar carbon substituting pattern and approximately similar steric profile near the carbonyl unit between diisopropyl ketone and 2‐adamantanone makes it likely that the reaction proceeds via a dianion stage, but because diisopropyl ketone is enolisable, the dianion deprotonates a second equivalent of diisopropyl ketone to form the mixture of **9** and **10**. It is worth noting that no intense colours or pinacol coupling products were observed and the reaction likely does not proceed via ketyl radical anions.

**Figure 3:**
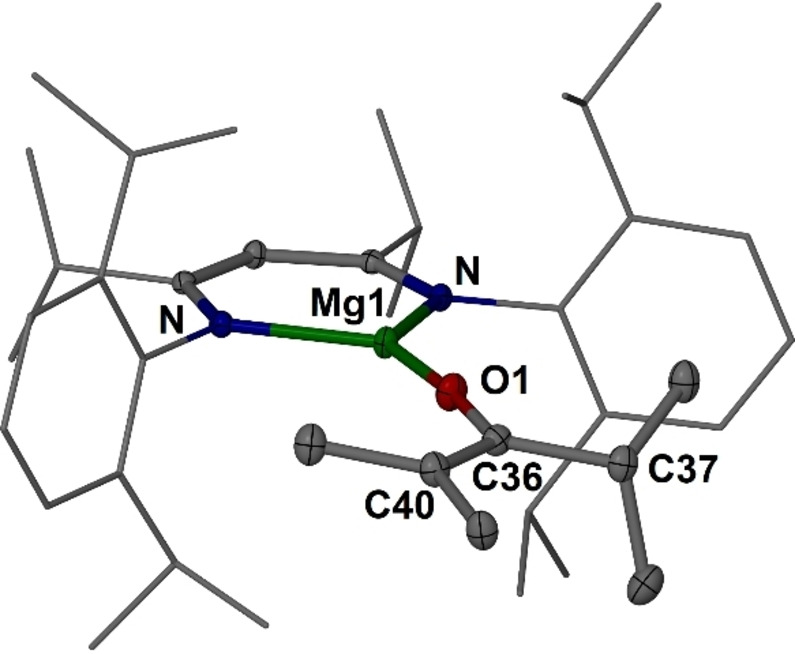
Molecular structures (30 % thermal ellipsoid) of complex **10**. Hydrogen atoms omitted for clarity. Dip and *i*Pr groups shown as wireframe. Selected bond lengths [Å] and angles [°]: Mg1−O1 1.7984(10), O1−C36 1.3477(15), C36−C40 1.3528(19), C36−C37 1.5279(19); C36−O1−Mg1 173.77(10), O1−C36−C40 122.44(12).

Monomeric complexes **9** and **10** co‐crystallise due to their similar overall structure and thus could not be separated. Complex **9** was independently prepared by a hydromagnesation of [{(^*i*PrDip^nacnac)Mg(μ‐H)}_2_] **5** with diisopropyl ketone and complex **10** was independently afforded via a salt‐metathesis of [{(^*i*PrDip^nacnac)Mg(μ‐I)}_2_] **3** with the sodium enolate of diisopropyl ketone.^[27]^ A molecular structure of **9** showed poor quality due to severe disorder and is not reported here, but the molecular structure of [(^*i*PrDip^nacnac)Mg{OC(=CMe_2_)*i*Pr}] **10** is well‐ordered (Figure 3) and provides metrical data for a monomeric enolate complex.

**Scheme 4:**
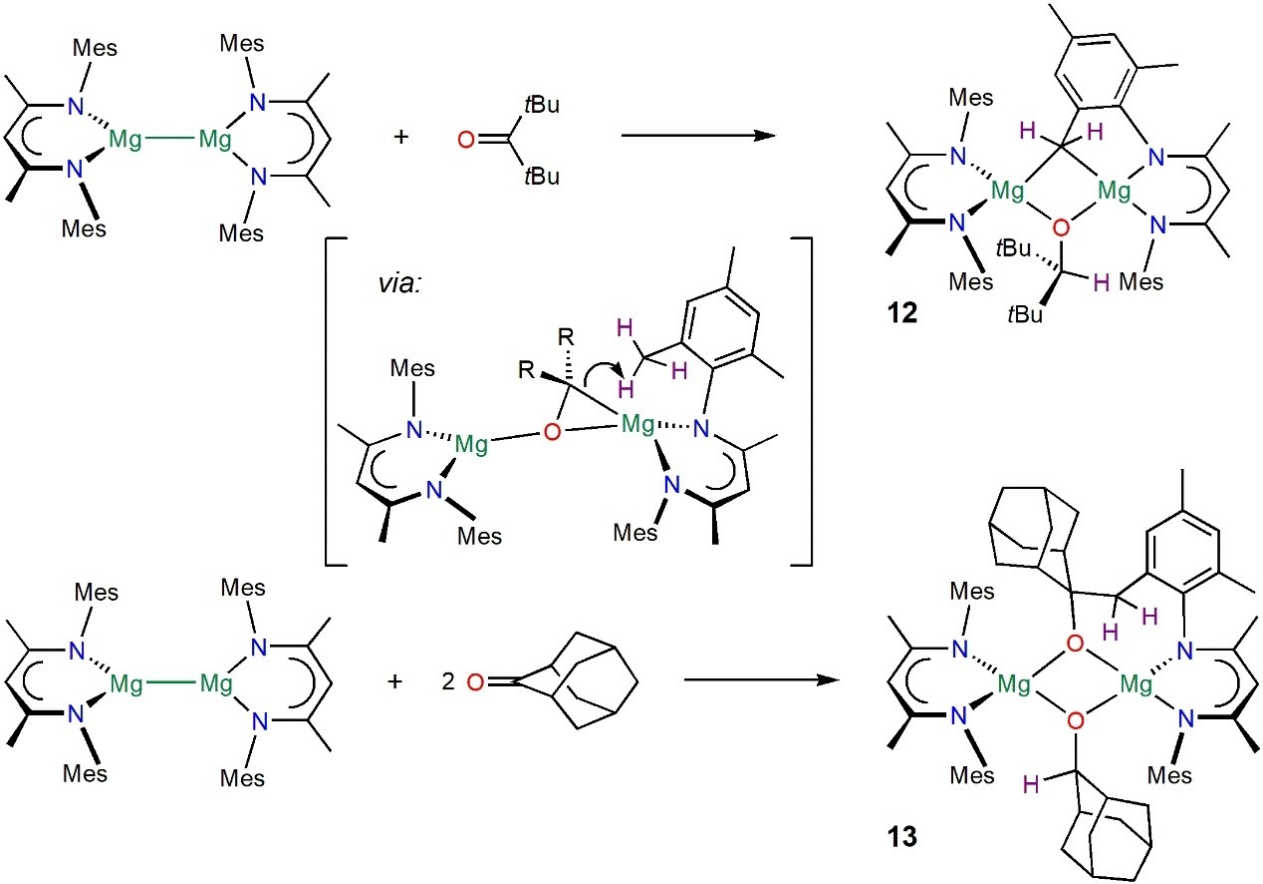
Synthesis of complexes **12** and **13**. Isolated yields: **12** (64 %), **13** (68 %) and higher in situ yields.

**Figure 4:**
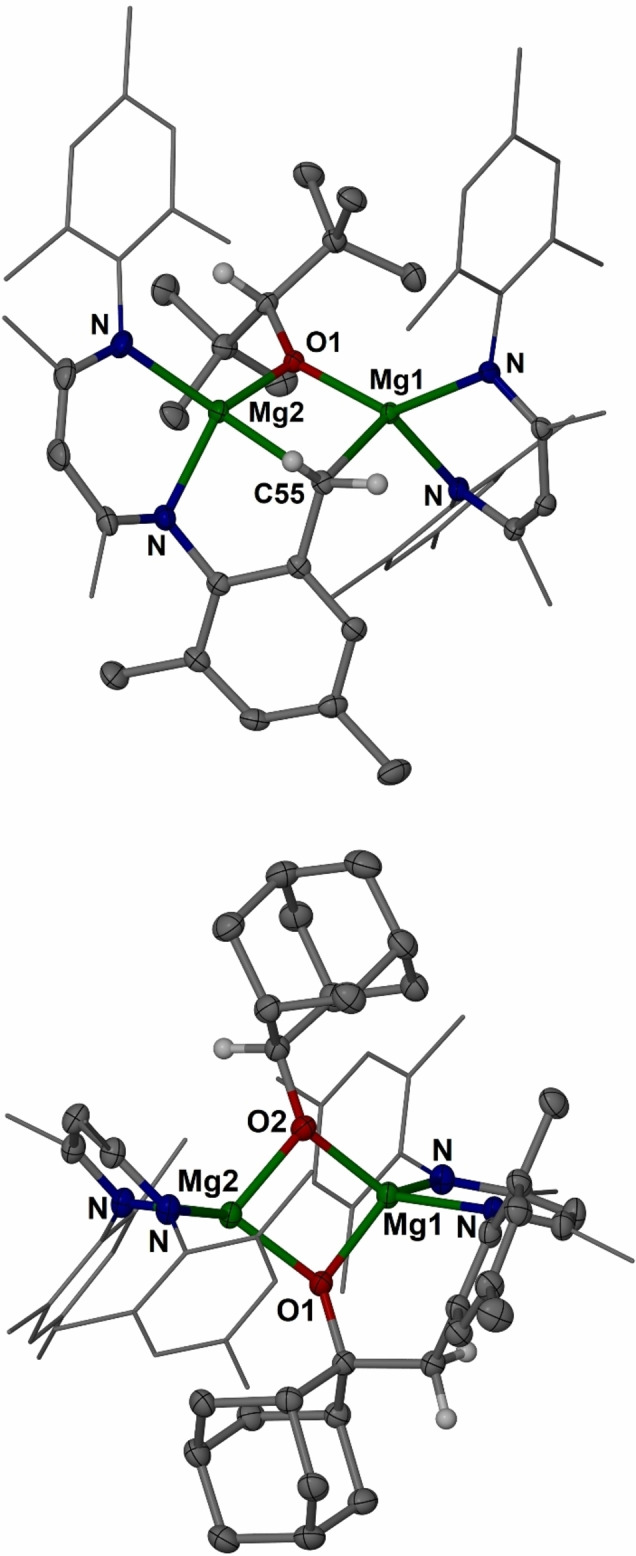
Molecular structures (30 % thermal ellipsoid) of complexes **12** (top) and **13** (bottom). Hydrogen atoms, except those from CH activated groups, and minor disordered parts omitted for clarity. Mes and Me groups shown as wireframe. Selected bond lengths [Å]: **12**: Mg1−O1 1.9491(13), Mg2−O1 1.9619(14), Mg1−C55 2.293(2), Mg2−C55 2.368(2); **13**: Mg1−O2 1.9550(13), Mg1−O1 2.0136(13), Mg2−O1 1.9785(13), Mg2−O2 1.9844(13).

To study a different non‐enolisable ketone with a potentially more basic accessible dianion, we investigated reductions of di‐*tert*‐butyl ketone (2,2,4,4‐tetramethyl‐3‐pentanone).[^34^, ^35^] Complex [{(^*i*PrDip^nacnac)Mg}_2_] **4** does not react with di‐*tert*‐butyl ketone at room temperature or when briefly heated, likely for steric reasons, but slowly formed [(^*i*PrDip^nacnac)Mg(OCH*t*Bu_2_)] **11** after two weeks at 80 °C from hydrogen abstraction (scheme 3), plus other products. The smaller Mg^I^ complex [{(^MeMes^nacnac)Mg}_2_], ^MeMes^nacnac=HC(MeCNMes)_2_, Mes=2,4,6‐Me_3_‐C_6_H_2_,^[28c]^ however, rapidly reacts with di‐*tert*‐butyl ketone at room temperature to a single product, [{(^MeMes^nacnac)Mg}(μ‐OCH*t*Bu_2_)(μ‐CH_2_‐^MeMes−H^nacnac)Mg] **12**, where CH_2_‐^MeMes−H^nacnac denotes the ligand deprotonated in an ortho methyl group position, see scheme 4 and Figure 4. To form complex **12**, the putative extremely basic intermediate [{(^MeMes^nacnac)Mg}_2_(μ‐OC*t*Bu_2_)] deprotonates an ortho methyl group from a Mes substituent furnishing the bridging RCH_2_ unit between the two Mg centres and a bridging alkoxide OC(H)*t*Bu_2_. Complex **12** does not react with further equivalents of di‐*tert*‐butyl ketone. In the ^1^H NMR spectrum of **12**, the bridging CH_2_ group shows two doublets (^2^ *J*_HH_=14 Hz) at *δ*=1.58 and *δ*=2.16 ppm for the two inequivalent hydrogen atoms and a resonance at *δ*=17.1 ppm in the ^13^C{^1^H} NMR spectrum. The smaller Mg^I^ complex [{(^MeMes^nacnac)Mg}_2_] was then reacted with 2‐adamantanone at room temperature and was found to preferably react in a 1 : 2 ratio to afford [{(^MeMes^nacnac)Mg}(μ‐OAdH)(μ‐O(Ad)CH_2_‐^MeMes−H^nacnac)Mg] **13**, see scheme 4 and Figure 4. Seemingly, [{(^MeMes^nacnac)Mg}_2_] reacts with one equivalent of OAd to the reactive intermediate [{(^MeMes^nacnac)Mg}_2_(μ‐OAd)], c.f. complex **6**, which CH‐activates a Mes substituent to an intermediate similar to complex **12**, i.e. [{(^MeMes^nacnac)Mg}(μ‐OAdH)(μ‐CH_2_‐^MeMes−H^nacnac)Mg], which can be observed alongside **13** when the reaction is carried out in a 1 : 1 stoichiometric ratio, and nucleophilicly attacks another equivalent of OAd to form complex **13**.

## Conclusion

With the help of an Mg−Mg complex of a new robust β‐diketiminate ligand and non‐enolisable 2‐adamantanone (OAd) we have prepared the first *s*‐block metal complex of a dianionic aliphatic ketone, [{(^*i*PrDip^nacnac)Mg}_2_(μ‐OAd)] **6**. A fine balance of sterics and a significant influence of dispersion forces^[36]^ between the adamantane‐cage and the Dip substituents plus the robustness of the new ligand system allowed the stabilisation of this extremely basic organometallic fragment that undergoes decomposition via CH activation reactions and C−O bond scission. The molecular structure of **6** shows a pyramidal arrangement around the anionic carbon centre in its coordination to an Mg centre and a very long C−O bond. DFT computational studies show an anionic charge on both the carbon and oxygen centres of the dianionic ketone fragment demonstrating the direct Umpolung by reduction of the ketone. Complex **6** readily reacts with dihydrogen under mild conditions. Reduction pathways via highly reactive aliphatic ketone dianions also explain other outcomes of ketone reductions studied in here, namely the formation of enolate and reduced alkoxide for enolisable ketones, and the facile CH activation, e.g., of stabilising ligand moieties, and reduced alkoxides for non‐enolisable ketones. No pinacol coupling products were observed from the reactions studied in here and the observations are in line with proceeding via dianions. This work shows that reactions can proceed via complexes of dianions of aliphatic ketones.

## Conflict of interest

The authors declare no conflict of interest.

1

## Supporting information

As a service to our authors and readers, this journal provides supporting information supplied by the authors. Such materials are peer reviewed and may be re‐organized for online delivery, but are not copy‐edited or typeset. Technical support issues arising from supporting information (other than missing files) should be addressed to the authors.

Supporting Information

Supporting Information

Supporting Information

Supporting Information

Supporting Information

Supporting Information

Supporting Information

Supporting Information

Supporting Information

Supporting Information

Supporting Information

Supporting Information

Supporting Information

Supporting Information

Supporting Information

Supporting Information

## Data Availability

X‐ray crystallography data has been deposited with the CCDC. The research data (NMR spectroscopy, computational studies) supporting this publication can be accessed at https://doi.org/10.17630/326e2023‐ecb9‐4814‐90af‐62caa75828a5. Further data that support the findings of this study are available in the supplementary material of this article.
