# Supplementary material for: Umpolung of an Aliphatic Ketone to a Magnesium Ketone‐1,2‐diide Complex with Vicinal Dianionic Charge
Source: Angew Chem Int Ed Engl. 2022 Jul 11;61(34):e202204472. doi: 10.1002/anie.202204472 (PMC9541192; doi:10.1002/anie.202204472)

checkCIF/PLATON report

You have not supplied any structure factors. As a result the full set of tests cannot be run.

THIS REPORT IS FOR GUIDANCE ONLY. IF USED AS PART OF A REVIEW PROCEDURE FOR PUBLICATION, IT SHOULD NOT REPLACE THE EXPERTISE OF AN EXPERIENCED CRYSTALLOGRAPHIC REFEREE.

No syntax errors found. CIF dictionary Interpreting this report

Datablock: MDAS43

Bond precision: C-C = 0.0033 A Wavelength=0.71075

Cell:	a=17.0179(12)	b=42.405(3)	c=8.3891(6)
	alpha=90	beta=90	gamma=90
Temperature:	173 K		

	Calculated	Reported
Volume	6053.9(7)	6053.9(7)
Space group	F d d 2	F d d 2
Hall group	F 2 -2d	F 2 -2d
Moiety formula	C33 H50 N2	C33 H50 N2
Sum formula	C33 H50 N2	C33 H50 N2
Mr	474.75	474.77
Dx,g cm-3	1.042	1.042
Z	8	8
Mu (mm-1)	0.059	0.059
F000	2096.0	2096.0
F000'	2096.65	
h,k,lmax	20,50,10	20,50,10
Nref	2785[1496]	2764
Tmin,Tmax	0.991,0.991	0.861,0.991
Tmin'	0.991	

Correction method= # Reported T Limits: Tmin=0.861 Tmax=0.991
AbsCorr = MULTI-SCAN

Data completeness= 1.85/0.99 Theta(max)= 25.381

R(reflections)= 0.0341(2684) wR2(reflections)= 0.0957(2764)

S = 1.046 Npar= 169

The following ALERTS were generated. Each ALERT has the format
test-name_ALERT_alert-type_alert-level.
Click on the hyperlinks for more details of the test.

Alert level C

```
PLAT220_ALERT_2_C NonSolvent   Resd 1   C   Ueq(max)/Ueq(min) Range        4.3 Ratio
PLAT242_ALERT_2_C Low    'MainMol' Ueq as Compared to Neighbors of        C10 Check
PLAT242_ALERT_2_C Low    'MainMol' Ueq as Compared to Neighbors of        C13 Check
```

Alert level G

```
CHEMS02_ALERT_1_G  Please check that you have entered the correct
            _publ_requested_category classification of your compound;
            FI or CI or EI for inorganic; FM or CM or EM for metal-organic;
            FO or CO or EO for organic.
            From the CIF: _publ_requested_category   CHOOSE FI FM FO CI CM CO or A
            From the CIF: _chemical_formula_sum :C33 H50 N2
PLAT002_ALERT_2_G Number of Distance or Angle Restraints on AtSite          2 Note
PLAT032_ALERT_4_G Std. Uncertainty on Flack Parameter Value High .      0.500 Report
PLAT172_ALERT_4_G The CIF-Embedded .res File Contains DFIX Records          1 Report
PLAT300_ALERT_4_G Atom Site Occupancy of H1         Constrained at        0.5 Check
PLAT850_ALERT_4_G Check Flack Parameter Exact Value 0.00 with s.u.       0.50 Check
PLAT860_ALERT_3_G Number of Least-Squares Restraints .............          2 Note
```

0 **ALERT level A** = Most likely a serious problem - resolve or explain
0 **ALERT level B** = A potentially serious problem, consider carefully
3 **ALERT level C** = Check. Ensure it is not caused by an omission or oversight
7 **ALERT level G** = General information/check it is not something unexpected

1 ALERT type 1 CIF construction/syntax error, inconsistent or missing data
4 ALERT type 2 Indicator that the structure model may be wrong or deficient
1 ALERT type 3 Indicator that the structure quality may be low
4 ALERT type 4 Improvement, methodology, query or suggestion
0 ALERT type 5 Informative message, check

It is advisable to attempt to resolve as many as possible of the alerts in all categories. Often the minor alerts point to easily fixed oversights, errors and omissions in your CIF or refinement strategy, so attention to these fine details can be worthwhile. In order to resolve some of the more serious problems it may be necessary to carry out additional measurements or structure refinements. However, the purpose of your study may justify the reported deviations and the more serious of these should normally be commented upon in the discussion or experimental section of a paper or in the "special_details" fields of the CIF. checkCIF was carefully designed to identify outliers and unusual parameters, but every test has its limitations and alerts that are not important in a particular case may appear. Conversely, the absence of alerts does not guarantee there are no aspects of the results needing attention. It is up to the individual to critically assess their own results and, if necessary, seek expert advice.

Publication of your CIF in IUCr journals

A basic structural check has been run on your CIF. These basic checks will be run on all CIFs submitted for publication in IUCr journals (*Acta Crystallographica*, *Journal of Applied Crystallography*, *Journal of Synchrotron Radiation*); however, if you intend to submit to *Acta Crystallographica Section C* or *E* or *IUCrData*, you should make sure that full publication checks are run on the final version of your CIF prior to submission.

Publication of your CIF in other journals

Please refer to the *Notes for Authors* of the relevant journal for any special instructions relating to CIF submission.

PLATON version of 13/07/2021; check.def file version of 13/07/2021

Datablock MDAS43 - ellipsoid plot

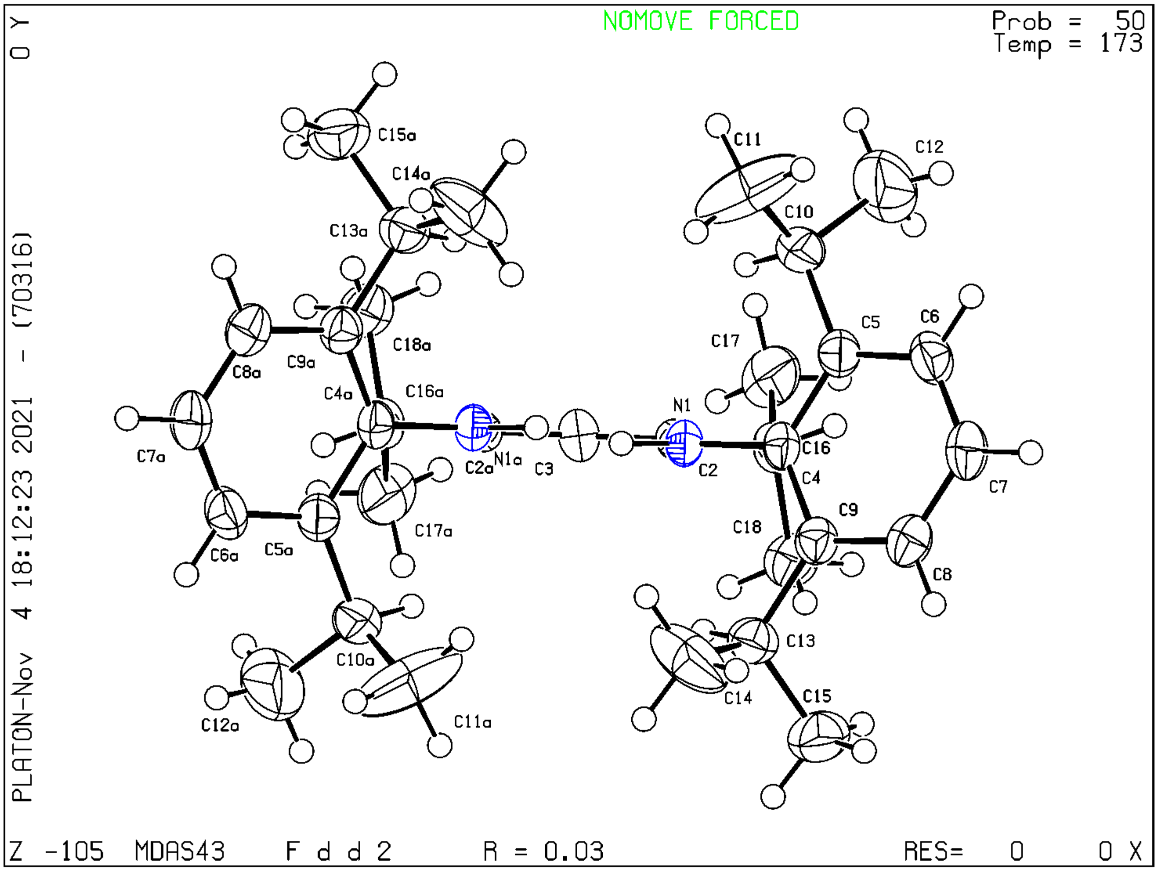

checkCIF/PLATON report

You have not supplied any structure factors. As a result the full set of tests cannot be run.

THIS REPORT IS FOR GUIDANCE ONLY. IF USED AS PART OF A REVIEW PROCEDURE FOR PUBLICATION, IT SHOULD NOT REPLACE THE EXPERTISE OF AN EXPERIENCED CRYSTALLOGRAPHIC REFEREE.

No syntax errors found. CIF dictionary Interpreting this report

Datablock: ASSB77

Bond precision: C-C = 0.0030 A Wavelength=1.54184

Cell:	a=16.9852(5)	b=42.3795(13)	c=8.3707(2)
	alpha=90	beta=90	gamma=90
Temperature:	173 K		

	Calculated	Reported
Volume	6025.4(3)	6025.4(3)
Space group	F d d 2	F d d 2
Hall group	F 2 -2d	F 2 -2d
Moiety formula	C33 H50 N2	C33 H50 N2
Sum formula	C33 H50 N2	C33 H50 N2
Mr	474.75	474.77
Dx,g cm-3	1.047	1.047
Z	8	8
Mu (mm-1)	0.444	0.444
F000	2096.0	2096.0
F000’	2100.96	
h,k,lmax	21,52,10	21,52,10
Nref	3121[1672]	2737
Tmin,Tmax	0.957,0.957	0.737,0.957
Tmin’	0.957	

Correction method= # Reported T Limits: Tmin=0.737 Tmax=0.957
AbsCorr = MULTI-SCAN

Data completeness= 1.64/0.88 Theta(max)= 75.420

R(reflections)= 0.0383(2723) wR2(reflections)= 0.0989(2737)

S = 1.055 Npar= 170

The following ALERTS were generated. Each ALERT has the format
test-name_ALERT_alert-type_alert-level.
Click on the hyperlinks for more details of the test.

Alert level C

```
PLAT220_ALERT_2_C NonSolvent   Resd 1  C   Ueq(max)/Ueq(min) Range        5.0 Ratio
PLAT222_ALERT_3_C NonSolvent Resd 1  H   Uiso(max)/Uiso(min) Range        4.2 Ratio
PLAT242_ALERT_2_C Low    'MainMol' Ueq as Compared to Neighbors of        C10 Check
PLAT242_ALERT_2_C Low    'MainMol' Ueq as Compared to Neighbors of        C13 Check
```

Alert level G

```
CHEMS02_ALERT_1_G  Please check that you have entered the correct
            _publ_requested_category classification of your compound;
            FI or CI or EI for inorganic; FM or CM or EM for metal-organic;
            FO or CO or EO for organic.
            From the CIF: _publ_requested_category   CHOOSE FI FM FO CI CM CO or A
            From the CIF: _chemical_formula_sum :C33 H50 N2
PLAT002_ALERT_2_G Number of Distance or Angle Restraints on AtSite          2 Note
PLAT172_ALERT_4_G The CIF-Embedded .res File Contains DFIX Records          1 Report
PLAT300_ALERT_4_G Atom Site Occupancy of H1         Constrained at        0.5 Check
PLAT860_ALERT_3_G Number of Least-Squares Restraints ............           2 Note
PLAT933_ALERT_2_G Number of OMIT Records in Embedded .res File ...          2 Note
```

0 **ALERT level A** = Most likely a serious problem - resolve or explain
0 **ALERT level B** = A potentially serious problem, consider carefully
4 **ALERT level C** = Check. Ensure it is not caused by an omission or oversight
6 **ALERT level G** = General information/check it is not something unexpected

1 ALERT type 1 CIF construction/syntax error, inconsistent or missing data
5 ALERT type 2 Indicator that the structure model may be wrong or deficient
2 ALERT type 3 Indicator that the structure quality may be low
2 ALERT type 4 Improvement, methodology, query or suggestion
0 ALERT type 5 Informative message, check

It is advisable to attempt to resolve as many as possible of the alerts in all categories. Often the minor alerts point to easily fixed oversights, errors and omissions in your CIF or refinement strategy, so attention to these fine details can be worthwhile. In order to resolve some of the more serious problems it may be necessary to carry out additional measurements or structure refinements. However, the purpose of your study may justify the reported deviations and the more serious of these should normally be commented upon in the discussion or experimental section of a paper or in the "special_details" fields of the CIF. checkCIF was carefully designed to identify outliers and unusual parameters, but every test has its limitations and alerts that are not important in a particular case may appear. Conversely, the absence of alerts does not guarantee there are no aspects of the results needing attention. It is up to the individual to critically assess their own results and, if necessary, seek expert advice.

Publication of your CIF in IUCr journals

A basic structural check has been run on your CIF. These basic checks will be run on all CIFs submitted for publication in IUCr journals (*Acta Crystallographica*, *Journal of Applied Crystallography*, *Journal of Synchrotron Radiation*); however, if you intend to submit to *Acta Crystallographica Section C* or *E* or *IUCrData*, you should make sure that full publication checks are run on the final version of your CIF prior to submission.

Publication of your CIF in other journals

Please refer to the *Notes for Authors* of the relevant journal for any special instructions relating to CIF submission.

PLATON version of 13/07/2021; check.def file version of 13/07/2021

Datablock ASSB77 - ellipsoid plot

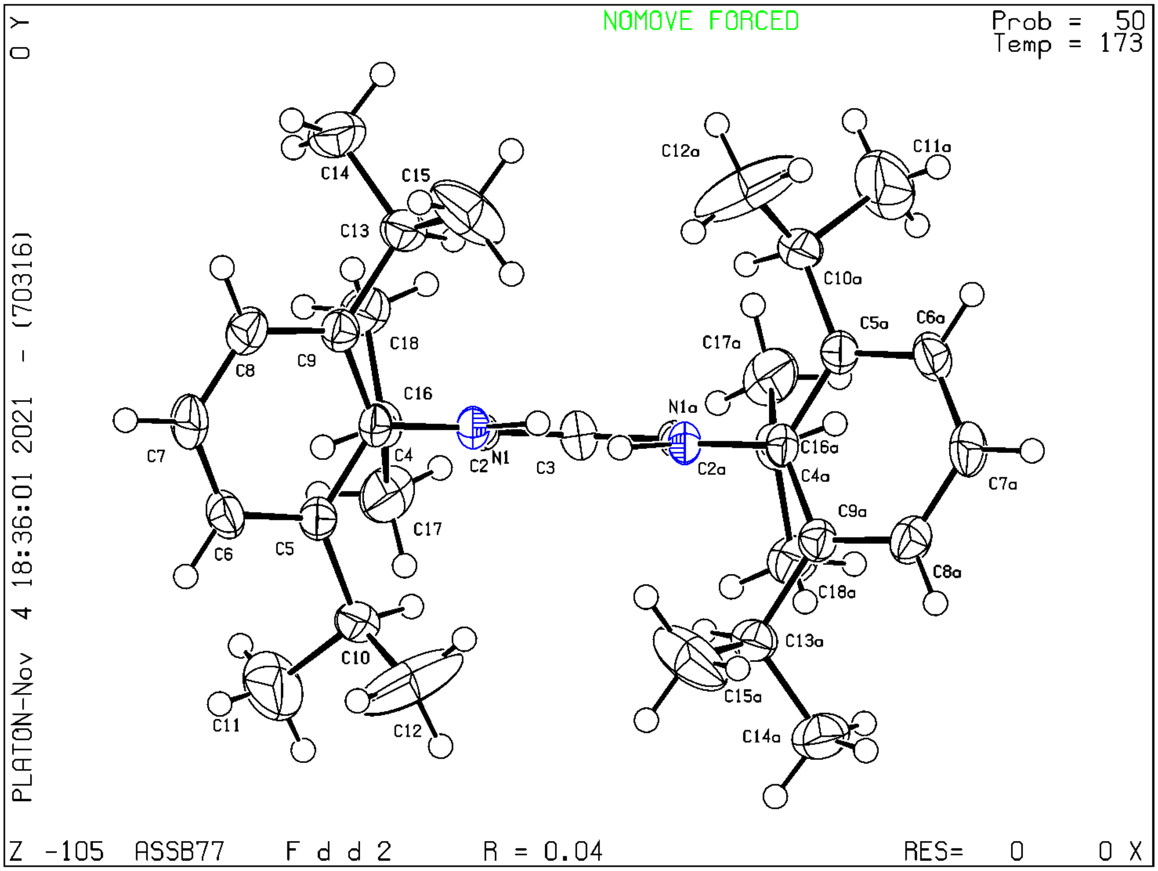

checkCIF/PLATON report

Structure factors have been supplied for datablock(s) 58q

THIS REPORT IS FOR GUIDANCE ONLY. IF USED AS PART OF A REVIEW PROCEDURE FOR PUBLICATION, IT SHOULD NOT REPLACE THE EXPERTISE OF AN EXPERIENCED CRYSTALLOGRAPHIC REFEREE.

No syntax errors found. CIF dictionary Interpreting this report

Datablock: 58q

```
Bond precision:    C-C = 0.0024 A            Wavelength=0.71075

Cell:               a=16.9412(19)      b=11.1598(10)      c=24.343(3)
                    alpha=90           beta=90            gamma=90
Temperature:        173 K

                  Calculated                       Reported
Volume            4602.3(9)                        4602.3(9)
Space group       P n m a                          P n m a
Hall group        -P 2ac 2n                        -P 2ac 2n
Moiety formula    C37 H58 Mg N2, 2(C6 H6)          ?
Sum formula       C49 H70 Mg N2                    C49 H70 Mg N2
Mr                711.38                           711.38
Dx,g cm-3         1.027                            1.027
Z                 4                                4
Mu (mm-1)         0.071                            0.070
F000              1560.0                           1560.0
F000'             1560.62
h,k,lmax          20,13,29                         20,13,29
Nref              4459                             4452
Tmin,Tmax         0.992,0.998                      0.952,0.998
Tmin'             0.990

Correction method= # Reported T Limits: Tmin=0.952 Tmax=0.998
AbsCorr = MULTI-SCAN

Data completeness= 0.998            Theta(max)= 25.370

                                                      wR2(reflections)=
R(reflections)= 0.0394( 3840)                         0.1172( 4452)

S = 1.056                    Npar= 293
```

The following ALERTS were generated. Each ALERT has the format
test-name_ALERT_alert-type_alert-level.
Click on the hyperlinks for more details of the test.

Alert level C

```
PLAT260_ALERT_2_C Large Average Ueq of Residue Including       C34      0.135 Check
PLAT331_ALERT_2_C Small Aver Phenyl C-C Dist C31      --C31_b    .       1.36 Ang.
```

Alert level G

```
PLAT002_ALERT_2_G Number of Distance or Angle Restraints on AtSite          7 Note
PLAT171_ALERT_4_G The CIF-Embedded .res File Contains EADP Records          3 Report
PLAT172_ALERT_4_G The CIF-Embedded .res File Contains DFIX Records          3 Report
PLAT300_ALERT_4_G Atom Site Occupancy of C25        Constrained at       0.25 Check
PLAT300_ALERT_4_G Atom Site Occupancy of C25A       Constrained at       0.25 Check
PLAT300_ALERT_4_G Atom Site Occupancy of C26        Constrained at       0.25 Check
PLAT300_ALERT_4_G Atom Site Occupancy of C26A       Constrained at       0.25 Check
PLAT300_ALERT_4_G Atom Site Occupancy of C27        Constrained at       0.25 Check
PLAT300_ALERT_4_G Atom Site Occupancy of C27A       Constrained at       0.25 Check
PLAT300_ALERT_4_G Atom Site Occupancy of H25A       Constrained at       0.25 Check
PLAT300_ALERT_4_G Atom Site Occupancy of H25B       Constrained at       0.25 Check
PLAT300_ALERT_4_G Atom Site Occupancy of H25C       Constrained at       0.25 Check
PLAT300_ALERT_4_G Atom Site Occupancy of H25D       Constrained at       0.25 Check
PLAT300_ALERT_4_G Atom Site Occupancy of H26A       Constrained at       0.25 Check
PLAT300_ALERT_4_G Atom Site Occupancy of H26B       Constrained at       0.25 Check
PLAT300_ALERT_4_G Atom Site Occupancy of H26C       Constrained at       0.25 Check
PLAT300_ALERT_4_G Atom Site Occupancy of H26D       Constrained at       0.25 Check
PLAT300_ALERT_4_G Atom Site Occupancy of H27A       Constrained at       0.25 Check
PLAT300_ALERT_4_G Atom Site Occupancy of H27B       Constrained at       0.25 Check
PLAT300_ALERT_4_G Atom Site Occupancy of H27C       Constrained at       0.25 Check
PLAT300_ALERT_4_G Atom Site Occupancy of H27D       Constrained at       0.25 Check
PLAT300_ALERT_4_G Atom Site Occupancy of H27E       Constrained at       0.25 Check
PLAT300_ALERT_4_G Atom Site Occupancy of H27F       Constrained at       0.25 Check
PLAT301_ALERT_3_G Main Residue  Disorder ..............(Resd  1  )          8% Note
PLAT410_ALERT_2_G Short Intra H...H Contact  H24       ..H25D     .       2.11 Ang.
                                                    x,1/2-y,z  =      8_565 Check
PLAT789_ALERT_4_G Atoms with Negative _atom_site_disorder_group  #         20 Check
PLAT860_ALERT_3_G Number of Least-Squares Restraints ............           6 Note
PLAT883_ALERT_1_G No Info/Value for _atom_sites_solution_primary .     Please Do !
PLAT912_ALERT_4_G Missing # of FCF Reflections Above STh/L=  0.600          6 Note
PLAT913_ALERT_3_G Missing # of Very Strong Reflections in FCF ....          1 Note
PLAT978_ALERT_2_G Number C-C Bonds with Positive Residual Density.         10 Info
```

0 **ALERT level A** = Most likely a serious problem - resolve or explain
0 **ALERT level B** = A potentially serious problem, consider carefully
2 **ALERT level C** = Check. Ensure it is not caused by an omission or oversight
31 **ALERT level G** = General information/check it is not something unexpected

1 ALERT type 1 CIF construction/syntax error, inconsistent or missing data
5 ALERT type 2 Indicator that the structure model may be wrong or deficient
3 ALERT type 3 Indicator that the structure quality may be low
24 ALERT type 4 Improvement, methodology, query or suggestion
0 ALERT type 5 Informative message, check

It is advisable to attempt to resolve as many as possible of the alerts in all categories. Often the minor alerts point to easily fixed oversights, errors and omissions in your CIF or refinement strategy, so attention to these fine details can be worthwhile. In order to resolve some of the more serious problems it may be necessary to carry out additional measurements or structure refinements. However, the purpose of your study may justify the reported deviations and the more serious of these should normally be commented upon in the discussion or experimental section of a paper or in the "special_details" fields of the CIF. checkCIF was carefully designed to identify outliers and unusual parameters, but every test has its limitations and alerts that are not important in a particular case may appear. Conversely, the absence of alerts does not guarantee there are no aspects of the results needing attention. It is up to the individual to critically assess their own results and, if necessary, seek expert advice.

Publication of your CIF in IUCr journals

A basic structural check has been run on your CIF. These basic checks will be run on all CIFs submitted for publication in IUCr journals (*Acta Crystallographica*, *Journal of Applied Crystallography*, *Journal of Synchrotron Radiation*); however, if you intend to submit to *Acta Crystallographica Section C* or *E* or *IUCrData*, you should make sure that full publication checks are run on the final version of your CIF prior to submission.

Publication of your CIF in other journals

Please refer to the *Notes for Authors* of the relevant journal for any special instructions relating to CIF submission.

PLATON version of 20/01/2022; check.def file version of 19/01/2022

Datablock 58q - ellipsoid plot

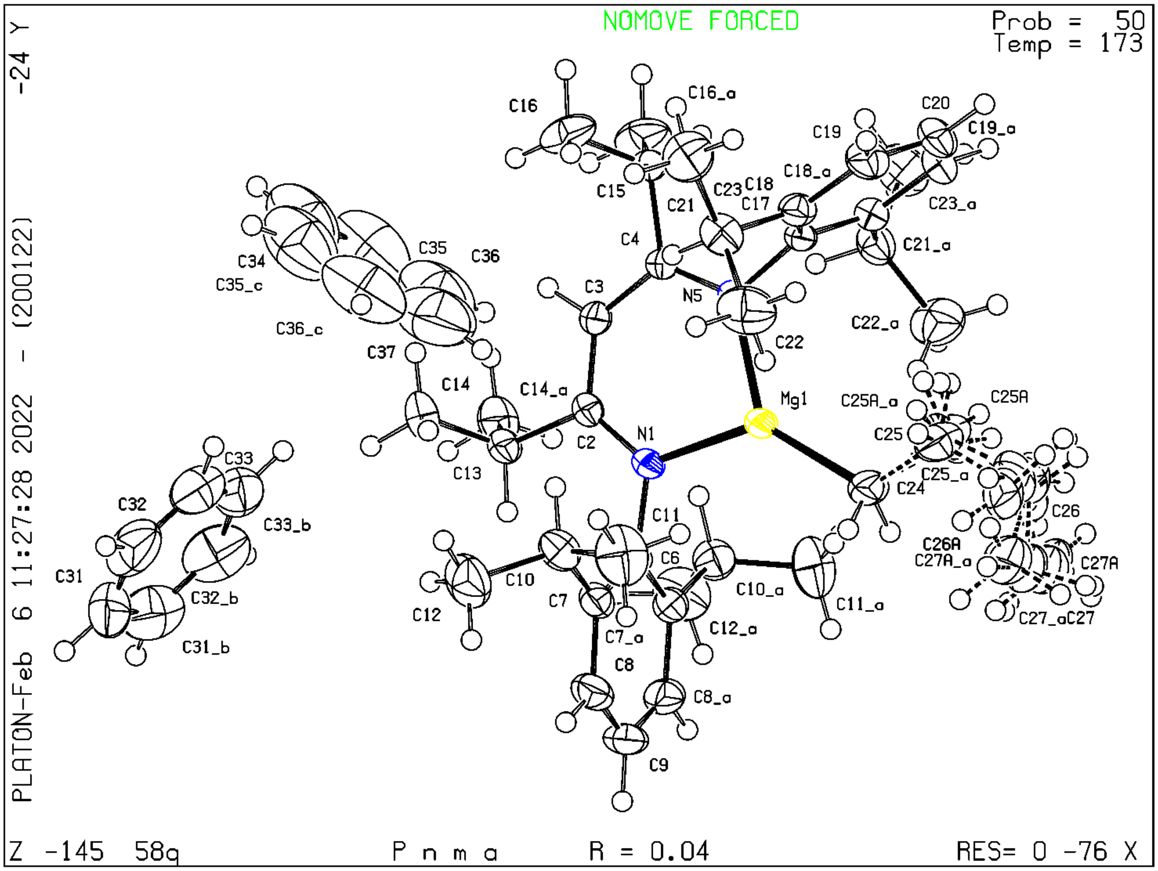

checkCIF/PLATON report

You have not supplied any structure factors. As a result the full set of tests cannot be run.

THIS REPORT IS FOR GUIDANCE ONLY. IF USED AS PART OF A REVIEW PROCEDURE FOR PUBLICATION, IT SHOULD NOT REPLACE THE EXPERTISE OF AN EXPERIENCED CRYSTALLOGRAPHIC REFEREE.

No syntax errors found. CIF dictionary Interpreting this report

Datablock: 51

Bond precision: C-C = 0.0049 A Wavelength=0.71075

Cell:	a=12.1464(14)	b=12.9556(12)	c=14.577(1)
	alpha=66.436(7)	beta=77.284(9)	gamma=87.734(10)
Temperature:	93 K		

	Calculated	Reported
Volume	2048.0(4)	2048.0(4)
Space group	P -1	P -1
Hall group	-P 1	-P 1
Moiety formula	C74 H116 Mg2 N4 O2, 2(C6 H14)	C43 H72 Mg N2 O
Sum formula	C86 H144 Mg2 N4 O2	C43 H72 Mg N2 O
Mr	1314.67	657.36
Dx,g cm-3	1.066	1.066
Z	1	2
Mu (mm-1)	0.076	0.076
F000	728.0	728.0
F000'	728.30	
h,k,lmax	14,15,17	14,15,17
Nref	7532	7508
Tmin,Tmax	0.992,0.996	0.679,0.996
Tmin'	0.992	

Correction method= # Reported T Limits: Tmin=0.679 Tmax=0.996
AbsCorr = MULTI-SCAN

Data completeness= 0.997 Theta(max)= 25.369

R(reflections)= 0.0751(6088) wR2(reflections)= 0.2323(7508)

S = 1.056 Npar= 439

The following ALERTS were generated. Each ALERT has the format
test-name_ALERT_alert-type_alert-level.
Click on the hyperlinks for more details of the test.

Alert level C

```
PLAT243_ALERT_4_C High   'Solvent' Ueq as Compared to Neighbors of        C45 Check
PLAT244_ALERT_4_C Low    'Solvent' Ueq as Compared to Neighbors of        C44 Check
PLAT250_ALERT_2_C Large U3/U1 Ratio for Average U(i,j) Tensor ....        3.1 Note
PLAT260_ALERT_2_C Large Average Ueq of Residue Including       C41      0.161 Check
PLAT340_ALERT_3_C Low Bond Precision on  C-C Bonds ...............     0.0049 Ang.
PLAT360_ALERT_2_C Short  C(sp3)-C(sp3) Bond  C41      - C42      .       1.43 Ang.
PLAT360_ALERT_2_C Short  C(sp3)-C(sp3) Bond  C43      - C44      .       1.37 Ang.
PLAT360_ALERT_2_C Short  C(sp3)-C(sp3) Bond  C44      - C45      .       1.38 Ang.
PLAT360_ALERT_2_C Short  C(sp3)-C(sp3) Bond  C45      - C46      .       1.36 Ang.
```

Alert level G

```
PLAT042_ALERT_1_G Calc. and Reported Moiety Formula Strings Differ     Please Check
PLAT045_ALERT_1_G Calculated and Reported Z Differ by a Factor ...       0.50 Check
PLAT072_ALERT_2_G SHELXL First  Parameter in WGHT  Unusually Large       0.15 Report
```

0 **ALERT level A** = Most likely a serious problem - resolve or explain
0 **ALERT level B** = A potentially serious problem, consider carefully
9 **ALERT level C** = Check. Ensure it is not caused by an omission or oversight
3 **ALERT level G** = General information/check it is not something unexpected

2 ALERT type 1 CIF construction/syntax error, inconsistent or missing data
7 ALERT type 2 Indicator that the structure model may be wrong or deficient
1 ALERT type 3 Indicator that the structure quality may be low
2 ALERT type 4 Improvement, methodology, query or suggestion
0 ALERT type 5 Informative message, check

It is advisable to attempt to resolve as many as possible of the alerts in all categories. Often the minor alerts point to easily fixed oversights, errors and omissions in your CIF or refinement strategy, so attention to these fine details can be worthwhile. In order to resolve some of the more serious problems it may be necessary to carry out additional measurements or structure refinements. However, the purpose of your study may justify the reported deviations and the more serious of these should normally be commented upon in the discussion or experimental section of a paper or in the "special_details" fields of the CIF. checkCIF was carefully designed to identify outliers and unusual parameters, but every test has its limitations and alerts that are not important in a particular case may appear. Conversely, the absence of alerts does not guarantee there are no aspects of the results needing attention. It is up to the individual to critically assess their own results and, if necessary, seek expert advice.

Publication of your CIF in IUCr journals

A basic structural check has been run on your CIF. These basic checks will be run on all CIFs submitted for publication in IUCr journals (*Acta Crystallographica*, *Journal of Applied Crystallography*, *Journal of Synchrotron Radiation*); however, if you intend to submit to *Acta Crystallographica Section C* or *E* or *IUCrData*, you should make sure that full publication checks are run on the final version of your CIF prior to submission.

Publication of your CIF in other journals

Please refer to the *Notes for Authors* of the relevant journal for any special instructions relating to CIF submission.

PLATON version of 13/07/2021; check.def file version of 13/07/2021

Datablock 51 - ellipsoid plot

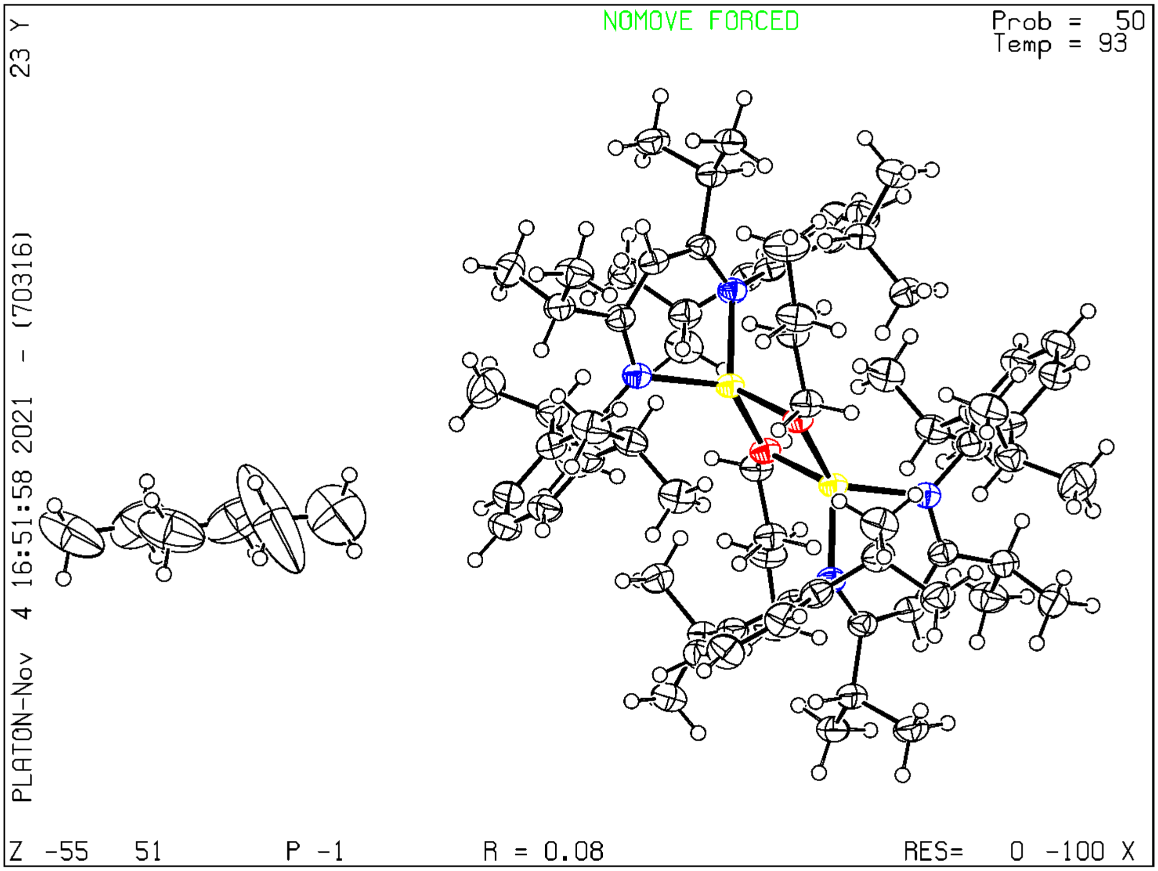

checkCIF/PLATON report

You have not supplied any structure factors. As a result the full set of tests cannot be run.

THIS REPORT IS FOR GUIDANCE ONLY. IF USED AS PART OF A REVIEW PROCEDURE FOR PUBLICATION, IT SHOULD NOT REPLACE THE EXPERTISE OF AN EXPERIENCED CRYSTALLOGRAPHIC REFEREE.

No syntax errors found. CIF dictionary Interpreting this report

Datablock: 68

```
Bond precision:   C-C = 0.0076 A          Wavelength=0.71075

Cell:            a=14.2924(19)    b=25.706(3)        c=10.8234(16)
                 alpha=90         beta=103.041(4)    gamma=90
Temperature:     173 K

                Calculated                    Reported
Volume          3874.0(9)                     3874.0(9)
Space group     P 21/c                        P 1 21/c 1
Hall group      -P 2ybc                       -P 2ybc
Moiety formula  C66 H98 I2 Mg2 N4, 2(C7 H8) C80 H114 I2 Mg2 N4
Sum formula     C80 H114 I2 Mg2 N4            C80 H114 I2 Mg2 N4
Mr              1434.17                       1434.23
Dx,g cm-3       1.230                         1.229
Z               2                             2
Mu (mm-1)       0.870                         0.871
F000            1504.0                        1504.0
F000'           1502.66
h,k,lmax        17,31,13                      17,30,13
Nref            7126                          7102
Tmin,Tmax       0.957,0.957                   0.676,0.957
Tmin'           0.957

Correction method= # Reported T Limits: Tmin=0.676 Tmax=0.957
AbsCorr = MULTI-SCAN

Data completeness= 0.997          Theta(max)= 25.396

                                                  wR2(reflections)=
R(reflections)= 0.0446( 5257)                     0.1330( 7102)

S = 0.803                  Npar= 410
```

The following ALERTS were generated. Each ALERT has the format
test-name_ALERT_alert-type_alert-level.
Click on the hyperlinks for more details of the test.

Alert level C

```
PLAT220_ALERT_2_C NonSolvent   Resd 1  C   Ueq(max)/Ueq(min) Range        3.6 Ratio
PLAT242_ALERT_2_C Low    'MainMol' Ueq as Compared to Neighbors of        C33 Check
```

Alert level G

```
PLAT042_ALERT_1_G Calc. and Reported Moiety Formula Strings Differ     Please Check
PLAT083_ALERT_2_G SHELXL Second Parameter in WGHT  Unusually Large      13.35 Why ?
PLAT380_ALERT_4_G Incorrectly? Oriented X(sp2)-Methyl Moiety .....        C36 Check
```

0 **ALERT level A** = Most likely a serious problem - resolve or explain
0 **ALERT level B** = A potentially serious problem, consider carefully
2 **ALERT level C** = Check. Ensure it is not caused by an omission or oversight
3 **ALERT level G** = General information/check it is not something unexpected

1 ALERT type 1 CIF construction/syntax error, inconsistent or missing data
3 ALERT type 2 Indicator that the structure model may be wrong or deficient
0 ALERT type 3 Indicator that the structure quality may be low
1 ALERT type 4 Improvement, methodology, query or suggestion
0 ALERT type 5 Informative message, check

It is advisable to attempt to resolve as many as possible of the alerts in all categories. Often the minor alerts point to easily fixed oversights, errors and omissions in your CIF or refinement strategy, so attention to these fine details can be worthwhile. In order to resolve some of the more serious problems it may be necessary to carry out additional measurements or structure refinements. However, the purpose of your study may justify the reported deviations and the more serious of these should normally be commented upon in the discussion or experimental section of a paper or in the "special_details" fields of the CIF. checkCIF was carefully designed to identify outliers and unusual parameters, but every test has its limitations and alerts that are not important in a particular case may appear. Conversely, the absence of alerts does not guarantee there are no aspects of the results needing attention. It is up to the individual to critically assess their own results and, if necessary, seek expert advice.

Publication of your CIF in IUCr journals

A basic structural check has been run on your CIF. These basic checks will be run on all CIFs submitted for publication in IUCr journals (*Acta Crystallographica*, *Journal of Applied Crystallography*, *Journal of Synchrotron Radiation*); however, if you intend to submit to *Acta Crystallographica Section C* or *E* or *IUCrData*, you should make sure that full publication checks are run on the final version of your CIF prior to submission.

Publication of your CIF in other journals

Please refer to the *Notes for Authors* of the relevant journal for any special instructions relating to CIF submission.

PLATON version of 13/07/2021; check.def file version of 13/07/2021

Datablock 68 - ellipsoid plot

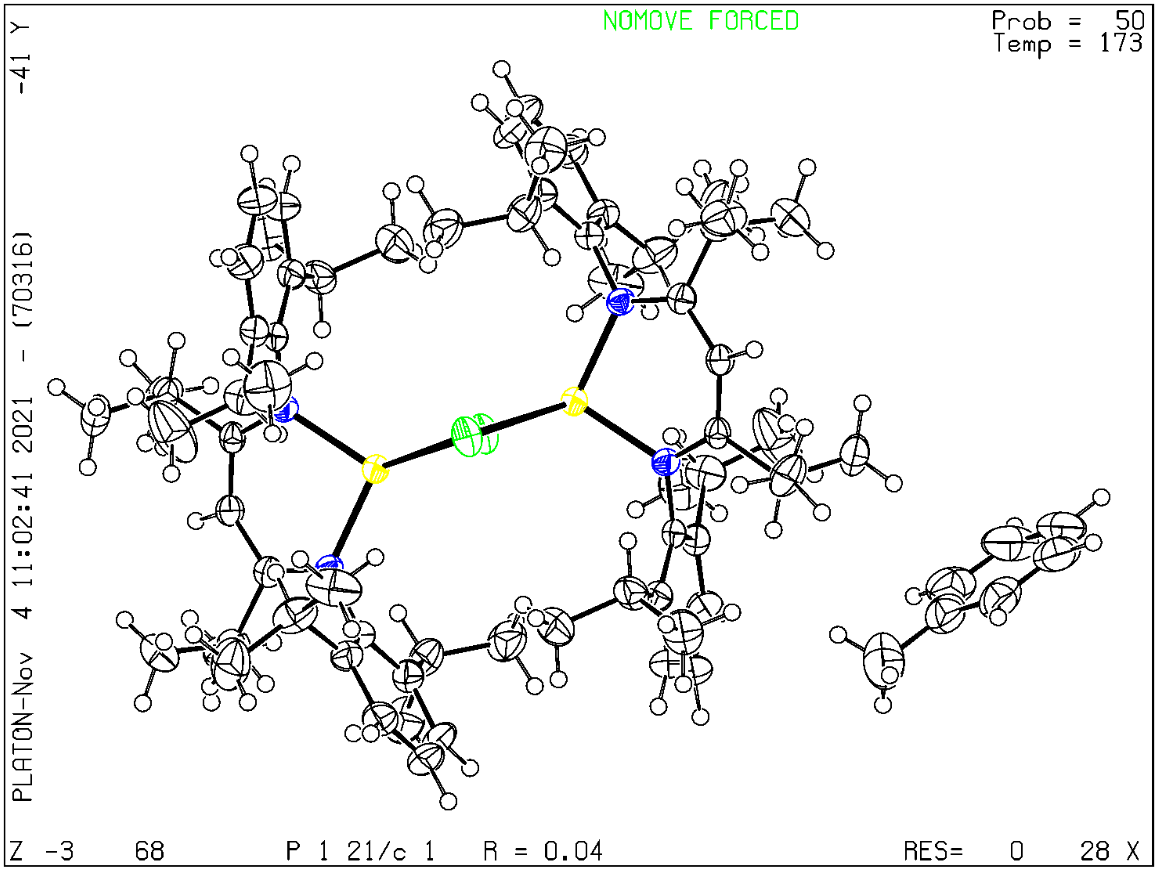

checkCIF/PLATON report

You have not supplied any structure factors. As a result the full set of tests cannot be run.

THIS REPORT IS FOR GUIDANCE ONLY. IF USED AS PART OF A REVIEW PROCEDURE FOR PUBLICATION, IT SHOULD NOT REPLACE THE EXPERTISE OF AN EXPERIENCED CRYSTALLOGRAPHIC REFEREE.

No syntax errors found. CIF dictionary Interpreting this report

Datablock: 110

```
Bond precision:   C-C = 0.0049 A          Wavelength=0.71075

Cell:             a=15.9100(15)     b=9.7639(5)         c=27.790(2)
                  alpha=90          beta=100.771(3)     gamma=90
Temperature:      173 K

                 Calculated                     Reported
Volume           4240.9(5)                      4240.9(5)
Space group      P 21/n                         P 1 21/n 1
Hall group       -P 2yn                         -P 2yn
Moiety formula   C66 H98 I2 Mg2 N4, 4(C6 H6) C90 H122 I2 Mg2 N4
Sum formula      C90 H122 I2 Mg2 N4             C90 H122 I2 Mg2 N4
Mr               1562.34                        1562.40
Dx,g cm-3        1.224                          1.223
Z                2                              2
Mu (mm-1)        0.801                          0.801
F000             1640.0                         1640.0
F000'            1638.71
h,k,lmax         19,11,33                       19,11,33
Nref             7768                           7753
Tmin,Tmax        0.887,0.887                    0.795,0.887
Tmin'            0.887

Correction method= # Reported T Limits: Tmin=0.795 Tmax=0.887
AbsCorr = MULTI-SCAN

Data completeness= 0.998           Theta(max)= 25.351

                                                    wR2(reflections)=
R(reflections)= 0.0317( 7281)                       0.0926( 7753)

S = 1.041                   Npar= 442
```

The following ALERTS were generated. Each ALERT has the format
test-name_ALERT_alert-type_alert-level.
Click on the hyperlinks for more details of the test.

Alert level C

```
PLAT220_ALERT_2_C NonSolvent   Resd 1  C   Ueq(max)/Ueq(min) Range        3.4 Ratio
PLAT242_ALERT_2_C Low    'MainMol' Ueq as Compared to Neighbors of        C12 Check
PLAT250_ALERT_2_C Large U3/U1 Ratio for Average U(i,j) Tensor ....        2.5 Note
PLAT250_ALERT_2_C Large U3/U1 Ratio for Average U(i,j) Tensor ....        3.6 Note
PLAT260_ALERT_2_C Large Average Ueq of Residue Including       C42      0.122 Check
PLAT331_ALERT_2_C Small Aver Phenyl C-C Dist C36      --C41      .       1.37 Ang.
```

Alert level G

```
PLAT042_ALERT_1_G Calc. and Reported Moiety Formula Strings Differ     Please Check
PLAT232_ALERT_2_G Hirshfeld Test Diff (M-X)  I1       --Mg1      .       10.2 s.u.
PLAT232_ALERT_2_G Hirshfeld Test Diff (M-X)  I1       --Mg1_a    .        6.8 s.u.
PLAT779_ALERT_4_G Suspect or Irrelevant (Bond) Angle(s) in CIF ...      40.57 Deg.
              N1   -C2   -MG1     1_555   1_555   1_555 ......     #   32 Check
PLAT779_ALERT_4_G Suspect or Irrelevant (Bond) Angle(s) in CIF ...      40.65 Deg.
              N5   -C4   -MG1     1_555   1_555   1_555 ......     #   41 Check
PLAT933_ALERT_2_G Number of OMIT Records in Embedded .res File ...          2 Note
```

0 **ALERT level A** = Most likely a serious problem - resolve or explain
0 **ALERT level B** = A potentially serious problem, consider carefully
6 **ALERT level C** = Check. Ensure it is not caused by an omission or oversight
6 **ALERT level G** = General information/check it is not something unexpected

1 ALERT type 1 CIF construction/syntax error, inconsistent or missing data
9 ALERT type 2 Indicator that the structure model may be wrong or deficient
0 ALERT type 3 Indicator that the structure quality may be low
2 ALERT type 4 Improvement, methodology, query or suggestion
0 ALERT type 5 Informative message, check

It is advisable to attempt to resolve as many as possible of the alerts in all categories. Often the minor alerts point to easily fixed oversights, errors and omissions in your CIF or refinement strategy, so attention to these fine details can be worthwhile. In order to resolve some of the more serious problems it may be necessary to carry out additional measurements or structure refinements. However, the purpose of your study may justify the reported deviations and the more serious of these should normally be commented upon in the discussion or experimental section of a paper or in the "special_details" fields of the CIF. checkCIF was carefully designed to identify outliers and unusual parameters, but every test has its limitations and alerts that are not important in a particular case may appear. Conversely, the absence of alerts does not guarantee there are no aspects of the results needing attention. It is up to the individual to critically assess their own results and, if necessary, seek expert advice.

Publication of your CIF in IUCr journals

A basic structural check has been run on your CIF. These basic checks will be run on all CIFs submitted for publication in IUCr journals (*Acta Crystallographica*, *Journal of Applied Crystallography*, *Journal of Synchrotron Radiation*); however, if you intend to submit to *Acta Crystallographica Section C* or *E* or *IUCrData*, you should make sure that full publication checks are run on the final version of your CIF prior to submission.

Publication of your CIF in other journals

Please refer to the *Notes for Authors* of the relevant journal for any special instructions relating to CIF submission.

PLATON version of 13/07/2021; check.def file version of 13/07/2021

Datablock 110 - ellipsoid plot

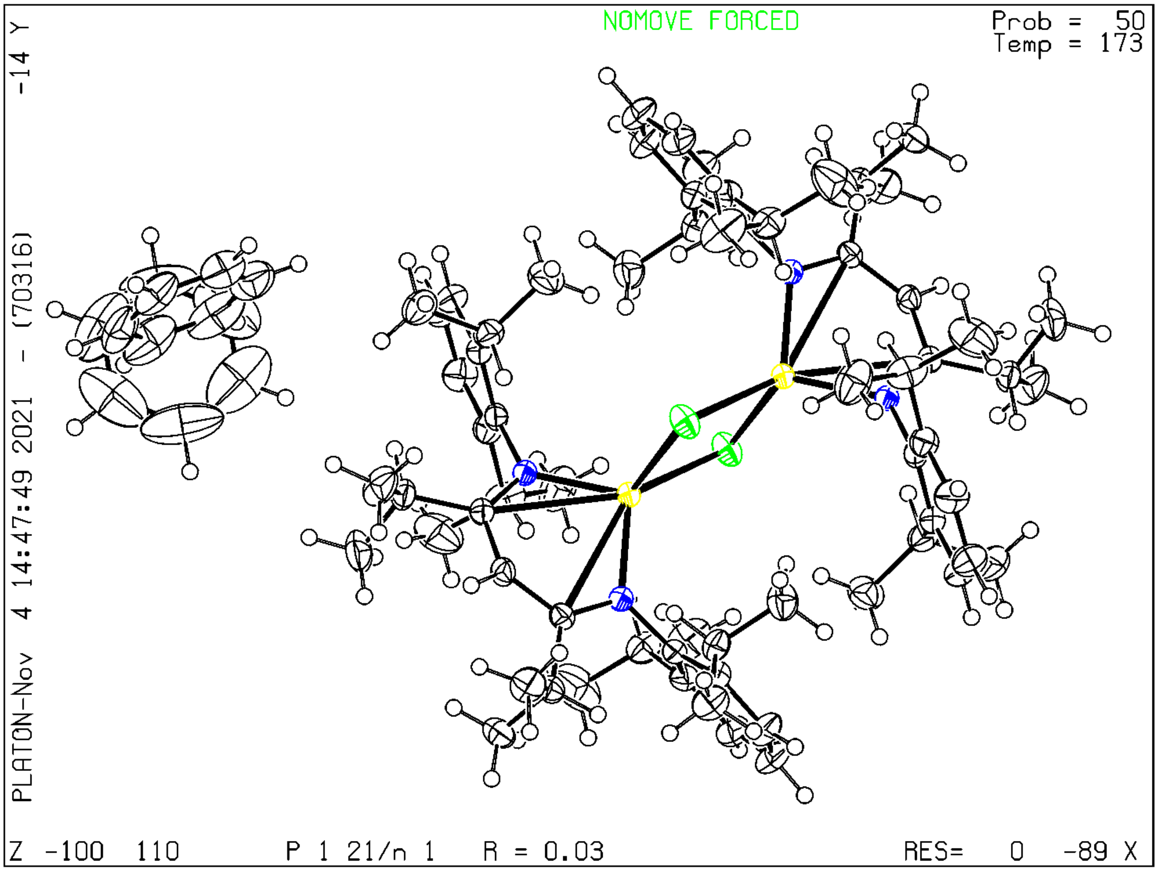

checkCIF/PLATON report

You have not supplied any structure factors. As a result the full set of tests cannot be run.

THIS REPORT IS FOR GUIDANCE ONLY. IF USED AS PART OF A REVIEW PROCEDURE FOR PUBLICATION, IT SHOULD NOT REPLACE THE EXPERTISE OF AN EXPERIENCED CRYSTALLOGRAPHIC REFEREE.

No syntax errors found. CIF dictionary Interpreting this report

Datablock: 76

Bond precision:	C-C = 0.0082 A		Wavelength=0.71075
Cell:	a=12.3655(8)	b=9.9726(7)	c=34.742(2)
	alpha=90	beta=92.480(17)	gamma=90
Temperature:	173 K		

	Calculated	Reported
Volume	4280.2(5)	4280.2(5)
Space group	P 21/c	P 1 21/c 1
Hall group	-P 2ybc	-P 2ybc
Moiety formula	C37 H57 I Mg N2 O, C6 H6	C43 H63 I Mg N2 O
Sum formula	C43 H63 I Mg N2 O	C43 H63 I Mg N2 O
Mr	775.16	775.19
Dx,g cm-3	1.203	1.203
Z	4	4
Mu (mm-1)	0.794	0.795
F000	1632.0	1632.0
F000'	1630.73	
h,k,lmax	16,12,45	16,12,44
Nref	9856	9617
Tmin,Tmax	0.771,0.895	0.599,0.895
Tmin'	0.756	

Correction method= # Reported T Limits: Tmin=0.599 Tmax=0.895
AbsCorr = MULTI-SCAN

Data completeness= 0.976 Theta(max)= 27.533

R(reflections)= 0.0676(6288) wR2(reflections)= 0.1483(9617)

S = 1.110 Npar= 433

The following ALERTS were generated. Each ALERT has the format
test-name_ALERT_alert-type_alert-level.
Click on the hyperlinks for more details of the test.

Alert level C

```
PLAT220_ALERT_2_C NonSolvent   Resd 1  C   Ueq(max)/Ueq(min) Range        4.8 Ratio
PLAT222_ALERT_3_C NonSolvent Resd 1  H   Uiso(max)/Uiso(min) Range        4.1 Ratio
PLAT241_ALERT_2_C High   'MainMol' Ueq as Compared to Neighbors of        C38 Check
PLAT241_ALERT_2_C High   'MainMol' Ueq as Compared to Neighbors of        C39 Check
PLAT242_ALERT_2_C Low    'MainMol' Ueq as Compared to Neighbors of        C15 Check
PLAT260_ALERT_2_C Large Average Ueq of Residue Including       C41      0.108 Check
PLAT342_ALERT_3_C Low Bond Precision on  C-C Bonds ...............    0.00822 Ang.
PLAT360_ALERT_2_C Short  C(sp3)-C(sp3) Bond  C38      - C39      .       1.42 Ang.
```

Alert level G

```
PLAT042_ALERT_1_G Calc. and Reported Moiety Formula Strings Differ     Please Check
PLAT083_ALERT_2_G SHELXL Second Parameter in WGHT  Unusually Large       9.17 Why ?
PLAT779_ALERT_4_G Suspect or Irrelevant (Bond) Angle(s) in CIF ...      39.85 Deg.
              N1   -C2   -MG1     1_555   1_555   1_555 ......     #   23 Check
PLAT790_ALERT_4_G Centre of Gravity not Within Unit Cell: Resd.  #          2 Note
              C6 H6
PLAT941_ALERT_3_G Average HKL Measurement Multiplicity ...........        3.5 Low
```

0 **ALERT level A** = Most likely a serious problem - resolve or explain
0 **ALERT level B** = A potentially serious problem, consider carefully
8 **ALERT level C** = Check. Ensure it is not caused by an omission or oversight
5 **ALERT level G** = General information/check it is not something unexpected

1 ALERT type 1 CIF construction/syntax error, inconsistent or missing data
7 ALERT type 2 Indicator that the structure model may be wrong or deficient
3 ALERT type 3 Indicator that the structure quality may be low
2 ALERT type 4 Improvement, methodology, query or suggestion
0 ALERT type 5 Informative message, check

It is advisable to attempt to resolve as many as possible of the alerts in all categories. Often the minor alerts point to easily fixed oversights, errors and omissions in your CIF or refinement strategy, so attention to these fine details can be worthwhile. In order to resolve some of the more serious problems it may be necessary to carry out additional measurements or structure refinements. However, the purpose of your study may justify the reported deviations and the more serious of these should normally be commented upon in the discussion or experimental section of a paper or in the "special_details" fields of the CIF. checkCIF was carefully designed to identify outliers and unusual parameters, but every test has its limitations and alerts that are not important in a particular case may appear. Conversely, the absence of alerts does not guarantee there are no aspects of the results needing attention. It is up to the individual to critically assess their own results and, if necessary, seek expert advice.

Publication of your CIF in IUCr journals

A basic structural check has been run on your CIF. These basic checks will be run on all CIFs submitted for publication in IUCr journals (*Acta Crystallographica*, *Journal of Applied Crystallography*, *Journal of Synchrotron Radiation*); however, if you intend to submit to *Acta Crystallographica Section C* or *E* or *IUCrData*, you should make sure that full publication checks are run on the final version of your CIF prior to submission.

Publication of your CIF in other journals

Please refer to the *Notes for Authors* of the relevant journal for any special instructions relating to CIF submission.

PLATON version of 13/07/2021; check.def file version of 13/07/2021

Datablock 76 - ellipsoid plot

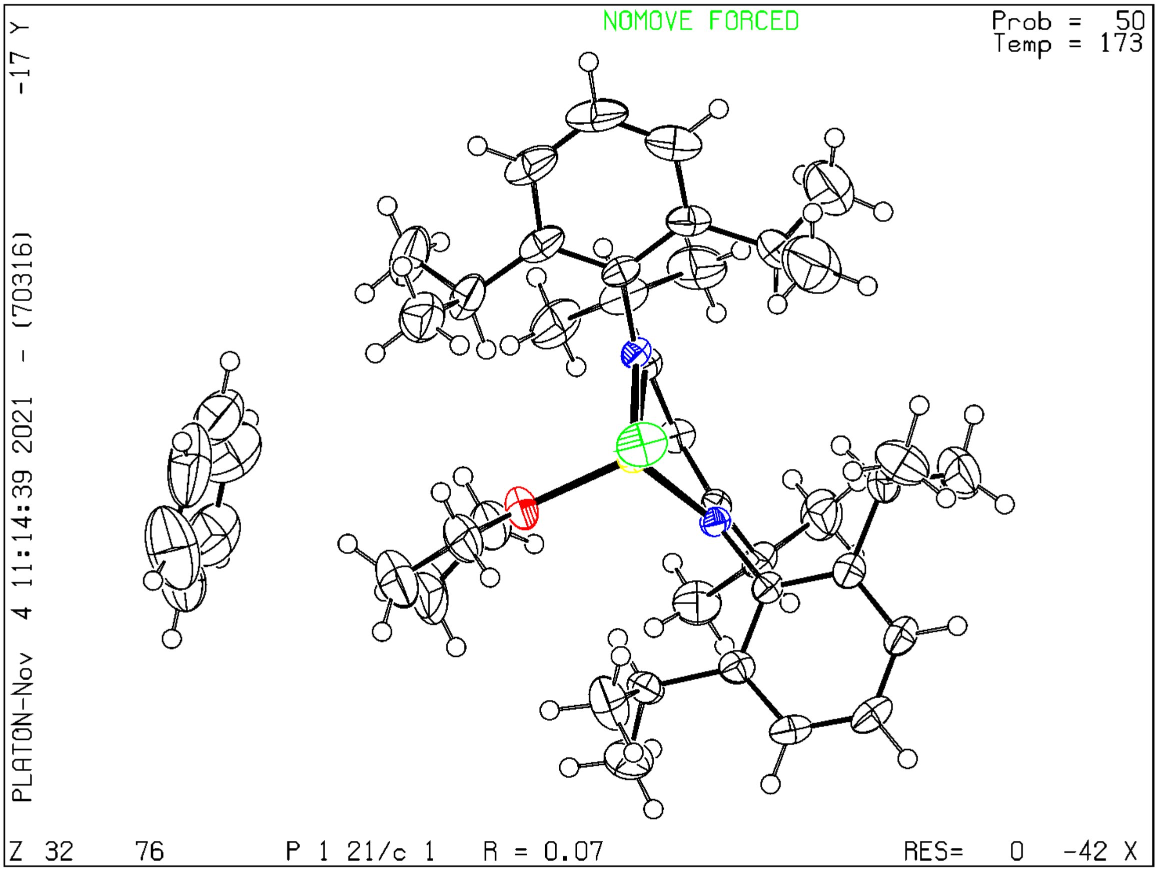

checkCIF/PLATON report

You have not supplied any structure factors. As a result the full set of tests cannot be run.

THIS REPORT IS FOR GUIDANCE ONLY. IF USED AS PART OF A REVIEW PROCEDURE FOR PUBLICATION, IT SHOULD NOT REPLACE THE EXPERTISE OF AN EXPERIENCED CRYSTALLOGRAPHIC REFEREE.

No syntax errors found. CIF dictionary Interpreting this report

Datablock: 43

```
Bond precision:   C-C = 0.0025 A          Wavelength=1.54184

Cell:            a=16.23500(17)     b=16.54720(17)     c=46.6005(5)
                 alpha=90           beta=90            gamma=90
Temperature:     125 K

                Calculated                   Reported
Volume          12518.9(2)                   12518.9(2)
Space group     C 2 2 21                     C 2 2 21
Hall group      C 2c 2                       C 2c 2
Moiety formula  C66 H100 Mg2 N4              C66 H100 Mg2 N4
Sum formula     C66 H100 Mg2 N4              C66 H100 Mg2 N4
Mr              998.12                       998.15
Dx,g cm-3       1.059                        1.059
Z               8                            8
Mu (mm-1)       0.633                        0.634
F000            4384.0                       4384.0
F000'           4396.46
h,k,lmax        20,20,58                     20,20,58
Nref            13010[ 7050]                 12538
Tmin,Tmax       0.927,0.939                  0.895,0.939
Tmin'           0.909

Correction method= # Reported T Limits: Tmin=0.895 Tmax=0.939
AbsCorr = MULTI-SCAN

Data completeness= 1.78/0.96       Theta(max)= 75.506

R(reflections)= 0.0306( 12512)               wR2(reflections)=
                                             0.0820( 12538)
S = 1.075                 Npar= 658
```

The following ALERTS were generated. Each ALERT has the format
test-name_ALERT_alert-type_alert-level.
Click on the hyperlinks for more details of the test.

Alert level G

```
PLAT002_ALERT_2_G Number of Distance or Angle Restraints on AtSite          6 Note
PLAT172_ALERT_4_G The CIF-Embedded .res File Contains DFIX Records          1 Report
PLAT303_ALERT_2_G Full Occupancy Atom H1A       with # Connections       2.00 Check
PLAT303_ALERT_2_G Full Occupancy Atom H1B       with # Connections       2.00 Check
PLAT303_ALERT_2_G Full Occupancy Atom H41A      with # Connections       2.00 Check
PLAT303_ALERT_2_G Full Occupancy Atom H41B      with # Connections       2.00 Check
PLAT860_ALERT_3_G Number of Least-Squares Restraints .............          4 Note
PLAT933_ALERT_2_G Number of OMIT Records in Embedded .res File ...          4 Note
```

0 **ALERT level A** = Most likely a serious problem - resolve or explain
0 **ALERT level B** = A potentially serious problem, consider carefully
0 **ALERT level C** = Check. Ensure it is not caused by an omission or oversight
8 **ALERT level G** = General information/check it is not something unexpected

0 ALERT type 1 CIF construction/syntax error, inconsistent or missing data
6 ALERT type 2 Indicator that the structure model may be wrong or deficient
1 ALERT type 3 Indicator that the structure quality may be low
1 ALERT type 4 Improvement, methodology, query or suggestion
0 ALERT type 5 Informative message, check

It is advisable to attempt to resolve as many as possible of the alerts in all categories. Often the minor alerts point to easily fixed oversights, errors and omissions in your CIF or refinement strategy, so attention to these fine details can be worthwhile. In order to resolve some of the more serious problems it may be necessary to carry out additional measurements or structure refinements. However, the purpose of your study may justify the reported deviations and the more serious of these should normally be commented upon in the discussion or experimental section of a paper or in the "special_details" fields of the CIF. checkCIF was carefully designed to identify outliers and unusual parameters, but every test has its limitations and alerts that are not important in a particular case may appear. Conversely, the absence of alerts does not guarantee there are no aspects of the results needing attention. It is up to the individual to critically assess their own results and, if necessary, seek expert advice.

Publication of your CIF in IUCr journals

A basic structural check has been run on your CIF. These basic checks will be run on all CIFs submitted for publication in IUCr journals (*Acta Crystallographica*, *Journal of Applied Crystallography*, *Journal of Synchrotron Radiation*); however, if you intend to submit to *Acta Crystallographica Section C* or *E* or *IUCrData*, you should make sure that full publication checks are run on the final version of your CIF prior to submission.

Publication of your CIF in other journals

Please refer to the *Notes for Authors* of the relevant journal for any special instructions relating to CIF submission.

PLATON version of 13/07/2021; check.def file version of 13/07/2021

Datablock 43 - ellipsoid plot

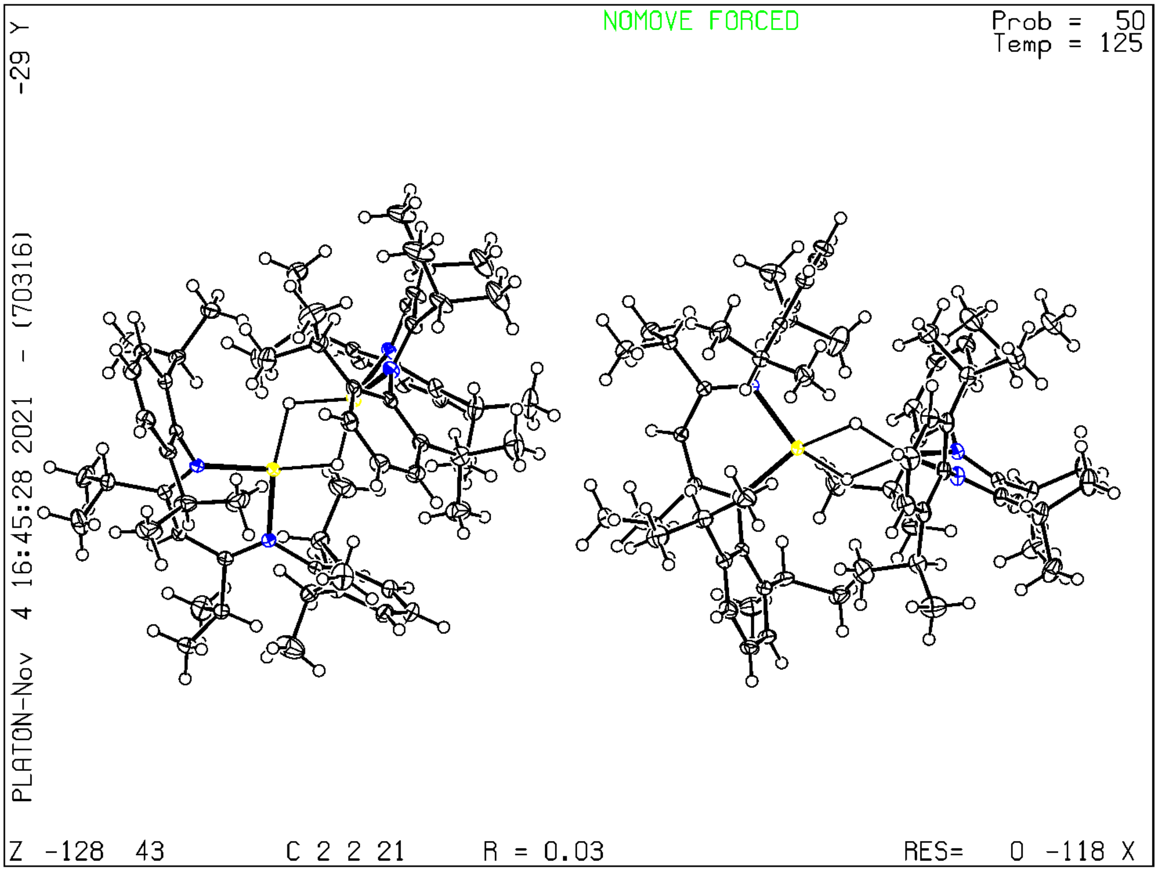

checkCIF/PLATON report

Structure factors have been supplied for datablock(s) 92x

THIS REPORT IS FOR GUIDANCE ONLY. IF USED AS PART OF A REVIEW PROCEDURE FOR PUBLICATION, IT SHOULD NOT REPLACE THE EXPERTISE OF AN EXPERIENCED CRYSTALLOGRAPHIC REFEREE.

No syntax errors found. CIF dictionary Interpreting this report

Datablock: 92x

Bond precision:	C-C = 0.0070 A		Wavelength=0.71075
Cell:	a=15.576(4)	b=19.302(5)	c=25.015(6)
	alpha=90	beta=95.386(5)	gamma=90
Temperature:	173 K		

	Calculated	Reported
Volume	7488(3)	7488(3)
Space group	P 21/c	P 21/c
Hall group	-P 2ybc	-P 2ybc
Moiety formula	C76 H112 Mg2 N4 O, C6 H6	?
Sum formula	C82 H118 Mg2 N4 O	C82 H118 Mg2 N4 O
Mr	1224.43	1224.42
Dx,g cm-3	1.086	1.086
Z	4	4
Mu (mm-1)	0.078	0.078
F000	2680.0	2680.0
F000'	2681.13	
h,k,lmax	18,23,30	18,23,30
Nref	13541	13522
Tmin,Tmax	0.992,0.999	0.786,0.999
Tmin'	0.992	

Correction method= # Reported T Limits: Tmin=0.786 Tmax=0.999
AbsCorr = MULTI-SCAN

Data completeness= 0.999 Theta(max)= 25.243

R(reflections)= 0.0909(7391) wR2(reflections)= 0.1963(13522)

S = 1.100 Npar= 940

The following ALERTS were generated. Each ALERT has the format
test-name_ALERT_alert-type_alert-level.
Click on the hyperlinks for more details of the test.

Alert level C

```
RINTA01_ALERT_3_C  The value of Rint is greater than 0.12
            Rint given   0.144
PLAT020_ALERT_3_C The Value of Rint is Greater Than 0.12 .........      0.144 Report
PLAT220_ALERT_2_C NonSolvent   Resd 1  C   Ueq(max)/Ueq(min) Range        3.2 Ratio
PLAT234_ALERT_4_C Large Hirshfeld Difference C29      --C33A     .       0.18 Ang.
PLAT241_ALERT_2_C High   'MainMol' Ueq as Compared to Neighbors of        C82 Check
PLAT242_ALERT_2_C Low    'MainMol' Ueq as Compared to Neighbors of        C58 Check
PLAT340_ALERT_3_C Low Bond Precision on  C-C Bonds ..............    0.00703 Ang.
PLAT906_ALERT_3_C Large K Value in the Analysis of Variance ......      5.478 Check
PLAT906_ALERT_3_C Large K Value in the Analysis of Variance ......      2.021 Check
PLAT911_ALERT_3_C Missing FCF Refl Between Thmin & STh/L=    0.600         18 Report
```

Alert level G

```
PLAT002_ALERT_2_G Number of Distance or Angle Restraints on AtSite         26 Note
PLAT083_ALERT_2_G SHELXL Second Parameter in WGHT  Unusually Large      16.70 Why ?
PLAT172_ALERT_4_G The CIF-Embedded .res File Contains DFIX Records         10 Report
PLAT173_ALERT_4_G The CIF-Embedded .res File Contains DANG Records          4 Report
PLAT174_ALERT_4_G The CIF-Embedded .res File Contains FLAT Records          2 Report
PLAT300_ALERT_4_G Atom Site Occupancy of C91        Constrained at        0.5 Check
PLAT300_ALERT_4_G Atom Site Occupancy of C92        Constrained at        0.5 Check
PLAT300_ALERT_4_G Atom Site Occupancy of C93        Constrained at        0.5 Check
PLAT300_ALERT_4_G Atom Site Occupancy of C94        Constrained at        0.5 Check
PLAT300_ALERT_4_G Atom Site Occupancy of C95        Constrained at        0.5 Check
PLAT300_ALERT_4_G Atom Site Occupancy of C96        Constrained at        0.5 Check
PLAT300_ALERT_4_G Atom Site Occupancy of H91        Constrained at        0.5 Check
PLAT300_ALERT_4_G Atom Site Occupancy of H92        Constrained at        0.5 Check
PLAT300_ALERT_4_G Atom Site Occupancy of H93        Constrained at        0.5 Check
PLAT300_ALERT_4_G Atom Site Occupancy of H94        Constrained at        0.5 Check
PLAT300_ALERT_4_G Atom Site Occupancy of H95        Constrained at        0.5 Check
PLAT300_ALERT_4_G Atom Site Occupancy of H96        Constrained at        0.5 Check
PLAT300_ALERT_4_G Atom Site Occupancy of C91A       Constrained at        0.5 Check
PLAT300_ALERT_4_G Atom Site Occupancy of C92A       Constrained at        0.5 Check
PLAT300_ALERT_4_G Atom Site Occupancy of C93A       Constrained at        0.5 Check
PLAT300_ALERT_4_G Atom Site Occupancy of C94A       Constrained at        0.5 Check
PLAT300_ALERT_4_G Atom Site Occupancy of C95A       Constrained at        0.5 Check
PLAT300_ALERT_4_G Atom Site Occupancy of C96A       Constrained at        0.5 Check
PLAT300_ALERT_4_G Atom Site Occupancy of H91A       Constrained at        0.5 Check
PLAT300_ALERT_4_G Atom Site Occupancy of H92A       Constrained at        0.5 Check
PLAT300_ALERT_4_G Atom Site Occupancy of H93A       Constrained at        0.5 Check
PLAT300_ALERT_4_G Atom Site Occupancy of H94A       Constrained at        0.5 Check
PLAT300_ALERT_4_G Atom Site Occupancy of H95A       Constrained at        0.5 Check
PLAT300_ALERT_4_G Atom Site Occupancy of H96A       Constrained at        0.5 Check
PLAT301_ALERT_3_G Main Residue  Disorder .............(Resd  1  )          7% Note
PLAT302_ALERT_4_G Anion/Solvent/Minor-Residue Disorder (Resd  2  )       100% Note
PLAT302_ALERT_4_G Anion/Solvent/Minor-Residue Disorder (Resd  3  )       100% Note
PLAT343_ALERT_2_G Unusual sp?      Angle Range in Main Residue for        C76 Check
PLAT367_ALERT_2_G Long?  C(sp?)-C(sp?) Bond  C76      - C77      .       1.52 Ang.
PLAT367_ALERT_2_G Long?  C(sp?)-C(sp?) Bond  C76      - C81      .       1.53 Ang.
PLAT412_ALERT_2_G Short Intra XH3 .. XHn     H22C     ..H34D     .       1.93 Ang.
```

```
                                                  x,y,z  =      1_555 Check
PLAT412_ALERT_2_G Short Intra XH3 .. XHn     H28      ..H35F     .       2.08 Ang.
                                                  x,y,z  =      1_555 Check
PLAT412_ALERT_2_G Short Intra XH3 .. XHn     H35B     ..H81      .       2.05 Ang.
                                                  x,y,z  =      1_555 Check
PLAT412_ALERT_2_G Short Intra XH3 .. XHn     H54A     ..H59A     .       1.93 Ang.
                                                  x,y,z  =      1_555 Check
PLAT413_ALERT_2_G Short Inter XH3 .. XHn     H59C     ..H94A     .       2.10 Ang.
                                             -x,1-y,1-z  =      3_566 Check
PLAT860_ALERT_3_G Number of Least-Squares Restraints ............         42 Note
PLAT883_ALERT_1_G No Info/Value for _atom_sites_solution_primary .     Please Do !
PLAT910_ALERT_3_G Missing # of FCF Reflection(s) Below Theta(Min).          1 Note
PLAT933_ALERT_2_G Number of HKL-OMIT Records in Embedded .res File          9 Note
PLAT965_ALERT_2_G The SHELXL WEIGHT Optimisation has not Converged     Please Check
PLAT967_ALERT_5_G Note: Two-Theta Cutoff Value in Embedded .res ..       50.5 Degree
PLAT978_ALERT_2_G Number C-C Bonds with Positive Residual Density.          0 Info
```

---

  0 **ALERT level A** = Most likely a serious problem - resolve or explain
  0 **ALERT level B** = A potentially serious problem, consider carefully
10 **ALERT level C** = Check. Ensure it is not caused by an omission or oversight
47 **ALERT level G** = General information/check it is not something unexpected

```
 1 ALERT type 1 CIF construction/syntax error, inconsistent or missing data
16 ALERT type 2 Indicator that the structure model may be wrong or deficient
 9 ALERT type 3 Indicator that the structure quality may be low
30 ALERT type 4 Improvement, methodology, query or suggestion
 1 ALERT type 5 Informative message, check
```

It is advisable to attempt to resolve as many as possible of the alerts in all categories. Often the minor alerts point to easily fixed oversights, errors and omissions in your CIF or refinement strategy, so attention to these fine details can be worthwhile. In order to resolve some of the more serious problems it may be necessary to carry out additional measurements or structure refinements. However, the purpose of your study may justify the reported deviations and the more serious of these should normally be commented upon in the discussion or experimental section of a paper or in the "special_details" fields of the CIF. checkCIF was carefully designed to identify outliers and unusual parameters, but every test has its limitations and alerts that are not important in a particular case may appear. Conversely, the absence of alerts does not guarantee there are no aspects of the results needing attention. It is up to the individual to critically assess their own results and, if necessary, seek expert advice.

Publication of your CIF in IUCr journals

A basic structural check has been run on your CIF. These basic checks will be run on all CIFs submitted for publication in IUCr journals (*Acta Crystallographica*, *Journal of Applied Crystallography*, *Journal of Synchrotron Radiation*); however, if you intend to submit to *Acta Crystallographica Section C* or *E* or *IUCrData*, you should make sure that full publication checks are run on the final version of your CIF prior to submission.

Publication of your CIF in other journals

Please refer to the *Notes for Authors* of the relevant journal for any special instructions relating to CIF submission.

PLATON version of 20/01/2022; check.def file version of 19/01/2022

Datablock 92x - ellipsoid plot

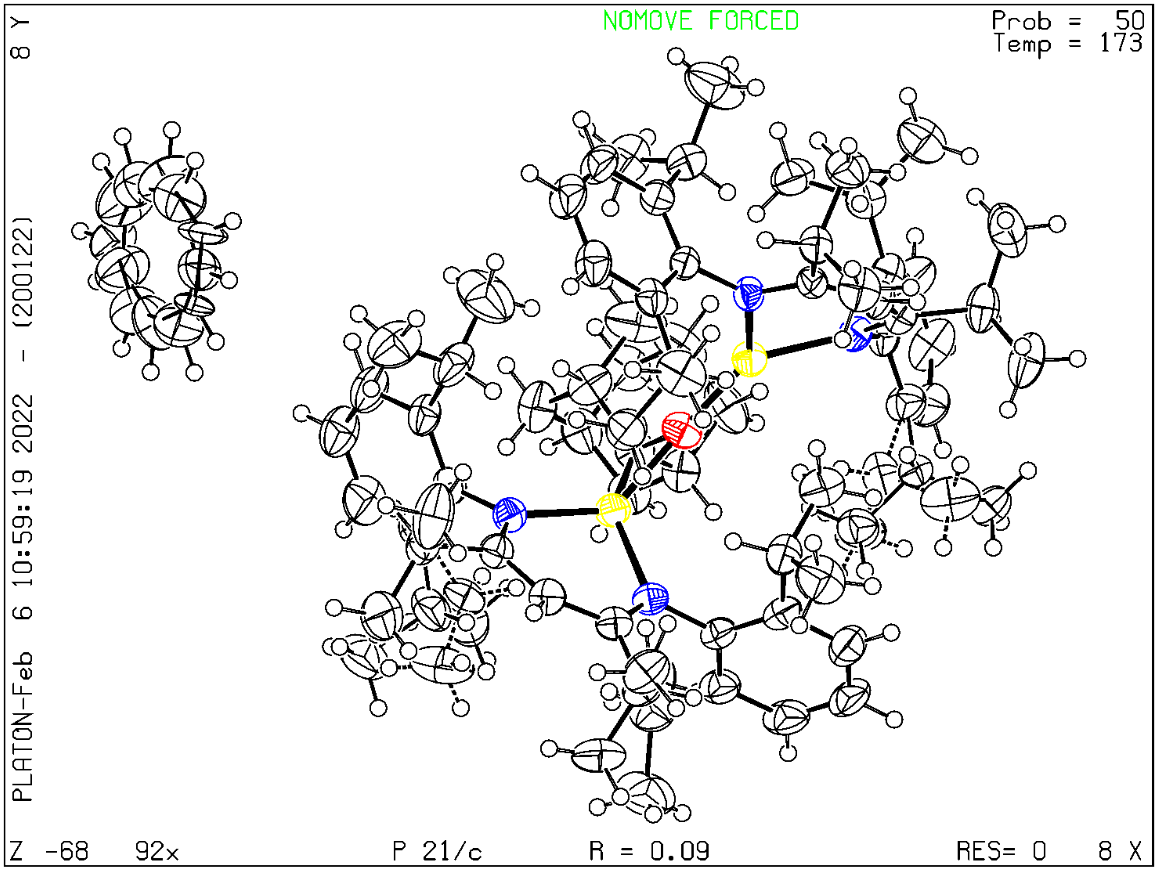

checkCIF/PLATON report

Structure factors have been supplied for datablock(s) 109x

THIS REPORT IS FOR GUIDANCE ONLY. IF USED AS PART OF A REVIEW PROCEDURE FOR PUBLICATION, IT SHOULD NOT REPLACE THE EXPERTISE OF AN EXPERIENCED CRYSTALLOGRAPHIC REFEREE.

No syntax errors found. CIF dictionary Interpreting this report

Datablock: 109x

Bond precision:	C-C = 0.0017 A		Wavelength=1.54184
Cell:	a=12.7474(1)	b=14.2094(1)	c=20.8167(1)
	alpha=90	beta=92.386(1)	gamma=90
Temperature:	125 K		

	Calculated	Reported
Volume	3767.32(4)	3767.32(4)
Space group	P 21/n	P 21/n
Hall group	-P 2yn	-P 2yn
Moiety formula	C74 H114 Mg2 N4 O3, C6 H6	?
Sum formula	C80 H120 Mg2 N4 O3	C80 H120 Mg2 N4 O3
Mr	1234.42	1234.41
Dx,g cm-3	1.088	1.088
Z	2	2
Mu (mm-1)	0.642	0.642
F000	1352.0	1352.0
F000'	1355.89	
h,k,lmax	16,17,26	15,17,25
Nref	7785	7599
Tmin,Tmax	0.968,0.968	0.744,0.968
Tmin'	0.968	

Correction method= # Reported T Limits: Tmin=0.744 Tmax=0.968
AbsCorr = MULTI-SCAN

Data completeness= 0.976 Theta(max)= 75.458

R(reflections)= 0.0395(7181) wR2(reflections)= 0.1092(7599)

S = 1.039 Npar= 453

The following ALERTS were generated. Each ALERT has the format
test-name_ALERT_alert-type_alert-level.
Click on the hyperlinks for more details of the test.

Alert level C

```
PLAT220_ALERT_2_C NonSolvent   Resd 1  C   Ueq(max)/Ueq(min) Range        4.2 Ratio
PLAT222_ALERT_3_C NonSolvent Resd 1  H   Uiso(max)/Uiso(min) Range        4.2 Ratio
PLAT242_ALERT_2_C Low    'MainMol' Ueq as Compared to Neighbors of        C30 Check
PLAT911_ALERT_3_C Missing FCF Refl Between Thmin & STh/L=    0.600         19 Report
```

Alert level G

```
PLAT002_ALERT_2_G Number of Distance or Angle Restraints on AtSite          9 Note
PLAT142_ALERT_4_G s.u. on b - Axis Small or Missing .............     0.00010 Ang.
PLAT143_ALERT_4_G s.u. on c - Axis Small or Missing .............     0.00010 Ang.
PLAT153_ALERT_1_G The s.u.'s on the Cell Axes   are Equal ..(Note)     0.0001 Ang.
PLAT172_ALERT_4_G The CIF-Embedded .res File Contains DFIX Records          4 Report
PLAT230_ALERT_2_G Hirshfeld Test Diff for    C38A     --C39A     .       18.5 s.u.
PLAT232_ALERT_2_G Hirshfeld Test Diff (M-X)  Mg1      --O36      .        5.0 s.u.
PLAT301_ALERT_3_G Main Residue  Disorder .............(Resd  1  )        10% Note
PLAT860_ALERT_3_G Number of Least-Squares Restraints ............          10 Note
PLAT883_ALERT_1_G No Info/Value for _atom_sites_solution_primary .     Please Do !
PLAT912_ALERT_4_G Missing # of FCF Reflections Above STh/L=  0.600        163 Note
PLAT978_ALERT_2_G Number C-C Bonds with Positive Residual Density.         16 Info
PLAT992_ALERT_5_G Repd & Actual _reflns_number_gt Values Differ by          2 Check
```

0 **ALERT level A** = Most likely a serious problem - resolve or explain
0 **ALERT level B** = A potentially serious problem, consider carefully
4 **ALERT level C** = Check. Ensure it is not caused by an omission or oversight
13 **ALERT level G** = General information/check it is not something unexpected

2 ALERT type 1 CIF construction/syntax error, inconsistent or missing data
6 ALERT type 2 Indicator that the structure model may be wrong or deficient
4 ALERT type 3 Indicator that the structure quality may be low
4 ALERT type 4 Improvement, methodology, query or suggestion
1 ALERT type 5 Informative message, check

It is advisable to attempt to resolve as many as possible of the alerts in all categories. Often the minor alerts point to easily fixed oversights, errors and omissions in your CIF or refinement strategy, so attention to these fine details can be worthwhile. In order to resolve some of the more serious problems it may be necessary to carry out additional measurements or structure refinements. However, the purpose of your study may justify the reported deviations and the more serious of these should normally be commented upon in the discussion or experimental section of a paper or in the "special_details" fields of the CIF. checkCIF was carefully designed to identify outliers and unusual parameters, but every test has its limitations and alerts that are not important in a particular case may appear. Conversely, the absence of alerts does not guarantee there are no aspects of the results needing attention. It is up to the individual to critically assess their own results and, if necessary, seek expert advice.

Publication of your CIF in IUCr journals

A basic structural check has been run on your CIF. These basic checks will be run on all CIFs submitted for publication in IUCr journals (*Acta Crystallographica*, *Journal of Applied Crystallography*, *Journal of Synchrotron Radiation*); however, if you intend to submit to *Acta Crystallographica Section C* or *E* or *IUCrData*, you should make sure that full publication checks are run on the final version of your CIF prior to submission.

Publication of your CIF in other journals

Please refer to the *Notes for Authors* of the relevant journal for any special instructions relating to CIF submission.

PLATON version of 20/01/2022; check.def file version of 19/01/2022

Datablock 109x - ellipsoid plot

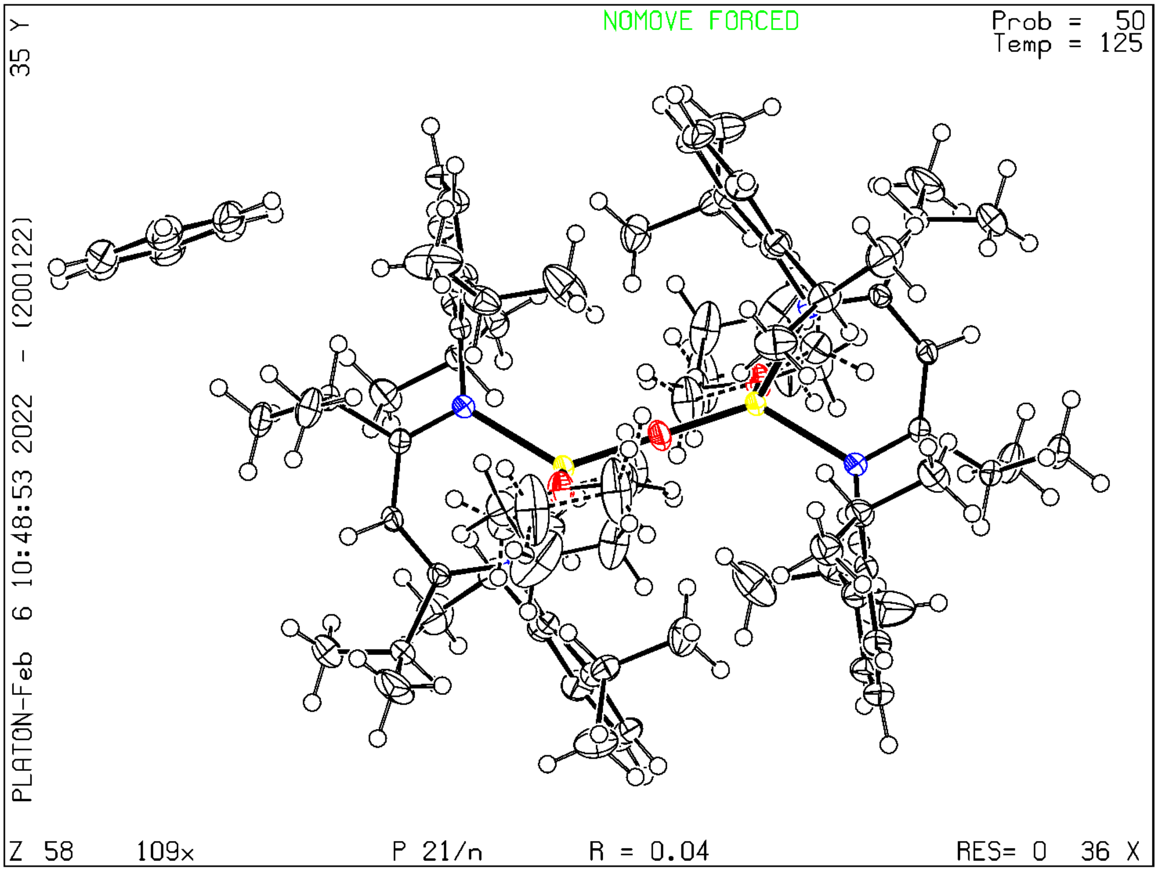

checkCIF/PLATON report

You have not supplied any structure factors. As a result the full set of tests cannot be run.

THIS REPORT IS FOR GUIDANCE ONLY. IF USED AS PART OF A REVIEW PROCEDURE FOR PUBLICATION, IT SHOULD NOT REPLACE THE EXPERTISE OF AN EXPERIENCED CRYSTALLOGRAPHIC REFEREE.

No syntax errors found. CIF dictionary Interpreting this report

Datablock: 114

```
Bond precision:   C-C = 0.0020 A           Wavelength=0.71075

Cell:          a=10.617(4)      b=12.201(5)      c=16.321(7)
               alpha=98.437(7)  beta=102.186(7)  gamma=107.801(5)
Temperature:   93 K

                Calculated                    Reported
Volume          1916.3(14)                    1916.3(14)
Space group     P -1                          P -1
Hall group      -P 1                          -P 1
Moiety formula  C40 H62 Mg N2 O               C40 H62 Mg N2 O
Sum formula     C40 H62 Mg N2 O               C40 H62 Mg N2 O
Mr              611.23                        611.25
Dx,g cm-3       1.059                         1.059
Z               2                             2
Mu (mm-1)       0.077                         0.077
F000            672.0                         672.0
F000'           672.29
h,k,lmax        12,14,19                      12,14,19
Nref            7028                          6999
Tmin,Tmax       0.992,0.992                   0.548,0.992
Tmin'           0.992

Correction method= # Reported T Limits: Tmin=0.548 Tmax=0.992
AbsCorr = MULTI-SCAN

Data completeness= 0.996           Theta(max)= 25.355

R(reflections)= 0.0441( 5935)                     wR2(reflections)=
                                                  0.1379( 6999)
S = 0.989                  Npar= 413
```

The following ALERTS were generated. Each ALERT has the format
test-name_ALERT_alert-type_alert-level.
Click on the hyperlinks for more details of the test.

Alert level C

PLAT230_ALERT_2_C Hirshfeld Test Diff for C30 --C32 . 5.2 s.u.

Alert level G

PLAT072_ALERT_2_G SHELXL First Parameter in WGHT Unusually Large 0.11 Report
PLAT933_ALERT_2_G Number of OMIT Records in Embedded .res File ... 8 Note

0 **ALERT level A** = Most likely a serious problem - resolve or explain
0 **ALERT level B** = A potentially serious problem, consider carefully
1 **ALERT level C** = Check. Ensure it is not caused by an omission or oversight
2 **ALERT level G** = General information/check it is not something unexpected

0 ALERT type 1 CIF construction/syntax error, inconsistent or missing data
3 ALERT type 2 Indicator that the structure model may be wrong or deficient
0 ALERT type 3 Indicator that the structure quality may be low
0 ALERT type 4 Improvement, methodology, query or suggestion
0 ALERT type 5 Informative message, check

It is advisable to attempt to resolve as many as possible of the alerts in all categories. Often the minor alerts point to easily fixed oversights, errors and omissions in your CIF or refinement strategy, so attention to these fine details can be worthwhile. In order to resolve some of the more serious problems it may be necessary to carry out additional measurements or structure refinements. However, the purpose of your study may justify the reported deviations and the more serious of these should normally be commented upon in the discussion or experimental section of a paper or in the "special_details" fields of the CIF. checkCIF was carefully designed to identify outliers and unusual parameters, but every test has its limitations and alerts that are not important in a particular case may appear. Conversely, the absence of alerts does not guarantee there are no aspects of the results needing attention. It is up to the individual to critically assess their own results and, if necessary, seek expert advice.

Publication of your CIF in IUCr journals

A basic structural check has been run on your CIF. These basic checks will be run on all CIFs submitted for publication in IUCr journals (*Acta Crystallographica*, *Journal of Applied Crystallography*, *Journal of Synchrotron Radiation*); however, if you intend to submit to *Acta Crystallographica Section C* or *E* or *IUCrData*, you should make sure that full publication checks are run on the final version of your CIF prior to submission.

Publication of your CIF in other journals

Please refer to the *Notes for Authors* of the relevant journal for any special instructions relating to CIF submission.

PLATON version of 13/07/2021; check.def file version of 13/07/2021

Datablock 114 - ellipsoid plot

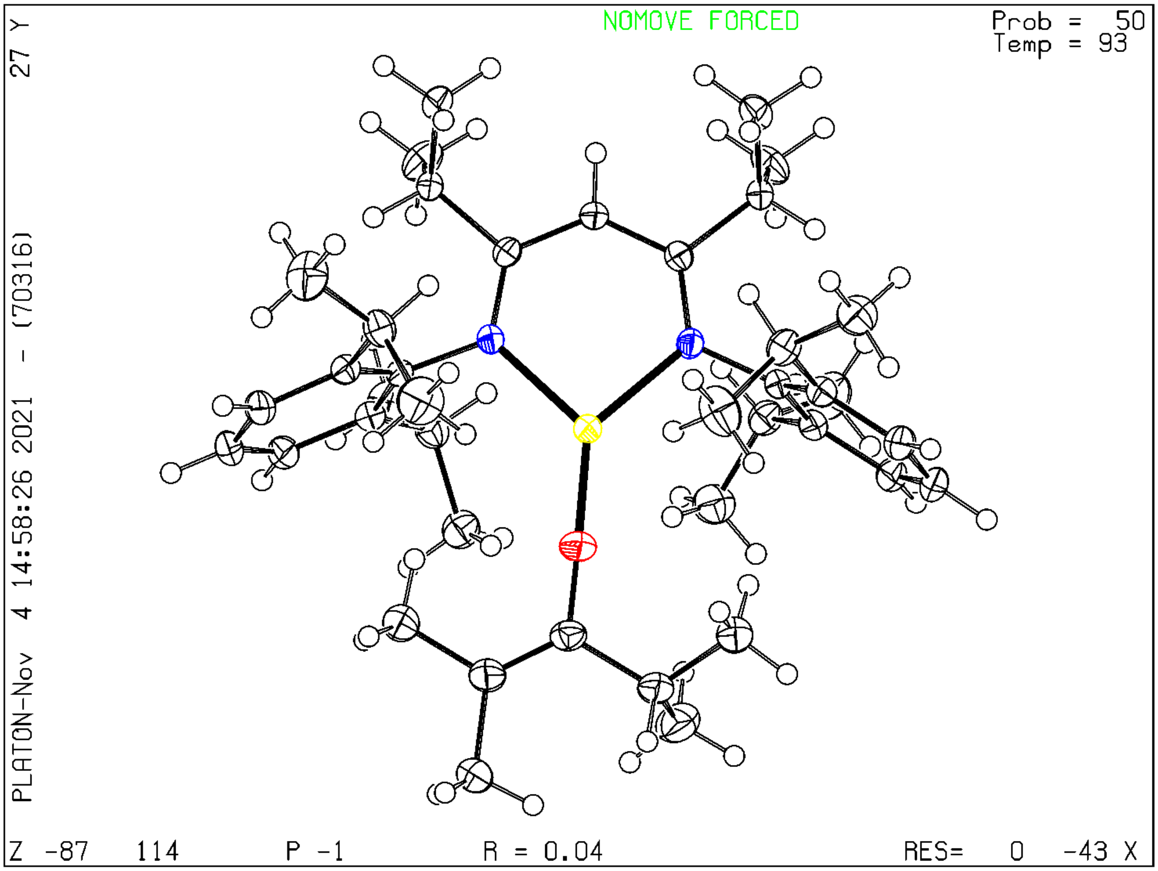

checkCIF/PLATON report

You have not supplied any structure factors. As a result the full set of tests cannot be run.

THIS REPORT IS FOR GUIDANCE ONLY. IF USED AS PART OF A REVIEW PROCEDURE FOR PUBLICATION, IT SHOULD NOT REPLACE THE EXPERTISE OF AN EXPERIENCED CRYSTALLOGRAPHIC REFEREE.

No syntax errors found. CIF dictionary Interpreting this report

Datablock: 120

```
Bond precision:   C-C = 0.0018 A          Wavelength=0.71073

Cell:          a=9.8100(2)       b=10.2482(2)       c=21.6956(6)
               alpha=81.568(2)   beta=88.7548(19)   gamma=85.5449(19)
Temperature:   93 K

                Calculated                   Reported
Volume          2150.95(9)                   2150.95(9)
Space group     P -1                         P -1
Hall group      -P 1                         -P 1
Moiety formula  C42 H68 Mg N2 O, 0.5(C6 H6) C45 H71 Mg N2 O
Sum formula     C45 H71 Mg N2 O              C45 H71 Mg N2 O
Mr              680.35                       680.37
Dx,g cm-3       1.051                        1.050
Z               2                            2
Mu (mm-1)       0.074                        0.074
F000            750.0                        750.0
F000'           750.31
h,k,lmax        13,13,29                     13,13,29
Nref            11005                        9124
Tmin,Tmax       0.997,0.998                  0.672,0.998
Tmin'           0.993

Correction method= # Reported T Limits: Tmin=0.672 Tmax=0.998
AbsCorr = MULTI-SCAN

Data completeness= 0.829           Theta(max)= 28.600

                                                    wR2(reflections)=
R(reflections)= 0.0425( 8127)                       0.1191( 9124)

S = 1.083                   Npar= 460
```

The following ALERTS were generated. Each ALERT has the format
test-name_ALERT_alert-type_alert-level.
Click on the hyperlinks for more details of the test.

Alert level A

```
PLAT331_ALERT_2_A Small Aver Phenyl C-C Dist C46      --C48_a    .       1.34 Ang.
```

Author Response: disordered solvent about a symmetry point.

Alert level C

```
PLAT220_ALERT_2_C NonSolvent   Resd 1   C   Ueq(max)/Ueq(min) Range        3.1 Ratio
```

Alert level G

```
PLAT002_ALERT_2_G Number of Distance or Angle Restraints on AtSite          3 Note
PLAT042_ALERT_1_G Calc. and Reported Moiety Formula Strings Differ     Please Check
PLAT172_ALERT_4_G The CIF-Embedded .res File Contains DFIX Records          1 Report
PLAT860_ALERT_3_G Number of Least-Squares Restraints ............          2 Note
PLAT933_ALERT_2_G Number of OMIT Records in Embedded .res File ...          6 Note
PLAT941_ALERT_3_G Average HKL Measurement Multiplicity ..........        2.7 Low
```

1 **ALERT level A** = Most likely a serious problem - resolve or explain
0 **ALERT level B** = A potentially serious problem, consider carefully
1 **ALERT level C** = Check. Ensure it is not caused by an omission or oversight
6 **ALERT level G** = General information/check it is not something unexpected

1 ALERT type 1 CIF construction/syntax error, inconsistent or missing data
4 ALERT type 2 Indicator that the structure model may be wrong or deficient
2 ALERT type 3 Indicator that the structure quality may be low
1 ALERT type 4 Improvement, methodology, query or suggestion
0 ALERT type 5 Informative message, check

It is advisable to attempt to resolve as many as possible of the alerts in all categories. Often the minor alerts point to easily fixed oversights, errors and omissions in your CIF or refinement strategy, so attention to these fine details can be worthwhile. In order to resolve some of the more serious problems it may be necessary to carry out additional measurements or structure refinements. However, the purpose of your study may justify the reported deviations and the more serious of these should normally be commented upon in the discussion or experimental section of a paper or in the "special_details" fields of the CIF. checkCIF was carefully designed to identify outliers and unusual parameters, but every test has its limitations and alerts that are not important in a particular case may appear. Conversely, the absence of alerts does not guarantee there are no aspects of the results needing attention. It is up to the individual to critically assess their own results and, if necessary, seek expert advice.

Publication of your CIF in IUCr journals

A basic structural check has been run on your CIF. These basic checks will be run on all CIFs submitted for publication in IUCr journals (*Acta Crystallographica*, *Journal of Applied Crystallography*, *Journal of Synchrotron Radiation*); however, if you intend to submit to *Acta Crystallographica Section C* or *E* or *IUCrData*, you should make sure that full publication checks are run on the final version of your CIF prior to submission.

Publication of your CIF in other journals

Please refer to the *Notes for Authors* of the relevant journal for any special instructions relating to CIF submission.

PLATON version of 13/07/2021; check.def file version of 13/07/2021

Datablock 120 - ellipsoid plot

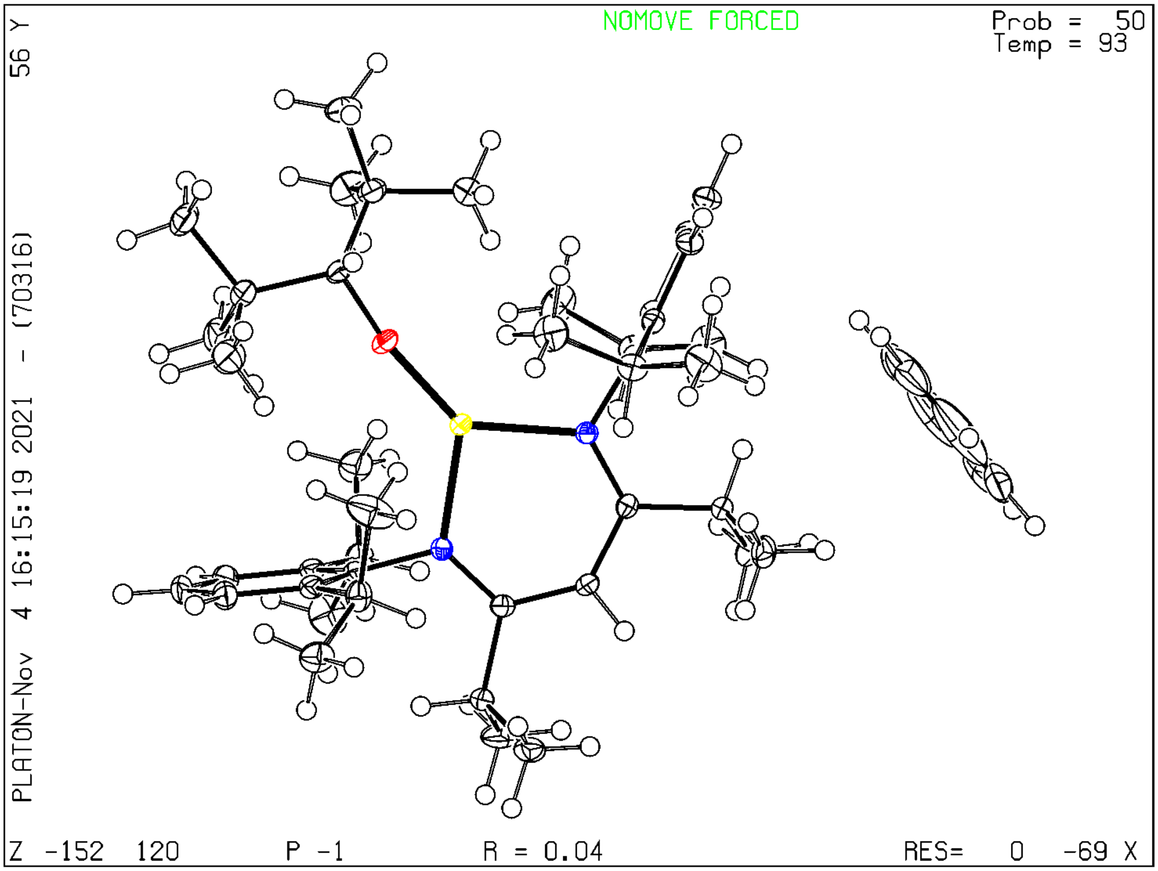

checkCIF/PLATON report

Structure factors have been supplied for datablock(s) 124x

THIS REPORT IS FOR GUIDANCE ONLY. IF USED AS PART OF A REVIEW PROCEDURE FOR PUBLICATION, IT SHOULD NOT REPLACE THE EXPERTISE OF AN EXPERIENCED CRYSTALLOGRAPHIC REFEREE.

No syntax errors found. CIF dictionary Interpreting this report

Datablock: 124x

Bond precision:	C-C = 0.0031 A		Wavelength=0.71075
Cell:	a=13.8663(16)	b=16.471(2)	c=22.411(3)
	alpha=90	beta=93.277(4)	gamma=90
Temperature:	173 K		

	Calculated	Reported
Volume	5110.1(11)	5110.1(11)
Space group	P 21/c	P 21/c
Hall group	-P 2ybc	-P 2ybc
Moiety formula	C55 H76 Mg2 N4 O	?
Sum formula	C55 H76 Mg2 N4 O	C55 H76 Mg2 N4 O
Mr	857.82	857.81
Dx,g cm-3	1.115	1.114
Z	4	4
Mu (mm-1)	0.088	0.088
F000	1864.0	1860.0
F000'	1864.87	
h,k,lmax	16,19,27	16,19,26
Nref	9385	9330
Tmin,Tmax	0.995,0.996	1.000,1.000
Tmin'	0.989	

Correction method= # Reported T Limits: Tmin=1.000 Tmax=1.000
AbsCorr = NONE

Data completeness= 0.994 Theta(max)= 25.387

R(reflections)= 0.0485(7713) wR2(reflections)= 0.1419(9330)

S = 1.088 Npar= 588

The following ALERTS were generated. Each ALERT has the format
test-name_ALERT_alert-type_alert-level.
Click on the hyperlinks for more details of the test.

Alert level C

```
PLAT068_ALERT_1_C Reported F000 Differs from Calcd (or Missing)...     Please Check
PLAT230_ALERT_2_C Hirshfeld Test Diff for    C19      --C20      .        5.5 s.u.
PLAT911_ALERT_3_C Missing FCF Refl Between Thmin & STh/L=    0.600         38 Report
```

Alert level G

```
PLAT066_ALERT_1_G Predicted and Reported Tmin&Tmax Range Identical          ? Check
PLAT303_ALERT_2_G Full Occupancy Atom H55A      with # Connections       2.00 Check
PLAT303_ALERT_2_G Full Occupancy Atom H55B      with # Connections       2.00 Check
PLAT303_ALERT_2_G Full Occupancy Atom H58C      with # Connections       2.00 Check
PLAT303_ALERT_2_G Full Occupancy Atom H62C      with # Connections       2.00 Check
PLAT343_ALERT_2_G Unusual sp?      Angle Range in Main Residue for        C55 Check
PLAT883_ALERT_1_G No Info/Value for _atom_sites_solution_primary .     Please Do !
PLAT910_ALERT_3_G Missing # of FCF Reflection(s) Below Theta(Min).          2 Note
PLAT912_ALERT_4_G Missing # of FCF Reflections Above STh/L=  0.600         13 Note
PLAT933_ALERT_2_G Number of HKL-OMIT Records in Embedded .res File          1 Note
PLAT965_ALERT_2_G The SHELXL WEIGHT Optimisation has not Converged     Please Check
PLAT978_ALERT_2_G Number C-C Bonds with Positive Residual Density.          7 Info
```

0 **ALERT level A** = Most likely a serious problem - resolve or explain
0 **ALERT level B** = A potentially serious problem, consider carefully
3 **ALERT level C** = Check. Ensure it is not caused by an omission or oversight
12 **ALERT level G** = General information/check it is not something unexpected

3 ALERT type 1 CIF construction/syntax error, inconsistent or missing data
9 ALERT type 2 Indicator that the structure model may be wrong or deficient
2 ALERT type 3 Indicator that the structure quality may be low
1 ALERT type 4 Improvement, methodology, query or suggestion
0 ALERT type 5 Informative message, check

It is advisable to attempt to resolve as many as possible of the alerts in all categories. Often the minor alerts point to easily fixed oversights, errors and omissions in your CIF or refinement strategy, so attention to these fine details can be worthwhile. In order to resolve some of the more serious problems it may be necessary to carry out additional measurements or structure refinements. However, the purpose of your study may justify the reported deviations and the more serious of these should normally be commented upon in the discussion or experimental section of a paper or in the "special_details" fields of the CIF. checkCIF was carefully designed to identify outliers and unusual parameters, but every test has its limitations and alerts that are not important in a particular case may appear. Conversely, the absence of alerts does not guarantee there are no aspects of the results needing attention. It is up to the individual to critically assess their own results and, if necessary, seek expert advice.

Publication of your CIF in IUCr journals

A basic structural check has been run on your CIF. These basic checks will be run on all CIFs submitted for publication in IUCr journals (*Acta Crystallographica*, *Journal of Applied Crystallography*, *Journal of Synchrotron Radiation*); however, if you intend to submit to *Acta Crystallographica Section C* or *E* or *IUCrData*, you should make sure that full publication checks are run on the final version of your CIF prior to submission.

Publication of your CIF in other journals

Please refer to the *Notes for Authors* of the relevant journal for any special instructions relating to CIF submission.

PLATON version of 20/01/2022; check.def file version of 19/01/2022

Datablock 124x - ellipsoid plot

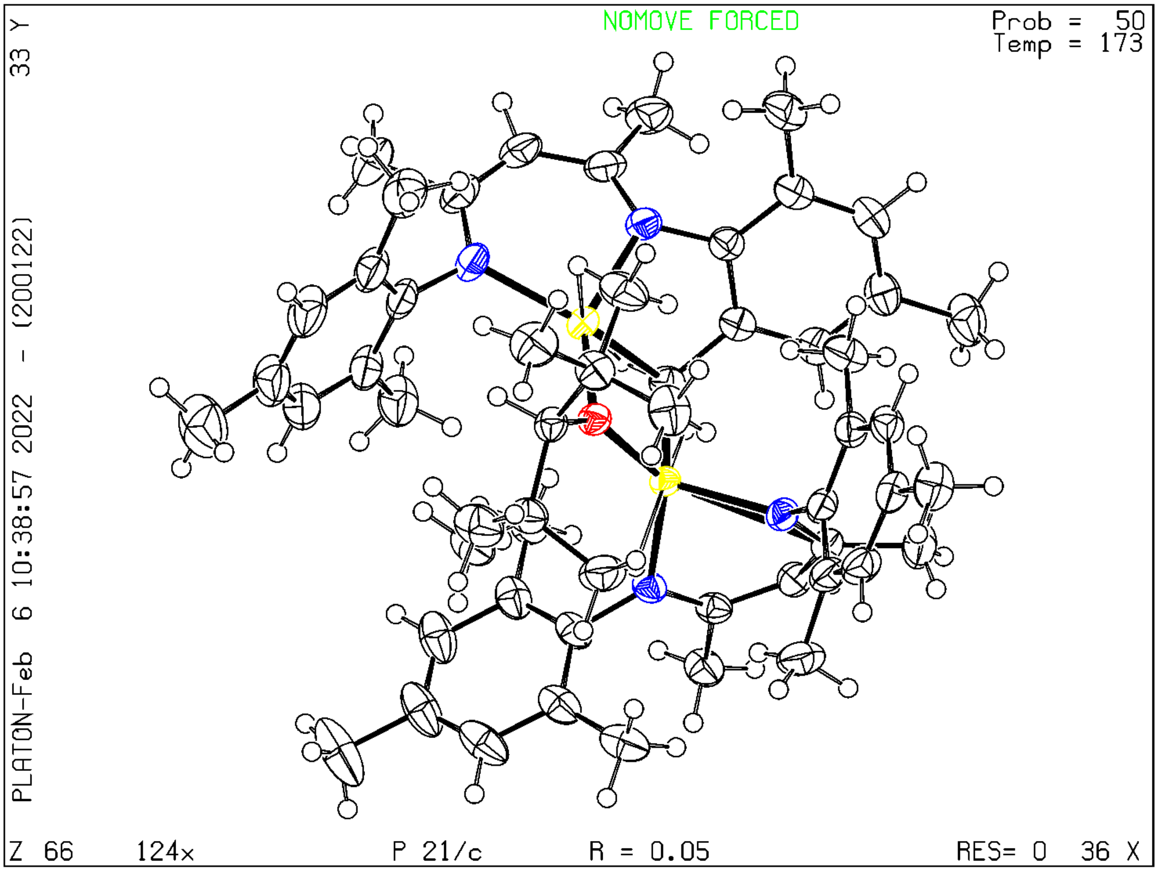

checkCIF/PLATON report

Structure factors have been supplied for datablock(s) 137x

THIS REPORT IS FOR GUIDANCE ONLY. IF USED AS PART OF A REVIEW PROCEDURE FOR PUBLICATION, IT SHOULD NOT REPLACE THE EXPERTISE OF AN EXPERIENCED CRYSTALLOGRAPHIC REFEREE.

No syntax errors found. CIF dictionary Interpreting this report

Datablock: 137x

```
Bond precision:   C-C = 0.0031 A          Wavelength=1.54184

Cell:             a=13.3421(3)     b=16.3669(3)       c=26.7682(5)
                  alpha=90         beta=96.610(2)     gamma=90
Temperature:      125 K

                 Calculated                    Reported
Volume           5806.5(2)                     5806.5(2)
Space group      P 21/c                        P 21/c
Hall group       -P 2ybc                       -P 2ybc
Moiety formula   C66 H86 Mg2 N4 O2             ?
Sum formula      C66 H86 Mg2 N4 O2             C66 H86 Mg2 N4 O2
Mr               1016.01                       1016.00
Dx,g cm-3        1.162                         1.162
Z                4                             4
Mu (mm-1)        0.724                         0.725
F000             2200.0                        2200.0
F000'            2206.62
h,k,lmax         16,20,33                      15,20,33
Nref             12226                         11680
Tmin,Tmax        0.917,0.930                   0.758,0.930
Tmin'            0.865

Correction method= # Reported T Limits: Tmin=0.758 Tmax=0.930
AbsCorr = MULTI-SCAN

Data completeness= 0.955           Theta(max)= 76.559

R(reflections)= 0.0600( 10302)                          wR2(reflections)=
                                                        0.1931( 11680)
S = 1.158                    Npar= 724
```

The following ALERTS were generated. Each ALERT has the format
test-name_ALERT_alert-type_alert-level.
Click on the hyperlinks for more details of the test.

Alert level C

```
PLAT220_ALERT_2_C NonSolvent   Resd 1  C   Ueq(max)/Ueq(min) Range        4.8 Ratio
PLAT222_ALERT_3_C NonSolvent Resd 1  H   Uiso(max)/Uiso(min) Range        4.4 Ratio
PLAT911_ALERT_3_C Missing FCF Refl Between Thmin & STh/L=    0.600         38 Report
PLAT918_ALERT_3_C Reflection(s) with I(obs) much Smaller I(calc) .          8 Check
PLAT939_ALERT_3_C Large Value of Not (SHELXL) Weight Optimized S .      10.70 Check
```

Alert level G

```
PLAT002_ALERT_2_G Number of Distance or Angle Restraints on AtSite         10 Note
PLAT172_ALERT_4_G The CIF-Embedded .res File Contains DFIX Records          4 Report
PLAT301_ALERT_3_G Main Residue  Disorder .............(Resd  1  )        14% Note
PLAT303_ALERT_2_G Full Occupancy Atom H28B      with # Connections       2.00 Check
PLAT413_ALERT_2_G Short Inter XH3 .. XHn     H63C     ..H72D     .       2.04 Ang.
                                         1-x,-1/2+y,1/2-z  =      2_645 Check
PLAT793_ALERT_4_G Model has Chirality at C26         (Centro SPGR)          S Verify
PLAT793_ALERT_4_G Model has Chirality at C31         (Centro SPGR)          R Verify
PLAT860_ALERT_3_G Number of Least-Squares Restraints ............          12 Note
PLAT883_ALERT_1_G No Info/Value for _atom_sites_solution_primary .     Please Do !
PLAT912_ALERT_4_G Missing # of FCF Reflections Above STh/L=  0.600        403 Note
PLAT933_ALERT_2_G Number of HKL-OMIT Records in Embedded .res File          1 Note
PLAT978_ALERT_2_G Number C-C Bonds with Positive Residual Density.          2 Info
```

0 **ALERT level A** = Most likely a serious problem - resolve or explain
0 **ALERT level B** = A potentially serious problem, consider carefully
5 **ALERT level C** = Check. Ensure it is not caused by an omission or oversight
12 **ALERT level G** = General information/check it is not something unexpected

1 ALERT type 1 CIF construction/syntax error, inconsistent or missing data
6 ALERT type 2 Indicator that the structure model may be wrong or deficient
6 ALERT type 3 Indicator that the structure quality may be low
4 ALERT type 4 Improvement, methodology, query or suggestion
0 ALERT type 5 Informative message, check

It is advisable to attempt to resolve as many as possible of the alerts in all categories. Often the minor alerts point to easily fixed oversights, errors and omissions in your CIF or refinement strategy, so attention to these fine details can be worthwhile. In order to resolve some of the more serious problems it may be necessary to carry out additional measurements or structure refinements. However, the purpose of your study may justify the reported deviations and the more serious of these should normally be commented upon in the discussion or experimental section of a paper or in the "special_details" fields of the CIF. checkCIF was carefully designed to identify outliers and unusual parameters, but every test has its limitations and alerts that are not important in a particular case may appear. Conversely, the absence of alerts does not guarantee there are no aspects of the results needing attention. It is up to the individual to critically assess their own results and, if necessary, seek expert advice.

Publication of your CIF in IUCr journals

A basic structural check has been run on your CIF. These basic checks will be run on all CIFs submitted for publication in IUCr journals (*Acta Crystallographica*, *Journal of Applied Crystallography*, *Journal of Synchrotron Radiation*); however, if you intend to submit to *Acta Crystallographica Section C* or *E* or *IUCrData*, you should make sure that full publication checks are run on the final version of your CIF prior to submission.

Publication of your CIF in other journals

Please refer to the *Notes for Authors* of the relevant journal for any special instructions relating to CIF submission.

PLATON version of 20/01/2022; check.def file version of 19/01/2022

Datablock 137x - ellipsoid plot

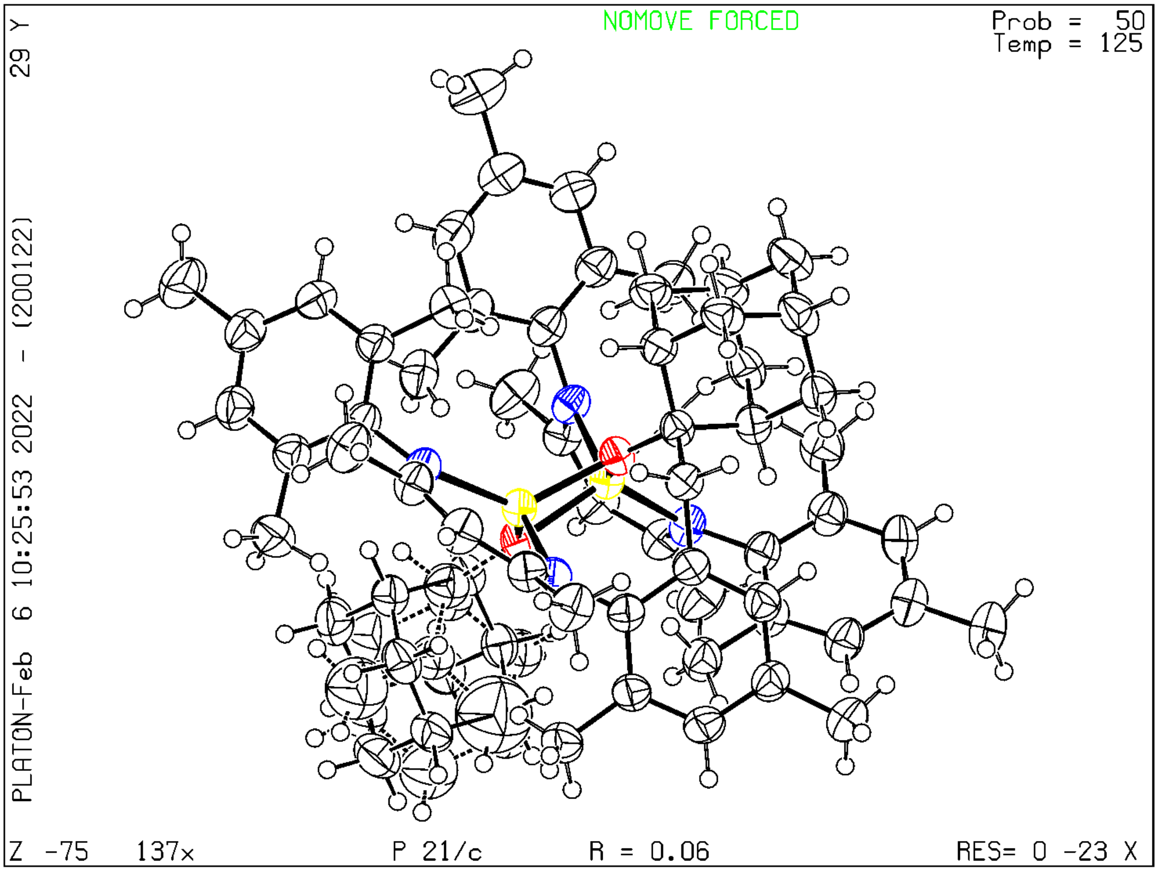

Supplement: Supplementary file 12 — Supporting Information [file ANIE-61-0-s010.pdf]
