# Supplementary material for: Umpolung of an Aliphatic Ketone to a Magnesium Ketone‐1,2‐diide Complex with Vicinal Dianionic Charge
Source: Angew Chem Int Ed Engl. 2022 Jul 11;61(34):e202204472. doi: 10.1002/anie.202204472 (PMC9541192; doi:10.1002/anie.202204472)
Supplement: Supplementary file 16 — Supporting Information [file ANIE-61-0-s011.pdf]

Angewandte Chemie
Eine Zeitschrift der Gesellschaft Deutscher Chemiker

Supporting Information

Umpolung of an Aliphatic Ketone to a Magnesium Ketone-1,2-diide Complex with Vicinal Dianionic Charge

*S. Burnett, C. Bourne, A. M. Z. Slawin, T. van Mourik, A. Stasch**

Electronic supplementary information

Table of Contents

1	Experimental Section	2
2	NMR spectroscopy	13
3	X-ray crystallography	74
4	Computational studies	94
5	References	98

1 Experimental Section

1.1 General considerations

All manipulations were carried out using standard Schlenk and glove box techniques under an atmosphere of high purity argon or dinitrogen. Benzene, toluene, *n*-hexane and *n*-pentane were either dried and distilled under inert gas over $LiAlH_4$, sodium or potassium, or taken from an MBraun solvent purification system and degassed prior to use. ^{1}H and $^{13}C\{^{1}H\}$ NMR spectra were recorded on a Bruker AVII 400 or Bruker AV III 500 spectrometer in deuterated benzene or toluene and were referenced to the residual ^{1}H or $^{13}C\{^{1}H\}$ resonances of the solvent used. Abbreviations: s = singlet, d = doublet, t = triplet, q = quartet, quint = quintet, sext = sextet, sept = septet, br = broad, vbr = very broad, m = multiplet, v = virtual signal, e.g., vsext, virtual sextet. Yields or conversions in solution were determined by integration of ^{1}H NMR spectra against an internal standard (such as residual C_6D_5H from deuterated benzene, added hexamethylbenzene etc). Melting points were determined in sealed glass capillaries under argon and are uncorrected. Elemental analyses were performed by the Elemental Analysis Service at London Metropolitan University. The elemental analyses appear to be affected by the highly air- and moisture sensitive nature of the compounds. Especially second runs showed lower values for C, H, and N, but mainly and routinely for C, in the repeat analyses likely due to oxidation, hydrolysis or interacting with other species during sample preparation, and thus it is likely that first runs were affected as well, but to a lower degree. The syntheses of $[\{(^{MeMes}nacnac)Mg\}_2]$ was performed according to a literature procedure.[1] All other compounds were used as received from chemical suppliers.

Selected NMR spectra are collected in **Section 2**. Unless stated otherwise, NMR spectra were recorded in deuterated benzene. Chemical shifts are given in ppm. Further information has been provided in the Figure captions.

1.2 Syntheses

^{iPrDip}nacnacH 1

A Schlenk flask with reflux condenser and nitrogen inlet was charged with P_4O_{10} (20.0 g, 70.45 mmol), hexamethyldisiloxane (48.0 mL, 36.7 g, 226 mmol) and the mixture was dissolved in dichloromethane (50 mL). The reaction mixture was heated to reflux for 1.5 h under a stream of nitrogen before cooling to 20 °C. All volatiles were removed *in vacuo*, affording a colourless, viscous syrup of PPSE.[2] 2,6-dimethyl-3,5-heptanedione (3.00 mL, 2.73 g, 17.48 mmol) and 2,6-diisopropylaniline (7.50 mL, 7.05 g, 39.8 mmol) were added to the flask under a flow of nitrogen. The reaction mixture was then heated to 170 °C and stirred for 24 h at this temperature. The reaction mixture was then cooled to 95 °C and an aqueous solution of NaOH (100 mL, 1.5 M solution) was slowly added via the top of the reflux condenser with vigorous stirring. The solid was extracted with dichloromethane (2 × 40 mL), the organic layer separated, and the aqueous phase washed with dichloromethane (1 × 30 mL) before all volatiles were removed in vacuo. Extraction into methanol (40 mL) results in almost immediate precipitation of **1** as a pale yellow powder which is subsequently isolated by filtration and drying under vacuum. Concentrating the supernatant solution to *ca*. 10 mL and storing at -40 ºC for one day affords additional colourless crystalline crops of **1**. Yield = 6.17 g (74%). Mp.: 268-270 °C.

^{1}H NMR (400.1 MHz, $CDCl_3$, 294 K) δ = 1.11 (d, J_{HH} = 6.6 Hz, 24H, Ar-*o*-CH(CH_3)$_2$), 1.27 (d, J_{HH} = 6.8 Hz, 12H, NC(CH(CH_3)$_2$)), 2.45 (sept, J_{HH} = 6.8 Hz, 2H, NC(C*H*(CH$_3$)$_2$)), 3.14 (sept, J_{HH} = 6.9 Hz, 4H, Ar-*o*-CH(CH$_3$)$_2$), 4.97 (s, 1H, NC(CH(CH$_3$)$_2$)C*H*), 7.14 (s, 6H, Ar-*H*), 11.84 (s, 1H, N*H*). ^{13}C{^{1}H} NMR (125.7 MHz, $CDCl_3$, 294 K): δ = 21.9 (NC(CH(*C*H$_3$)$_2$)), 22.9 (Ar-*o*-CH(*C*H$_3$)$_2$), 25.3 (Ar-*o*-CH(*C*H$_3$)$_2$), 27.8 (Ar-*o*-*C*H(CH$_3$)$_2$), 30.1 (NC(*C*H(CH$_3$)$_2$)), 122.9 (Ar-*C*), 124.7 (Ar-*C*), 140.0 (Ar-*C*), 142.7 (Ar-*C*), 170.1 (N*C*(CH(CH(CH$_3$)$_2$)). ^{1}H NMR (400.1 MHz, benzene-d_6, 294 K) δ = 1.27 (d, J_{HH} = 6.8 Hz, 12H, NC(CH(CH_3)$_2$)), 1.17 (d, J_{HH} = 6.6 Hz, 12H, Ar-*o*-CH(CH_3)$_2$), 1.23 (d, J_{HH} = 6.6 Hz, 12H, Ar-*o*-CH(CH_3)$_2$), 2.54 (sept, J_{HH} = 6.8 Hz, 2H, NC(C*H*(CH$_3$)$_2$)), 3.34 (sept, J_{HH} = 6.9 Hz, 4H, Ar-*o*-C*H*(CH$_3$)$_2$), 5.12 (s, 1H, NC(CH(CH$_3$)$_2$)C*H*), 7.15 (s, 6H, Ar-*H*), 12.39 (s, 1H, N*H*). ^{13}C{^{1}H} NMR (125.7 MHz, benzene-d_6, 294 K): δ = 22.1 (NC(CH(*C*H$_3$)$_2$)), 23.2 (Ar-*o*-CH(*C*H$_3$)$_2$), 25.7 (Ar-*o*-CH(*C*H$_3$)$_2$), 28.3 (Ar-*o*-*C*H(CH$_3$)$_2$), 30.4 (NC(*C*H(CH$_3$)$_2$)), 84.0 (NC(CH(CH$_3$)$_2$)*C*H), 123.7 (Ar-*C*), 125.7 (Ar-*C*), 140.4 (Ar-*C*), 143.0 (Ar-*C*), 171.5 (N*C*(CH(CH$_3$)$_2$)).

[(^{iPrDip}nacnac)Mg*n*Bu] 2

To a 0 °C toluene solution (10 mL) of Mg(*n*Bu)$_2$ (1.00 M, 6.51 mL, 1.03 equiv.) was added a toluene solution (60 mL) of ^{iPrDip}nacnacH **1** (3.00 g, 6.32 mmol). The solution was then warmed to ambient

temperature and allowed to stir overnight. The solution was then concentrated to *ca.* 20 mL and placed at -40 °C for two days which afforded a colourless crystalline crop of **2**. Concentrating the filtrate to *ca.* 10 mL and storing at -40 °C for one day afforded a second crop of **2**. Colourless crystals suitable for X-ray crystallographic analysis were grown from a concentrated toluene solution. Yield = 2.79 g (79%). Mp.: 284-286 °C. ^{1}H NMR (400.1 MHz, benzene-d_6, 294 K) δ = -0.29 (vt, J_{HH} = 7.9 Hz, 2H, Mg-CH_2CH$_2$CH$_2$CH$_3$), 0.82-0.86 (m, 3H, Mg-CH$_2$CH$_2$CH$_2$CH_3), 0.98 (vsext, J_{HH} = 7.4 Hz, 2H, Mg-CH$_2$CH$_2$CH_2CH$_3$), 1.04 (d, J_{HH} = 6.7 Hz, 12H, NCCH(CH_3)$_2$), 1.18 (d, J_{HH} = 6.9 Hz, 12H, Ar-*o*-CH(CH_3)$_2$), 1.28 (vquint, J_{HH} = 7.8 Hz, 2H, Mg-CH$_2$CH_2CH$_2$CH$_3$), 1.31 (d, J_{HH} = 6.8 Hz, 12H, Ar-*o*-CH(CH_3)$_2$), 2.61 (sept, J_{HH} = 6.8 Hz, 2H, NCCH(CH$_3$)$_2$), 3.21 (sept, J_{HH} = 6.8 Hz, 4H, Ar-*o*-CH(CH$_3$)$_2$), 5.02 (s, 1H, NC(CH(CH$_3$)$_2$)CH), 7.10 (s, 6H, Ar-H). ^{13}C{^{1}H} NMR (125.7 MHz, benzene-d_6, 294 K): δ = 6.8 (Mg-CH$_2$CH$_2$CH$_2$CH$_3$), 14.4 (Mg-CH$_2$CH$_2$CH$_2$$CH_3$), 22.9 (NC(CH($CH_3$)$_2$)), 23.3 (Ar-*o*-CH($CH_3$)$_2$), 25.3 (Ar-*o*-CH($CH_3$)$_2$), 28.0 (Ar-*o*-$C$H(CH$_3$)$_2$), 30.8 (Mg-CH$_2CH_2$$CH_2CH_3$), 31.1 (Mg-CH$_2$$CH_2CH_2CH_3$), 32.0 (NC($C$H(CH$_3$)$_2$)), 85.3 (NC(CH(CH$_3$)$_2$)$C$H), 124.1 (Ar-$C$), 125.9 (Ar-$C$), 142.5 (Ar-$C$), 142.9 (Ar-$C$), 178.9 (N$C$(CH(CH$_3$)$_2$)). Elemental analysis: calculated for $C_{37}H_{58}N_2Mg$: C 80.05; H 10.53; N 5.05%; found: C 78.01; H 10.23; N 4.76%.

[{(^{iPrDip}nacnac)Mg(μ-I)}$_2$] 3 and [(^{iPrDip}nacnac)Mg(THF)I]

A toluene solution (20 mL) of I_2 (0.41 g, 1.61 mmol, 1.05 equiv.) was added to a cooled toluene solution (20 mL) of [(^{iPrDip}nacnac)Mg*n*Bu] **2** (0.85 g, 1.53 mmol) and the resulting yellow solution was warmed to ambient temperature and stirred for three hours. The solvent was then removed *in vacuo* and the residue taken up in *n*-hexane (50 mL). Storing this solution at -40 °C for one day afforded **3** as a white powder. Concentrating the filtrate to *ca.* 10 mL and storing at -40 °C for one day afforded a second crop of **3**. Colourless crystals suitable for X-ray crystallographic analysis were grown from a concentrated toluene or benzene solution. Yield = 0.67 g (70%). Mp.: 200-202 °C. ^{1}H NMR (400.1 MHz, benzene-d_6, 294 K) δ = 0.95 (br d, 24H, NCCH(CH_3)$_2$), 1.20 (br d, 48H, Ar-*o*-CH(CH_3)$_2$), 2.56 (br sept, 4H, NCCH(CH$_3$)$_2$), 3.21 (br sept, 8H, Ar-*o*-CH(CH$_3$)$_2$), 4.95 (br s, 2H, NC(CH(CH$_3$)$_2$)CH), 7.10-7.12 (m, 8H, Ar-H). ^{13}C{^{1}H} NMR (125.7 MHz, benzene-d_6, 294 K): δ = 23.5 (NC(CH(CH$_3$)$_2$)), 25.0 (Ar-*o*-CH(CH$_3$)$_2$), 27.8 (Ar-*o*-CH(CH$_3$)$_2$), 32.1 (NC(CH(CH$_3$)$_2$)), 86.2 (NC(CH(CH$_3$)$_2$)CH), 124.5 (Ar-C), 125.6 (Ar-C), 179.9 (NC(CH(CH$_3$)$_2$)). Elemental analysis: calculated for $C_{66}H_{98}N_4Mg_2$:C 63.42; H 7.90; N 4.48%; found: C 64.61; H 8.62; N 3.98%.

Addition of THF-d_8 (0.1 mL) to benzene-d_6 solutions (0.5 mL) of **3** (15 mg, 0.024 mmol) in a J. Young NMR tube quantitatively afforded a colourless solution of [(^{iPrDip}nacnac)Mg(THF)I] as determined by ^{1}H NMR spectroscopy, which was later crystallographically characterised. ^{1}H NMR (400.1 MHz, benzene-d_6, 294 K) δ = 0.99 (d, J_{HH} = 6.6 Hz, NC(CH(CH_3)$_2$), 1.27 (d, J_{HH} = 6.6 Hz, 12H, Ar-*o*-CH(CH_3)$_2$), 1.38 (br, 12H, Ar-*o*-CH(CH_3)$_2$), 2.55 (sept, J_{HH} = 6.6 Hz, 2H, NCCH(CH$_3$)$_2$),

3.28 (br, 4H, Ar-*o*-C*H*(CH_3)$_2$), 4.90 (s, 1H, NC(CH(CH_3)$_2$)C*H*), 7.17 (s, 6H, Ar-*H*). $^{13}C\{^1H\}$ NMR (125.7 MHz, benzene-d_6, 294 K): δ = 23.3 (NC(CH(*C*H_3)$_2$)), 24.5 (Ar-*o*-CH(*C*H_3)$_2$), 27.0 (br, Ar-*o*-CH(*C*H_3)$_2$), 28.0 (Ar-*o*-*C*H(CH_3)$_2$), 31.9 (NC(*C*H(CH_3)$_2$)), 85.7 (NC(CH(CH_3)$_2$)*C*H), 124.2 (br, Ar-*C*), 125.7 (Ar-*C*), 143.2 (Ar-*C*), 179.5 (N*C*(CH(CH_3)$_2$)).

[{(^{iPrDip}nacnac)Mg}$_2$] 4

A toluene solution (20 mL) of [(^{iPrDip}nacnac)Mg(μ-I)}$_2$] **3** (2.00 g, 3.21 mmol) was stirred over a potassium mirror for 16 hours. The resulting yellow solution was then settled, filtered, concentrated to *ca.* 5 mL and stored at -40 °C for two days, affording **4** as a pale yellow solid. Concentrating the filtrate to *ca.* 3 mL and storing at -40 °C for two days afforded a second crop of **4**. Yield = 0.96 g (60%). Mp.: >300 °C (no visible decomp). ^1H NMR (400.1 MHz, benzene-d_6, 294 K) δ = 0.96 (d, J_{HH} = 6.4 Hz, 24H, NCCH(C*H*$_3$)$_2$), 0.99 (d, J_{HH} = 6.2 Hz, 24H, Ar-*o*-CH(C*H*$_3$)$_2$), 1.26 (d, J_{HH} = 6.2 Hz, 24H, Ar-*o*-CH(C*H*$_3$)$_2$), 2.48 (sept, J_{HH} = 6.2 Hz, 4H, NCC*H*(CH_3)$_2$), 3.15 (sept, J_{HH} = 6.4 Hz, 8H, Ar-*o*-C*H*(CH_3)$_2$), 4.89 (s, 2H, NC(CH(CH_3)$_2$)C*H*), 7.08 (s, 12H, Ar-*H*). $^{13}C\{^1H\}$ NMR (125.7 MHz, benzene-d_6, 294 K): δ = 23.1 (NC(CH(*C*H_3)$_2$)), 24.2 (Ar-*o*-CH(*C*H_3)$_2$), 26.6 (Ar-*o*-CH(*C*H_3)$_2$), 27.7 (Ar-*o*-*C*H(CH_3)$_2$), 31.8 (NC(*C*H(CH_3)$_2$)), 85.3 (NC(CH(CH_3)$_2$)*C*H), 123.9 (Ar-*C*), 125.0 (Ar-*C*), 143.0 (Ar-*C*), 144.3 (Ar-*C*), 177.5 (N*C*(CH(CH_3)$_2$)). Elemental analysis: calculated for $C_{66}H_{98}N_4Mg_2$: C 79.58; H 9.92; N 5.62%; found: C 78.43; H 9.61; N 5.31%.

[{(^{iPrDip}nacnac)Mg(μ-H)}$_2$] 5

[(^{iPrDip}nacnac)Mg*n*Bu] **2** (0.50 g, 0.90 mmol) was dissolved in hexane (40 mL) in a J. Young flask and $PhSiH_3$ (0.12 mL, 0.99 mmol, 1.1 equiv.) was added. The solution was then heated to reflux for two days, concentrated to *ca.* 10 mL and stored at -40 °C for a further two days to yield **5** as a colourless crystalline solid. Concentrating the filtrate to *ca.* 10 mL and storing at -40 °C for one day afforded a second crop of **5**. Colourless crystals suitable for X-ray crystallographic analysis were grown from a concentrated *n*-hexane solution. Yield = 0.17 g (37%). Mp.: Decomposition at 170 °C. ^1H NMR (400.1 MHz, benzene-d_6, 294 K) δ = 0.93-0.96 (m, 48H, Ar-*o*-CH(C*H*$_3$)$_2$ and NCCH(C*H*$_3$)$_2$), 1.22 (d, J_{HH} = 6.8 Hz, 24H, Ar-*o*-CH(C*H*$_3$)$_2$), 2.45 (sept, J_{HH} = 6.8 Hz, 4H, NCC*H*(CH_3)$_2$), 3.11 (sept, J_{HH} = 6.8 Hz, 8H, Ar-*o*-C*H*(CH_3)$_2$), 3.96 (s, 2H, Mg-*H*), 4.90 (s, 2H, NC(CH(CH_3)$_2$)C*H*), 7.04-7.10 (m, 12H, Ar-*H*). $^{13}C\{^1H\}$ NMR (125.7 MHz, benzene-d_6, 294 K): δ = 23.2 (NC(CH(*C*H_3)$_2$)), 24.1 (Ar-*o*-CH(*C*H_3)$_2$), 26.0 (Ar-*o*-CH(*C*H_3)$_2$), 27.8 (Ar-*o*-*C*H(CH_3)$_2$), 31.8 (NC(*C*H(CH_3)$_2$)), 85.2 (NC(CH(CH_3)$_2$)*C*H), 124.1 (Ar-*C*), 125.2 (Ar-*C*), 143.1 (Ar-*C*), 144.6 (Ar-*C*), 179.2 (N*C*(CH(CH_3)$_2$)). Elemental analysis: calculated for $C_{66}H_{100}N_4Mg_2$:C 79.42; H 10.10; N 5.61%; found: C 79.39; H 9.92; N 5.26%.

[{(^{iPrDip}nacnac)Mg}$_2$(μ-OAd)] 6

[{(^{iPrDip}nacnac)Mg}$_2$] (50 mg, 50 μmol) **4** was dissolved in *n*-pentane (15 mL) and cooled to -80 °C. An *n*-pentane (5 mL) solution of 2-adamantanone (7.5 mg, 50 μmol) was then added, yielding a red(-purple) solution. This was stirred for five minutes before being warmed to 20 °C and stirred for a further three hours, with a gradual colour change to pale yellow being observed. Concentrating the solution to *ca.* 5 mL and storing at -40 °C for one day afforded **6** as a yellow crystalline solid. Yellow crystals of **6**·C_6H_6 suitable for X-ray crystallographic analysis were grown from a concentrated benzene solution. Yellow crystals of **6** obtained from *n*-hexane showed full molecule disorder. N.B.: If the reaction is carried out at room temperature in benzene-d_6 solution then the formation of **6** is rapid and clean according to ^{1}H NMR spectroscopy, not accompanied by significant colour changes, and merely the shade of yellow changes. Yield (isolated from pentane) = 37.3 mg (65%). ^{1}H NMR (400.1 MHz, benzene-d_6, 294 K) δ = 0.34 (br d of q, 1H, Ad-*H*), 0.50 (d, J_{HH} = 6.8 Hz, 3H, Ar-*o*-CH(C*H*$_3$)$_2$), 0.60 (d, J_{HH} = 6.8 Hz, 3H, Ar-*o*-CH(C*H*$_3$)$_2$), 0.81 (d, J_{HH} = 6.7 Hz, 3H, NC(CH(C*H*$_3$)$_2$)), 0.88 (d, J_{HH} = 6.7 Hz, 6H, NC(CH(C*H*$_3$)$_2$)), 0.98 (d, J_{HH} = 6.7 Hz, 3H, NC(CH(C*H*$_3$)$_2$)), 1.12 (d, J_{HH} = 6.7 Hz, 3H, NC(CH(C*H*$_3$)$_2$)), 1.16-1.20 (m, 10H, Ar-*o*-CH(C*H*$_3$)$_2$ and NC(CH(C*H*$_3$)$_2$)), 1.22-1.26 (m, 14H, Ar-*o*-CH(C*H*$_3$)$_2$ and NC(CH(C*H*$_3$)$_2$)), 1.29 (br d, 9H, Ar-*o*-CH(C*H*$_3$)$_2$), 1.33 (d, J_{HH} = 6.9 Hz, 3H, Ar-*o*-CH(C*H*$_3$)$_2$), 1.36-1.39 (m, 9H, Ar-*o*-CH(C*H*$_3$)$_2$), 1.44-1.50 (m, 3H, Ad-*H* and Ad-*H*$_2$), 1.58-1.65 (m, 14H, Ar-*o*-CH(C*H*$_3$)$_2$ and Ad-*H*$_2$), 1.79 (br s, 1H, Ad-*H*), 2.03 (br d, 1H, Ad-*H*), 2.41-2.55 (m, 2H, NC(C*H*(CH$_3$)$_2$)), 2.57-2.63 (m, 2H, NC(C*H*(CH$_3$)$_2$)), 2.82-2.92 (m, 2H, Ar-*o*-C*H*(CH$_3$)$_2$), 3.16 (sept, J_{HH} = 6.8 Hz, 1H, Ar-*o*-C*H*(CH$_3$)$_2$), 3.27-3.39 (m, 3H, Ar-*o*-C*H*(CH$_3$)$_2$), 3.44-3.54 (m, 2H, Ar-*o*-C*H*(CH$_3$)$_2$), 4.93 (s, 1H, NC(CH(CH$_3$)$_2$)C*H*), 5.00 (s, 1H, NC(CH(CH$_3$)$_2$)C*H*), 7.01-7.05 (m, 4H, Ar-*H*), 7.08-7.15 (m, 6H, Ar-*H*), 7.17-7.22 (m, 2H, Ar-*H*). ^{13}C NMR (125.7 MHz, benzene-d_6, 294 K) δ 22.3 (Ar-*o*-CH(C*H*$_3$)$_2$ or NC(CH(*C*H$_3$)$_2$)), 22.4 (Ar-*o*-CH(C*H*$_3$)$_2$ or NC(CH(*C*H$_3$)$_2$)), 22.4 (Ar-*o*-CH(C*H*$_3$)$_2$ or NC(CH(*C*H$_3$)$_2$)), 22.8 (Ar-*o*-CH(C*H*$_3$)$_2$ or NC(CH(*C*H$_3$)$_2$)), 23.0 (Ar-*o*-CH(C*H*$_3$)$_2$ or NC(CH(*C*H$_3$)$_2$)), 23.2 (Ar-*o*-CH(C*H*$_3$)$_2$ or NC(CH(*C*H$_3$)$_2$)), 23.3 (Ar-*o*-CH(C*H*$_3$)$_2$ or NC(CH(*C*H$_3$)$_2$)), 23.3 (Ar-*o*-CH(C*H*$_3$)$_2$ or NC(CH(*C*H$_3$)$_2$)), 23.4 (Ar-*o*-CH(C*H*$_3$)$_2$ or NC(CH(*C*H$_3$)$_2$)), 23.5 (Ar-*o*-CH(C*H*$_3$)$_2$ or NC(CH(*C*H$_3$)$_2$)), 23.7 (Ar-*o*-CH(C*H*$_3$)$_2$ or NC(CH(*C*H$_3$)$_2$)), 24.0 (Ar-*o*-CH(C*H*$_3$)$_2$ or NC(CH(*C*H$_3$)$_2$)), 24.1 (Ar-*o*-CH(C*H*$_3$)$_2$ or NC(CH(*C*H$_3$)$_2$)), 24.2 (Ar-*o*-CH(C*H*$_3$)$_2$ or NC(CH(*C*H$_3$)$_2$)), 24.3 (Ar-*o*-CH(C*H*$_3$)$_2$ or NC(CH(*C*H$_3$)$_2$)), 24.7 (Ar-*o*-CH(C*H*$_3$)$_2$ or NC(CH(*C*H$_3$)$_2$)), 24.8 (Ar-*o*-CH(C*H*$_3$)$_2$ or NC(CH(*C*H$_3$)$_2$)), 25.3 (Ar-*o*-CH(C*H*$_3$)$_2$ or NC(CH(*C*H$_3$)$_2$)), 25.4 (Ar-*o*-CH(C*H*$_3$)$_2$ or NC(CH(*C*H$_3$)$_2$)), 25.8 (Ar-*o*-CH(C*H*$_3$)$_2$ or NC(CH(*C*H$_3$)$_2$)), 26.0 (Ar-*o*-CH(C*H*$_3$)$_2$ or NC(CH(*C*H$_3$)$_2$)), 26.9 (Ar-*o*-*C*H(CH$_3$)$_2$), 27.3 (Ar-*o*-*C*H(CH$_3$)$_2$), 27.4 (Ar-*o*-*C*H(CH$_3$)$_2$), 27.5 (Ar-*o*-*C*H(CH$_3$)$_2$), 27.8 (Ar-*o*-*C*H(CH$_3$)$_2$), 27.8 (Ar-*o*-*C*H(CH$_3$)$_2$), 28.0 (Ar-*o*-*C*H(CH$_3$)$_2$), 28.1 (Ar-*o*-*C*H(CH$_3$)$_2$), 28.4 (Ar-*o*-*C*H(CH$_3$)$_2$), 28.5 (Ar-*o*-

*C*H(CH_3)$_2$), 29.0 (Ar-*o*-*C*H(CH_3)$_2$), 30.6 (NC(*C*H(CH_3)$_2$)), 30.6 (NC(*C*H(CH_3)$_2$)), 31.2 (Ad-*C*H_2), 31.6 (NC(*C*H(CH_3)$_2$)), 31.8 (NC(*C*H(CH_3)$_2$)), 32.9 (Ad-*C*H_2), 36.8 (Ad-*C*H_2), 38.1 (Ad-*C*H_2), 38.9 (Ad-*C*H_2), 40.0 (Ad-*C*H), 41.2 (Ad-*C*H), 86.4 (NC(CH(CH_3)$_2$)*C*H), 87.4 (NC(CH(CH_3)$_2$)*C*H), 98.8 (MgO*C*(R)Mg), 122.5 (Ar-*C*), 123.0 (Ar-*C*), 123.5 (Ar-*C*), 123.8 (Ar-*C*), 123.8 (Ar-*C*), 124.1 (Ar-*C*), 124.2 (Ar-*C*), 124.4 (Ar-*C*), 124.8 (Ar-*C*), 125.0 (Ar-*C*), 125.6 (Ar-*C*), 125.9 (Ar-*C*), 129.0 (Ar-*C*), 141.7 (Ar-*C*), 142.0 (Ar-*C*), 142.1 (Ar-*C*), 142.8 (Ar-*C*), 143.4 (Ar-*C*), 143.4 (Ar-*C*), 143.4 (Ar-*C*), 143.8 (Ar-*C*), 144.2 (Ar-*C*), 144.4 (Ar-*C*), 145.1 (Ar-*C*), 145.3 (Ar-*C*), 177.5 (N*C*(CH(CH_3)$_2$)), 178.3 (N*C*(CH(CH_3)$_2$)), 179.6 (N*C*(CH(CH_3)$_2$)), 179.9 (N*C*(CH(CH_3)$_2$)).

Decomposition of a benzene-d_6 (0.5 mL) solution of **6** to two main products was observed by ^{1}H NMR spectroscopy over a period of 78 days at 20 °C, one of which is attributed to the formation of [(^{iPrDip}nacnac)Mg(OAdH)] **8**. The half-life of **6** at room temperature is approximately two weeks. Heating a benzene-d_6 (0.5 mL) solution of **6** to 60 °C for 25 hours results in the formation of the aforementioned **8**, along with [{(^{iPrDip}nacnac)Mg}$_2$O] **7** and one other as yet unidentified main product, likely a CH activation product.

Note: the reaction of [{(^{MeDip}nacnac)Mg}$_2$] with 2-adamantanone occurs preferentially in the 1:2 ratio to a different type of product compared with **6** that shows some stability issues and hasn't yet been unambiguously characterised.

[{(^{iPrDip}nacnac)Mg}$_2$O] 7 and [{(^{iPrDip}nacnac)Mg(THF)}$_2$O]

A toluene (40 mL) solution of [{(^{iPrDip}nacnac)Mg}$_2$] (200 mg, 0.200 mmol) **4** in a J. Young's flask was frozen (-196 °C), the headspace evacuated before refilling with pre-dried N_2O (*ca.* 1 bar), and the mixture warmed to room temperature. This process was repeated two more times, during which time the reaction mixture became colourless. The resulting mixture was allowed to stir at 20 °C for one hour and a white precipitate formed. Filtration and drying of the precipitate under vacuum afforded **7** as a white powder. Note: Some formation of [{(^{iPrDip}nacnac)Mg(μ-OH}$_2$] (^{1}H NMR resonance of MgOH: δ = -0.54 ppm) could not be avoided during the preparation and isolation of **7**. Subsequently isolated crops from solution, via concentration of the filtrate and storing at low temperature, contained increasing quantities of [{(^{iPrDip}nacnac)Mg(μ-OH}$_2$] with respect to **7**. Excessive handling can increase the quantity of [{(^{iPrDip}nacnac)Mg(μ-OH}$_2$] in [{(^{iPrDip}nacnac)Mg}$_2$O] **7**. Yield = 127.7 mg (39%). ^{1}H NMR (400.1 MHz, benzene-d_6, 294 K) δ = 0.96-1.00 (m, 48H, NCCH(CH_3)$_2$ and Ar-*o*-CH(CH_3)$_2$), 1.21 (d, J_{HH} = 6.9 Hz, Ar-*o*-CH(CH_3)$_2$), 2.46 (sept, 4H J_{HH} = 6.9 Hz, NCC*H*(CH_3)$_2$), 3.08 (sept, 8H J_{HH} = 6.9 Hz, Ar-*o*-C*H*(CH_3)$_2$), 4.91 (s, 1H, NC(CH(CH_3)$_2$)C*H*), 7.01-7.15 (m, 12H, Ar-*H*). ^{13}C{^{1}H} NMR (125.7 MHz, benzene-d_6, 294 K): δ = 22.3 (Ar-*o*-CH(*C*H_3)$_2$), 22.8 (NC(CH(*C*H_3)$_2$)), 25.7 (Ar-*o*-CH(*C*H_3)$_2$), 28.2 (Ar-*o*-*C*H(CH_3)$_2$), 31.6

(NC(CH(CH$_3$)$_2$)), 84.7 (NC(CH(CH$_3$)$_2$)CH), 123.8 (Ar-C), 125.2 (Ar-C), 125.7 (Ar-C), 128.6 (Ar-C), 129.3 (Ar-C), 142.5 (Ar-C), 144.5 (Ar-C), 178.7 (NC(CH(CH$_3$)$_2$)).

[{(^{iPrDip}nacnac)Mg}$_2$O] **7** converts quantitatively in solution to [{(^{iPrDip}nacnac)Mg(THF)}$_2$O]. NMR data for the THF adduct [{(^{iPrDip}nacnac)Mg(THF)}$_2$O]: ^{1}H NMR (400.1 MHz, benzene-d_6, 294 K) δ = 0.98-1.02 (m, 48H, NCCH(CH_3)$_2$ and Ar-o-CH(CH_3)$_2$), 1.27 (d, J_{HH} = 6.8 Hz, 12H, Ar-o-CH(CH_3)$_2$), 2.48 (sept, J_{HH} = 6.8 Hz, 4H, NCCH(CH$_3$)$_2$), 3.12 (sept, J_{HH} = 6.8 Hz, 8H, Ar-o-CH(CH$_3$)$_2$), 4.77 (s, 2H, NC(CH(CH$_3$)$_2$)CH), 7.09-7.13 (m, 12H, Ar-H). ^{13}C{^{1}H} NMR (125.7 MHz, benzene-d_6, 294 K): δ = 23.4 (NC(CH(CH$_3$)$_2$)), 24.4 (Ar-o-CH(CH$_3$)$_2$), 26.5 (Ar-o-CH(CH$_3$)$_2$), 27.8 (Ar-o-CH(CH$_3$)$_2$), 31.3 (NC(CH(CH$_3$)$_2$)), 84.6 (NC(CH(CH$_3$)$_2$)CH), 123.6 (Ar-C), 124.3 (Ar-C), 125.7 (Ar-C), 129.3 (Ar-C), 143.0 (Ar-C), 146.8 (Ar-C), 176.7 (NC(CH(CH$_3$)$_2$)).

[(^{iPrDip}nacnac)Mg(OAdH)] 8

Method 1: To an *n*-hexane (10 mL) solution of [(^{iPrDip}nacnac)Mg*n*Bu] **2** (100 mg, 0.180 mmol) at -40 ºC was added an *n*-hexane (10 mL) solution of 2-adamantanol (27.4 mg, 0.180 mmol). The solution was stirred for 30 min before being warmed to 20 °C and stirred for a further 16 hours. Concentrating to *ca.* 10 mL and storing at -40 °C for one day afforded **8** as colourless crystals. Concentrating the filtrate to *ca.* 2 mL and storing at -40 °C for two days afforded a second crop of **8**. Yield = 36.3 mg (31%).

Method 2: [{(^{iPrDip}nacnac)Mg(μ-H)}$_2$] **5** (10 mg, 10 μmol) was dissolved in benzene-d_6 (0.5 mL) in a J. Young's NMR tube and 2-adamantanone (3.0 mg, 20 μmol, 2 equivs.) was added at 20 °C. Analysis by ^{1}H NMR spectroscopy showed immediate consumption of starting materials and formation of **8**.

Method 3: In a J. Young's NMR tube a benzene-d_6 (0.5 mL) solution of [{(^{iPrDip}nacnac)Mg}$_2$(μ-OAd)] **6** (10 mg, 8.72 μmol) was freeze-pump-thawed under vacuum twice. Pre-dried H_2 (*ca.* 1 bar) was then introduced to the head space of the tube at 20 °C and the tube subsequently sealed. Immediate formation of an as yet unidentified intermediate was observed by ^{1}H NMR spectroscopy (*ca.* 50% conversion). After 16 hours at 20 °C almost full conversion to **8** was observed alongside various quantities of by-products. In some cases, **8** was the only dominant product at the end of the reaction. Data for **8**: ^{1}H NMR (400.1 MHz, benzene-d_6, 294 K) δ = 1.02 (d, J_{HH} = 6.6 Hz, 12H, NCCH(CH_3)$_2$), 1.19 (d, J_{HH} = 6.6 Hz, 12H, Ar-o-CH(CH_3)$_2$), 1.22-1.28 (m, 4H, Ad-H_2), 1.41 (d, J_{HH} = 6.6 Hz, 12H, Ar-o-CH(CH_3)$_2$), 1.64-1.70 (m, 6H, Ad-H and Ad-H_2), 1.75-1.79 (m, 2H, Ad-H), 1.89-1.91 (m, 2H, Ad-H), 2.62 (sept, J_{HH} = 6.8 Hz, 2H, NCCH(CH$_3$)$_2$), 3.21 (sept, J_{HH} = 7.0 Hz, 4H, Ar-o-CH(CH$_3$)$_2$), 3.74 (t, J_{HH} = 3.2 Hz, 1H, Mg-OCHR), 5.01 (s, 1H, NC(CH(CH$_3$)$_2$)CH), 7.11-7.12 (m, 6H, Ar-H). ^{13}C{^{1}H} NMR (125.7 MHz, benzene-d_6, 294 K): δ = 22.7 (NCCH(CH$_3$)$_2$), 23.1 (Ar-

o-CH(*C*H_3)$_2$), 25.4 (Ar-*o*-CH(*C*H_3)$_2$), 28.2 (Ar-*o*-*C*H(CH$_3$)$_2$), 28.3 (Ad-*C*H$_2$), 28.8 (Ad-*C*H$_2$), 31.7 (Ad-*C*H), 32.0 (NC*C*H(CH$_3$)$_2$), 37.4 (Ad-*C*H), 38.6 (Ad-*C*H$_2$), 38.9 (Ad-*C*H), 74.9 (Mg-O*C*HR), 85.0 (NC(CH(CH$_3$)$_2$)*C*H), 124.0 (Ar-*C*), 126.0 (Ar-*C*), 142.2 (Ar-*C*), 142.4 (Ar-*C*), 180.0 (N*C*CH(CH$_3$)$_2$).

Formation of [(^{iPrDip}nacnac)Mg(OCH*i*Pr$_2$)] 9 and [(^{iPrDip}nacnac)Mg{OC(=CMe$_2$)*i*Pr$_2$}] 10

[{(^{iPrDip}nacnac)Mg}$_2$] (10 mg, 10 μmol) **4** was dissolved in benzene-*d*$_6$ (0.5 mL) in a J. Youngs NMR tube and 2,4-dimethyl-3-pentanone (2.3 mg, 2.8 μL, 20 μmol, 2 equivs.) was added at 20 °C. Analysis by ^{1}H NMR spectroscopy showed immediate consumption of starting materials and formation of **9** and **10**.

[(^{iPrDip}nacnac)Mg(OCH*i*Pr$_2$)] 9

[{(^{iPrDip}nacnac)Mg(μ-H)}$_2$] (100 mg, 0.100 mmol) **5** and 2,4-dimethyl-3-pentanone (22.8 mg, 28.4 μL, 0.200 mmol, 2 equivs.) were dissolved in benzene (20 mL) and the resulting colourless solution was stirred at 20 °C for 16 hours. Concentrating the solution to *ca.* 5 mL and storing at 5 °C for one day afforded **9** as colourless crystals. Colourless crystals of **9** suitable for X-ray crystallographic analysis were grown from a concentrated benzene-*d*$_6$ solution. Yield = 47.7 mg (39%). ^{1}H NMR (400.1 MHz, benzene-*d*$_6$, 294 K) δ = 0.50 (d, J_{HH} = 6.7 Hz, 6H, MgOCH(CH(C*H*$_3$)$_2$)), 0.60 (d, J_{HH} = 6.6 Hz, 6H, MgOCH(CH(C*H*$_3$)$_2$)), 1.00 (d, J_{HH} = 6.8 Hz, 12H, NCCH(C*H*$_3$)$_2$), 1.21 (d, J_{HH} = 6.8 Hz, 12H, Ar-*o*-CH(C*H*$_3$)$_2$), 1.38 (d, J_{HH} = 6.8 Hz, 12H, Ar-*o*-CH(C*H*$_3$)$_2$), 1.45-1.55 (m, 2H, MgOCH(C*H*(CH$_3$)$_2$)), 2.63 (sept, J_{HH} = 6.7 Hz, 2H, NCC*H*(CH$_3$)$_2$), 3.01 (t, J_{HH} = 5.5 Hz, 1H, MgOC*H*(CH(CH$_3$)$_2$)), 3.20 (sept, J_{HH} = 6.9 Hz, 4H, Ar-*o*-C*H*(CH$_3$)$_2$), 5.01 (s, 1H, NC(CH(CH$_3$)$_2$)C*H*), 7.13 (s, 6H, Ar-*H*). ^{13}C{^{1}H} NMR (125.7 MHz, benzene-*d*$_6$, 294 K): δ = 17.7 (MgOCH(CH(*C*H$_3$)$_2$)), 20.5 (MgOCH(CH(*C*H$_3$)$_2$)), 22.7 (NCCH(*C*H$_3$)$_2$), 23.2 (Ar-*o*-CH(*C*H$_3$)$_2$), 25.4 (Ar-*o*-CH(*C*H$_3$)$_2$), 28.1 (Ar-*o*-*C*H(CH$_3$)$_2$), 31.9 (NC*C*H(CH$_3$)$_2$), 32.8 (MgOCH(*C*H(CH$_3$)$_2$)), 82.2 (MgO*C*H(CH(CH$_3$)$_2$)), 85.0 (NC(CH(CH$_3$)$_2$)*C*H), 124.2 (Ar-*C*), 126.0 (Ar-*C*), 142.3 (Ar-*C*), 142.9 (Ar-*C*), 180.0 (N*C*CH(CH$_3$)$_2$).

[Na{OC(=CMe$_2$)*i*Pr$_2$}]

Sodium bis(trimethylsilyl)amide (500 mg, 2.73 mmol) was slurried in *n*-hexane (50 mL) at 20 °C before dropwise addition of 2,4-dimethyl-3-pentanone (31 mg, 0.39 mL, 2.73 mmol). Immediate dissolution of the amide was followed by precipitation of a white solid. The reaction mixture was stirred for 16 hours and the product (Na{OC(=CMe$_2$)*i*Pr$_2$}) allowed to settle before being filtered and dried under vacuum. Yield = 294.3 mg (79%). Due to low product solubility collection of meaningful solution state NMR spectroscopic data was not possible and the solid was used as is for the preparation of **10**.

[(^{iPrDip}nacnac)Mg{OC(=CMe$_2$)*i*Pr$_2$}] 10

[{(^{iPrDip}nacnac)Mg(μ-I)}$_2$] **3** (100 mg, 0.16 mmol) and [Na{OC(=CMe$_2$)*i*Pr$_2$}] (21.8 mg, 0.16 mmol) were slurried in toluene (30 mL). The resulting colourless reaction mixture was stirred at 20 °C for 16 hours during which time precipitation of a white solid was observed. This was then allowed to settle before being filtered. Volatiles were removed from the filtrate *in vacuo* and the residue extracted with *n*-hexane (2 mL). Storing at -40 °C for three days afforded **10** as colourless crystals. Colourless crystals of **10** suitable for X-ray crystallographic analysis were grown from a concentrated *n*-hexane solution. Yield = 35.2 mg (36%). ^{1}H NMR (400.1 MHz, benzene-*d*$_6$, 294 K) δ = 0.69 (d, J_{HH} = 6.8 Hz, 6H, Mg(OC(CH(C*H*$_3$)$_2$)(C(CH$_3$)$_2$)), 0.94 (br s, 3H, Mg(OC(CH(CH$_3$)$_2$)(C(C*H*$_3$)$_2$)), 1.00 (d, J_{HH} = 6.8 Hz, 6H, NCCH(C*H*$_3$)$_2$), 1.19 (J_{HH} = 6.8 Hz, 12H, Ar-*o*-CH(C*H*$_3$)$_2$), 1.33 (d, J_{HH} = 6.8 Hz, 12H, Ar-*o*-CH(C*H*$_3$)$_2$), 1.76 (br s, 3H, Mg(OC(CH(CH$_3$)$_2$)(C(C*H*$_3$)$_2$)), 2.64 (sept, J_{HH} = 6.8 Hz, 2H, NCC*H*(CH$_3$)$_2$), 2.84 (sept, J_{HH} = 6.8 Hz, 1H, Mg(OC(C*H*(CH$_3$)$_2$)(C(CH$_3$)$_2$)), 3.19 (sept, J_{HH} = 6.8 Hz, 4H, Ar-*o*-C*H*(CH$_3$)$_2$), 5.05 (s, 1H, NC(CH(CH$_3$)$_2$)C*H*), 7.11-7.12 (m, 6H, Ar-*H*). ^{13}C{^{1}H} NMR (125.7 MHz, benzene-*d*$_6$, 294 K): δ = 17.4 (Mg(OC(CH(CH$_3$)$_2$)(C(*C*H$_3$)$_2$)), 19.3 (Mg(OC(CH(CH$_3$)$_2$)(C(*C*H$_3$)$_2$)), 20.6 (Mg(OC(CH(*C*H$_3$)$_2$)(C(CH$_3$)$_2$)), 22.7 (NCCH(*C*H$_3$)$_2$), 23.2 (Ar-*o*-CH(*C*H$_3$)$_2$), 25.3 (Ar-*o*-CH(*C*H$_3$)$_2$), 28.2 (Ar-*o*-*C*H(CH$_3$)$_2$), 29.3 (Mg(OC(*C*H(CH$_3$)$_2$)(C(CH$_3$)$_2$)), 31.9 (NC*C*H(CH$_3$)$_2$), 85.2 (NC(CH(CH$_3$)$_2$)*C*H), 93.4 (Mg(OC(CH(CH$_3$)$_2$)(*C*(CH$_3$)$_2$)), 124.4 (Ar-*C*), 126.1 (Ar-*C*), 142.3 (Ar-*C*), 142.5 (Ar-*C*), 153.9 (Mg(O*C*(CH(CH$_3$)$_2$)(C(CH$_3$)$_2$)), 180.3 (N*C*CH(CH$_3$)$_2$).

[{(^{iPrDip}nacnac)Mg(OCH*t*Bu$_2$)] 11

Method 1: [{(^{iPrDip}nacnac)Mg}$_2$] **4** (10 mg, 10 μmol) and 2,2,4,4-tetramethyl-3-pentanone (2.85 mg, 3.5 μL, 0.020 mmol, 2 equivs.) were dissolved in benzene-*d*$_6$ (0.5 mL) in a J. Youngs NMR tube at 20 °C before the mixture was heated to 80 °C for two weeks. Analysis by ^{1}H NMR spectroscopy showed formation of **11** as the main product alongside one other as yet unidentified product.

Method 2: [{(^{iPrDip}nacnac)Mg(μ-H)}$_2$] **5** (10 mg, 0.10 mmol) was dissolved in benzene-*d*$_6$ (0.5 mL) in a J. Youngs NMR tube and 2,2,4,4-tetramethyl-3-pentanone (2.85 mg, 3.5 μL, 0.020 mmol, 2 equivs.) was added at 20 °C. Analysis by ^{1}H NMR spectroscopy showed immediate consumption of starting materials and formation of **11**. Colourless crystals of **11** suitable for X-ray crystallographic analysis were grown from a concentrated benzene-*d*$_6$ solution. Yield = 5.4 mg (42%). ^{1}H NMR (400.1 MHz, benzene-*d*$_6$, 294 K) δ = 0.77 (s, 18H, MgOCH(C(C*H*$_3$)$_3$)$_2$), 1.01 (d, J_{HH} = 6.6 Hz, 12H, NCCH(C*H*$_3$)$_2$), 1.19 (d, J_{HH} = 6.8 Hz, 12H, Ar-*o*-CH(C*H*$_3$)$_2$), 1.39 (d, J_{HH} = 6.8 Hz, 12H, Ar-*o*-CH(C*H*$_3$)$_2$), 2.63 (sept, J_{HH} = 6.6 Hz, 2H, NCC*H*(CH$_3$)$_2$), 2.98 (s, 1H, MgOC*H*(C(CH$_3$)$_3$)$_2$), 3.18 (sept, J_{HH} = 6.8 Hz, 4H, Ar-*o*-C*H*(CH$_3$)$_2$), 5.02 (s, 1H, NC(CH(CH$_3$)$_2$)C*H*), 7.11 (s, 6H, Ar-*H*). ^{13}C{^{1}H} NMR (125.7 MHz, benzene-*d*$_6$, 294 K): δ = 22.8 (NCCH(*C*H$_3$)$_2$), 23.4 (Ar-*o*-CH(*C*H$_3$)$_2$), 25.2 (Ar-

o-$CH(CH_3)_2$), 28.2 (Ar-*o*-$CH(CH_3)_2$), 29.9 ($MgOCH(C(CH_3)_3)_2$), 32.0 ($NCCH(CH_3)_2$), 38.2 ($MgOCH(C(CH_3)_3)_2$), 85.1 ($NC(CH(CH_3)_2)CH$), 87.1 ($MgOCH(C(CH_3)_3)_2$), 124.4 (Ar-*C*), 126.0 (Ar-*C*), 142.1 (Ar-*C*), 143.1 (Ar-*C*), 180.0 ($NCCH(CH_3)_2$).

[{(^{MeMes}nacnac)Mg}(*μ*-OCH*t*Bu_2)(*μ*-CH_2-$^{MeMes\text{-}H}$nacnac)Mg] 12

To a toluene solution (10 mL) of [{(^{Mes}Nacnac)Mg}$_2$] (200 mg, 0.280 mmol) at 20 °C was added 2,2,4,4-tetramethyl-3-pentanone (39.8 mg, 48.3 μL, 0.280 mmol) and the solution was stirred for 48 hours. Volatiles were removed *in vacuo* before the residue was slurried in *n*-hexane. The white precipitate was allowed to settle before being filtered off and dried under vacuum affording **12** as a white solid. Storing the filtrate at -40 °C for four days afforded a second crop of **12**. Colourless crystals of **12** suitable for X-ray crystallographic analysis were grown from a concentrated benzene-d_6 solution. Yield = 153.2 mg (64%). ^{1}H NMR (400.1 MHz, benzene-d_6, 294 K) δ = 0.39 (s, 9H, $MgOCH(C(CH_3)_3)_2$), 0.58 (s, 9H, $MgOCH(C(CH_3)_3)_2$), 1.53 (s, 3H, Ar-*o*-CH_3), 1.58 (d, J_{HH} = 14 Hz, 1H, Ar-CH_2Mg) 1.67 (s, 3H, $NCCH_3$), 1.69 (s, 3H, $NC(CH_3)$), 1.75 (s, 3H, $NC(CH_3)$), 1.85 (s, 3H $NC(CH_3)$), 2.03 (s, 3H, Ar-*o*-CH_3), 2.06 (s, 1H, $MgOCH(C(CH_3)_3)_2$), 2.15 (s, 9H, Ar-*o*-CH_3), 2.16 (d, J_{HH} = 14 Hz, 1H, Ar-CH_2Mg), 2.19 (s, 3H, Ar-*o*-CH_3), 2.20 (s, 3H, Ar-*o*-CH_3), 2.22-2.24 (m, 9H, Ar-*o*-CH_3), 2.33 (s, 3H, Ar-*o*-CH_3), 4.93 (s, 1H, $NC(CH(CH_3)_2)CH$), 5.20 (s, 1H, $NC(CH(CH_3)_2)CH$), 6.62 (br s, 1H, Ar-*H*), 6.66 (br s, 1H, Ar-*H*), 6.69 (br s, 2H, Ar-*H*), 6.74 (br s, 1H, Ar-*H*), 6.81 (br s, 1H, Ar-*H*), 6.83 (br s, 2H, Ar-*H*), 7.29 (br s, 1H, Ar-*H*). ^{13}C{^{1}H} NMR (125.7 MHz, benzene-d_6, 294 K): δ = 17.1 (Ar-CH_2Mg), 18.4 (Ar-CH_3), 18.8 (Ar-CH_3), 19.0 (Ar-CH_3), 19.4 (Ar-CH_3), 20.6 (Ar-CH_3), 20.6 (Ar-CH_3), 20.8 (Ar-CH_3), 20.9 (Ar-CH_3), 21.2 (Ar-CH_3), 21.6 (Ar-CH_3), 22.6 ($NC(CH_3)$), 24.0 ($NC(CH_3)$), 24.1 ($NC(CH_3)$), 24.7 ($NC(CH_3)$), 29.0 ($MgOCH(C(CH_3)_3)_2$), 29.1 ($MgOCH(C(CH_3)_3)_2$), 36.3 ($MgOCH(C(CH_3)_3)_2$), 36.9 ($MgOCH(C(CH_3)_3)_2$), 85.7 ($MgOCH(C(CH_3)_3)_2$), 95.7 ($NC(CH(CH_3)_2)CH$), 96.0 ($NC(CH(CH_3)_2)CH$), 124.1 (Ar-*C*), 126.2 (Ar-*C*), 129.0 (Ar-*C*), 129.1 (Ar-*C*), 129.5 (Ar-*C*), 129.6 (Ar-*C*), 129.7 (Ar-*C*), 129.9 (Ar-*C*), 131.6 (Ar-*C*), 132.0 (Ar-*C*), 132.1 (Ar-*C*), 132.1 (Ar-*C*), 132.7 (Ar-*C*), 132.9 (Ar-*C*), 133.1 (Ar-*C*), 133.5 (Ar-*C*), 142.4 (Ar-*C*), 142.5 (Ar-*C*), 146.4 (Ar-*C*), 146.8 (Ar-*C*), 147.1 (Ar-*C*), 167.9 ($NCCH_3$), 168.1 ($NCCH_3$), 168.9 ($NCCH_3$), 170.2 ($NCCH_3$).

[{(^{MeMes}nacnac)Mg}(*μ*-OAd)(*μ*-O(Ad)CH_2-$^{MeMes\text{-}H}$nacnac)Mg] 13

[{(^{Mes}Nacnac)Mg}$_2$] (60.0 mg, 0.084 mmol) and 2-adamantanone (25.0 mg, 0.168 mmol, 2 equivs.) were dissolved in *n*-hexane (10 mL) and stirred at 20 °C. Precipitation of a white solid was observed after approximately ten minutes along with the gradual fading of the yellow solution to colourless, which was complete after 16 hours. The white precipitate was allowed to settle before being filtered off and dried under vacuum affording **13** as a white solid. Concentrating the solution to *ca.* 2 mL and

storing at -40 °C for two days afforded a second crop of **13**. Colourless crystals of **13** suitable for X-ray crystallographic analysis were grown from a concentrated *n*-hexane solution. Yield = 58 mg (68%). ^{1}H NMR (400.1 MHz, benzene-d_6, 294 K) δ = 0.84 (br s, 1H, Ad-C*H*), 0.94 (br d, 1H, Ad-C*H*), 1.27 (br d, 1H, Ad-C*H*), 1.36-1.40 (m, 2H, Ad-C*H*), 1.44-1.50 (m, 3H, Ad-C*H*), 1.52-1.54 (m, 17H, Ar-CH_3 and NCCH_3), 1.57 (s, 3H, NCCH_3), 1.59-1.63 (m, 4H, Ad-C*H*), 1.67-1.68 (m, 4H, Ad-C*H*), 1.75-2.01 (m, 16H, Ad-C*H*), 2.11 (s, 3H, Ar-CH_3), 2.17 (s, 3H, Ar-CH_3), 2.19 (s, 3H, Ar-CH_3), 2.25 (s, 3H, Ar-CH_3), 2.28 (s, 3H, Ar-CH_3), 2.34-2.36 (m, 10H, Ar-CH_3), 2.46-2.49 (m, 4H, Ar-CH_2 and Mg-OC*H*R), 3.23 (br d, 1H, Ad-C*H*), 3.88 (br t, 1H, Ad-C*H*), 4.83 (s, 1H, NC(CH(CH_3)$_2$)C*H*), 4.88 (s, 1H, NC(CH(CH_3)$_2$)C*H*), 6.63 (s, 1H, Ar-*H*), 6.78 (s, 1H, Ar-*H*), 6.82 (s, 1H, Ar-*H*), 6.88 (s, 1H, Ar-*H*), 6.92 (s, 1H, Ar-*H*), 6.95 (s, 1H, Ar-*H*), 7.06 (s, 1H, Ar-*H*). ^{13}C{^{1}H} NMR (125.7 MHz, benzene-d_6, 294 K): δ = 17.5 (Ar-*C*H$_3$), 18.5 (Ar-*C*H$_3$), 18.6 (Ar-*C*H$_3$), 18.8 (Ar-*C*H$_3$), 19.1 (Ar-*C*H$_3$), 19.6 (Ar-*C*H$_3$), 20.0 (Ar-*C*H$_3$), 21.0 (Ar-*C*H$_3$), 21.0 (Ar-*C*H$_3$), 21.1 (Ar-*C*H$_3$), 22.2 (Ar-*C*H$_3$), 22.9 (NC*C*H$_3$), 23.5 (NC*C*H$_3$), 23.9 (NC*C*H$_3$), 25.0 (NC*C*H$_3$), 27.7 (Ad-*C*), 27.8 (Ad-*C*), 28.0 (Ad-*C*), 28.2 (Ad-*C*), 28.6 (Ad-*C*), 31.4 (Ad-*C*), 32.5 (Ad-*C*), 32.9 (Ad-*C*), 35.8 (Ad-*C*), 36.1 (Ad-*C*), 36.6 (Ad-*C*), 36.7 (Ad-*C*), 37.1 (Ad-*C*), 37.8 (Ad-*C*), 37.8 (Ad-*C*), 38.5 (Ad-*C*), 39.0 (Ad-*C*), 39.7 (Ad-*C*), 40.6 (Ad-*C*H), 43.2 (), 74.8 (Mg-O*C*HR), 76.5 (Mg-O*C*R), 95.2 (NC(CH(CH_3)$_2$)*C*H), 95.7 (NC(CH(CH_3)$_2$)*C*H), 128.9 (Ar-*C*), 129.1 (Ar-*C*), 129.2 (Ar-*C*), 129.6 (Ar-*C*), 130.1 (Ar-*C*), 130.4 (Ar-*C*), 130.7 (Ar-*C*), 130.8 (Ar-*C*), 131.6 (Ar-*C*), 131.7 (Ar-*C*), 131.9 (Ar-*C*), 132.5 (Ar-*C*), 132.6 (Ar-*C*), 132.8 (Ar-*C*), 132.9 (Ar-*C*), 133.1 (Ar-*C*), 133.3 (Ar-*C*), 133.4 (Ar-*C*), 146.6 (Ar-*C*), 147.0 (Ar-*C*), 147.9 (Ar-*C*), 148.0 (Ar-*C*), 168.2 (N*C*(CH_3)CH), 168.7 (N*C*(CH_3)CH), 168.9 (N*C*(CH_3)CH), 169.8 (N*C*(CH_3)CH).

2 NMR spectroscopy

Resonances of residual solvent (deuterated benzene: blue; deuterated chloroform: green) and silicone grease (orange) are marked with coloured dots, unless otherwise stated in the figure captions.

2.1 NMR spectra of ^{iPrDip}nacnacH 1

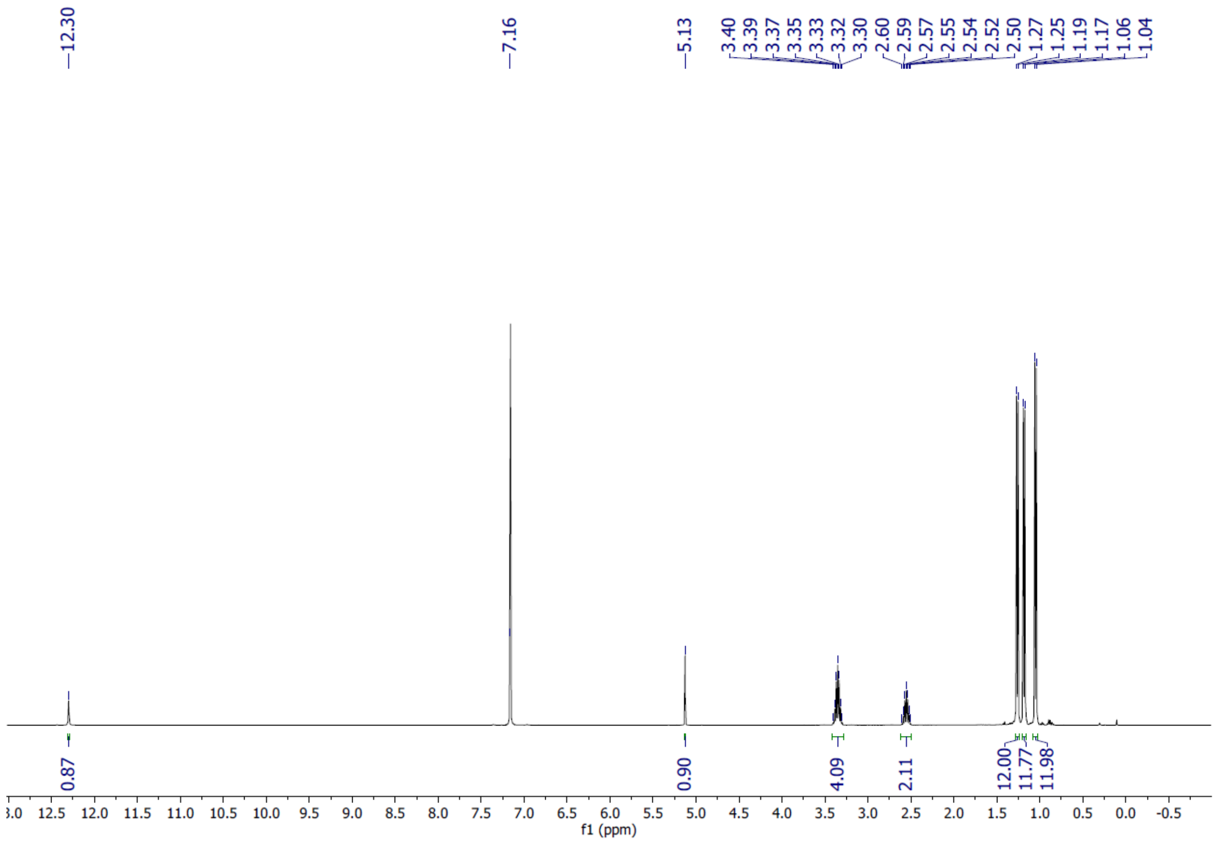

Figure S1. ^{1}H NMR spectrum (400.1 MHz, C_6D_6, 298 K) of ^{iPrDip}nacnacH **1.**

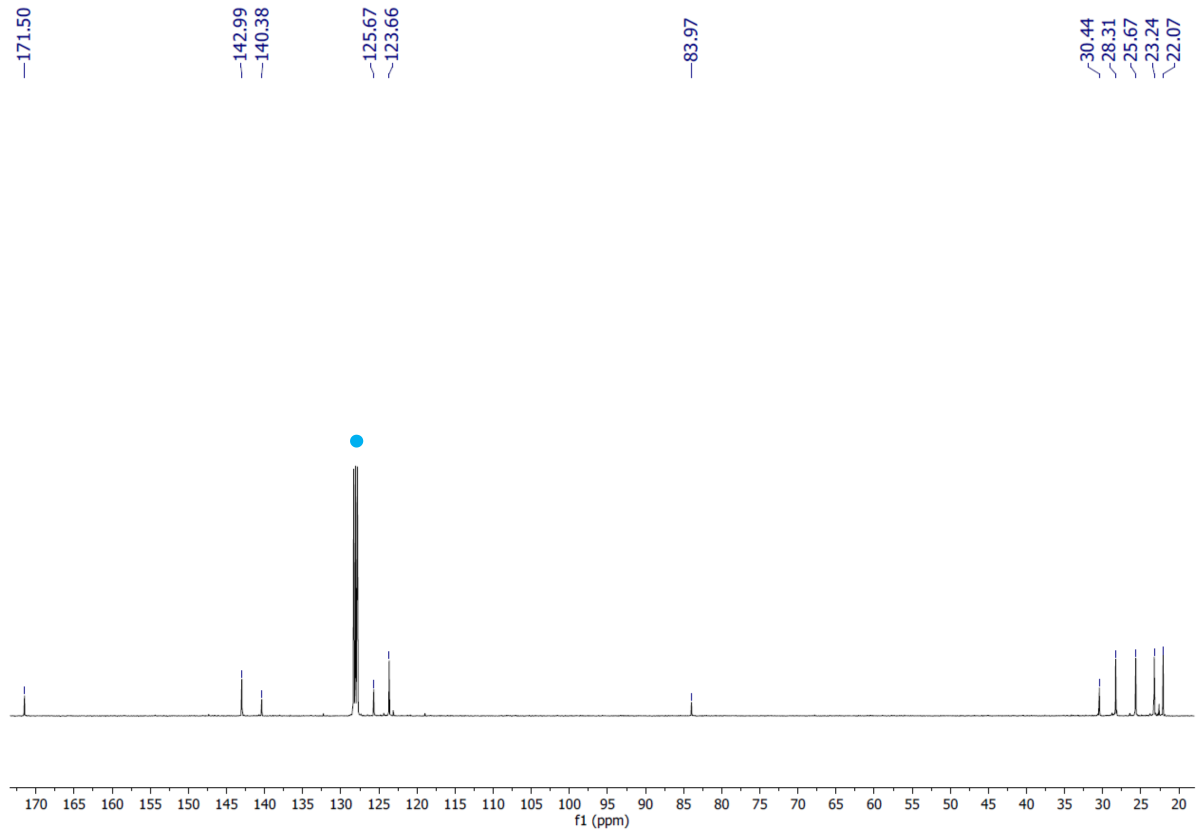

Figure S2. $^{13}C\{^{1}H\}$ NMR spectrum (100.5 MHz, C_6D_6, 298 K) of ^{iPrDip}nacnacH **1.**

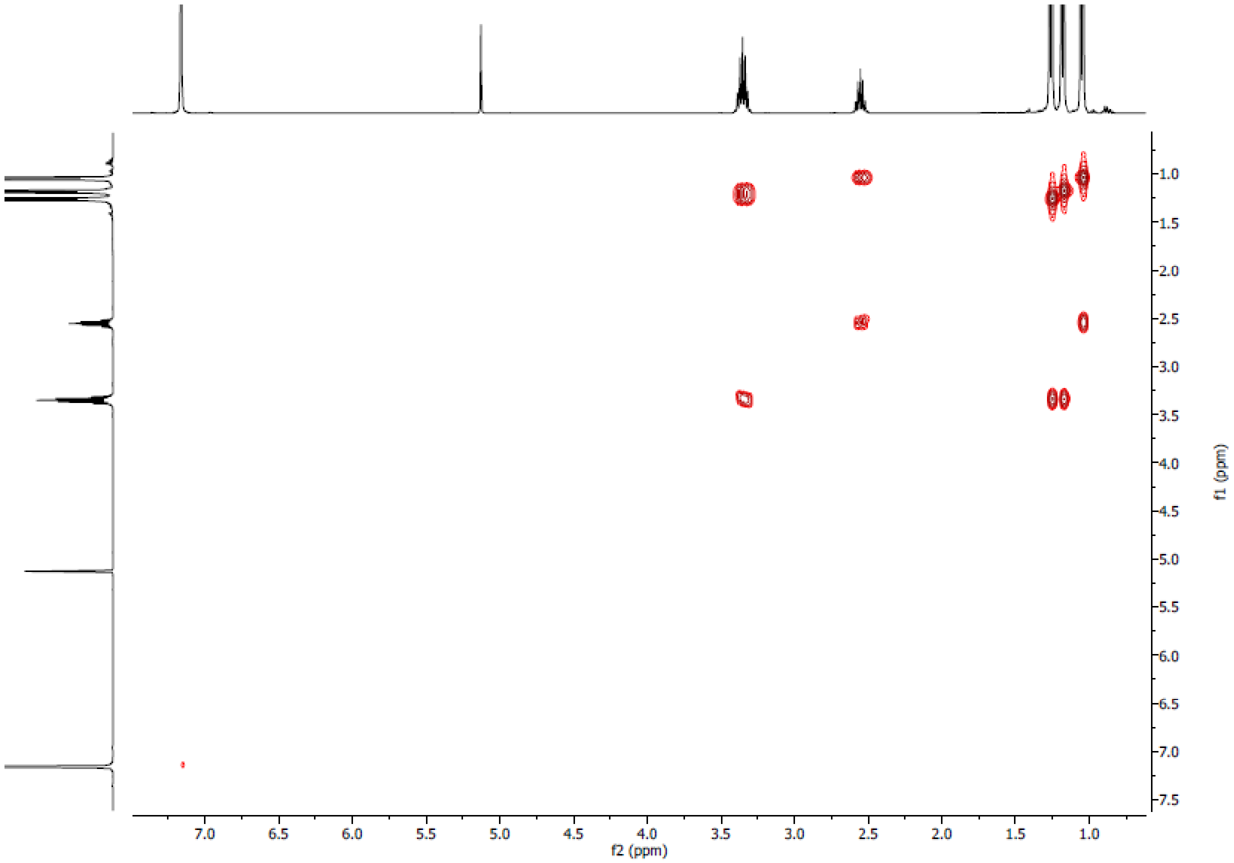

Figure S3. ^{1}H-^{1}H COSY NMR spectrum of ^{iPrDip}nacnacH **1.**

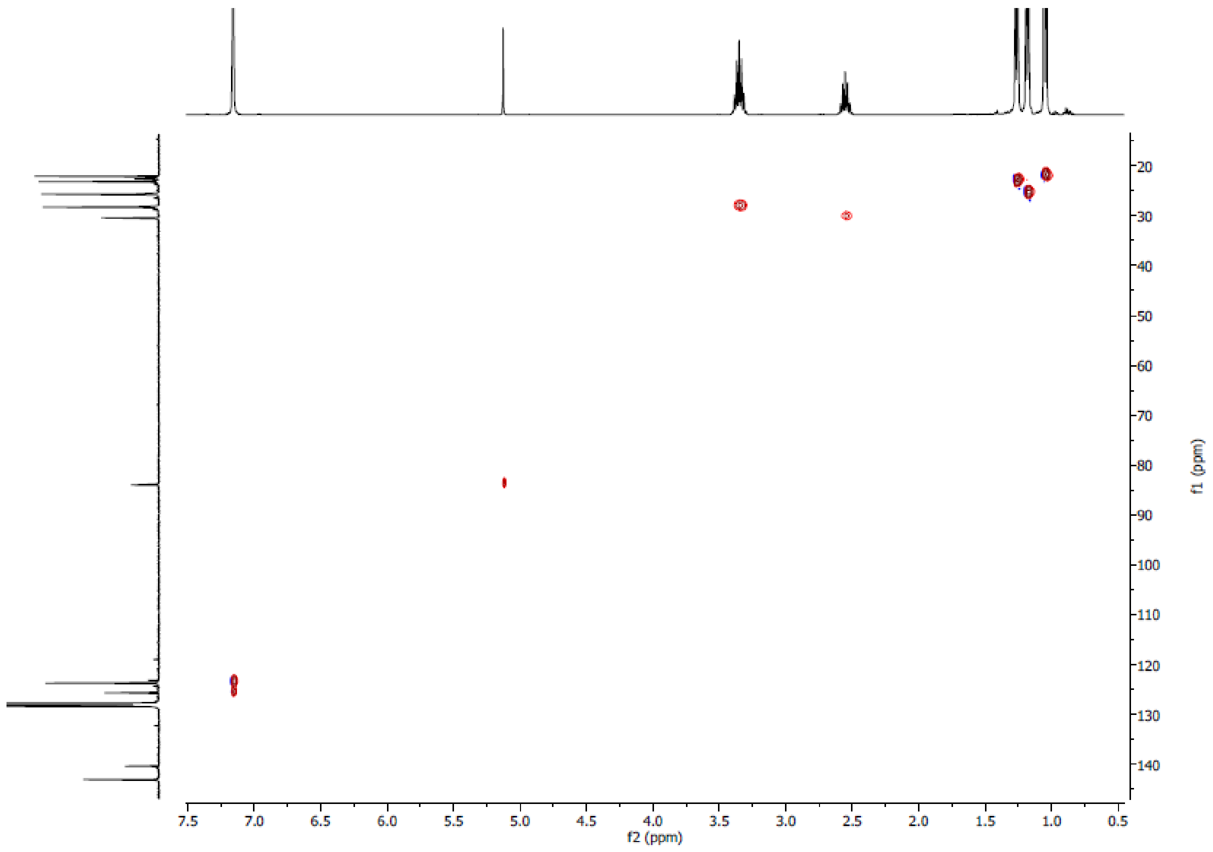

Figure S4. ^{1}H-^{13}C HSQC NMR spectrum of ^{iPrDip}nacnacH **1.**

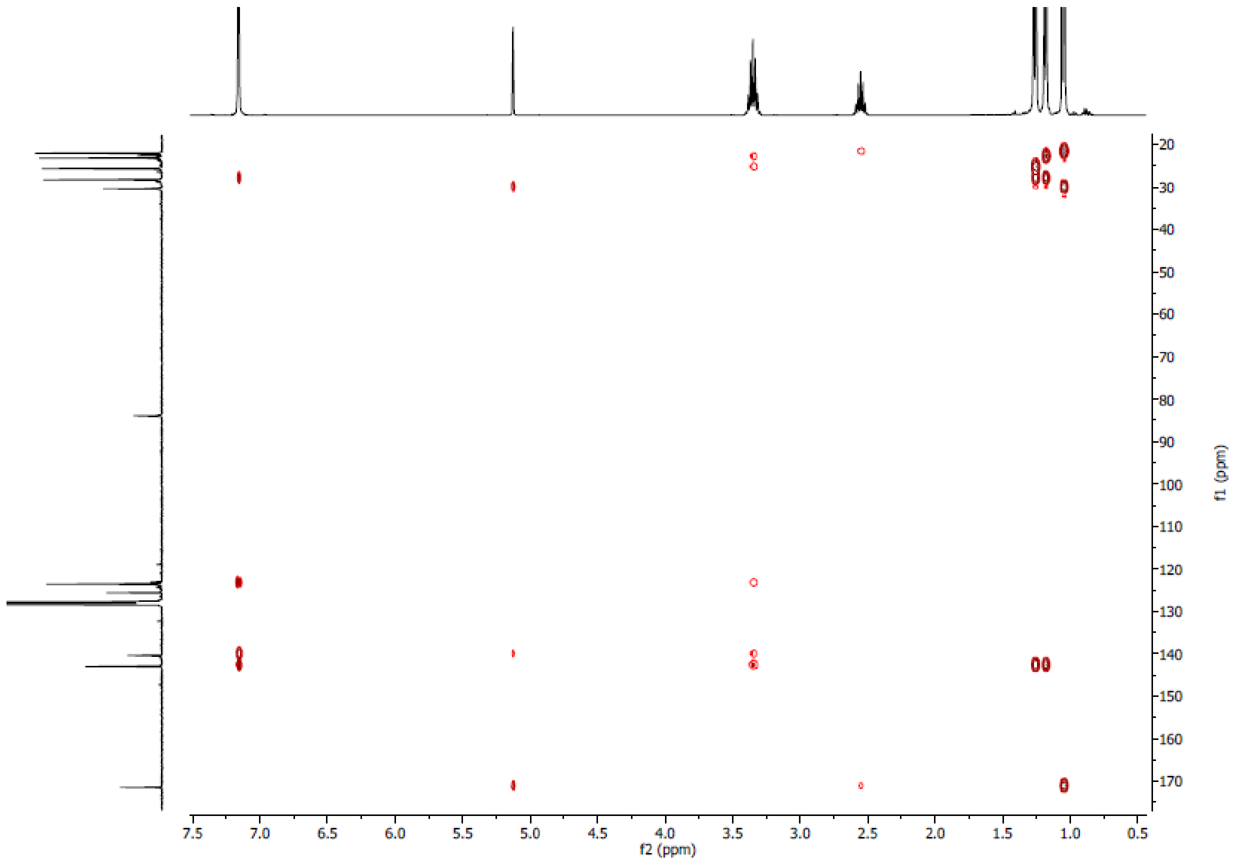

Figure S5. ^{1}H-^{13}C HMBC NMR spectrum of ^{iPrDip}nacnacH **1.**

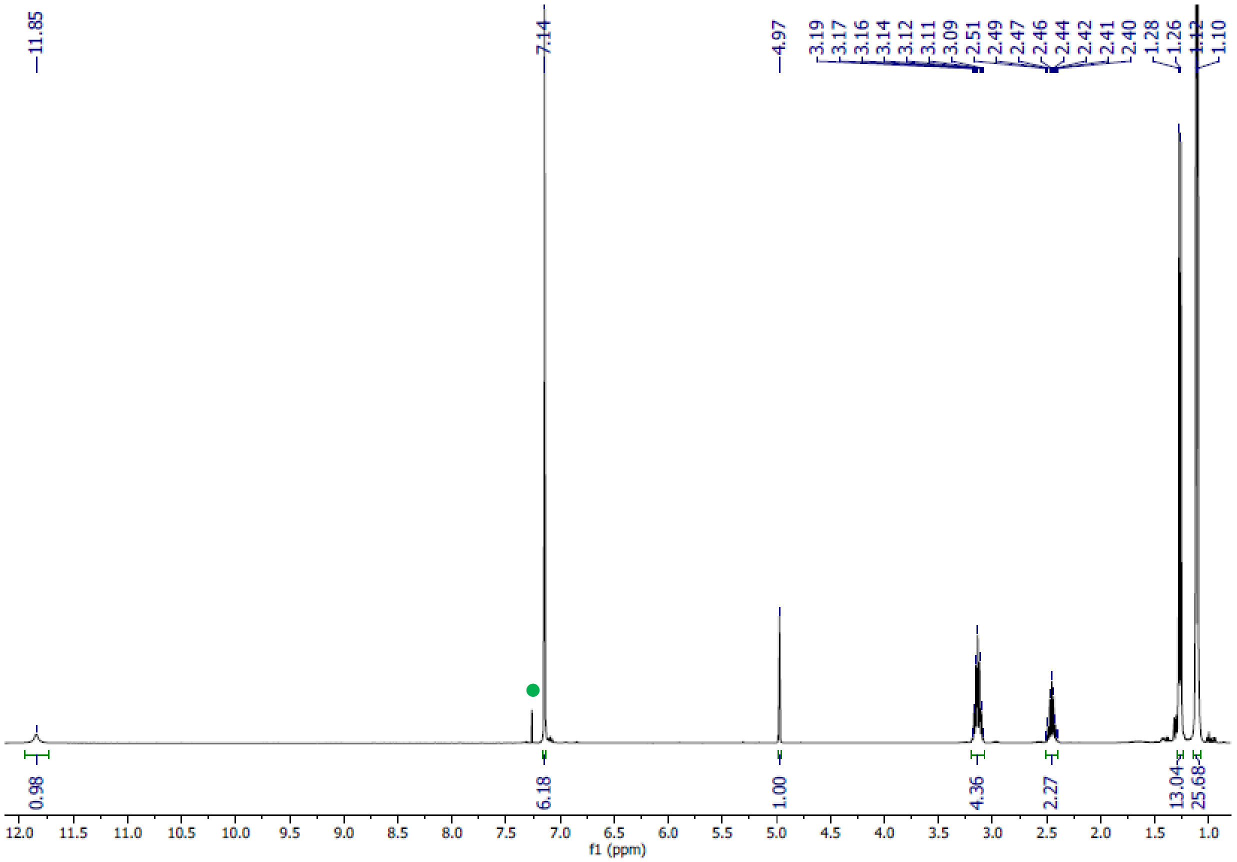

Figure S6. ^{1}H NMR spectrum (400.1 MHz, $CDCl_3$, 298 K) of ^{iPrDip}nacnacH **1.**

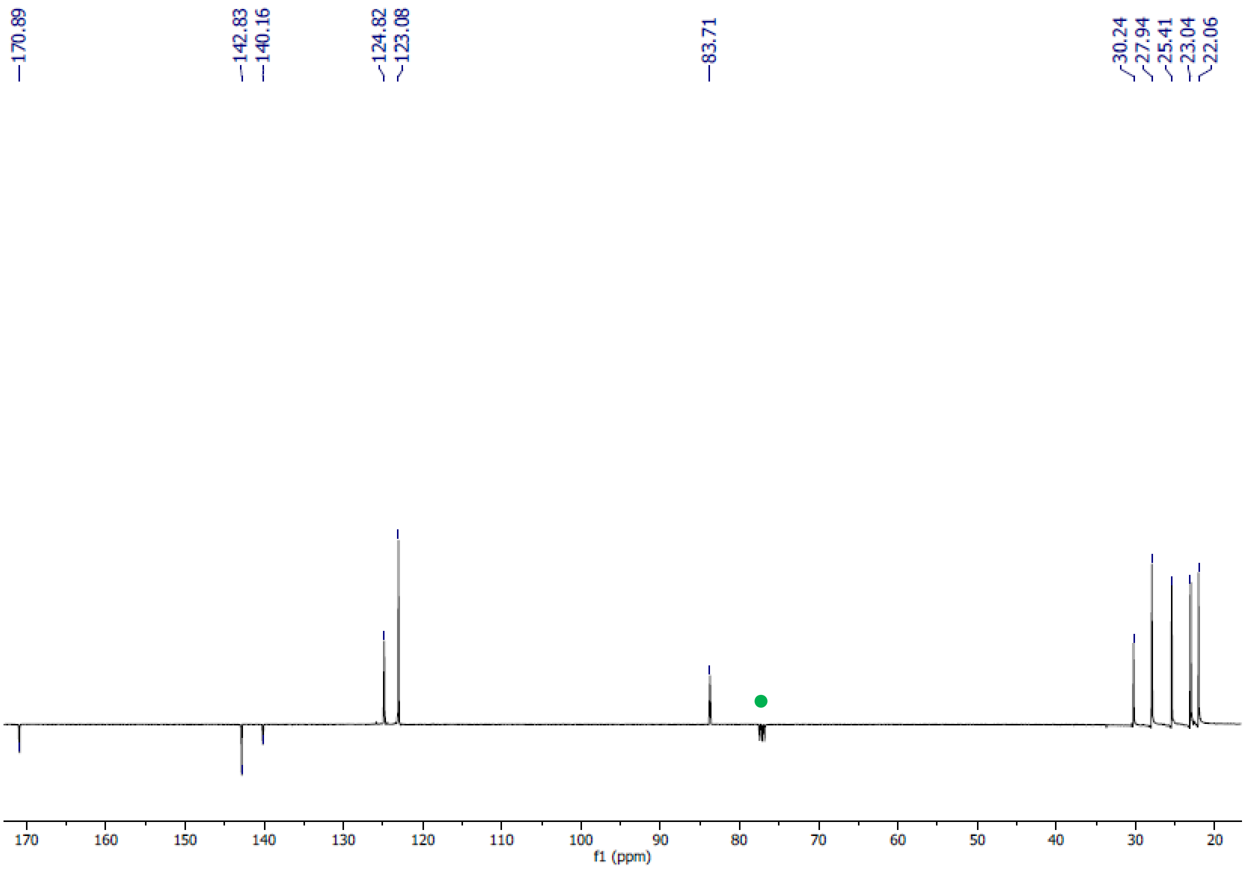

Figure S7. $^{13}C\{^{1}H\}$ (DEPT) NMR spectrum (100.5 MHz, $CDCl_3$, 298 K) of ^{iPrDip}nacnacH **1.**

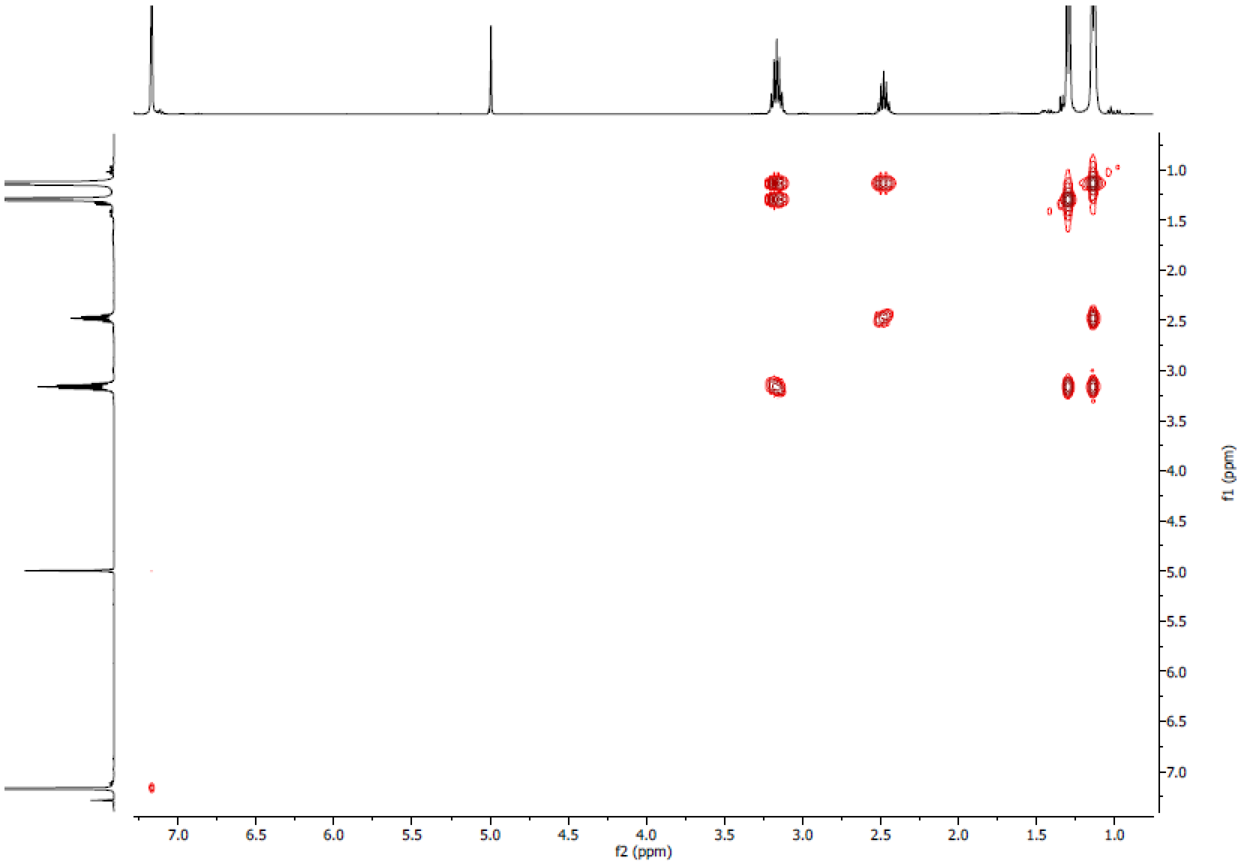

Figure S8. ^{1}H-^{1}H COSY NMR spectrum of ^{iPrDip}nacnacH **1.**

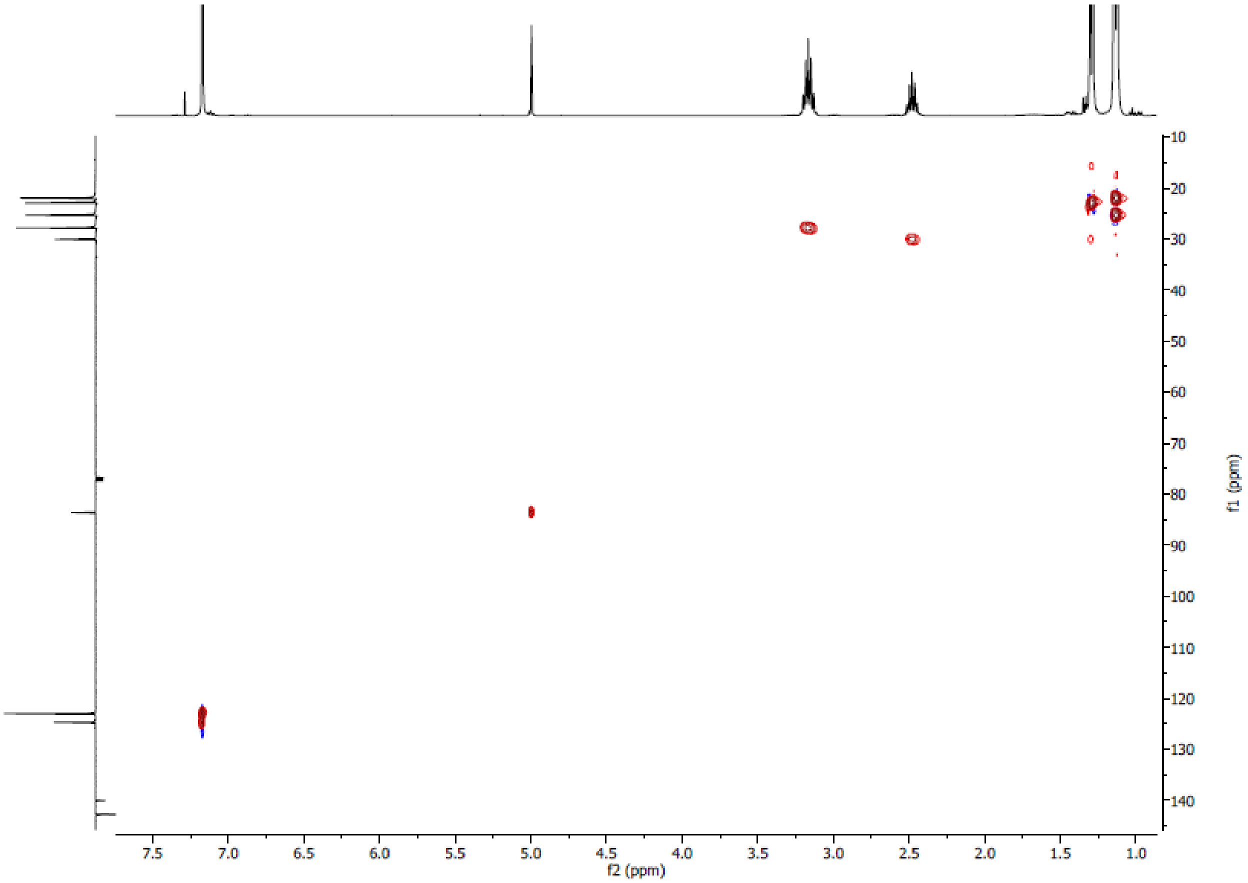

Figure S9. ^{1}H-^{13}C HSQC NMR spectrum of ^{iPrDip}nacnacH **1.**

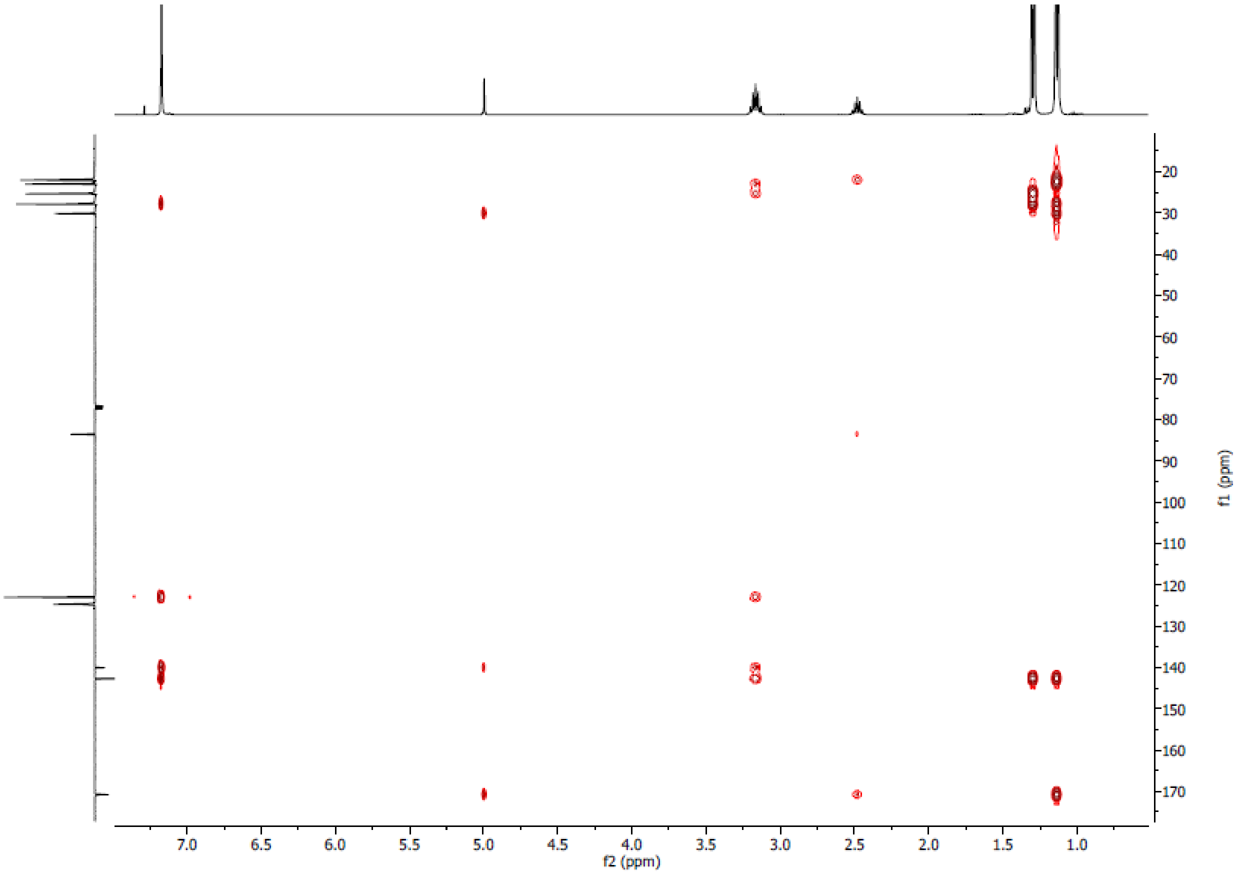

Figure S10. ^{1}H-^{13}C HMBC NMR spectrum of ^{iPrDip}nacnacH **1.**

2.2 NMR spectra of [(^{iPrDip}nacnac)MgnBu] 2

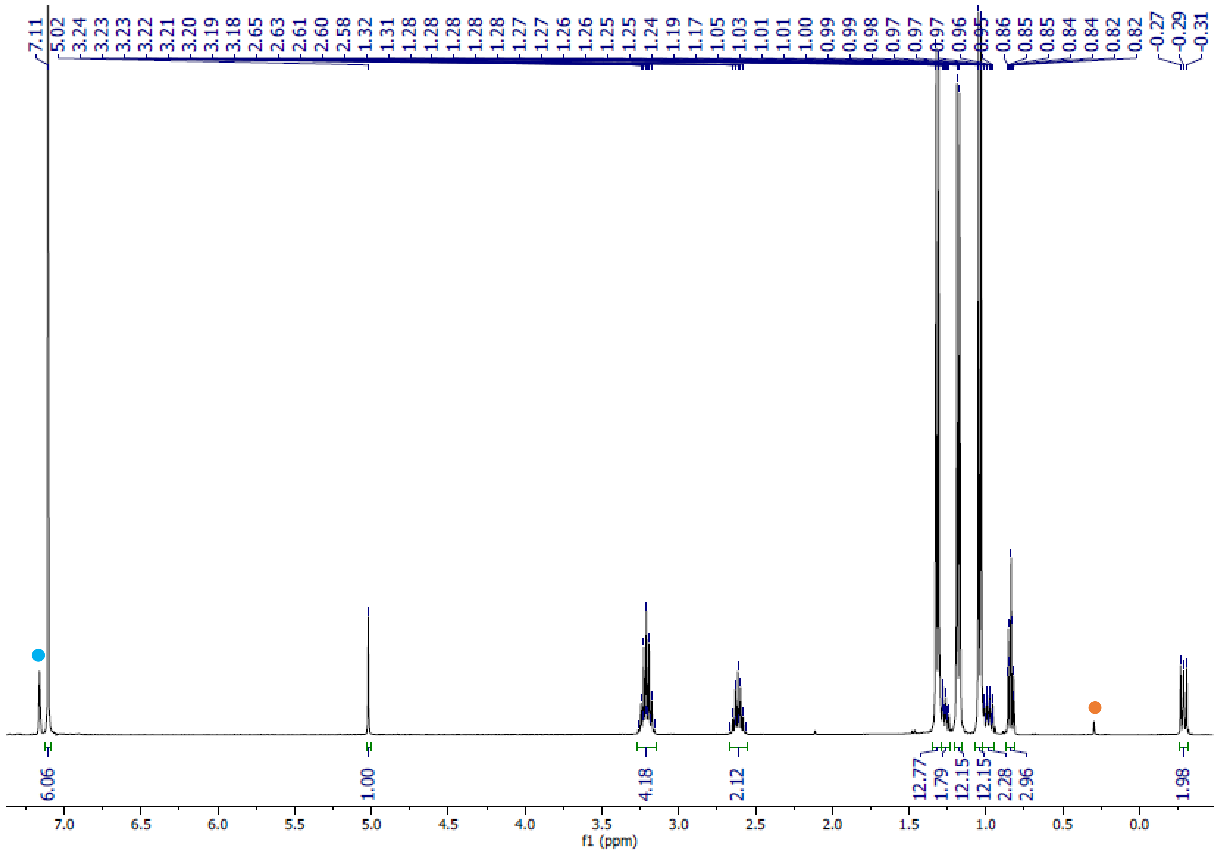

Figure S11. ^{1}H NMR spectrum (400.1 MHz, C_6D_6, 298 K) of [(^{iPrDip}nacnac)MgnBu] **2**.

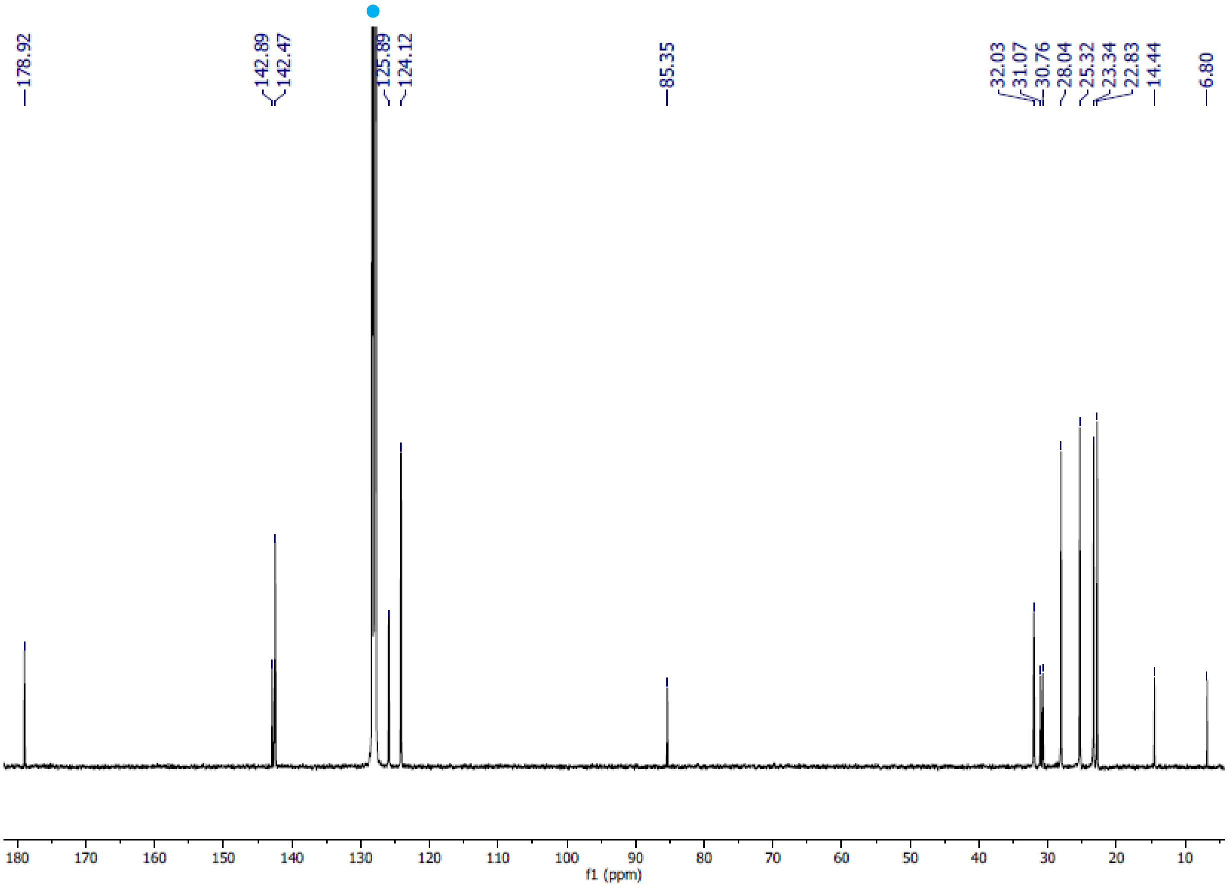

Figure S12. ^{13}C{^{1}H} NMR spectrum (100.5 MHz, C_6D_6, 298 K) of [(^{iPrDip}nacnac)MgnBu] **2**.

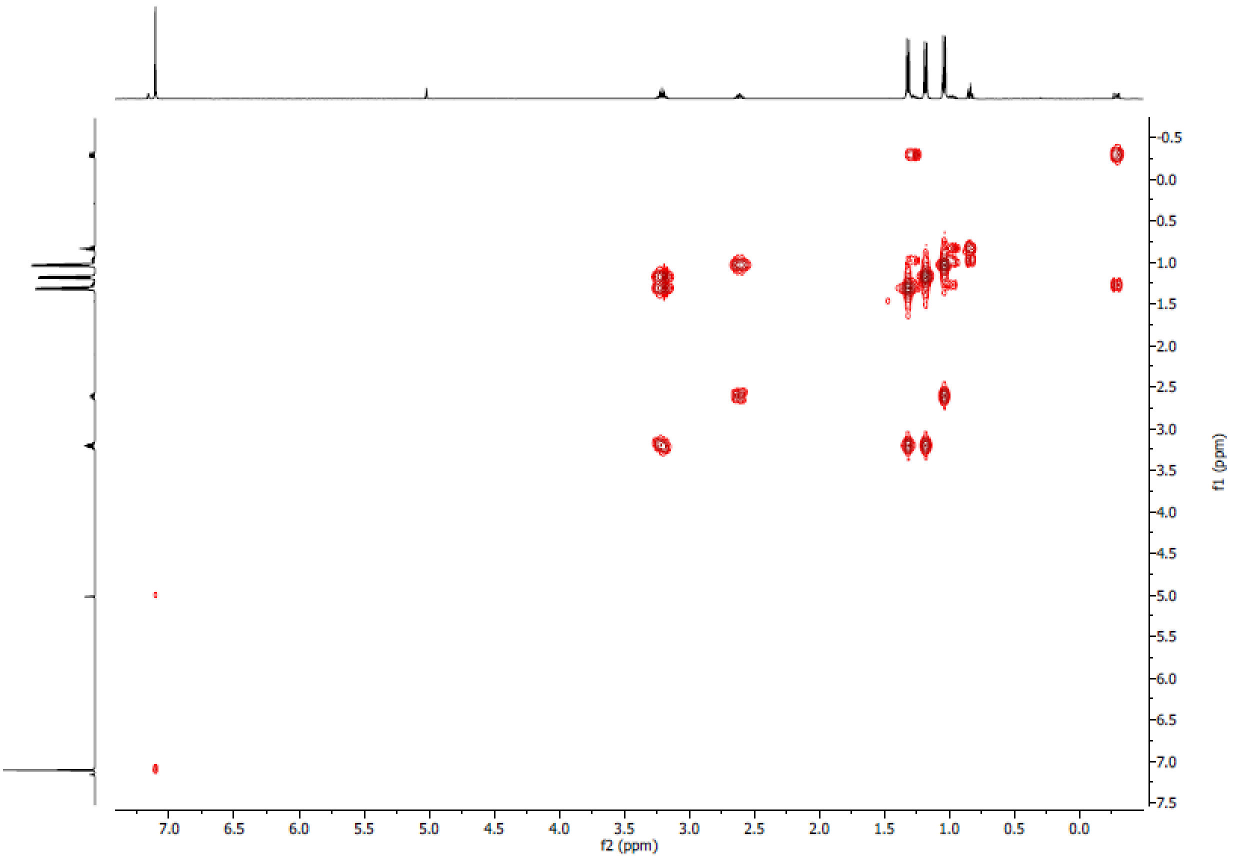

Figure S13. ^{1}H-^{1}H COSY NMR spectrum of [(^{i}PrDipnacnac)Mg*n*Bu] **2**.

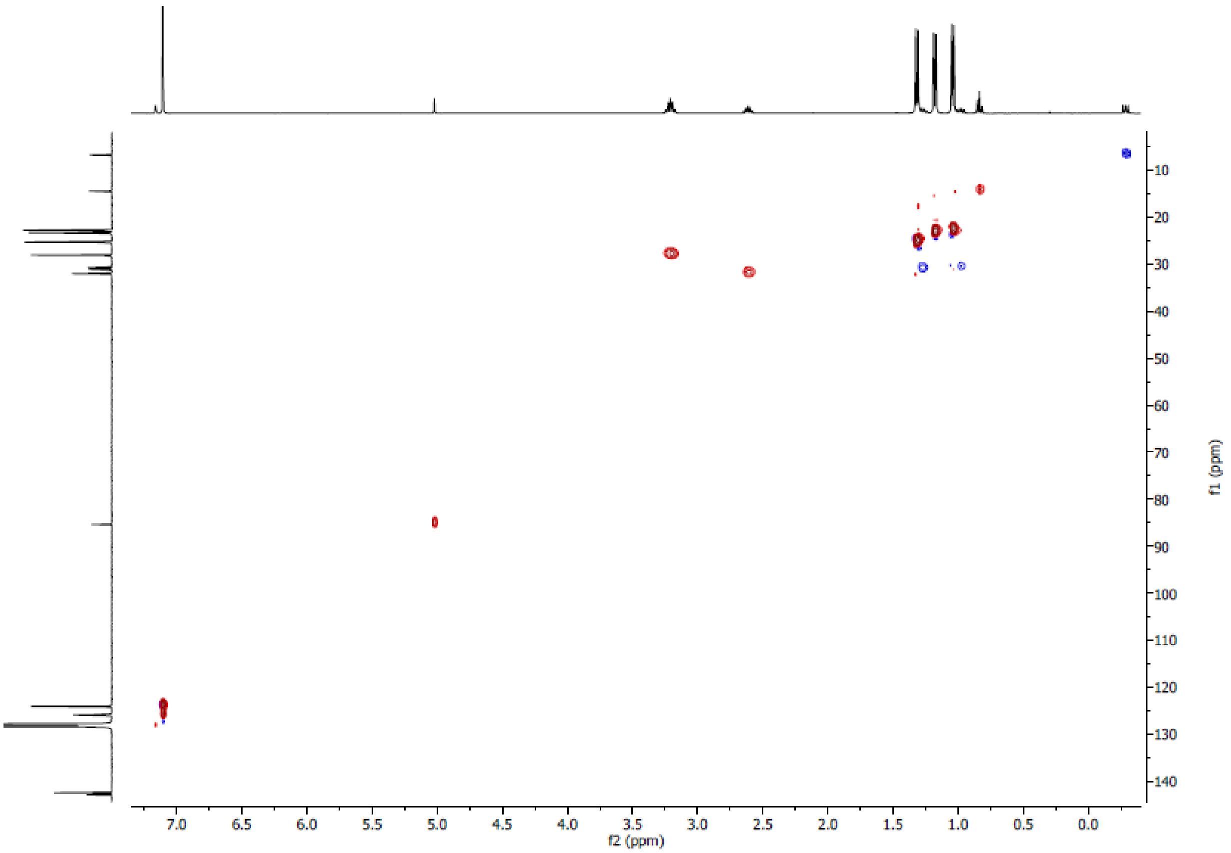

Figure S14. ^{1}H-^{13}C HSQC NMR spectrum of [(^{i}PrDipnacnac)Mg*n*Bu] **2**.

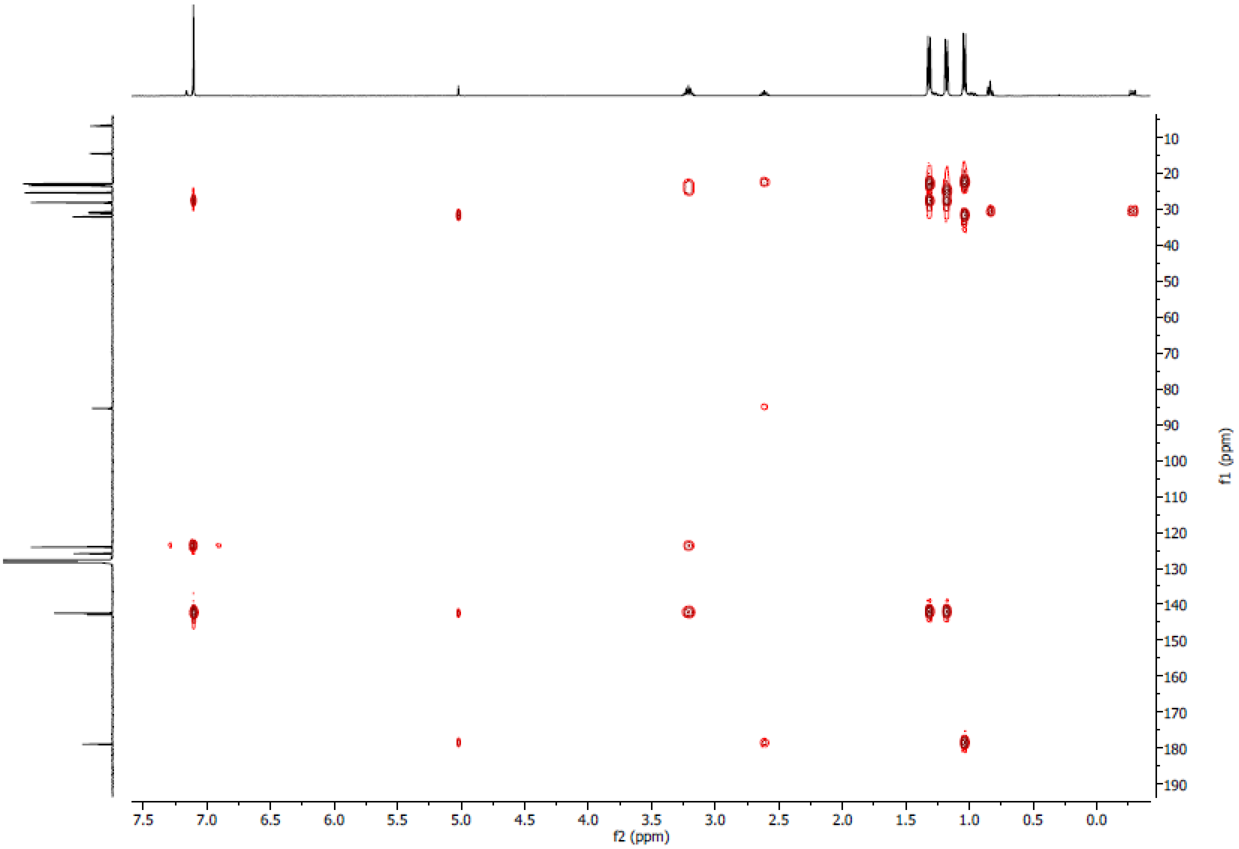

Figure S15. ^{1}H-^{13}C HMBC NMR spectrum of [(^{iPrDip}nacnac)Mg*n*Bu] **2**.

2.3 NMR spectra of [{(^{iPrDip}nacnac)Mg(μ-I)}$_2$] 3

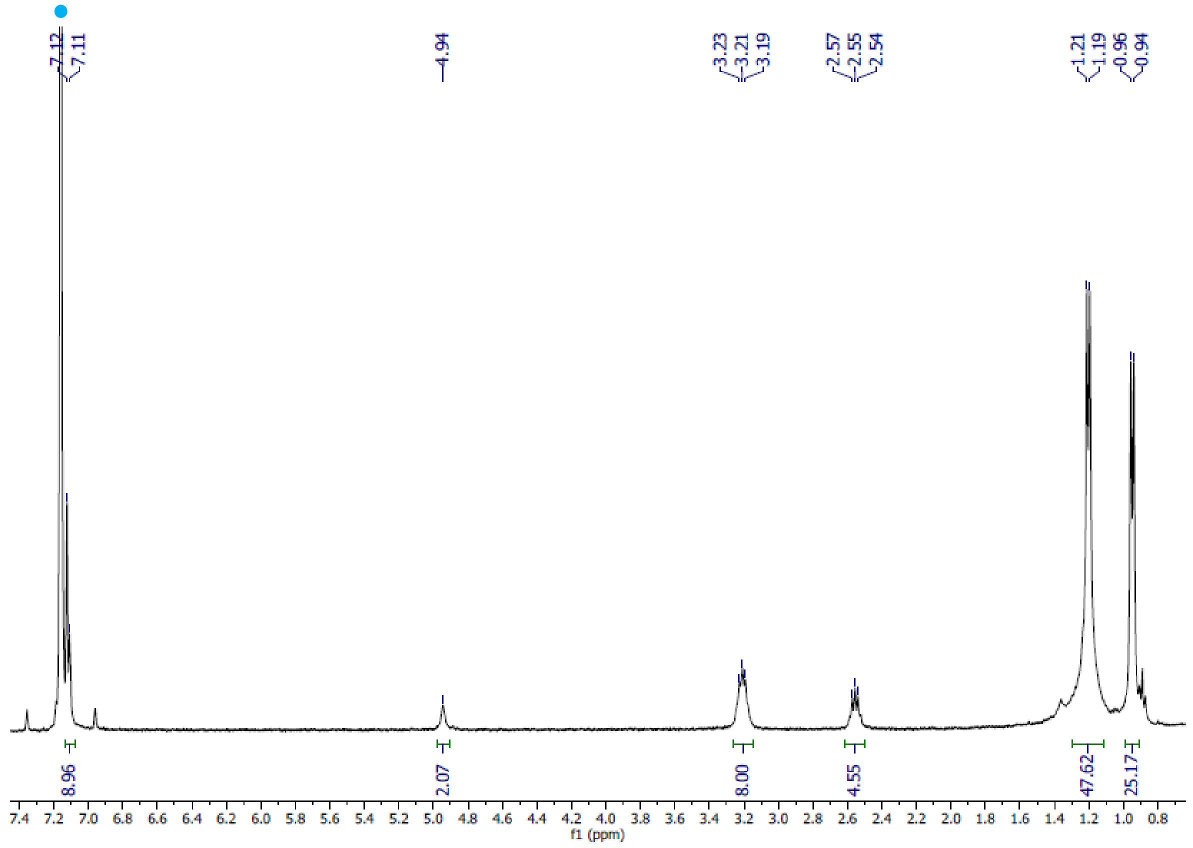

Figure S16. ^{1}H NMR spectrum (400.1 MHz, C_6D_6, 298 K) of [{(^{iPrDip}nacnac)Mg(μ-I)}$_2$] **3**.

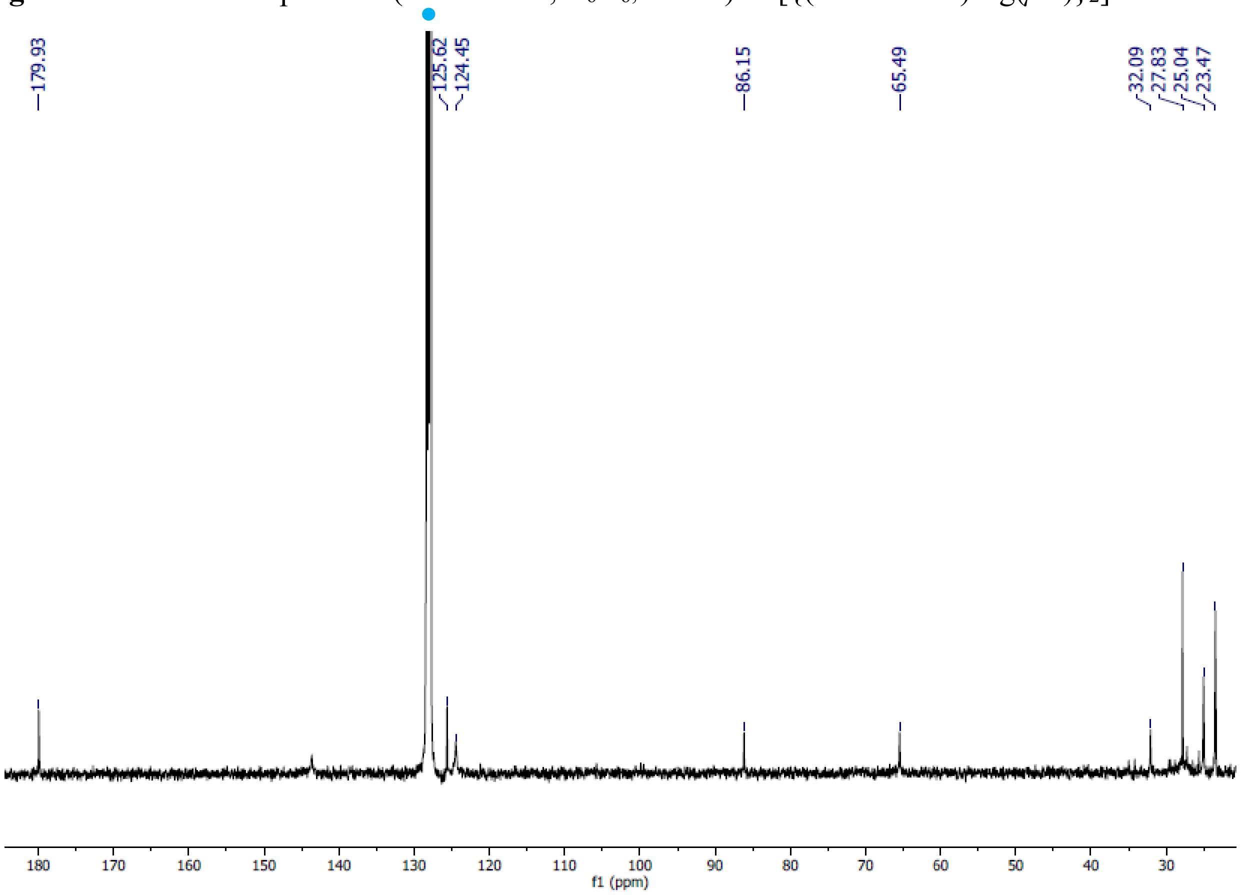

Figure S17. ^{13}C{^{1}H} NMR spectrum (100.5 MHz, C_6D_6, 298 K) of [{(^{iPrDip}nacnac)Mg(μ-I)}$_2$] **3**.

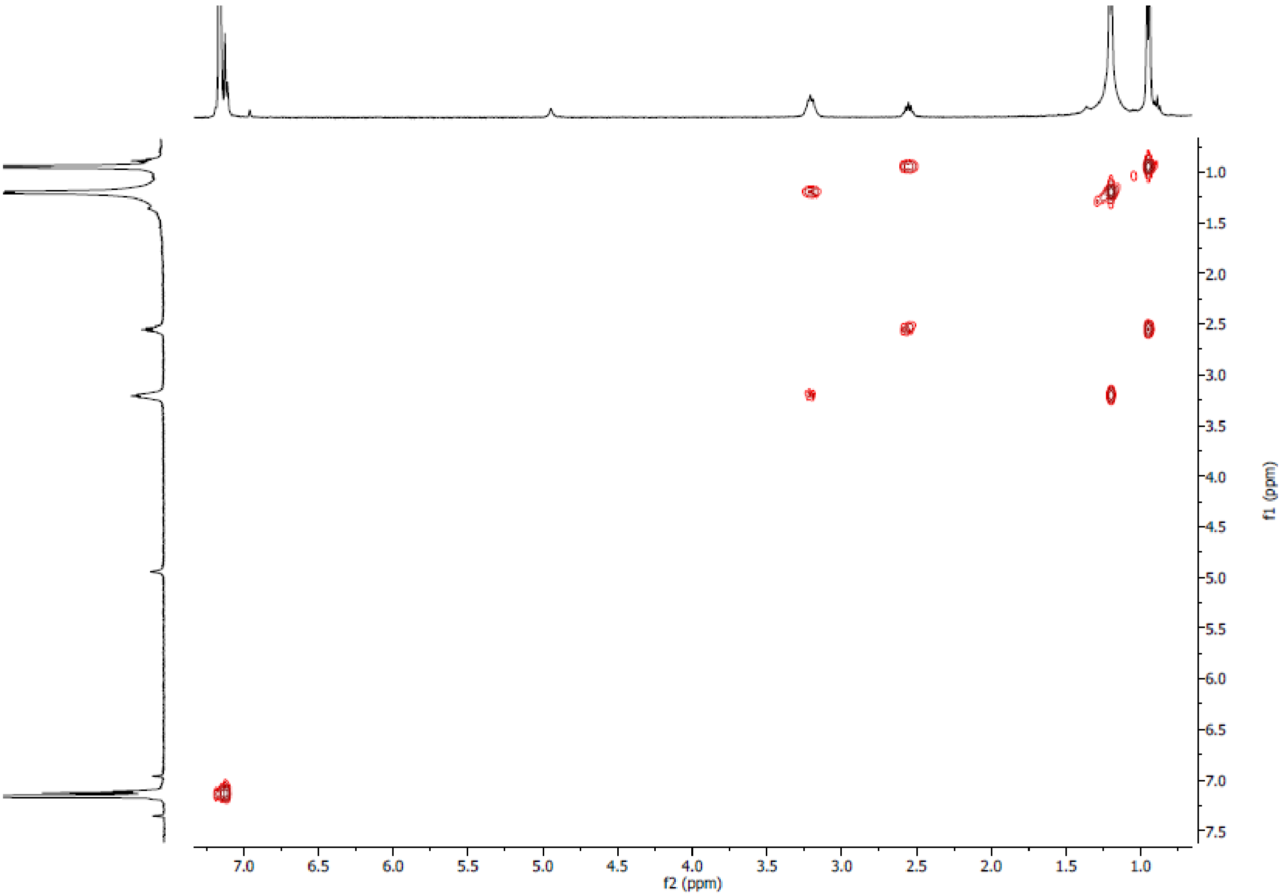

Figure S18. ^{1}H-^{1}H COSY NMR spectrum of [{(^{iPrDip}nacnac)Mg(μ-I)}$_2$] **3**.

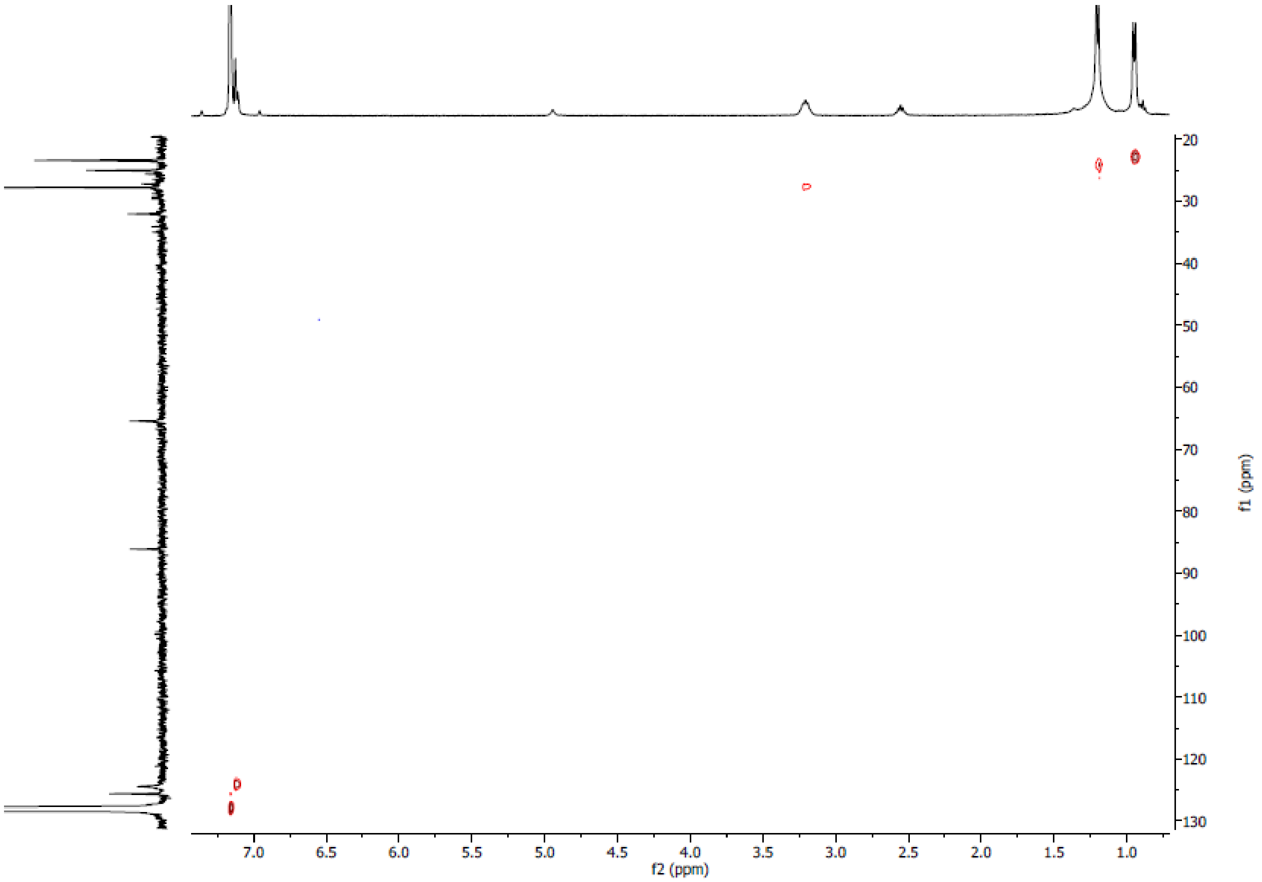

Figure S19. ^{1}H-^{13}C HSQC NMR spectrum of [{(^{iPrDip}nacnac)Mg(μ-I)}$_2$] **3**.

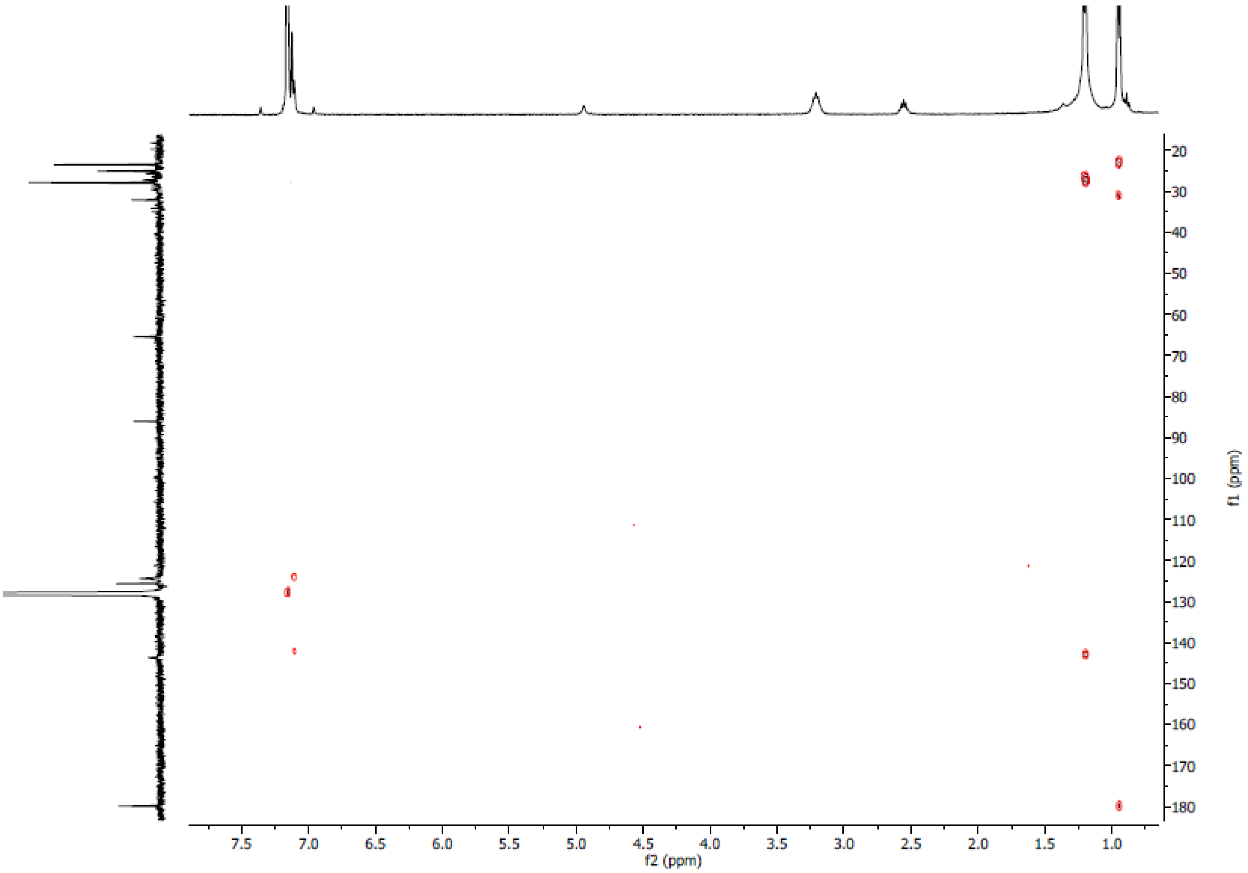

Figure S20. ^{1}H-^{13}C HMBC NMR spectrum of [{(^{iPrDip}nacnac)Mg(μ-I)}$_2$] **3**.

2.4 NMR spectra of [(^{iPrDip}nacnac)Mg(THF)I]

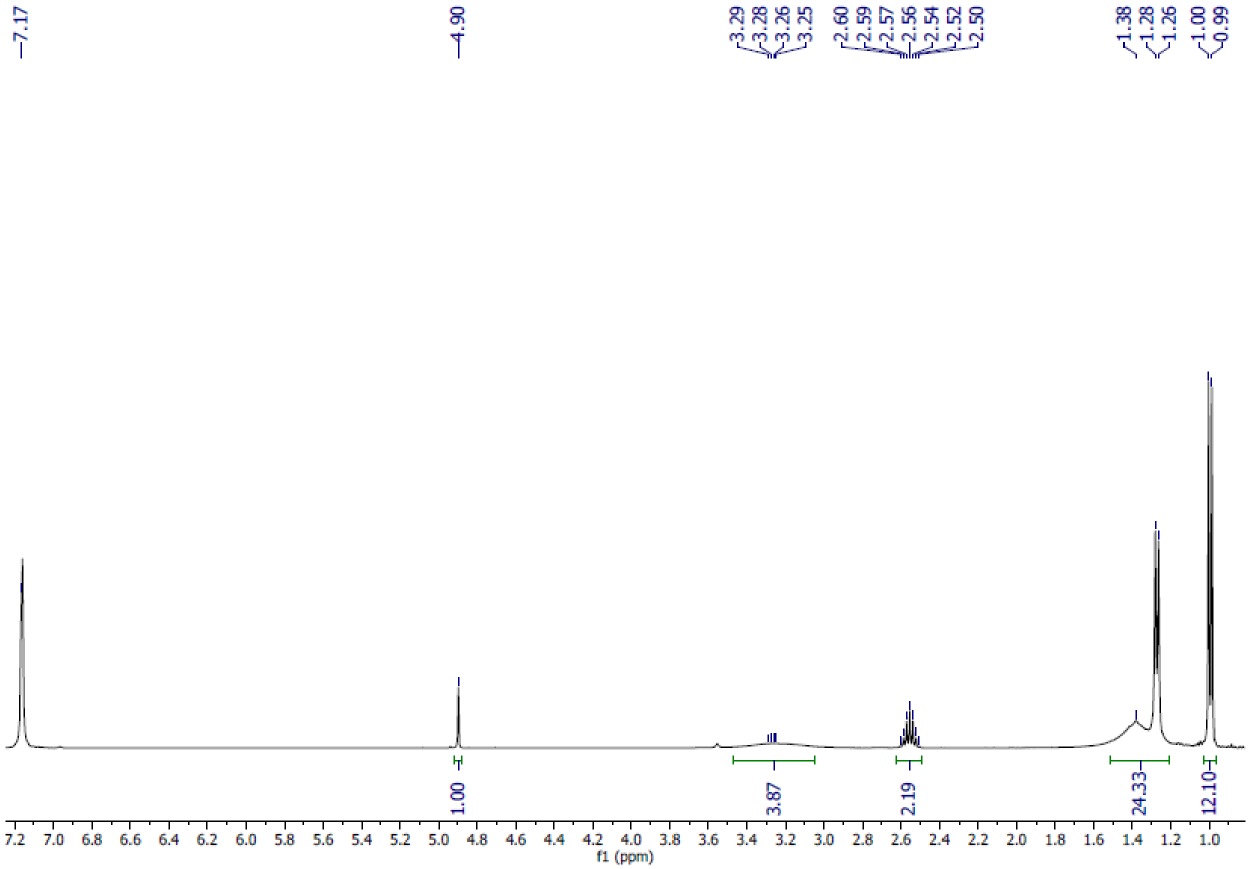

Figure S21. ^{1}H NMR spectrum (400.1 MHz, C_6D_6, 298 K) of [(^{iPrDip}nacnac)Mg(THF)I].

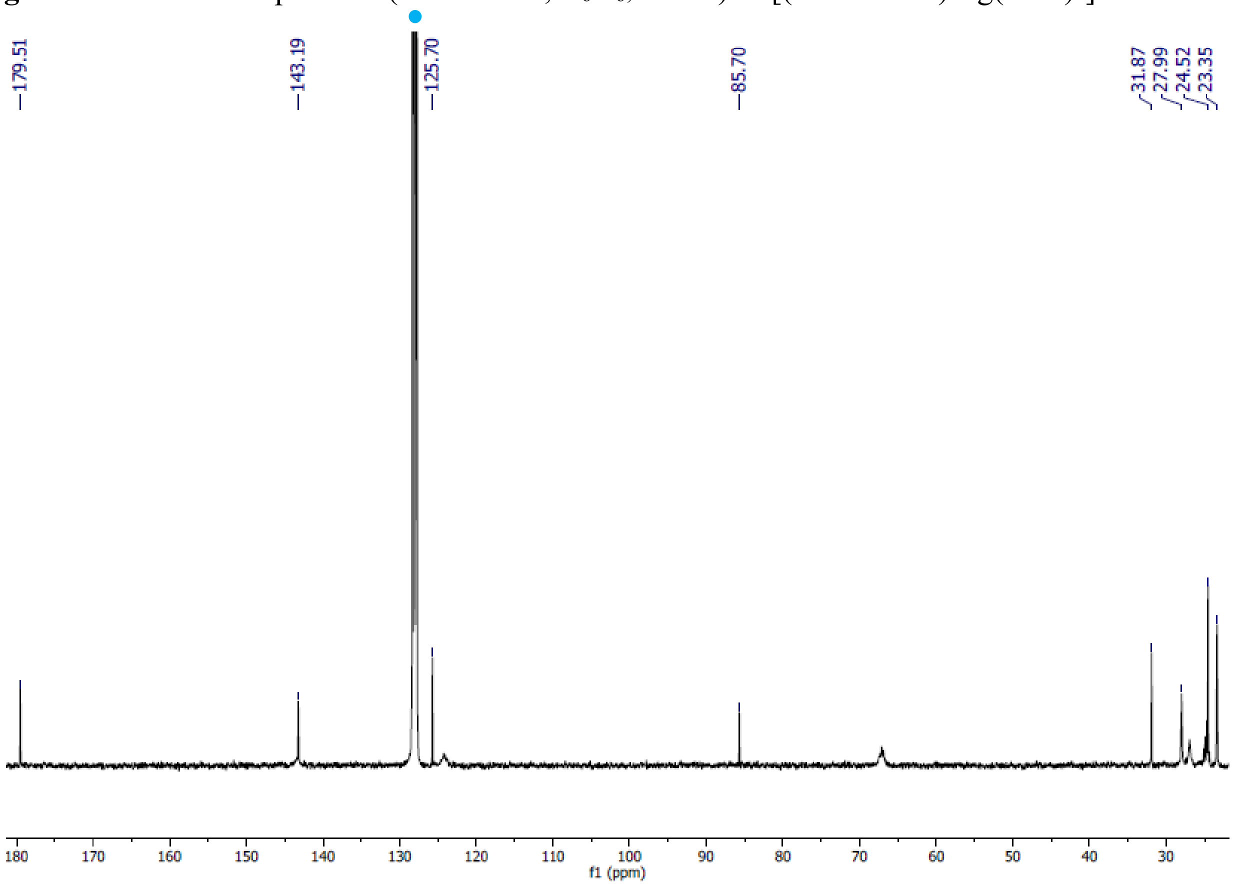

Figure S22. $^{13}C\{^{1}H\}$ NMR spectrum (100.5 MHz, C_6D_6, 298 K) of [(^{iPrDip}nacnac)Mg(THF)I].

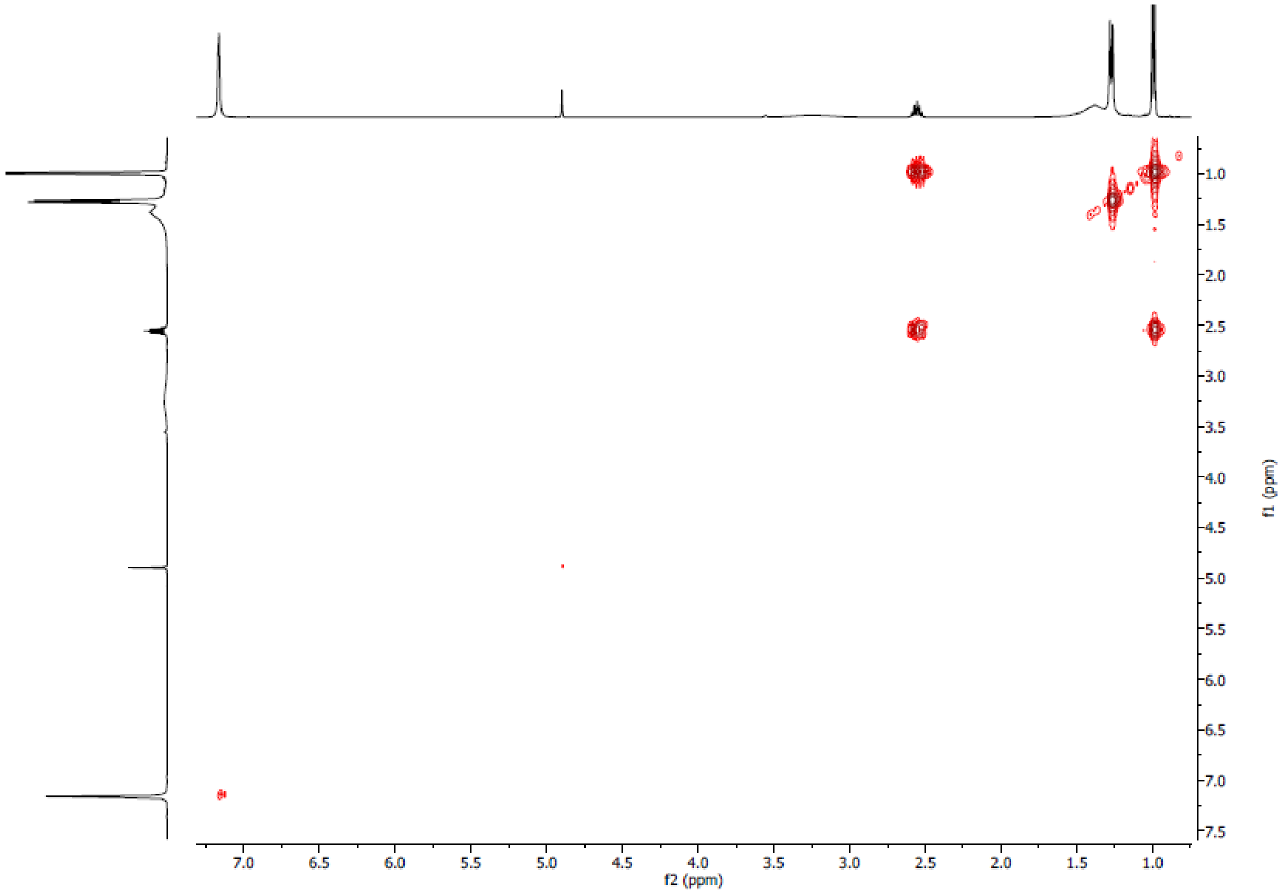

Figure S23. ^{1}H-^{1}H COSY NMR spectrum of [(^{iPrDip}nacnac)Mg(THF)I].

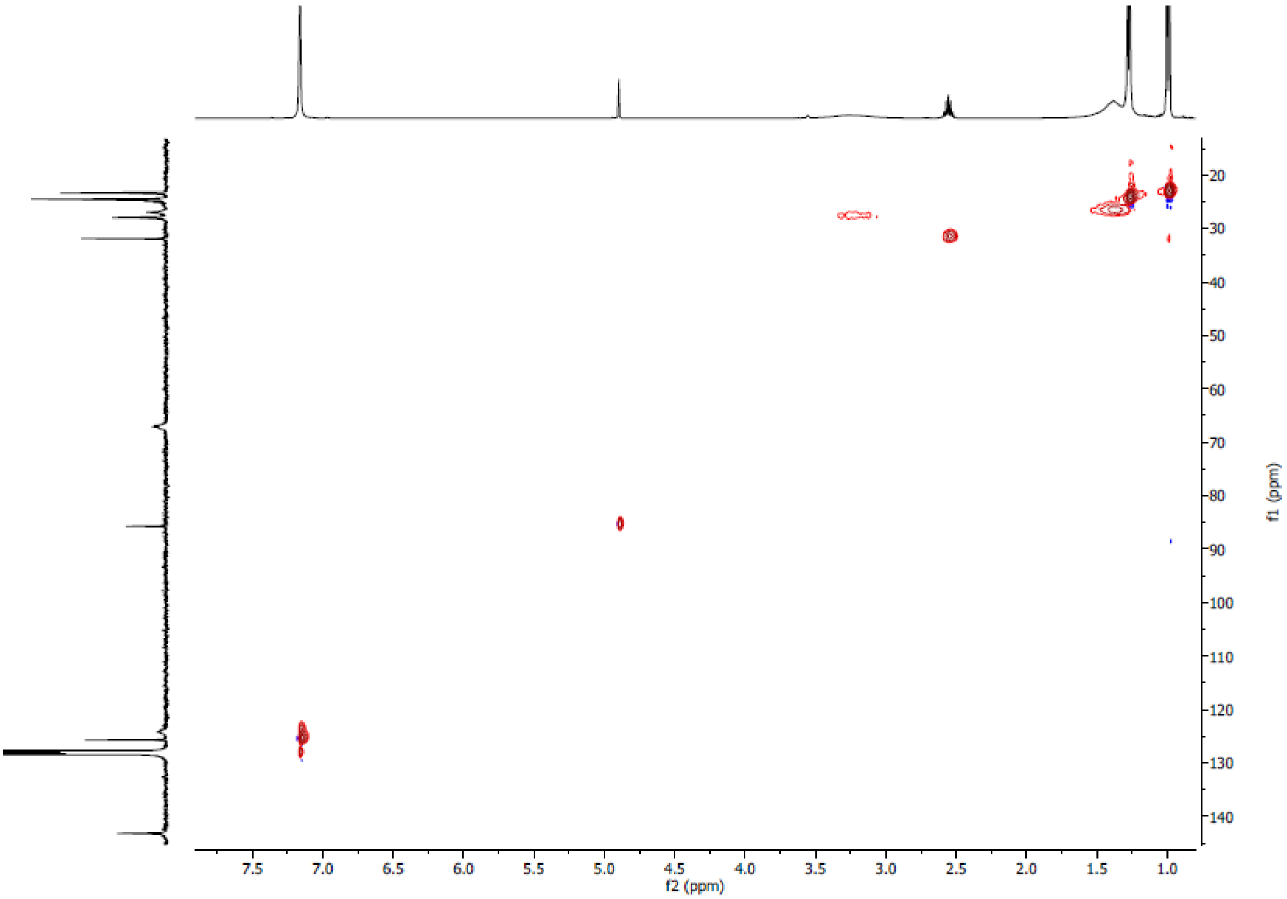

Figure S24. ^{1}H-^{13}C HSQC NMR spectrum of [(^{iPrDip}nacnac)Mg(THF)I].

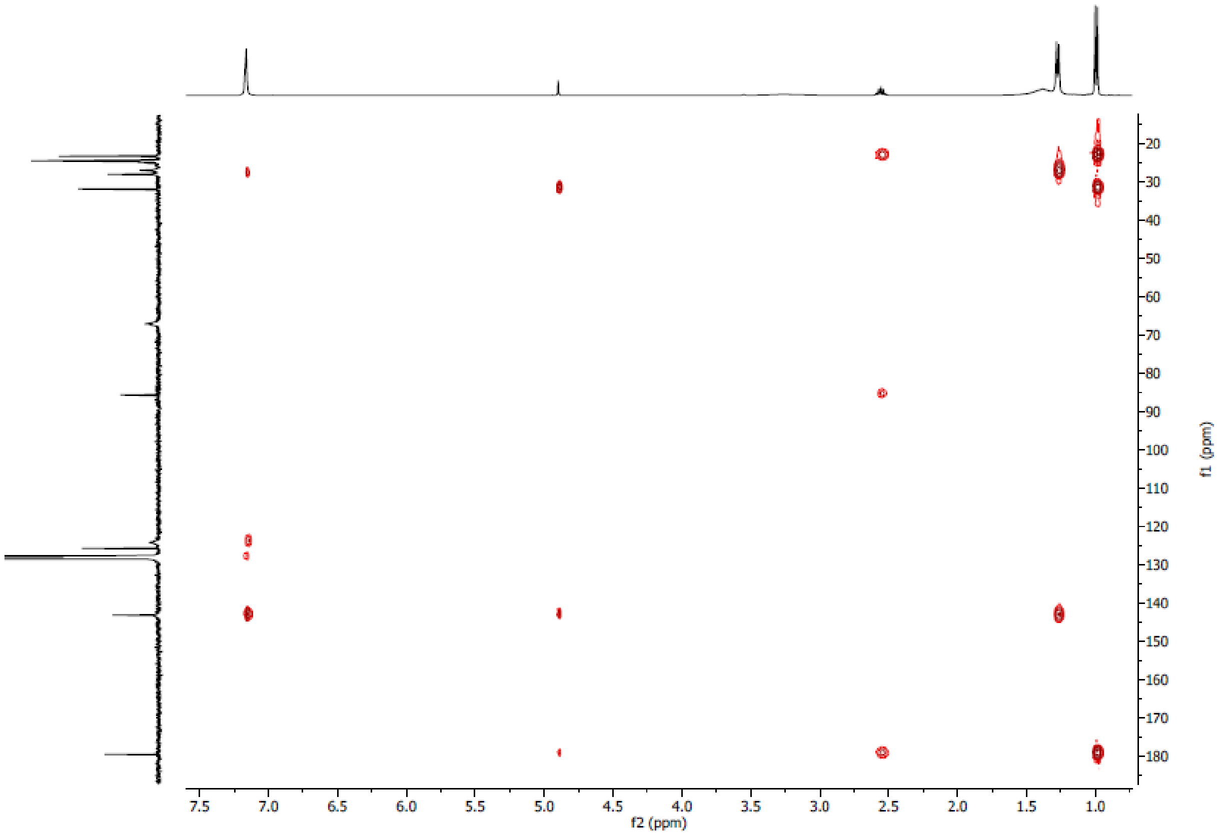

Figure S25. ^{1}H-^{13}C HMBC NMR spectrum of [(^{iPrDip}nacnac)Mg(THF)I].

2.5 NMR spectra of [{(^{iPrDip}nacnac)Mg}$_2$] 4

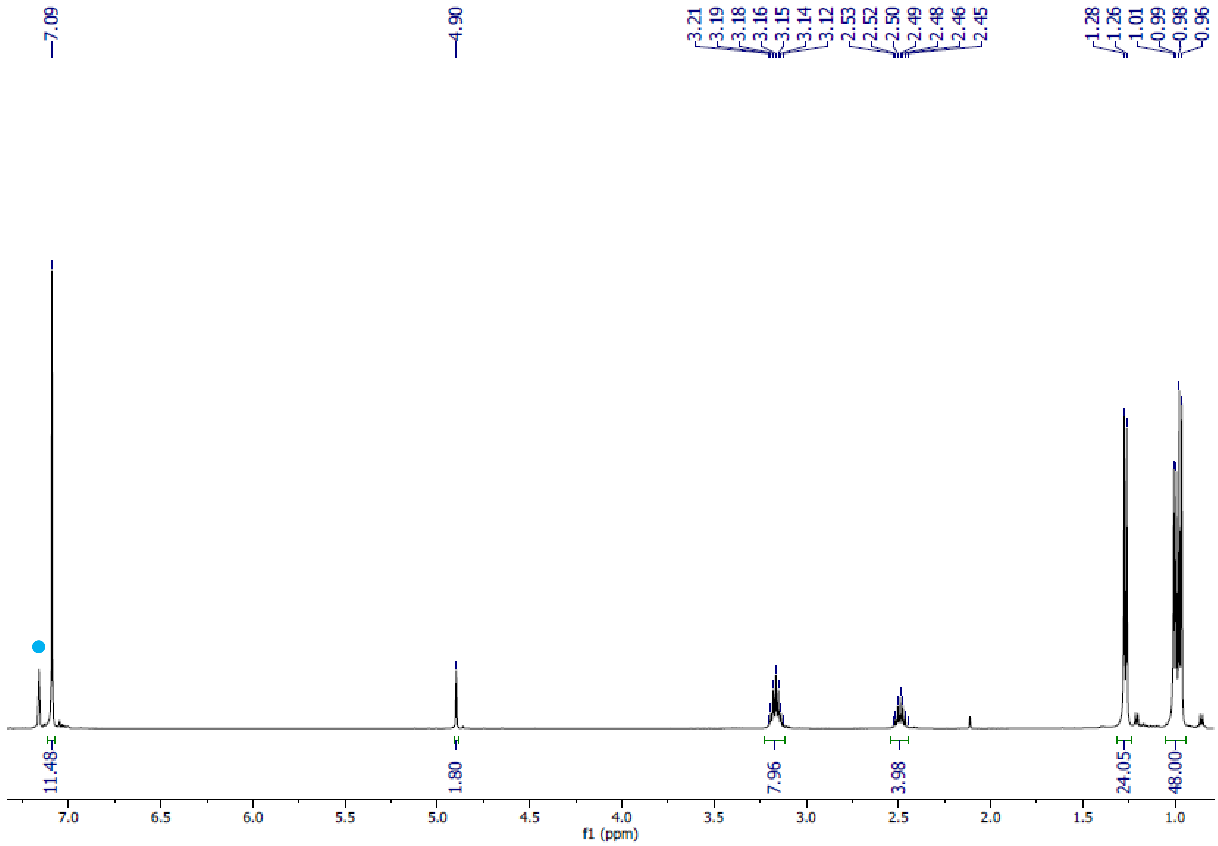

Figure S26. ^{1}H NMR spectrum (400.1 MHz, C_6D_6, 298 K) of [{(^{iPrDip}nacnac)Mg}$_2$] **4**.

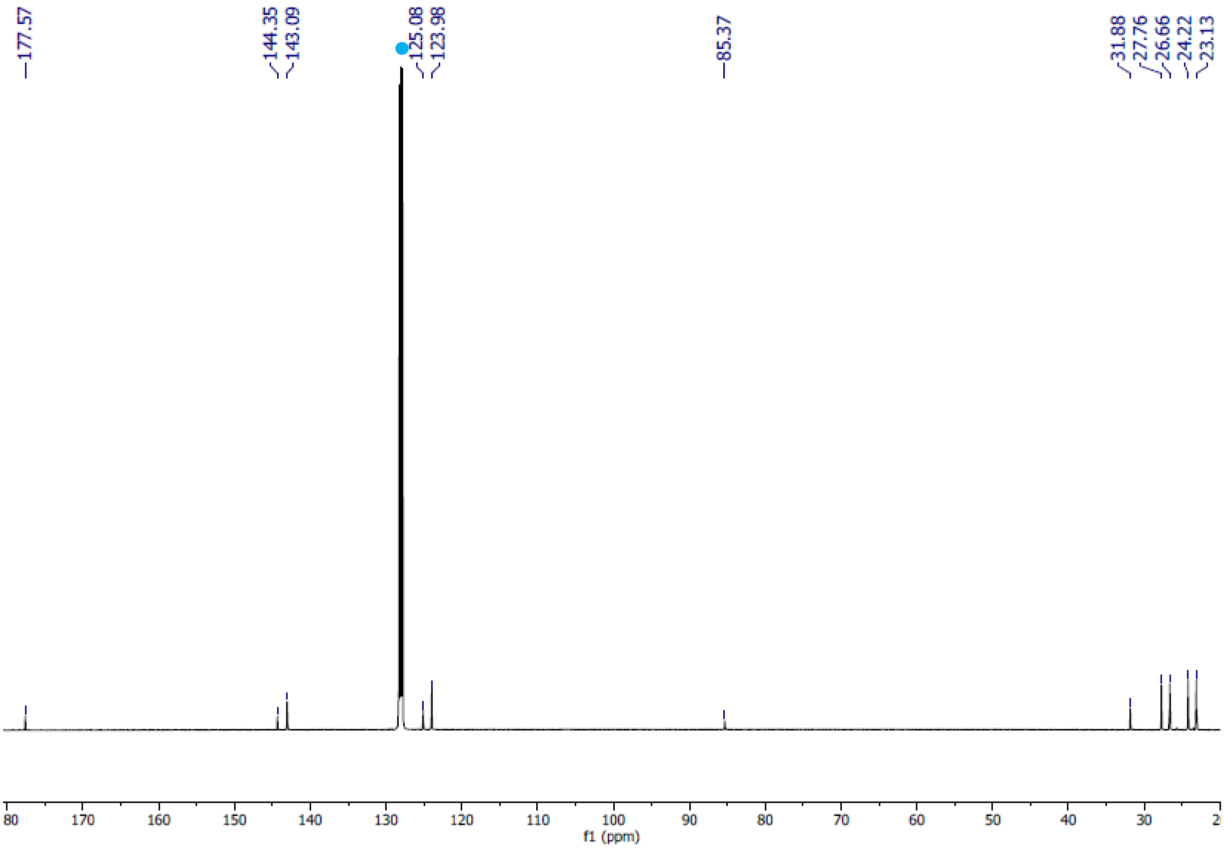

Figure S27. ^{13}C{^{1}H} NMR spectrum (100.5 MHz, C_6D_6, 298 K) of [{(^{iPrDip}nacnac)Mg}$_2$] **4**.

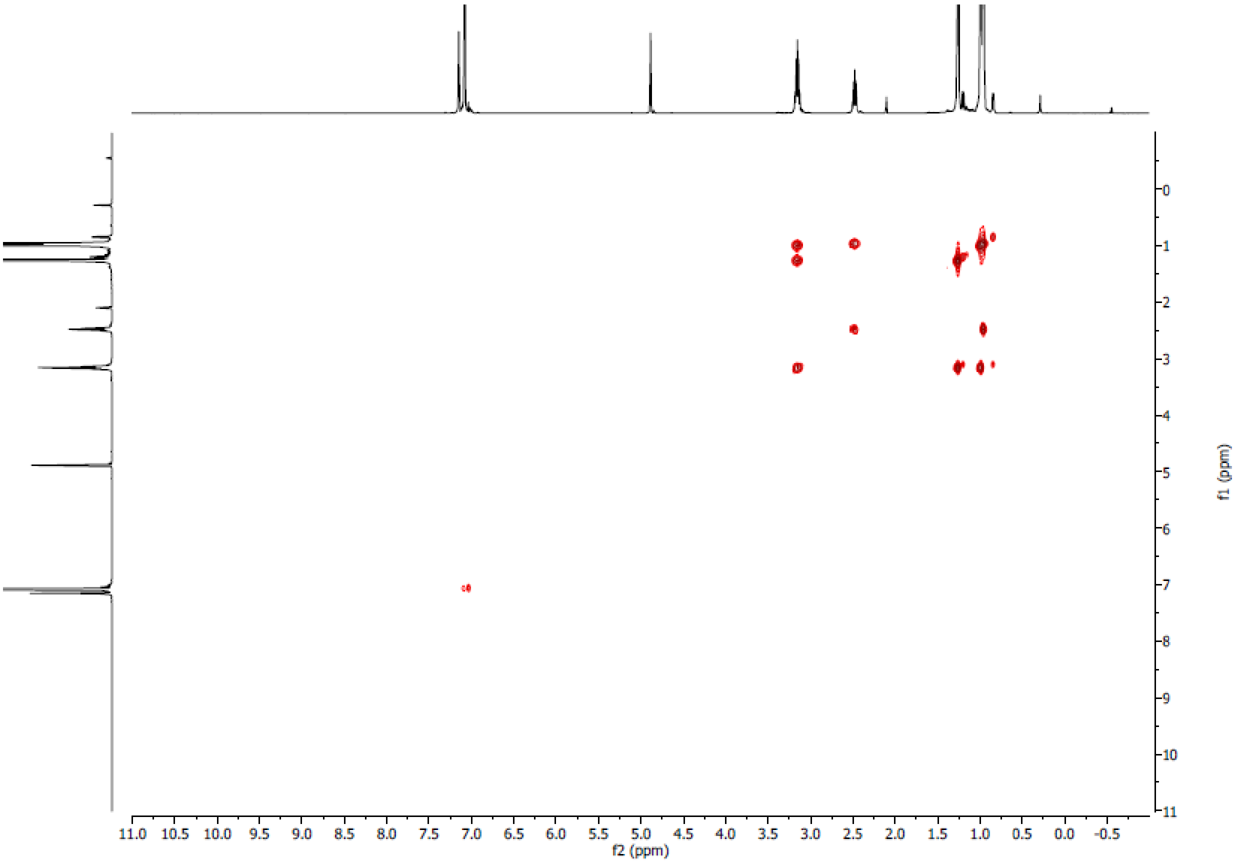

Figure S28. ^{1}H-^{1}H COSY NMR spectrum of [{(^{iPrDip}nacnac)Mg}$_2$] **4**.

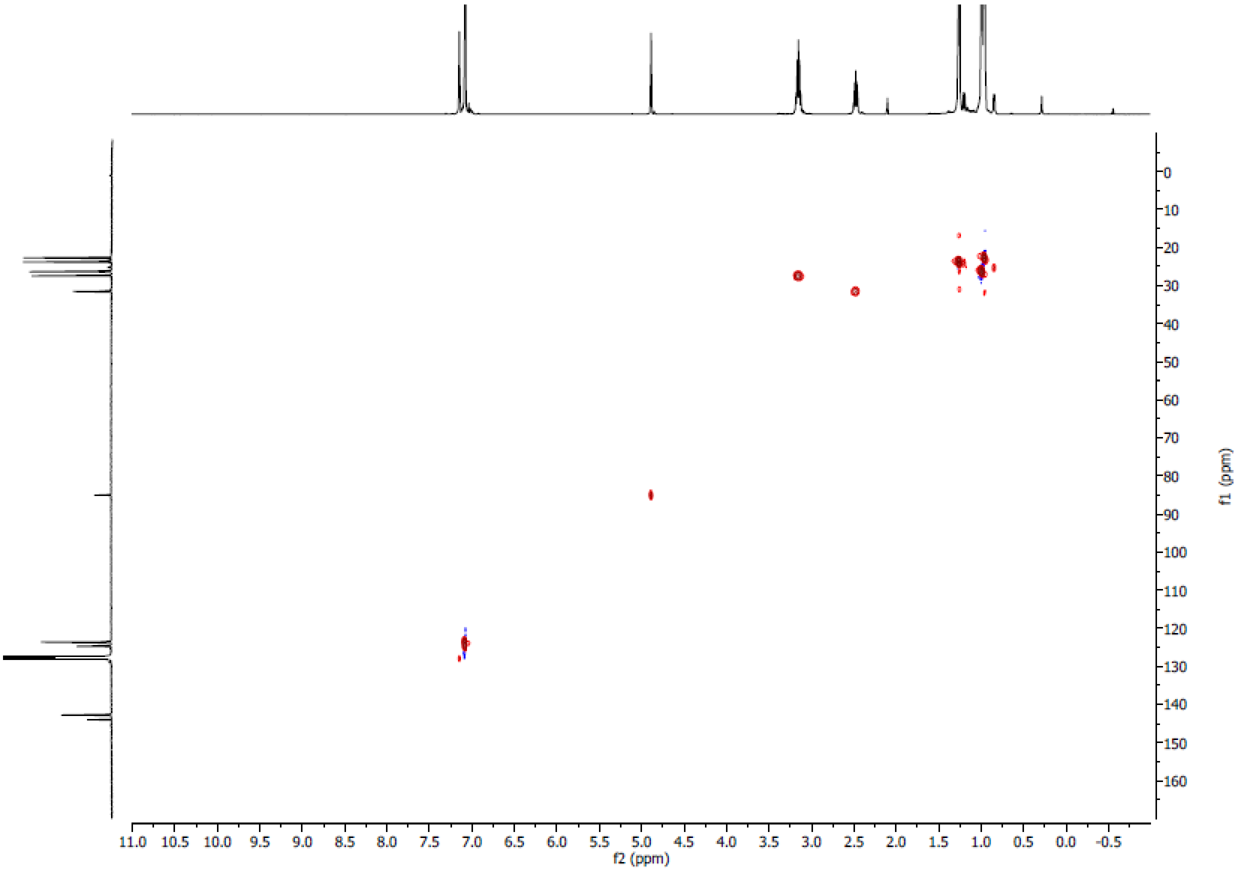

Figure S29. ^{1}H-^{13}C HSQC NMR spectrum of [{(^{iPrDip}nacnac)Mg}$_2$] **4**.

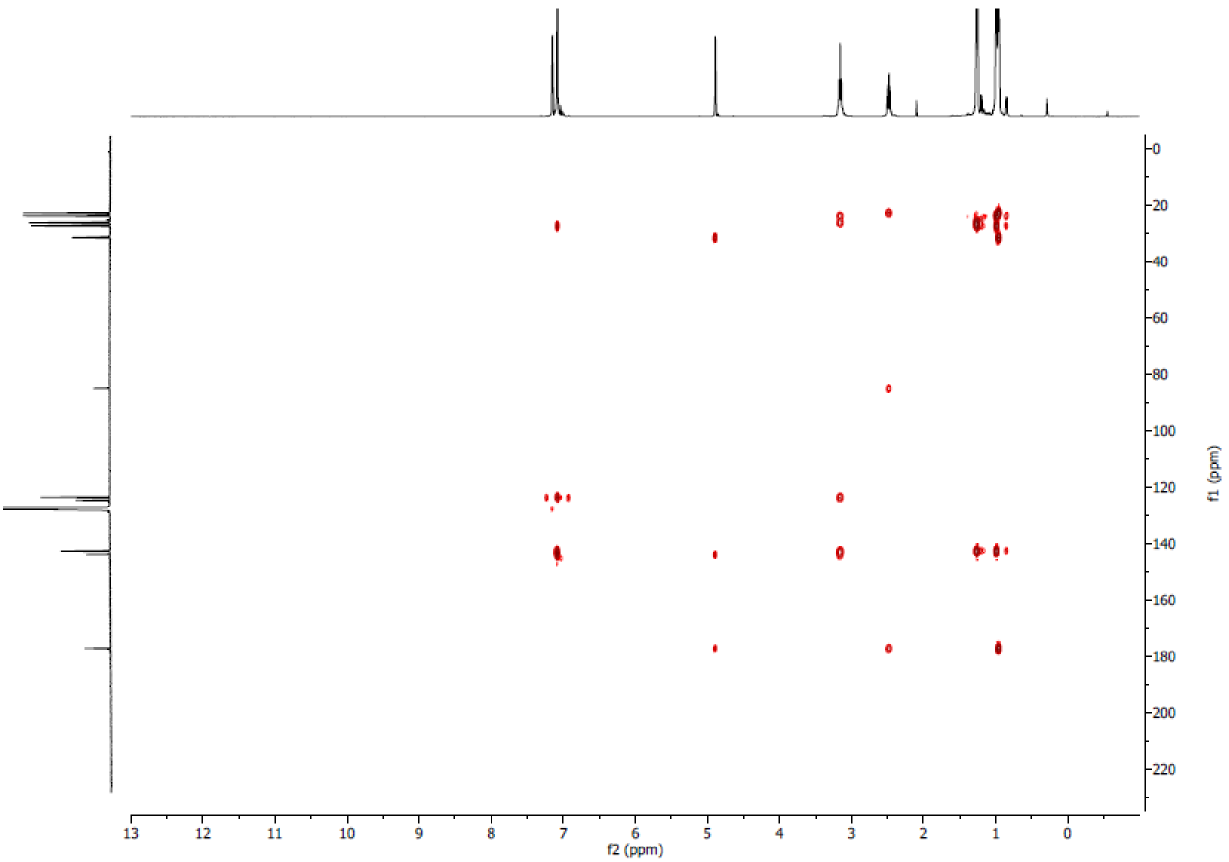

Figure S30. ^{1}H-^{13}C HMBC NMR spectrum of [{(^{iPrDip}nacnac)Mg}$_2$] **4**.

2.6 NMR spectra of [{(^{iPrDip}nacnac)Mg(μ-H)}$_2$] 5

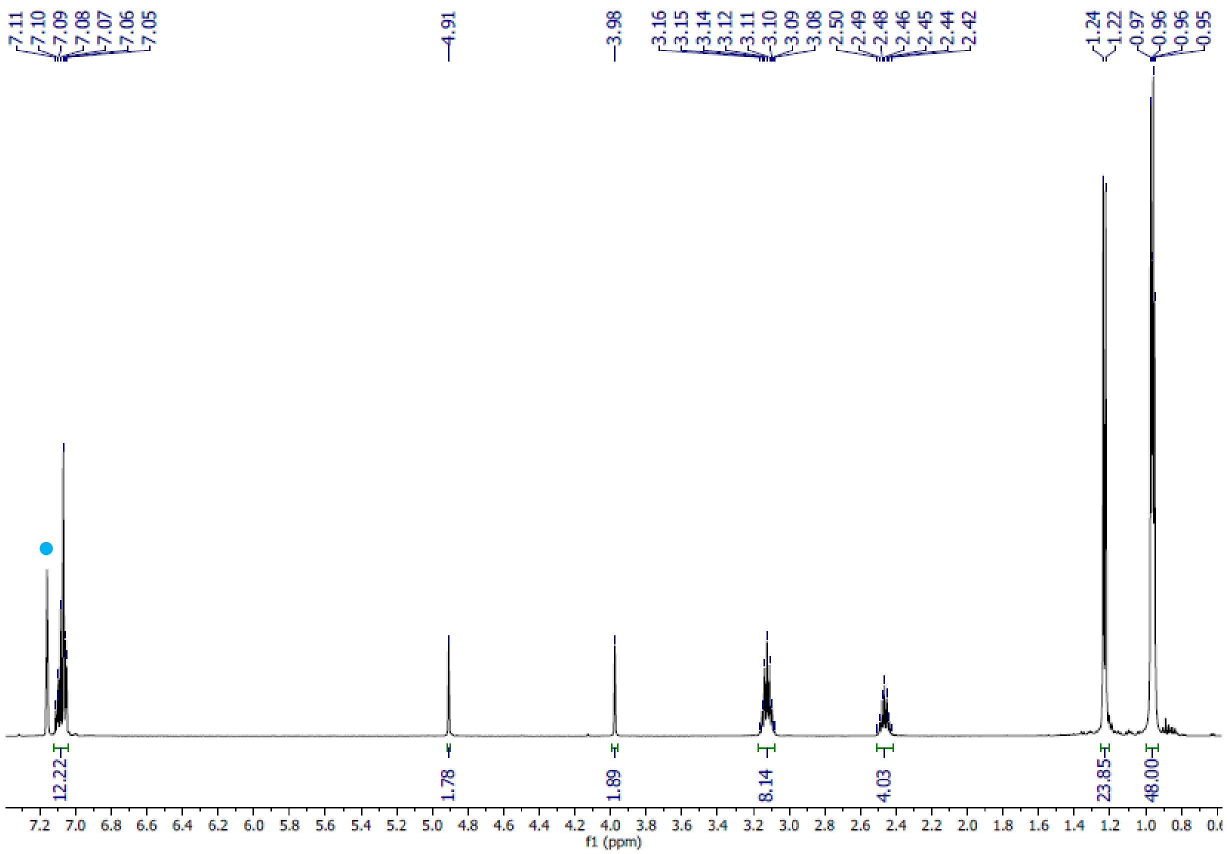

Figure S31. ^{1}H NMR spectrum (400.1 MHz, C_6D_6, 298 K) of [{(^{iPrDip}nacnac)Mg(μ-H)}$_2$] **5**.

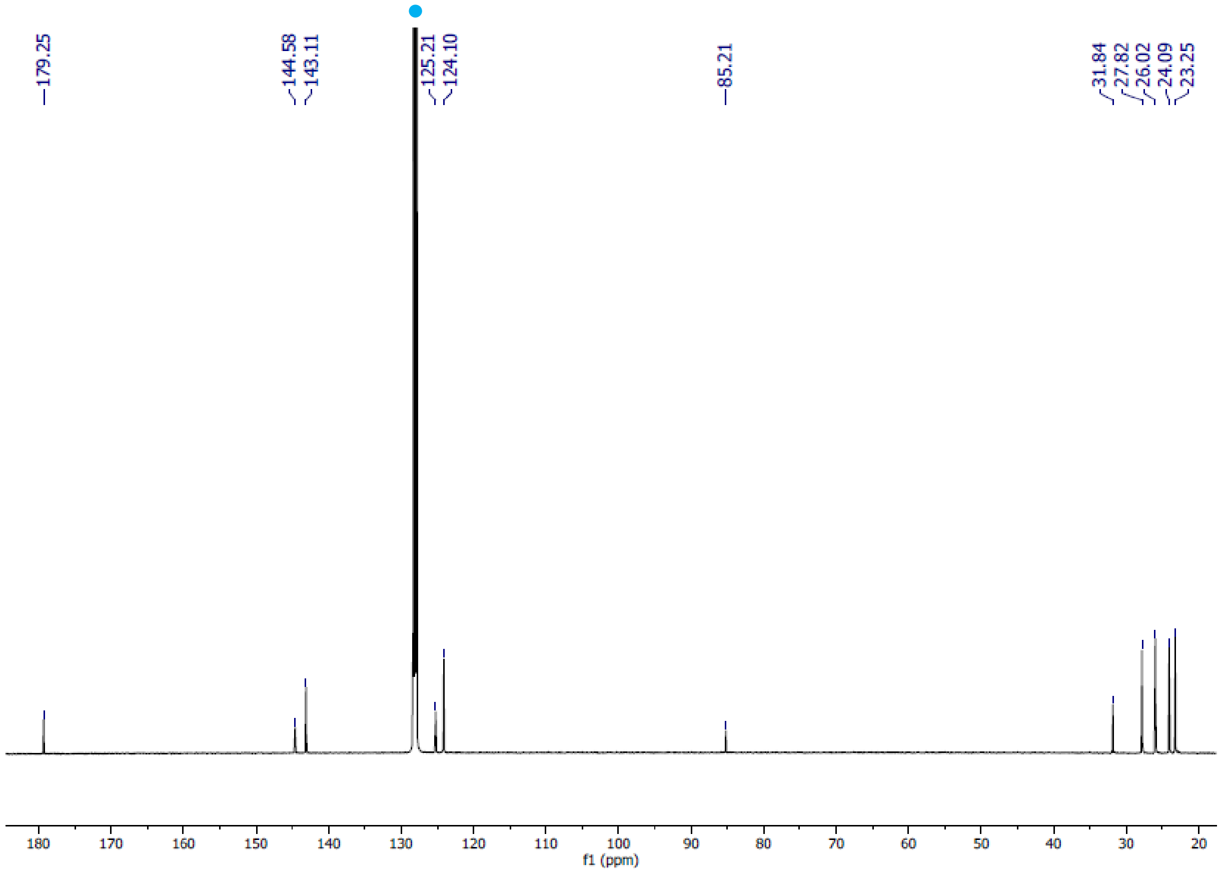

Figure S32. ^{13}C{^{1}H} NMR spectrum (100.5 MHz, C_6D_6, 298 K) of [{(^{iPrDip}nacnac)Mg(μ-H)}$_2$] **5**.

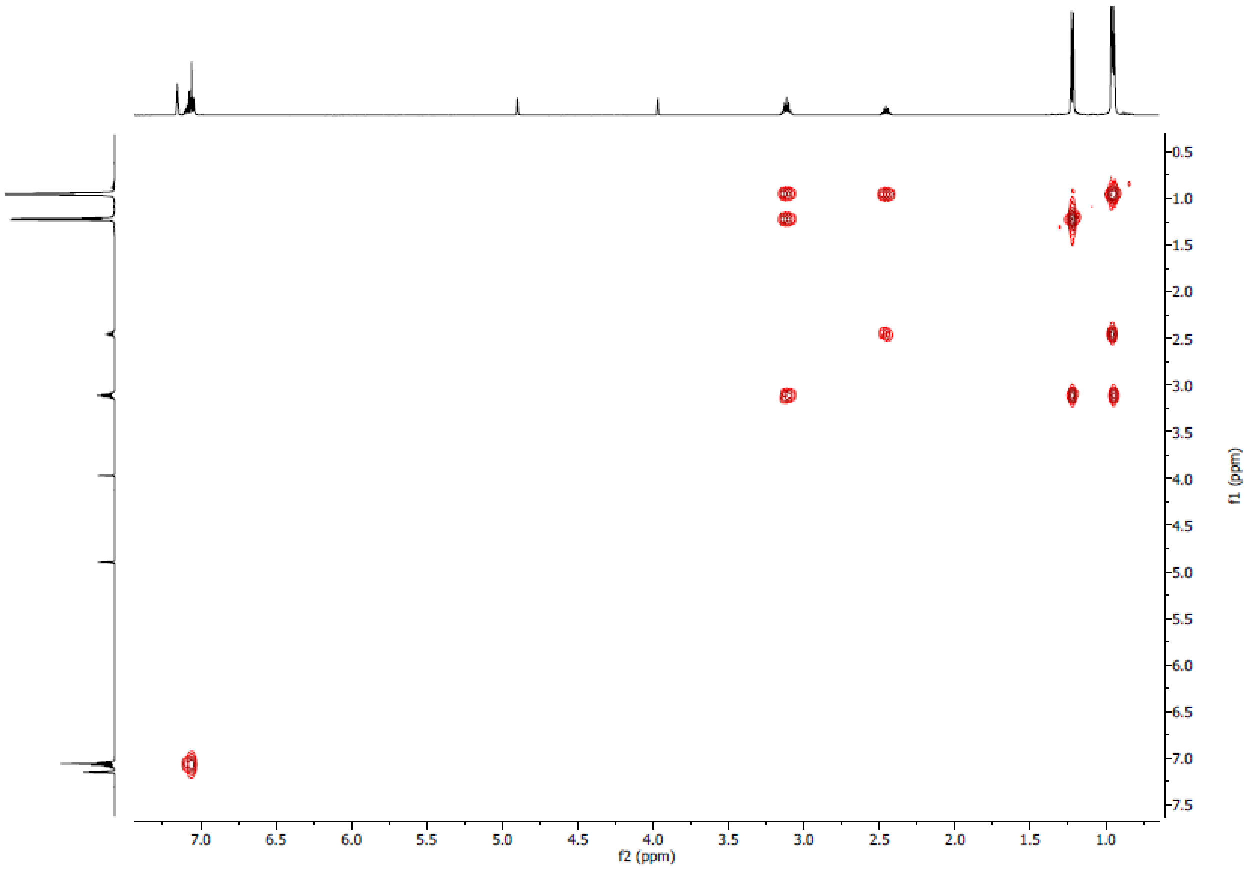

Figure S33. ^{1}H-^{1}H COSY NMR spectrum of [{(^{iPrDip}nacnac)Mg(μ-H)}$_2$] **5**.

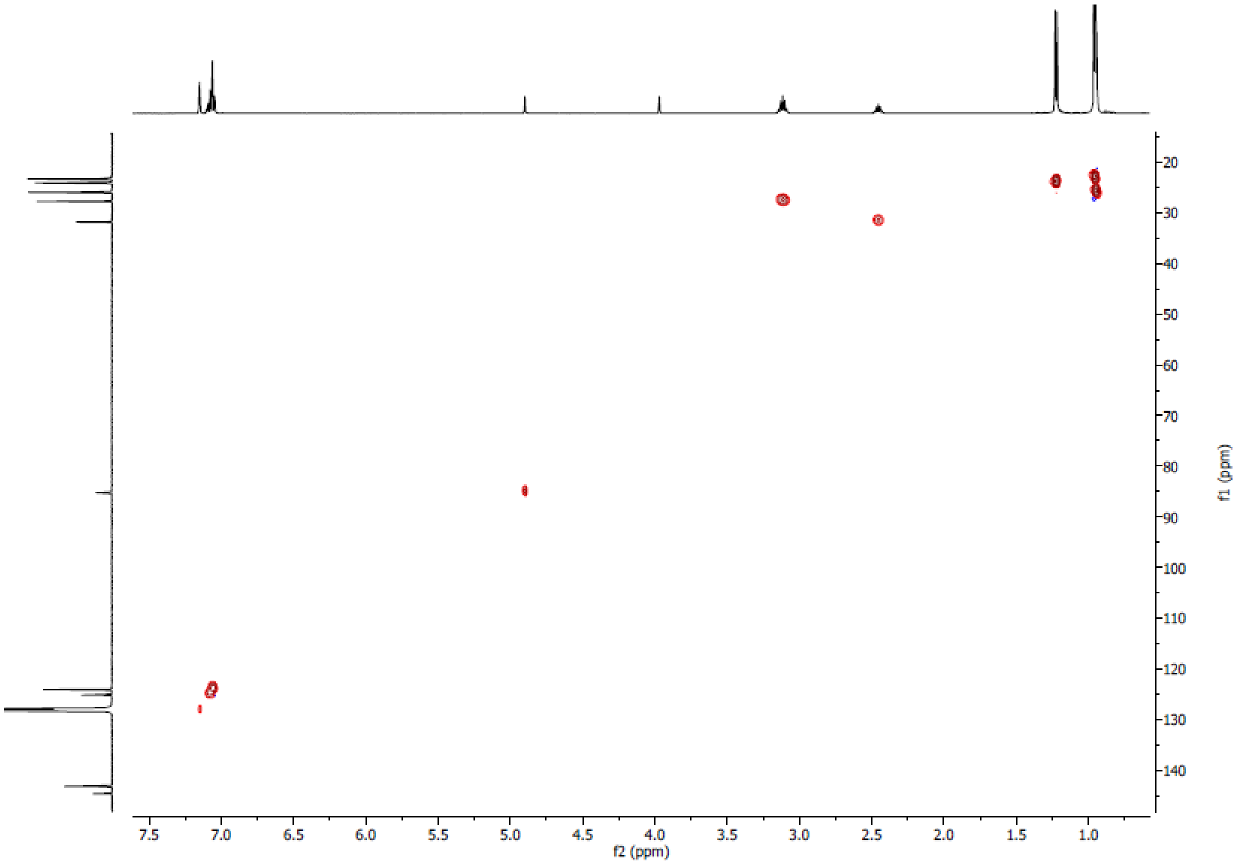

Figure S34. ^{1}H-^{13}C HSQC NMR spectrum of [{(^{iPrDip}nacnac)Mg(μ-H)}$_2$] **5**.

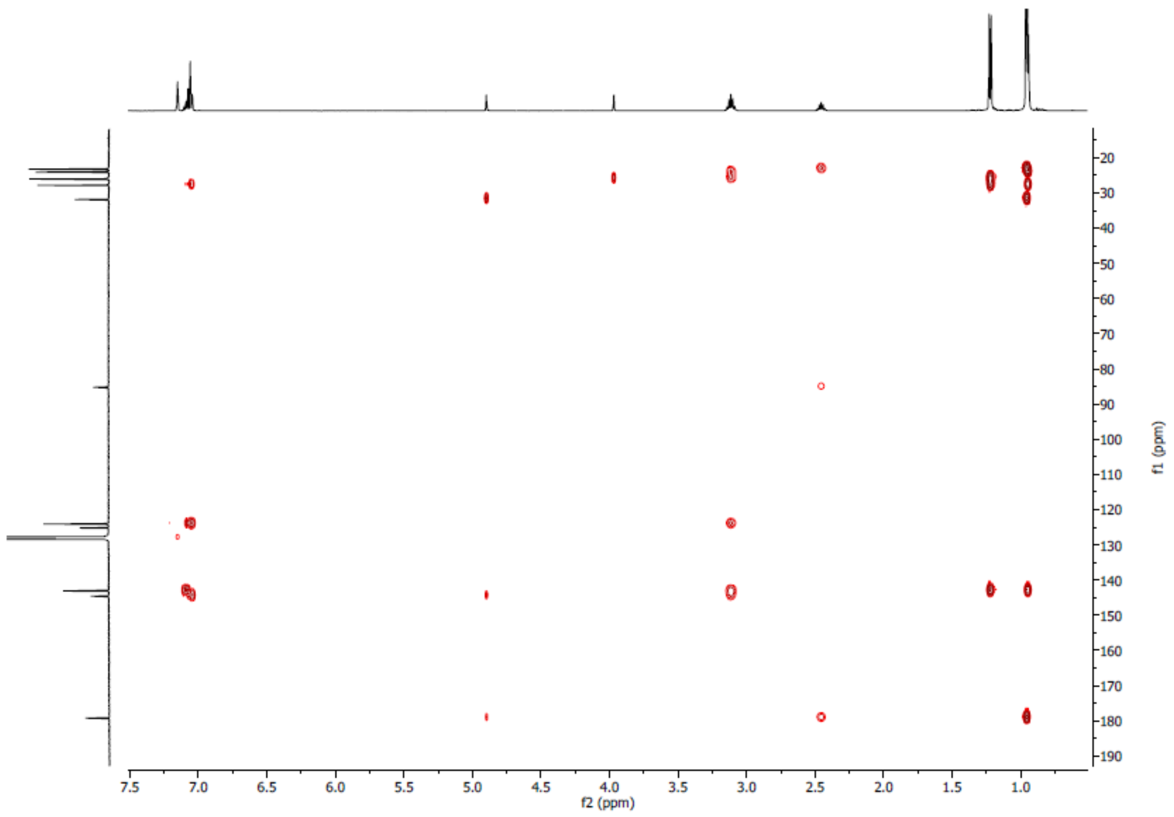

Figure S35. ^{1}H-^{13}C HMBC NMR spectrum of [{(^{iPrDip}nacnac)Mg(μ-H)}$_2$] **5**.

2.7 NMR spectra of [{(^{iPrDip}nacnac)Mg}$_2$(μ-OAd)] 6

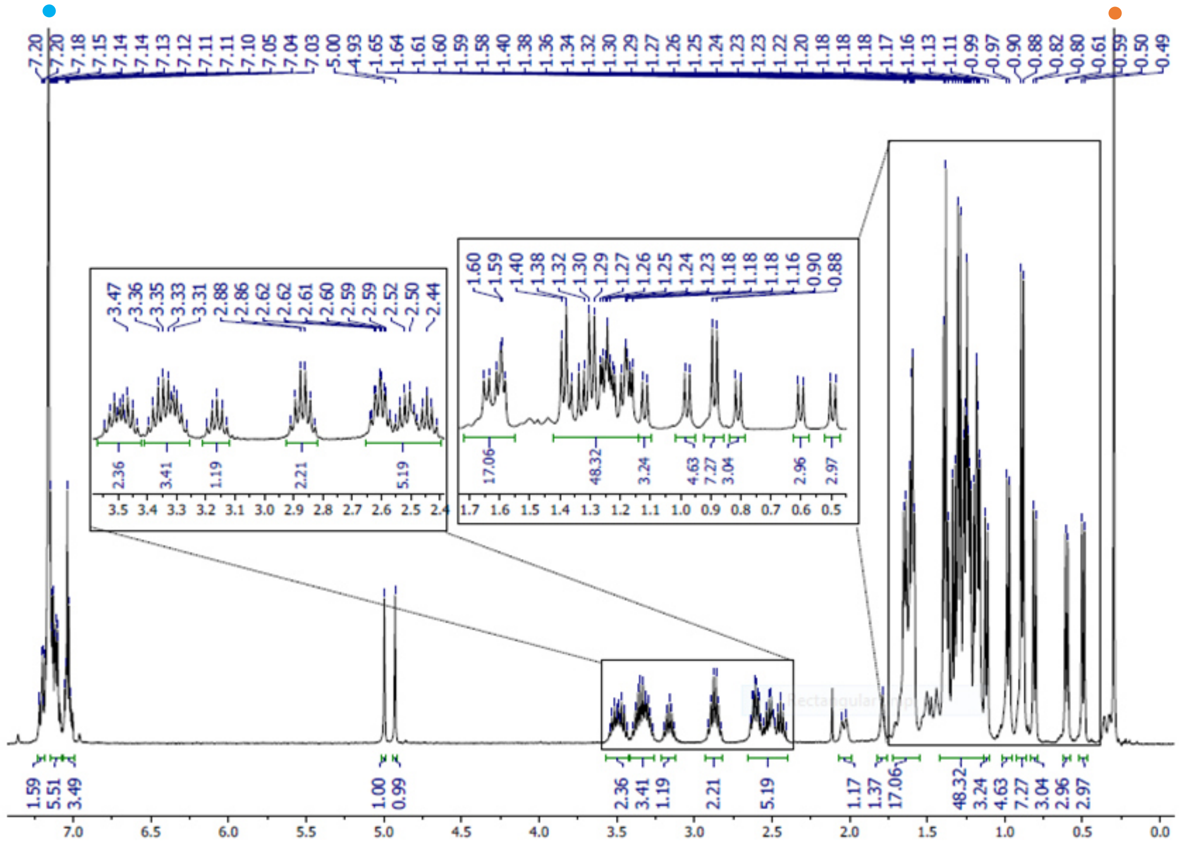

Figure S36. ^{1}H NMR spectrum (400.1 MHz, C_6D_6, 298 K) of [{(^{iPrDip}nacnac)Mg}$_2$(μ-OAd)] **6** (with aliphatic and septet region zooms).

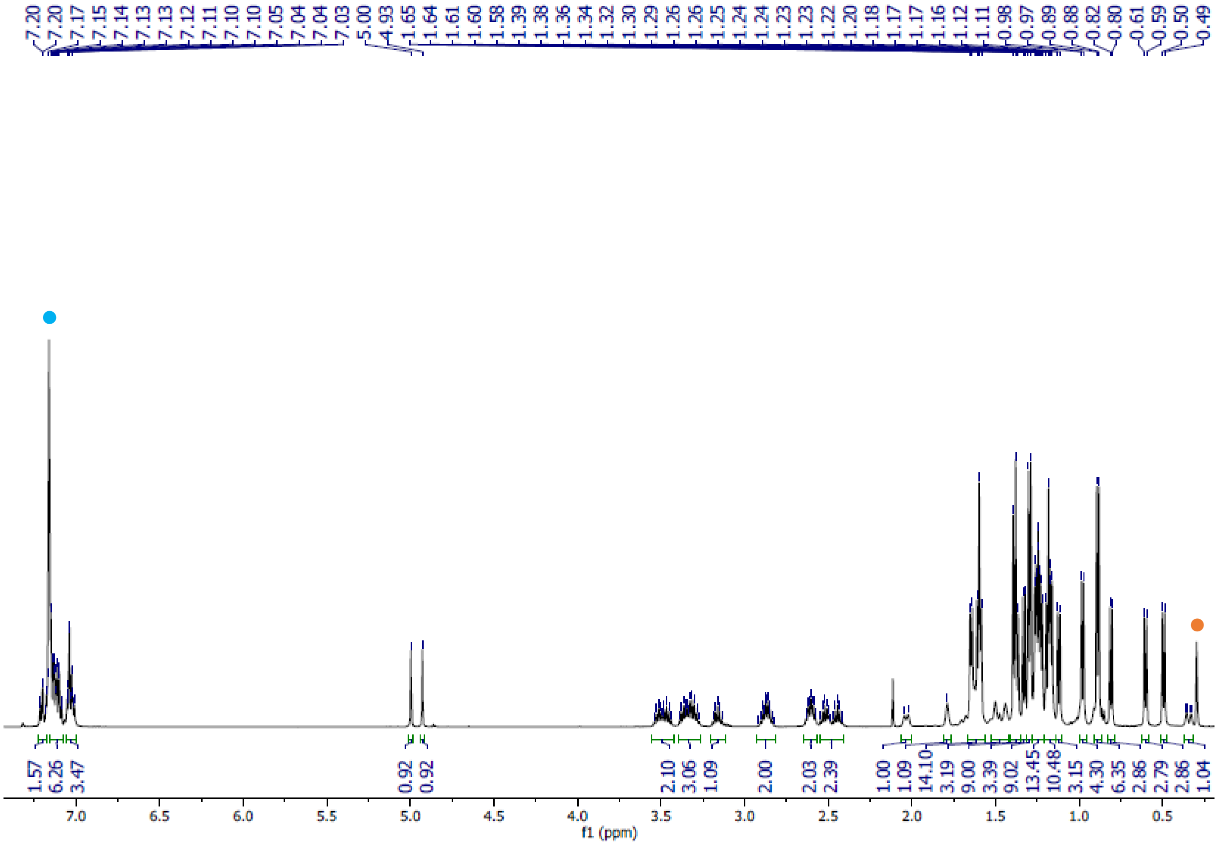

Figure S37. ^{1}H NMR spectrum (400.1 MHz, C_6D_6, 298 K) of [{(^{iPrDip}nacnac)Mg}$_2$(μ-OAd)] **6**.

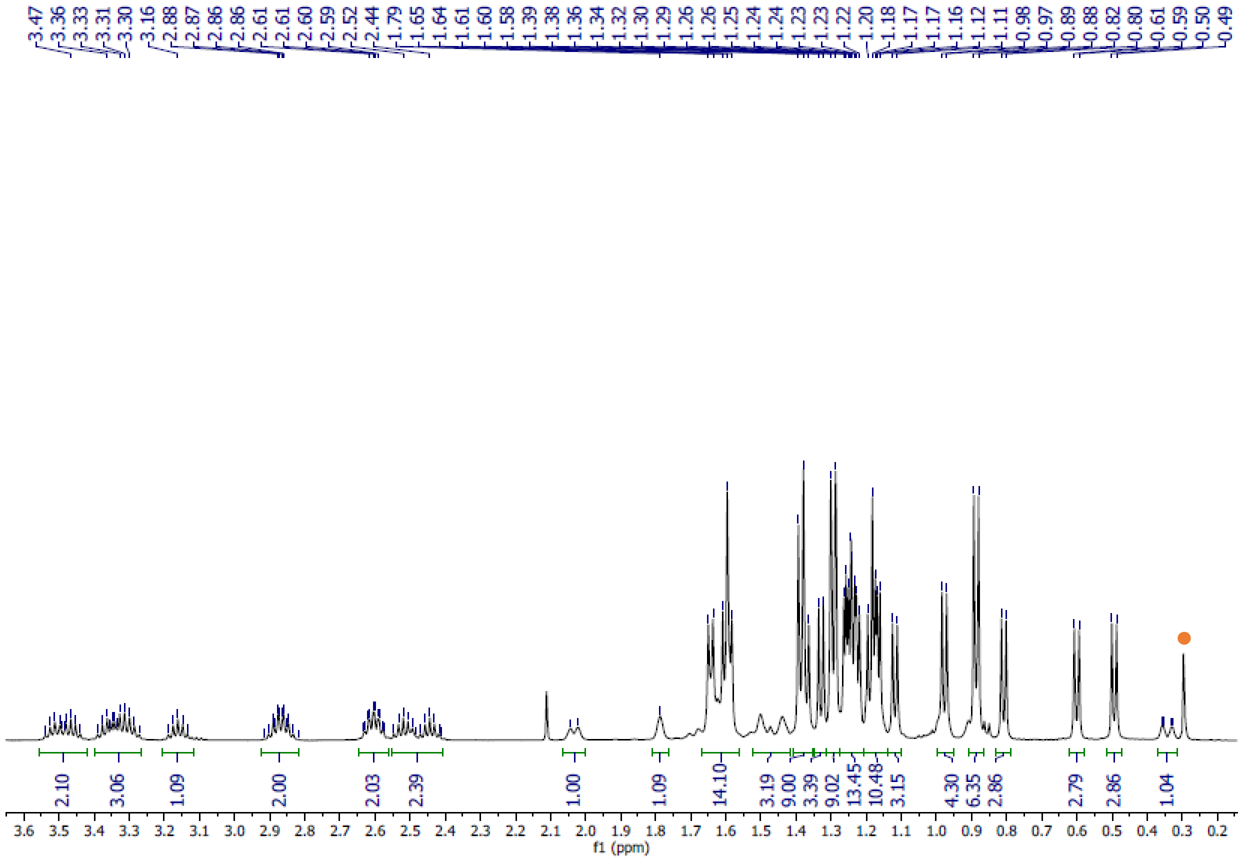

Figure S38. ^{1}H NMR spectrum (400.1 MHz, C_6D_6, 298 K) of **6** (chemical shift range 0.2-3.6 ppm)

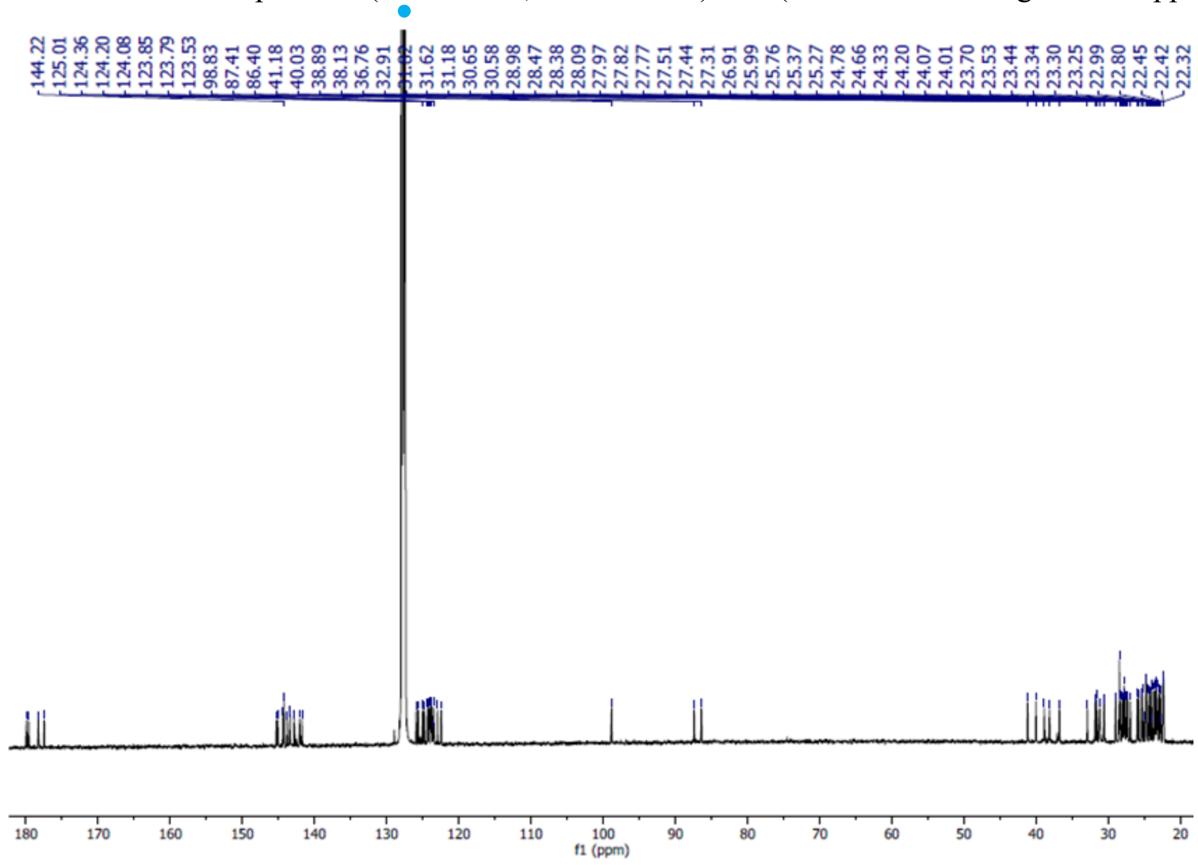

Figure S39. $^{13}C\{^{1}H\}$ NMR spectrum (100.5 MHz, C_6D_6, 298 K) of [{(^{iPrDip}nacnac)Mg}$_2$(μ-OAd)] **6**.

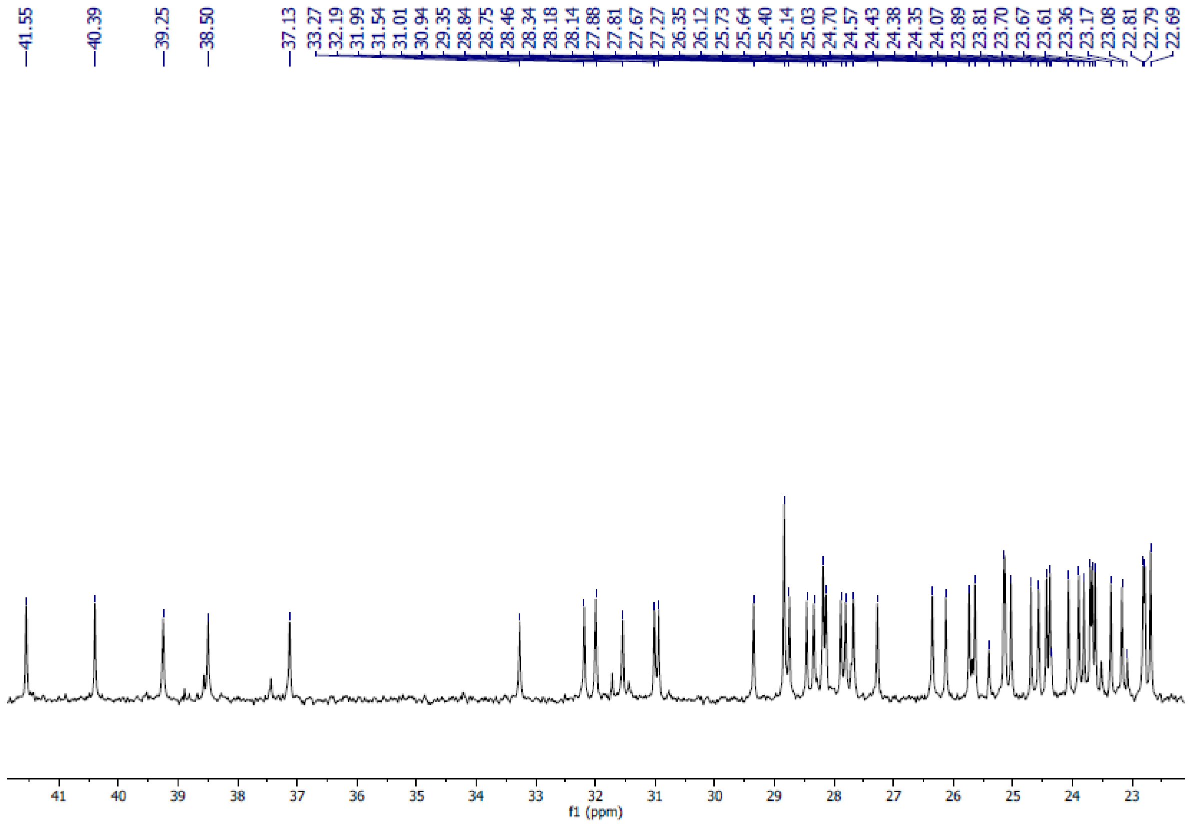

Figure S40. $^{13}C\{^1H\}$ NMR spectrum (100.5 MHz, C_6D_6, 298 K) of [{(^{iPrDip}nacnac)Mg}$_2$(μ-OAd)] **6** (chemical shift range 22-42 ppm).

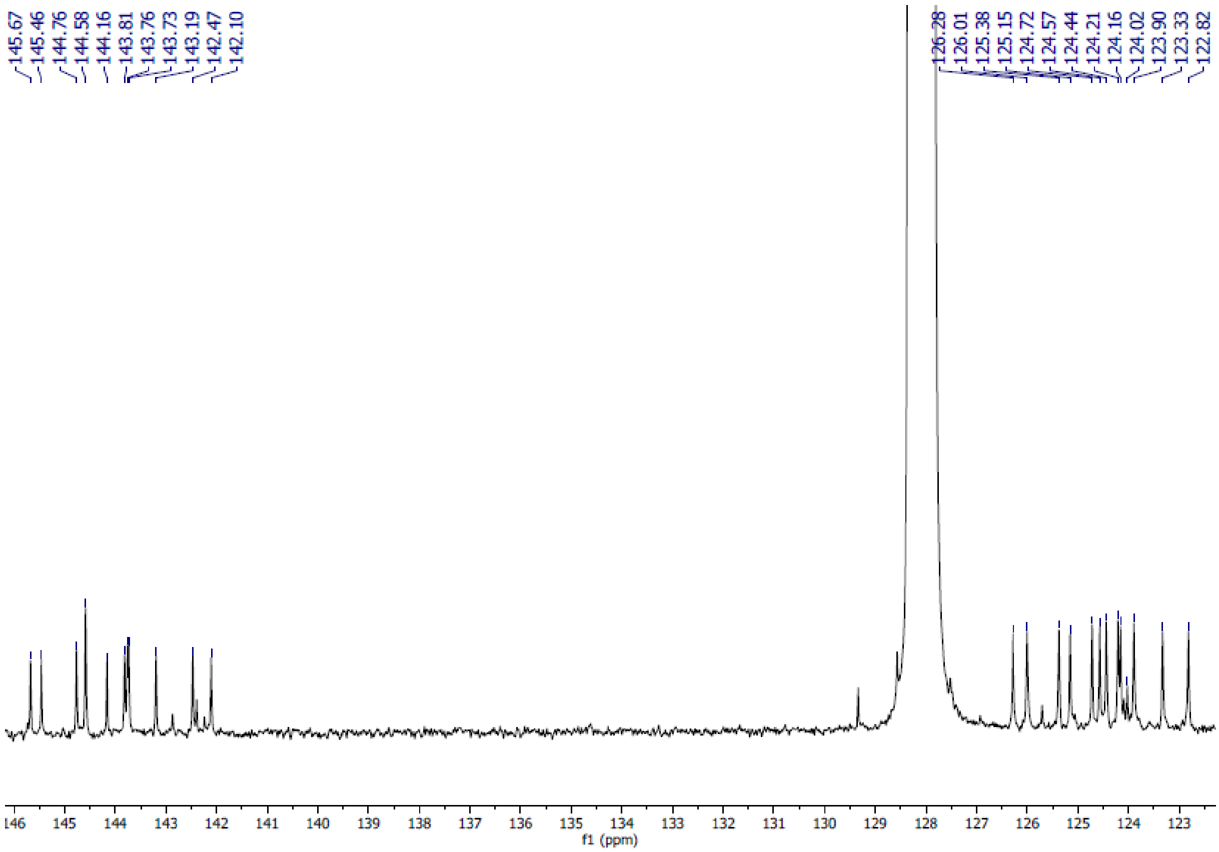

Figure S41. $^{13}C\{^1H\}$ NMR spectrum (100.5 MHz, C_6D_6, 298 K) of [{(^{iPrDip}nacnac)Mg}$_2$(μ-OAd)] **6** (chemical shift range 122-146 ppm).

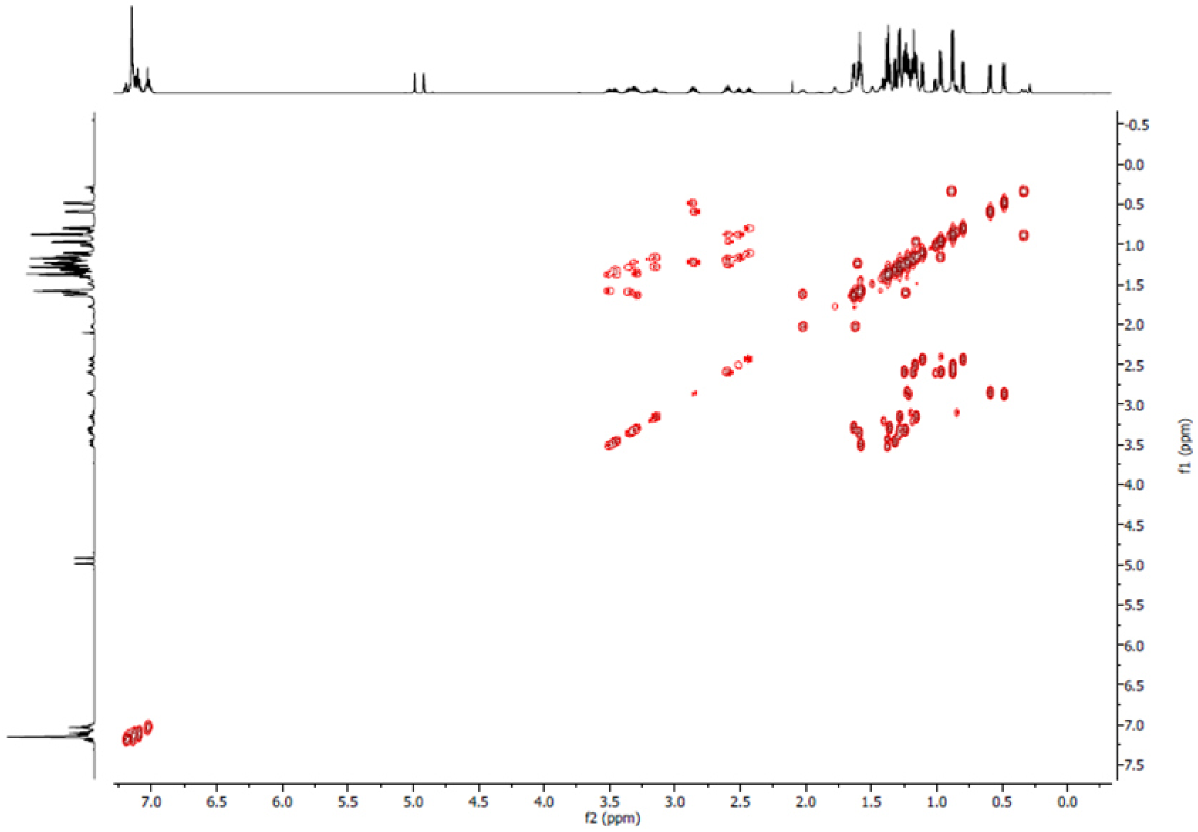

Figure S42. ^{1}H-^{1}H COSY NMR spectrum of [{(^{iPrDip}nacnac)Mg}$_2$(μ-OAd)] **6**.

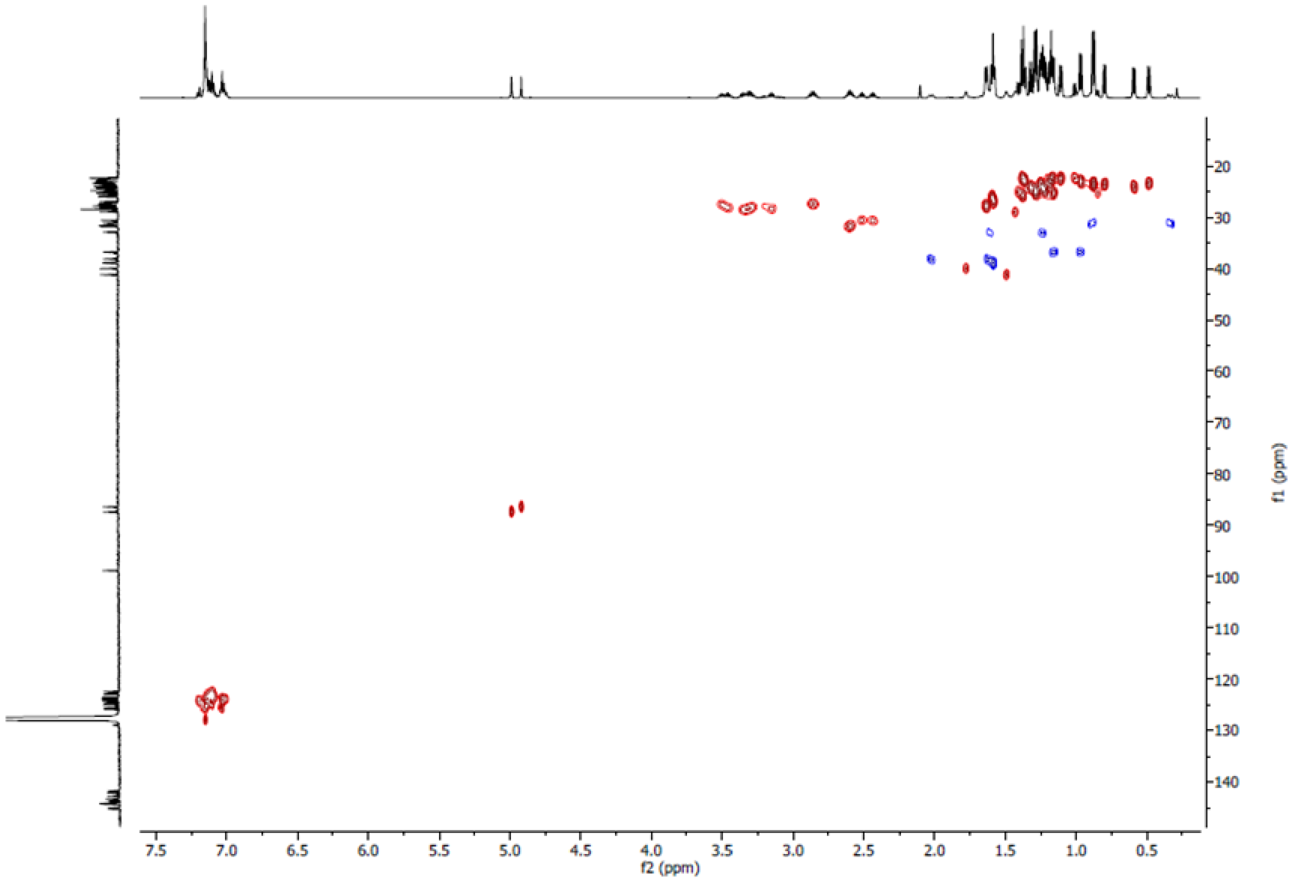

Figure S43. ^{1}H-^{13}C HSQC NMR spectrum of [{(^{iPrDip}nacnac)Mg}$_2$(μ-OAd)] **6**.

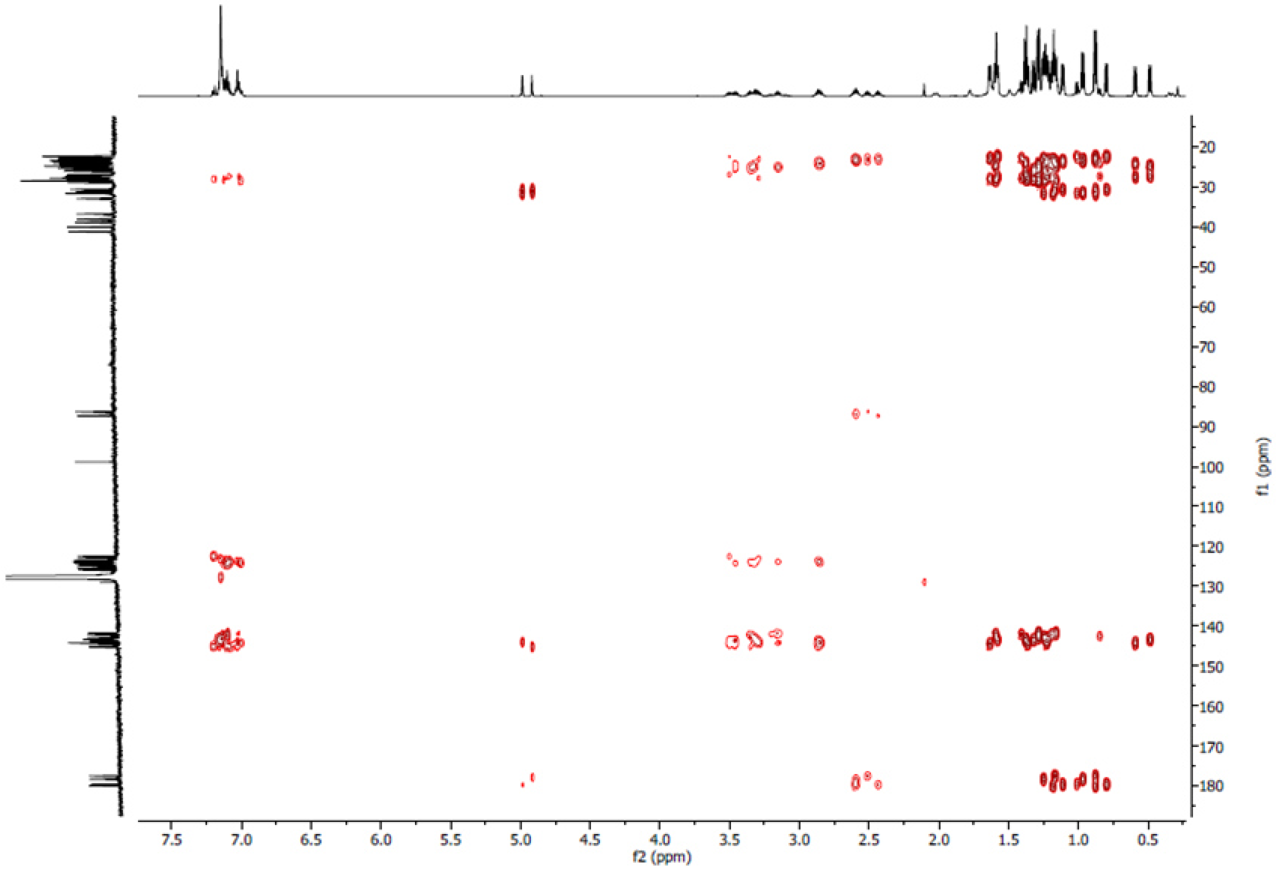

Figure S44. ^{1}H-^{13}C HMBC NMR spectrum of [{(^{iPrDip}nacnac)Mg}$_2$(μ-OAd)] **6**.

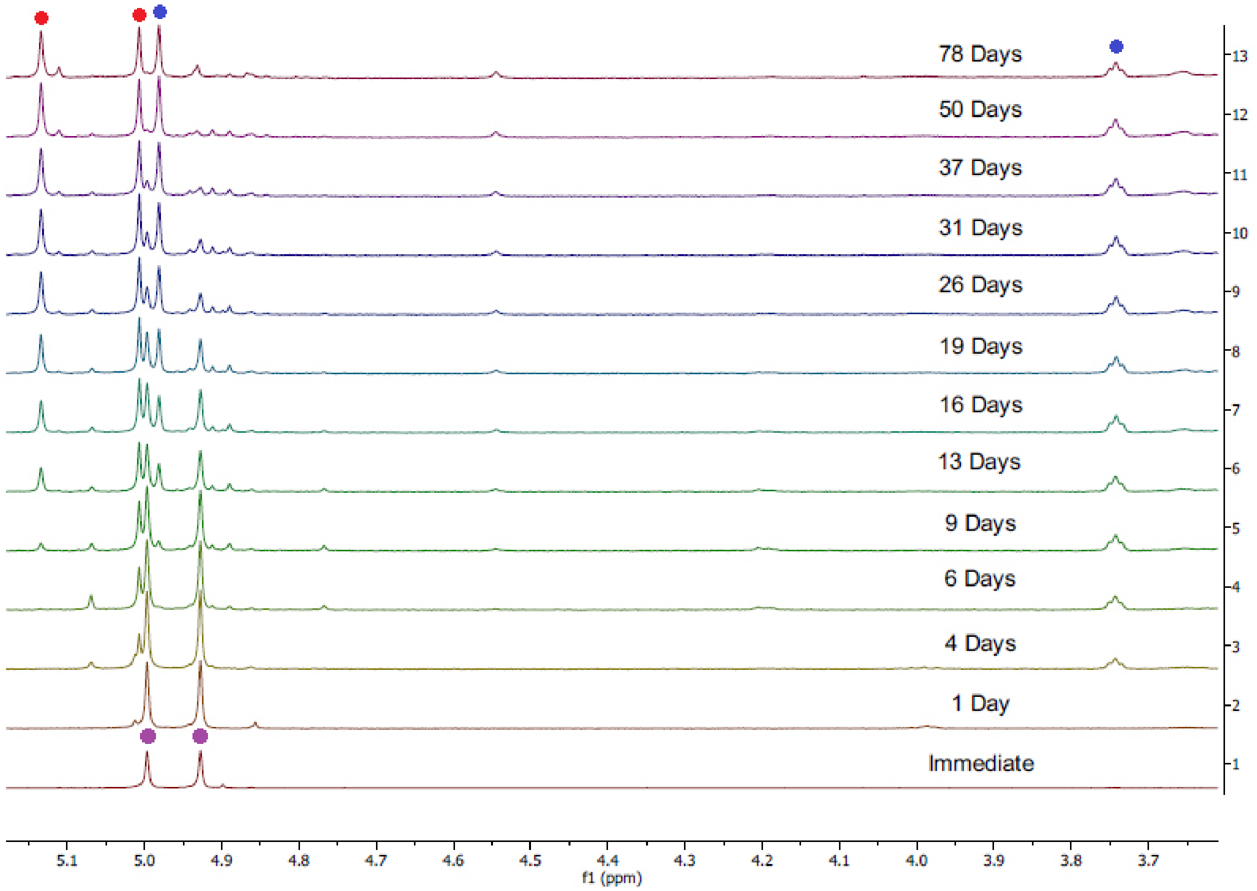

Figure S45. ^{1}H NMR spectroscopic time plots monitoring the room temperature decomposition of [{(^{iPrDip}nacnac)Mg}$_2$(μ-OAd)] **6** (chemical shift range 3.6-5.2 ppm). Blue dots: [(^{iPrDip}nacnac)Mg(OAdH)] **8**; purple dots: [{(^{iPrDip}nacnac)Mg}$_2$(μ-OAd)] **6**; red dots: unknown asymmetric NacNac compound, likely a CH activation product.

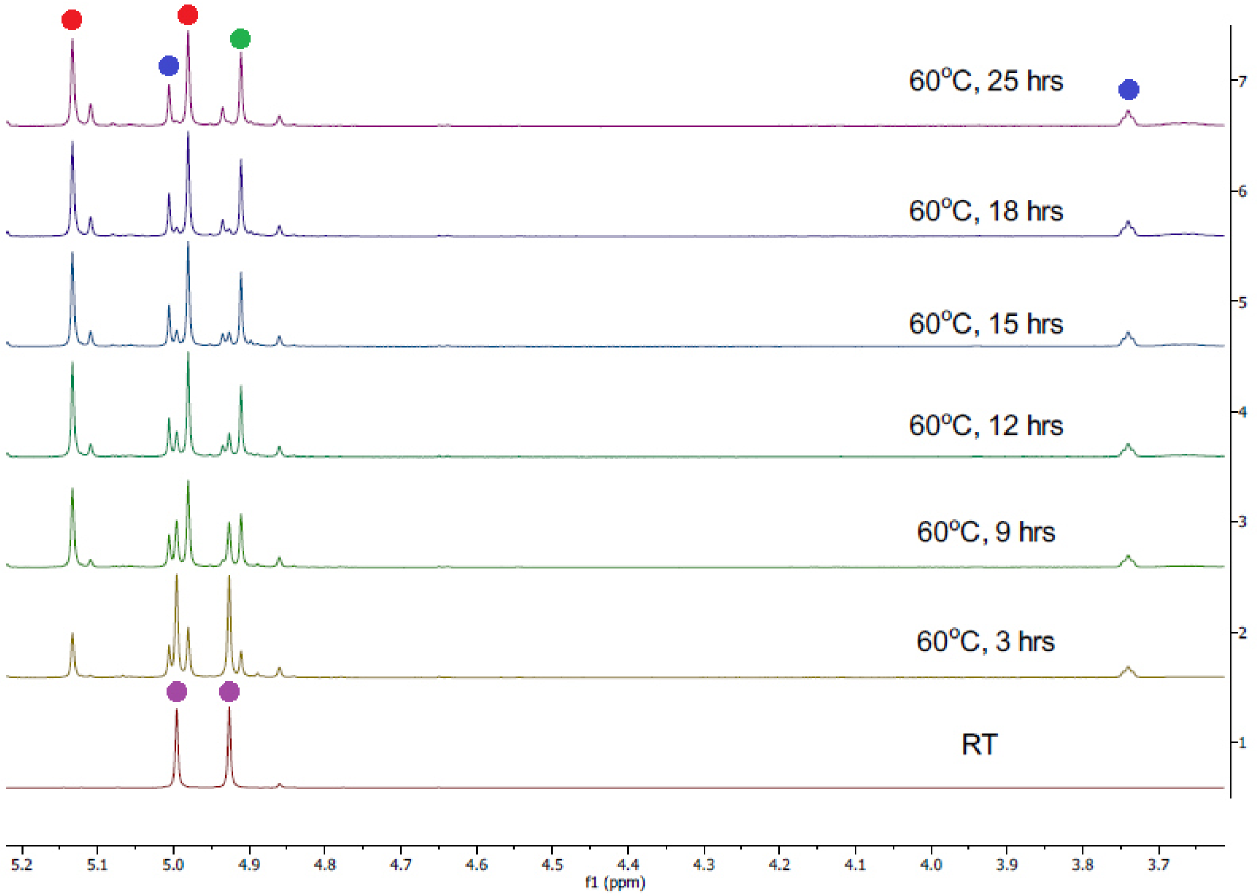

Figure S46. ^{1}H NMR spectroscopic time plots monitoring the decomposition of [{($^{i\text{PrDip}}$nacnac)Mg}$_2$(μ-OAd)] **6** at 60 °C (chemical shift range 3.6-5.2 ppm). Blue dots: [($^{i\text{PrDip}}$nacnac)Mg(OAdH)] **8**; purple dots: [{($^{i\text{PrDip}}$nacnac)Mg}$_2$(μ-OAd)] **6**; green dot: [{($^{i\text{PrDip}}$nacnac)Mg}$_2$(μ-O)] **7**; red dots: unknown asymmetric NacNac compound, likely a CH activation product. After 25 hours at 60°C, the main decomposition product is the asymmetric species (red dots). The ratio of **7** to **8** is approximately 1:2, and no starting material **6** is remaining.

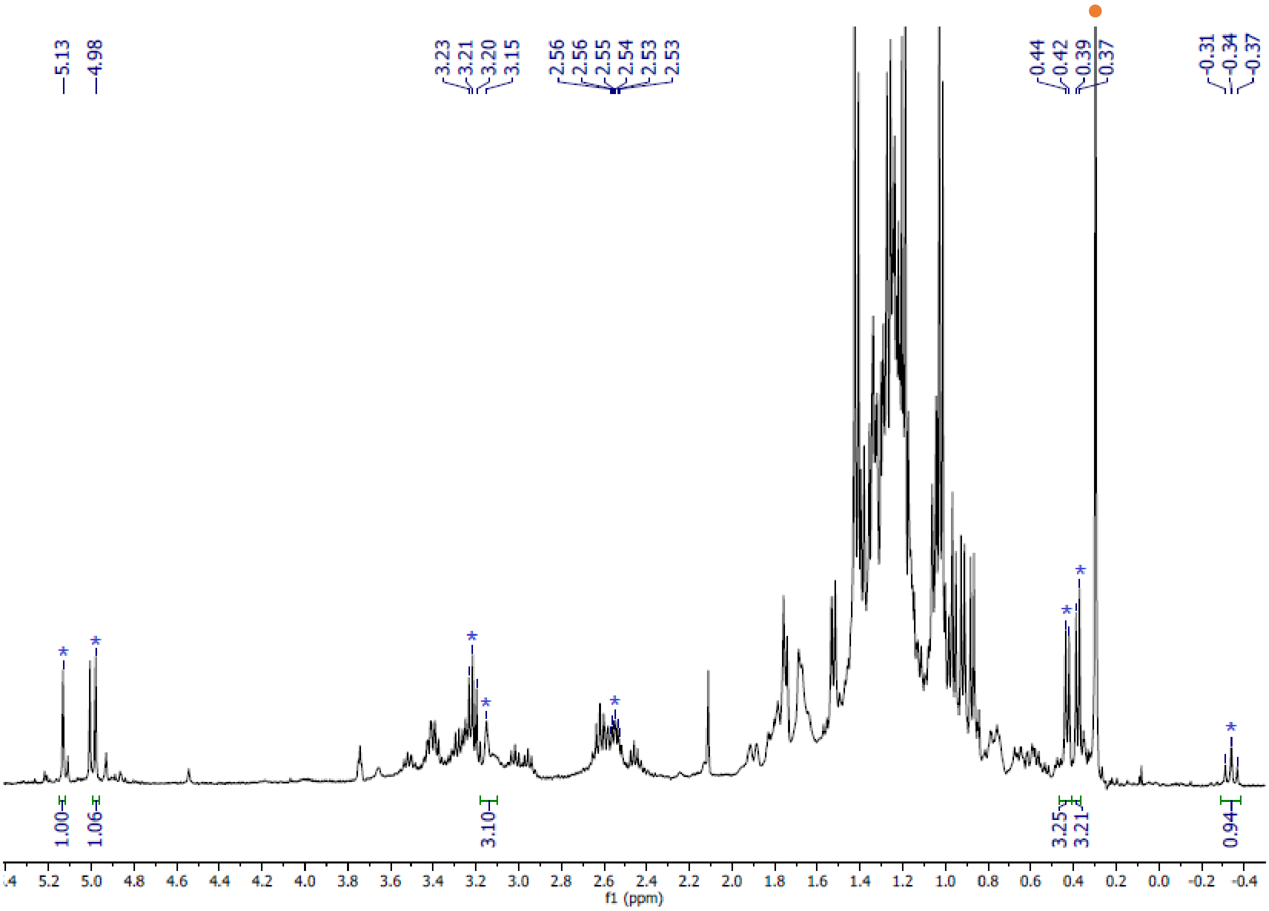

Figure S47. ^{1}H NMR spectrum (400.1 MHz, C_6D_6, 298 K) of room temperature decomposition of [{(^{iPrDip}nacnac)Mg}$_2$(μ-OAd)] **6** after 78 days (chemical shift range -0.5-5.4 ppm). Asterisks denote resonances assigned to proposed CH activation product. Only select integrals provided due to overlapping resonances from additional decomposition products.

2.8 NMR spectra of [{(^{iPrDip}nacnac)Mg}$_2$(μ-O)] 7

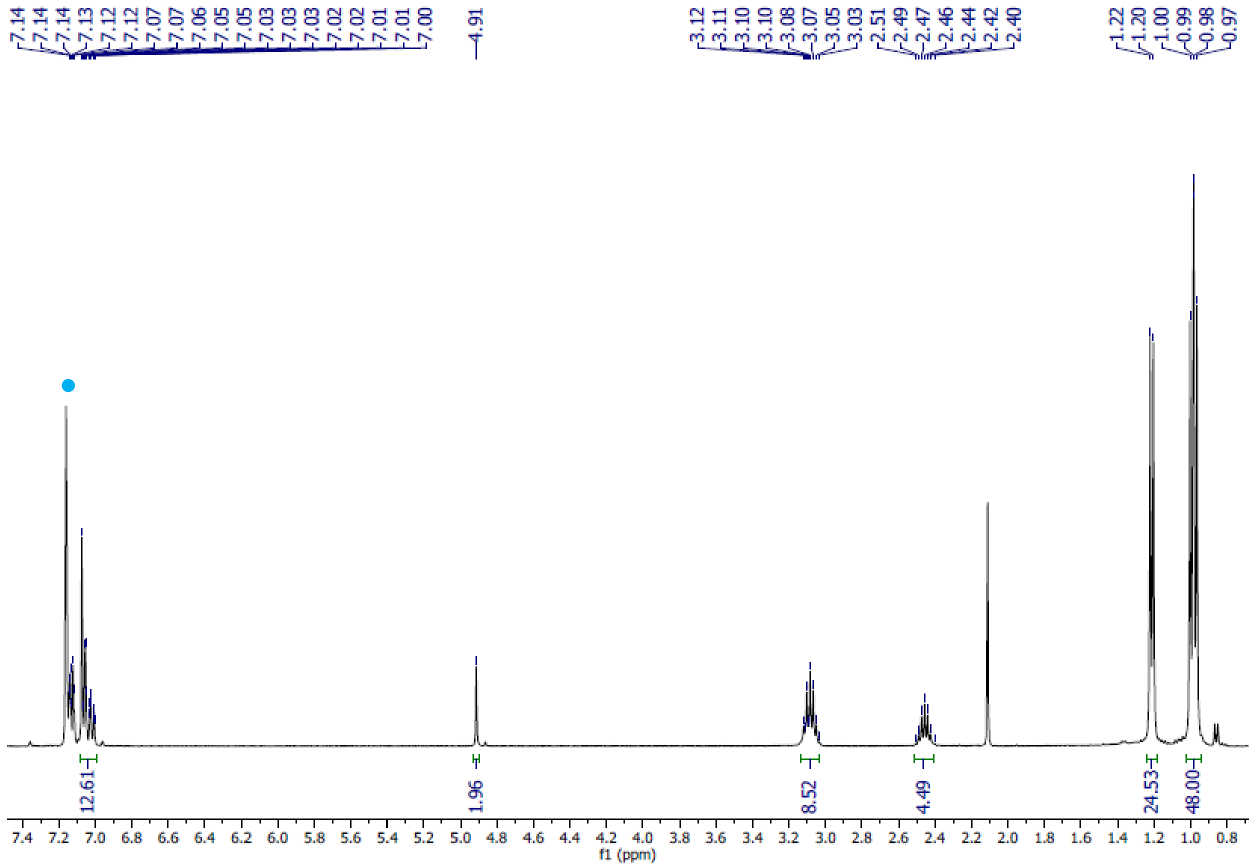

Figure S48. ^{1}H NMR spectrum (400.1 MHz, C_6D_6, 298 K) of [{(^{iPrDip}nacnac)Mg}$_2$(μ-O)] **7**.

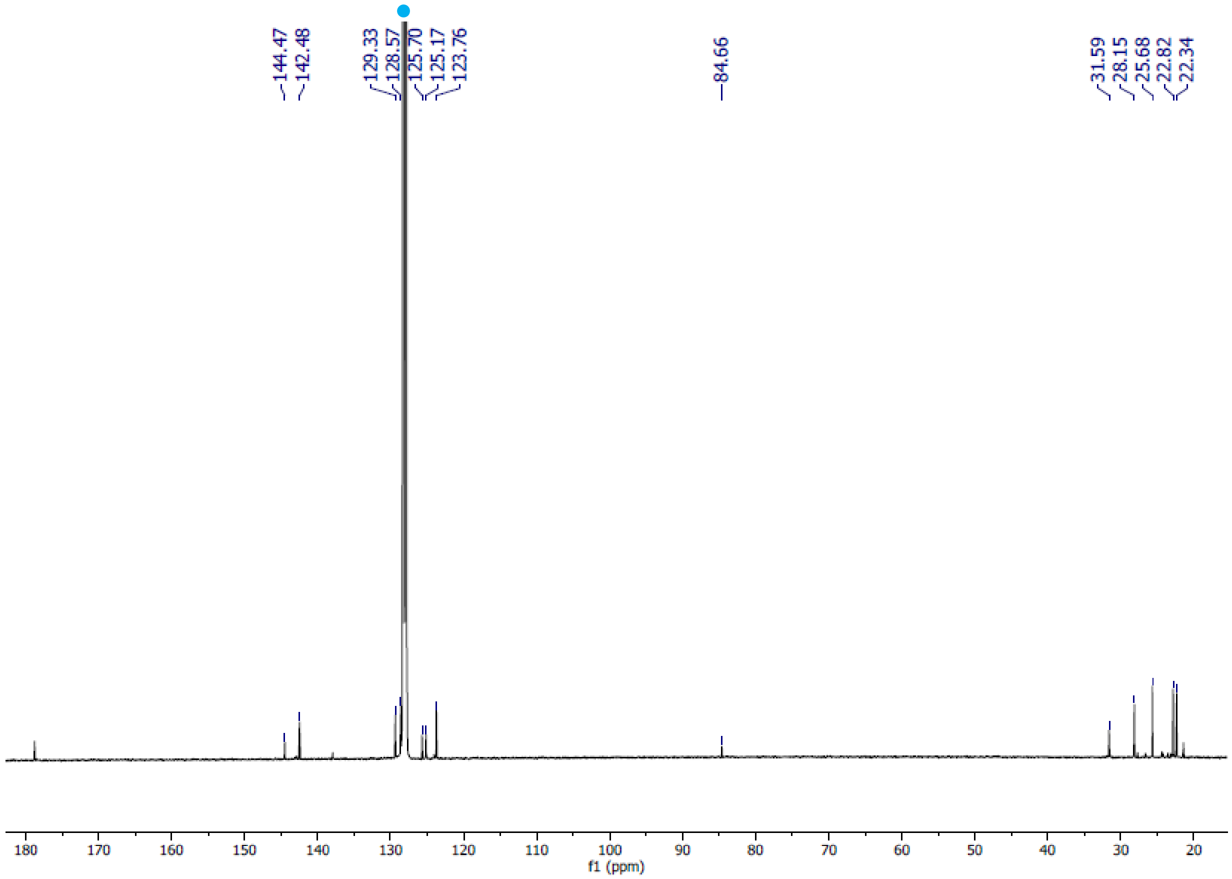

Figure S49. $^{13}C\{^1H\}$ NMR spectrum (100.5 MHz, C_6D_6, 298 K) of [{(^{iPrDip}nacnac)Mg}$_2$(μ-O)] **7**.

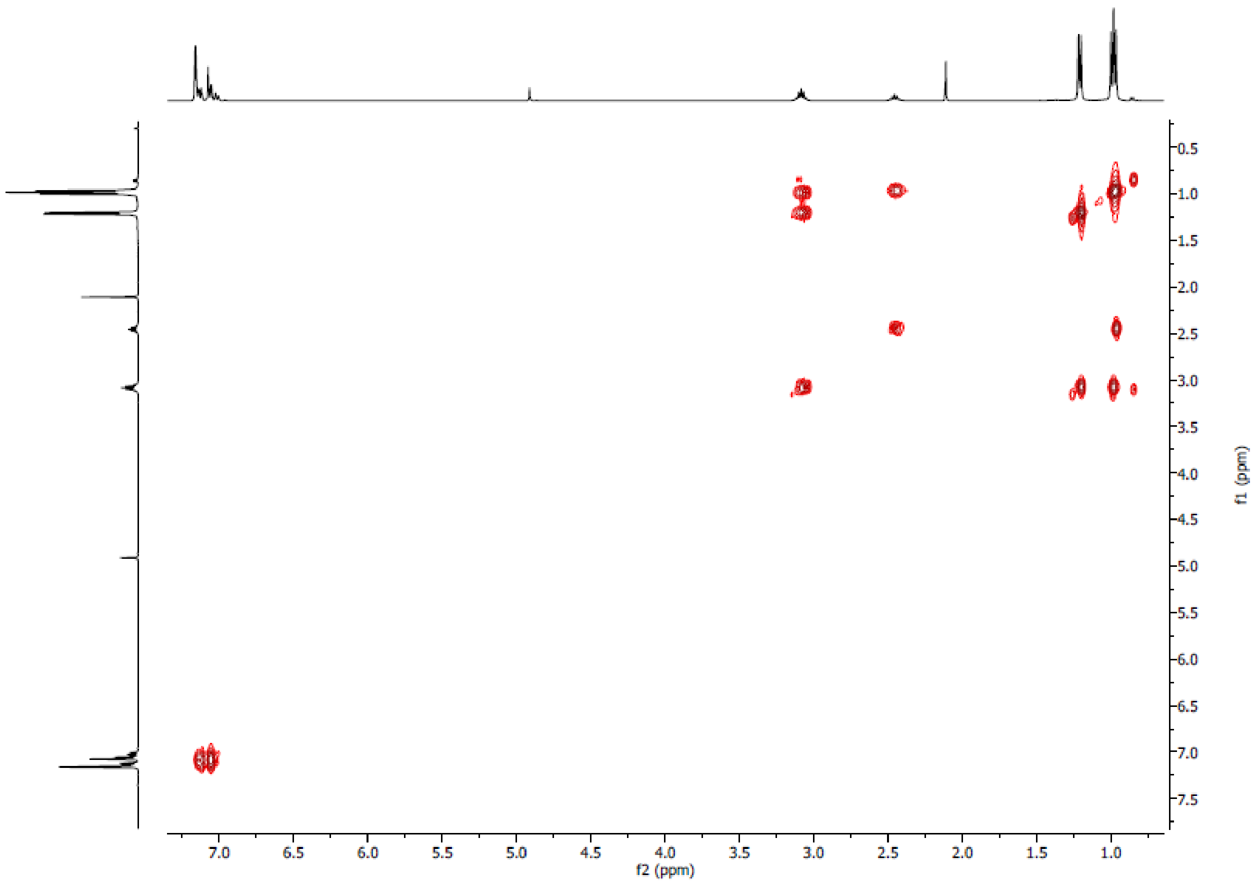

Figure S50. ^{1}H-^{1}H COSY NMR spectrum of [{(^{iPrDip}nacnac)Mg}$_2$(μ-O)] **7**.

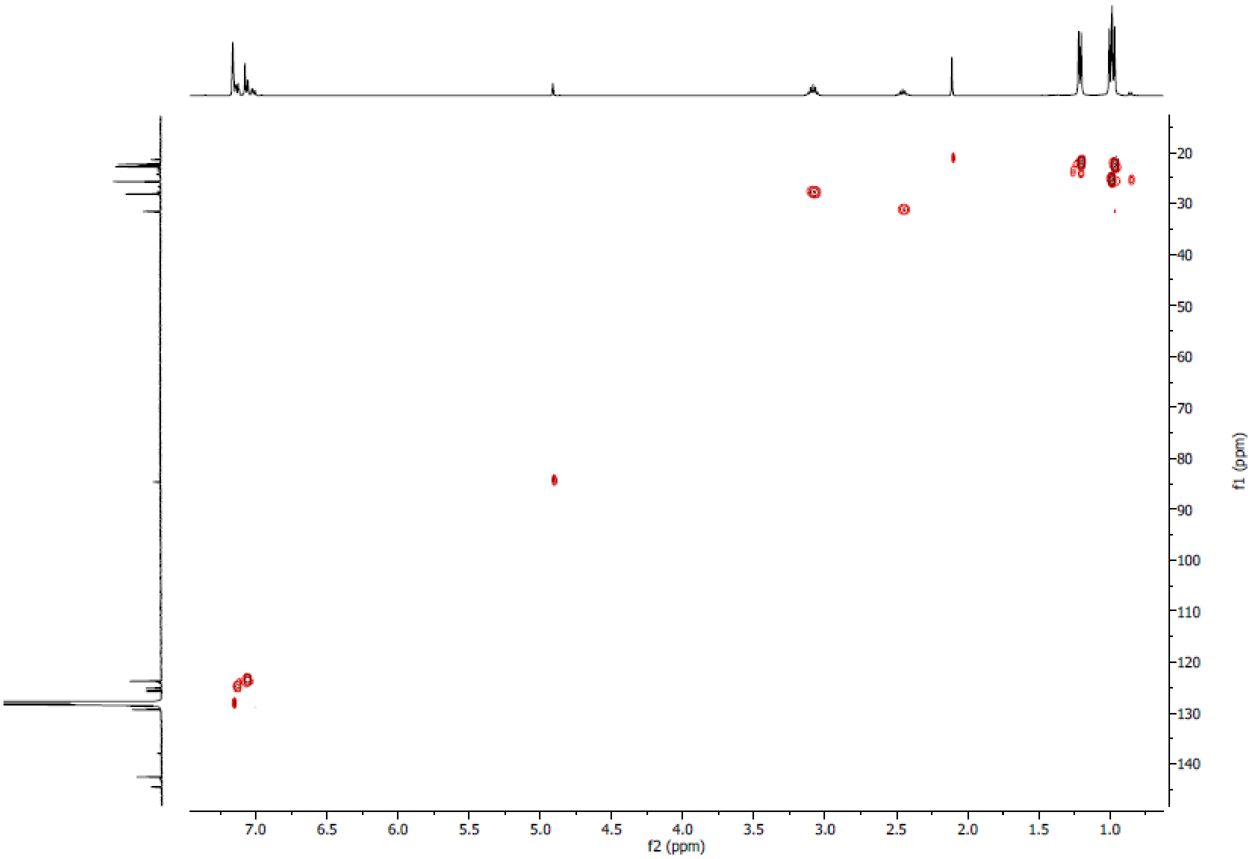

Figure S49. ^{1}H-^{13}C HSQC NMR spectrum of [{(^{iPrDip}nacnac)Mg}$_2$(μ-O)] **7**.

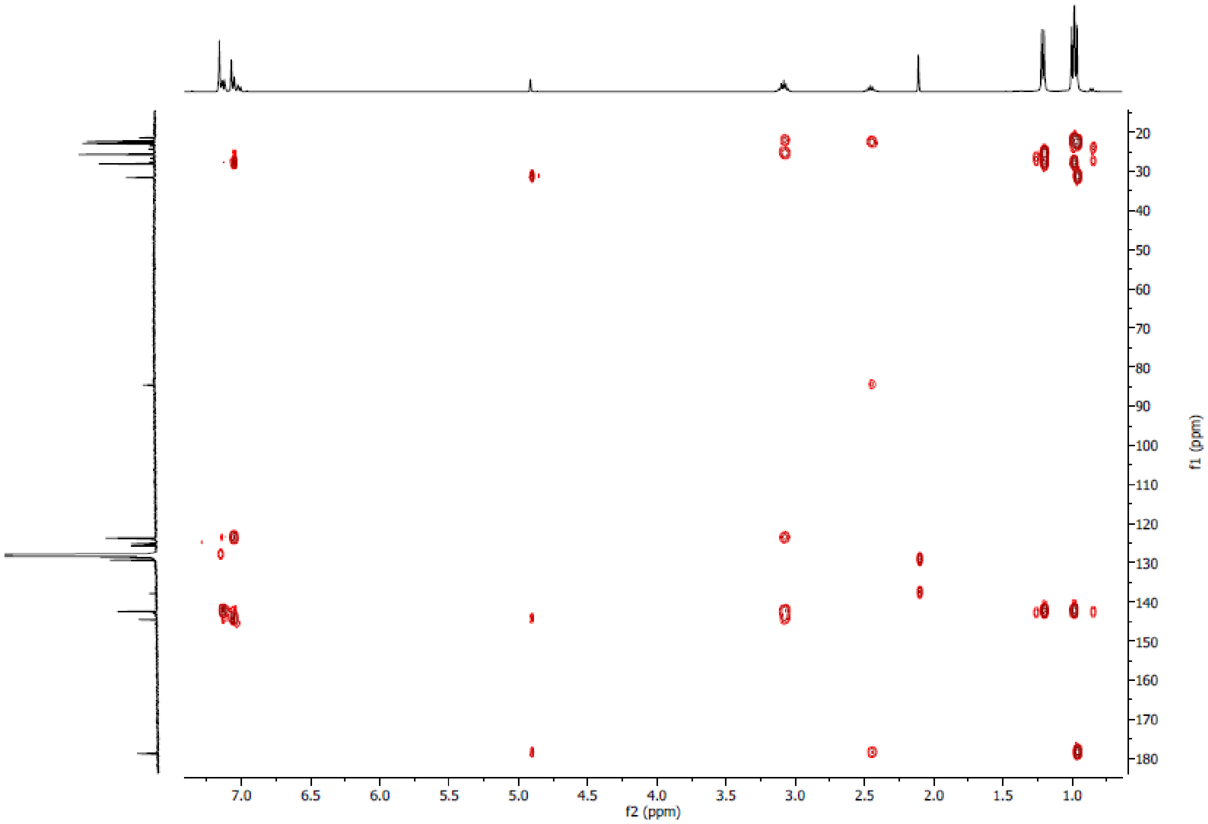

Figure S50. ^{1}H-^{13}C HMBC NMR spectrum of [{(^{iPrDip}nacnac)Mg}$_2$(μ-O)] **7**.

2.9 NMR spectra of [{(^{iPrDip}nacnac)Mg(THF)}$_2$(μ-O)]

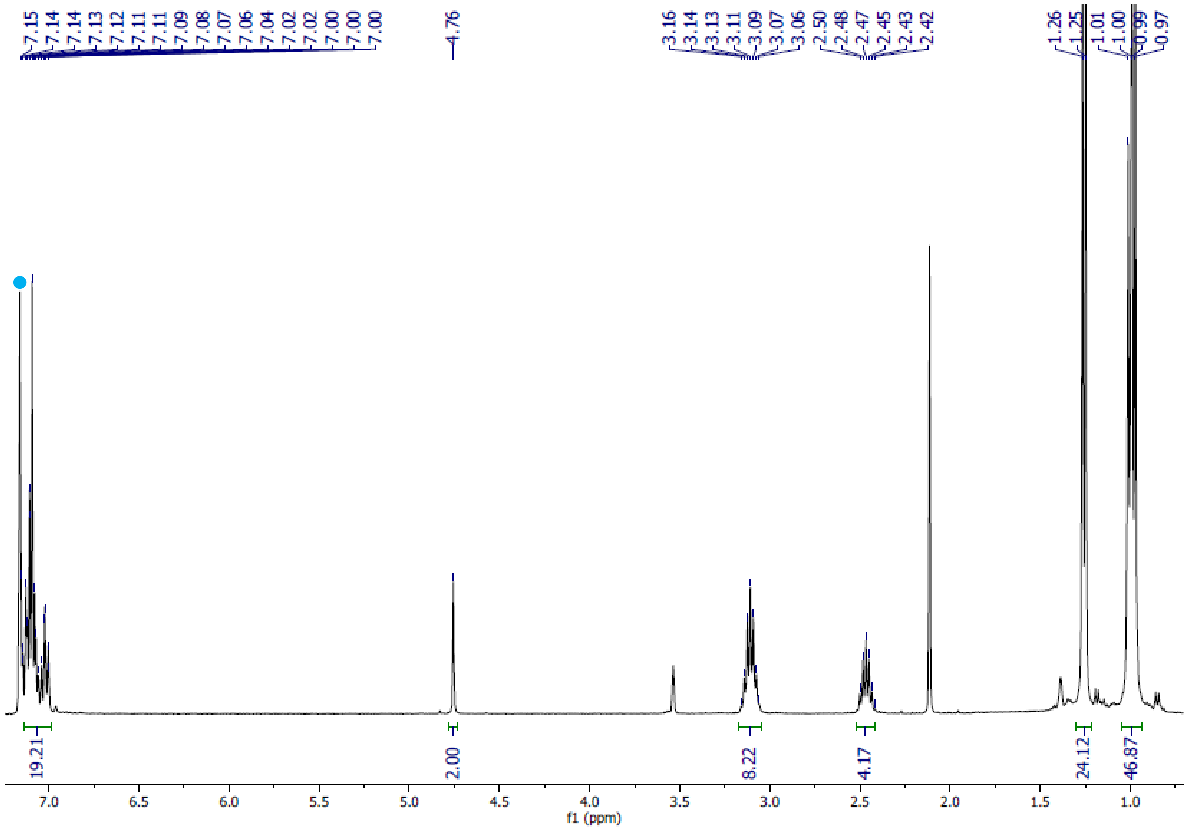

Figure S51. ^{1}H NMR spectrum (400.1 MHz, C_6D_6, 298 K) of [{(^{iPrDip}nacnac)Mg(THF)}$_2$(μ-O)].

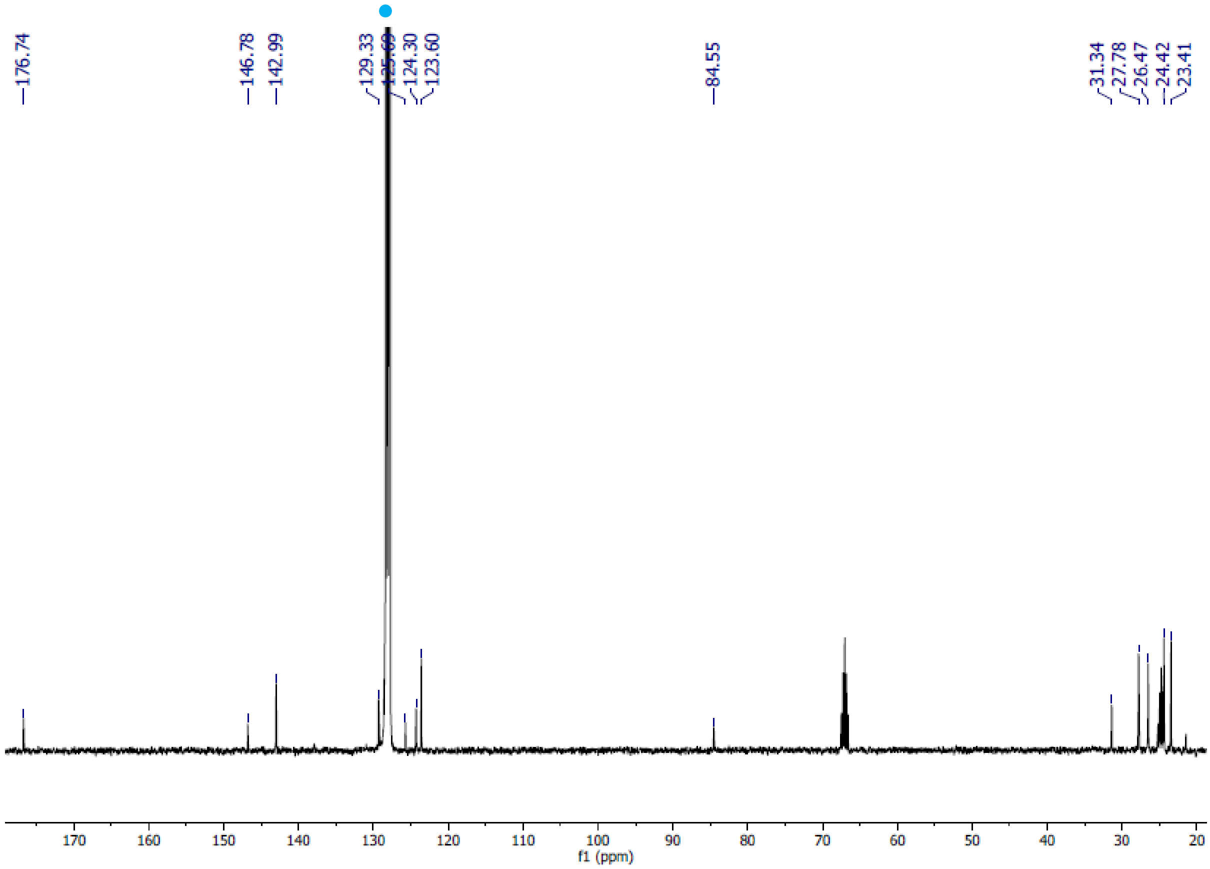

Figure S52. ^{13}C{^{1}H} NMR spectrum (100.5 MHz, C_6D_6, 298 K) of [{(^{iPrDip}nacnac)Mg(THF)}$_2$(μ-O)].

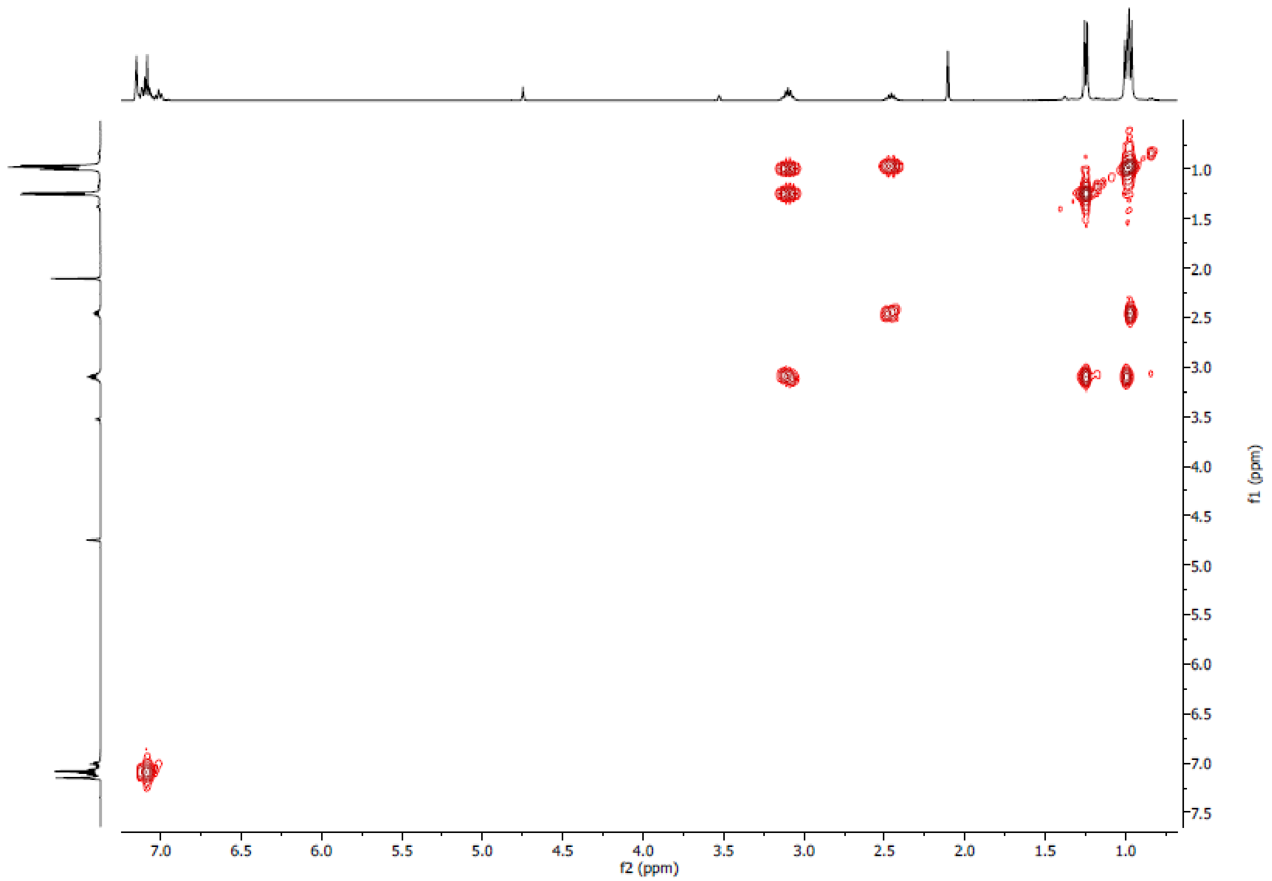

Figure S53. ^{1}H-^{1}H COSY NMR spectrum of [{(^{iPrDip}nacnac)Mg(THF)}$_2$(μ-O)].

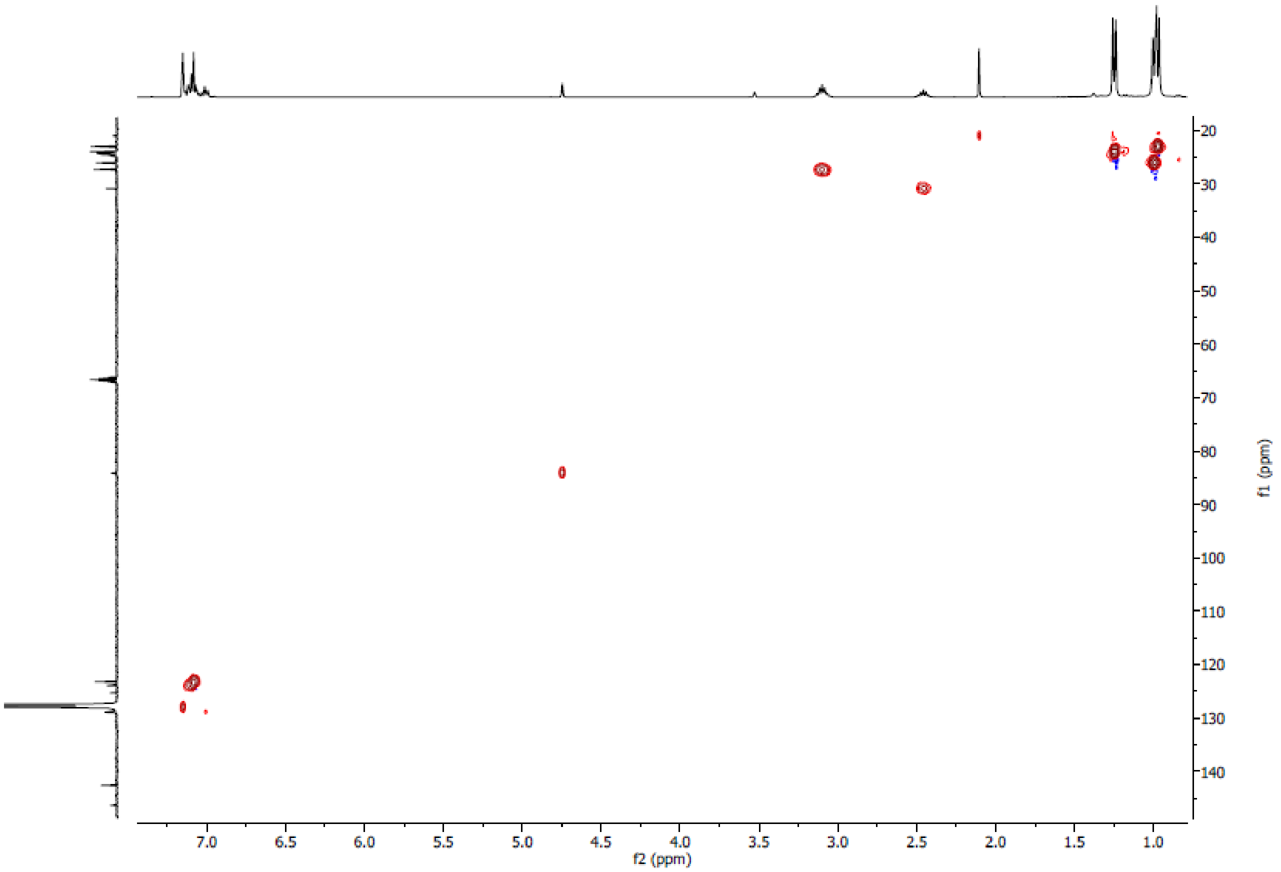

Figure S54. ^{1}H-^{13}C HSQC NMR spectrum of [{(^{iPrDip}nacnac)Mg(THF)}$_2$(μ-O)].

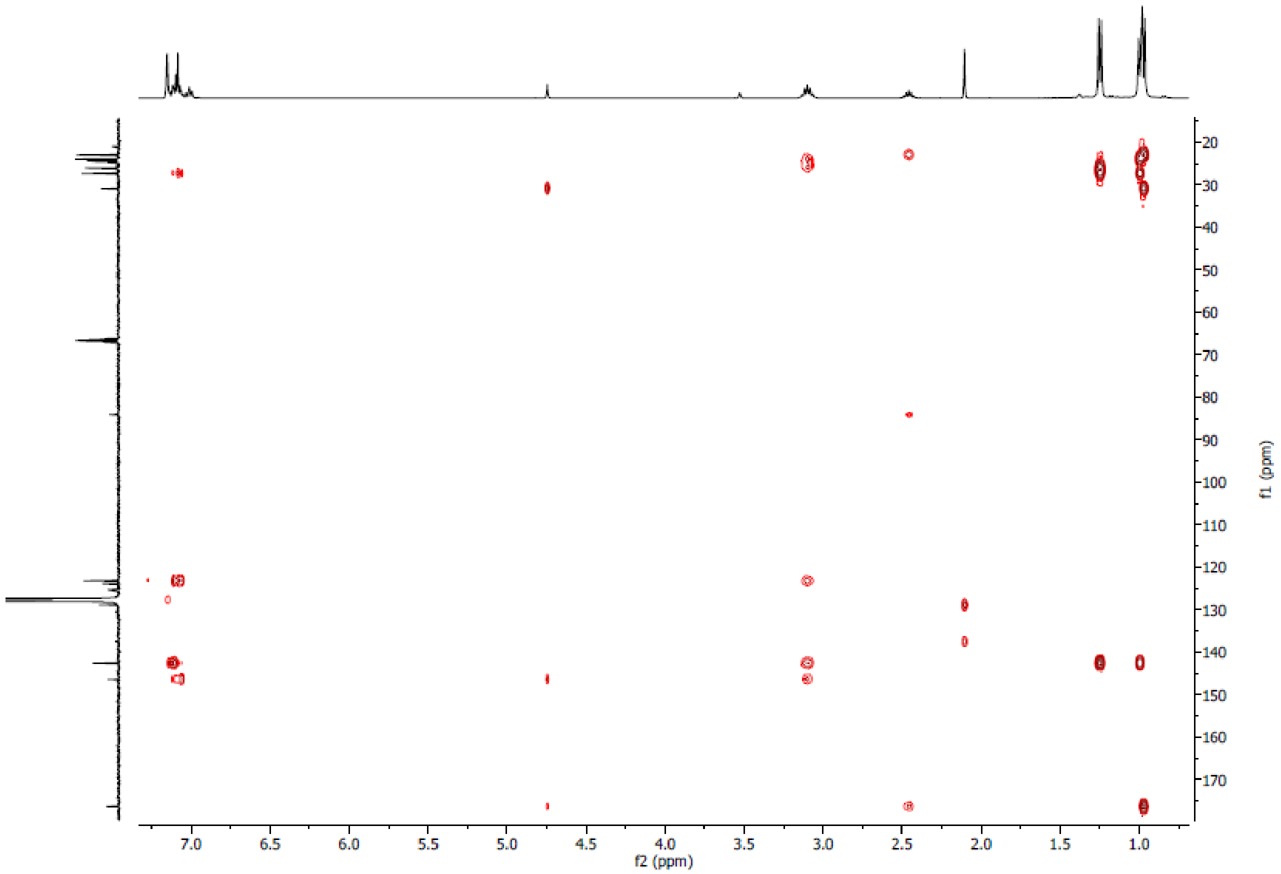

Figure S55. ^{1}H-^{13}C HMBC NMR spectrum of [{(^{iPrDip}nacnac)Mg(THF)}$_2$(μ-O)].

2.10 NMR spectra of [(^{iPrDip}nacnac)Mg(OAdH)] 8

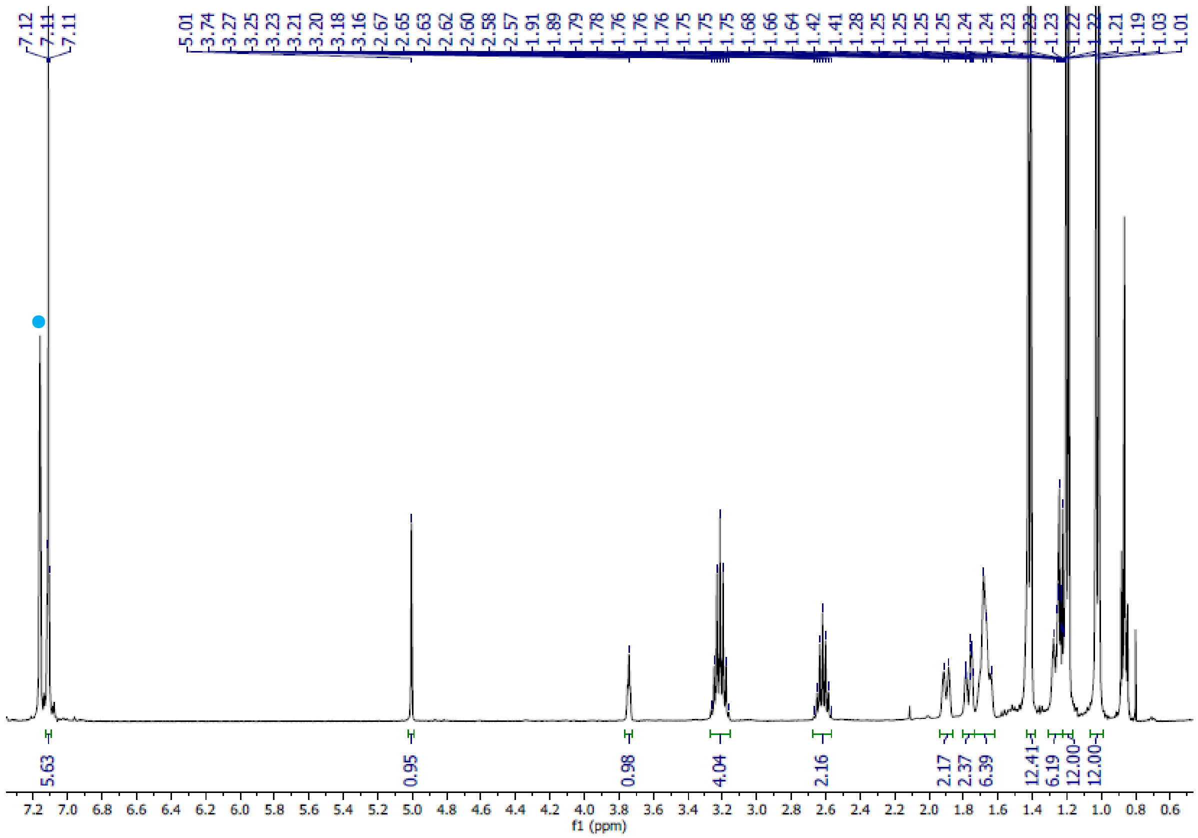

Figure S56. ^{1}H NMR spectrum (400.1 MHz, C_6D_6, 298 K) of [(^{iPrDip}nacnac)Mg(OAdH)] **8**.

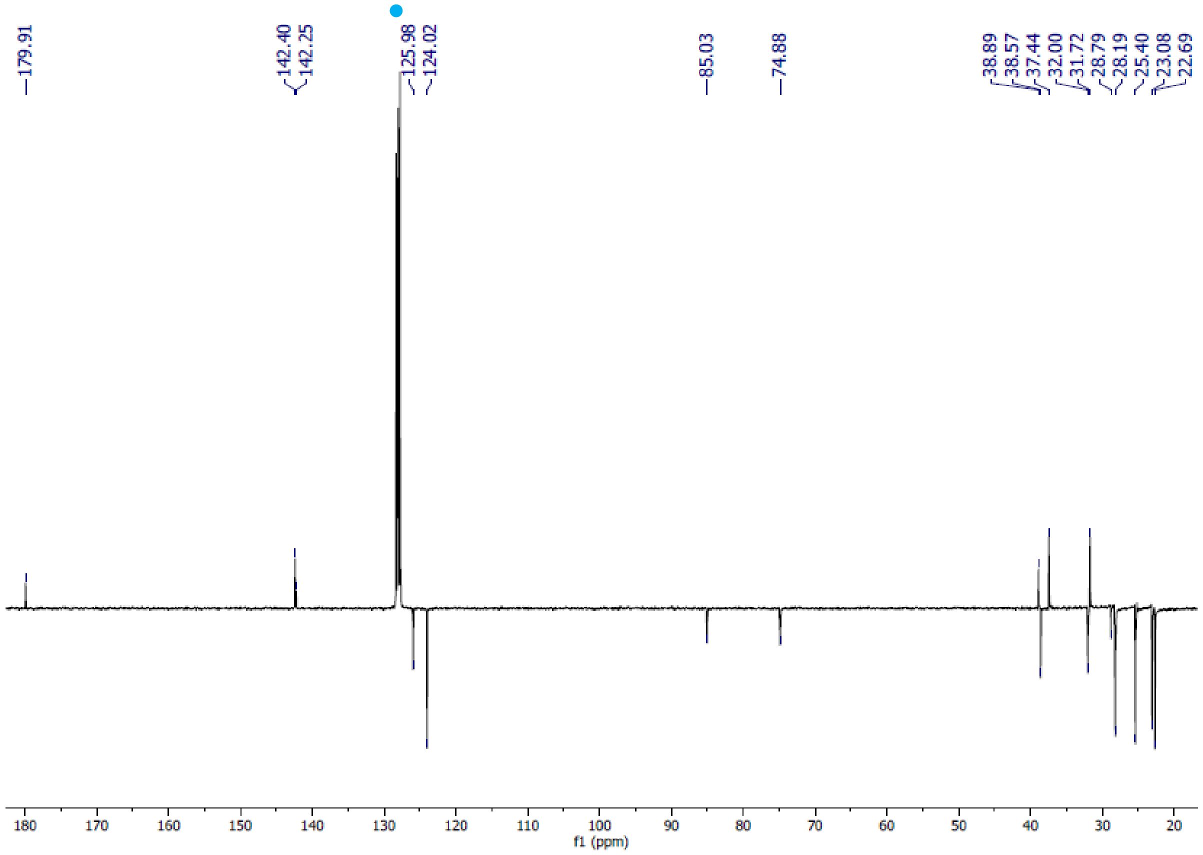

Figure S59. $^{13}C\{^1H\}$ (DEPT) NMR spectrum (100.5 MHz, C_6D_6, 298 K) of [(^{iPrDip}nacnac)Mg(OAdH)] **8**.

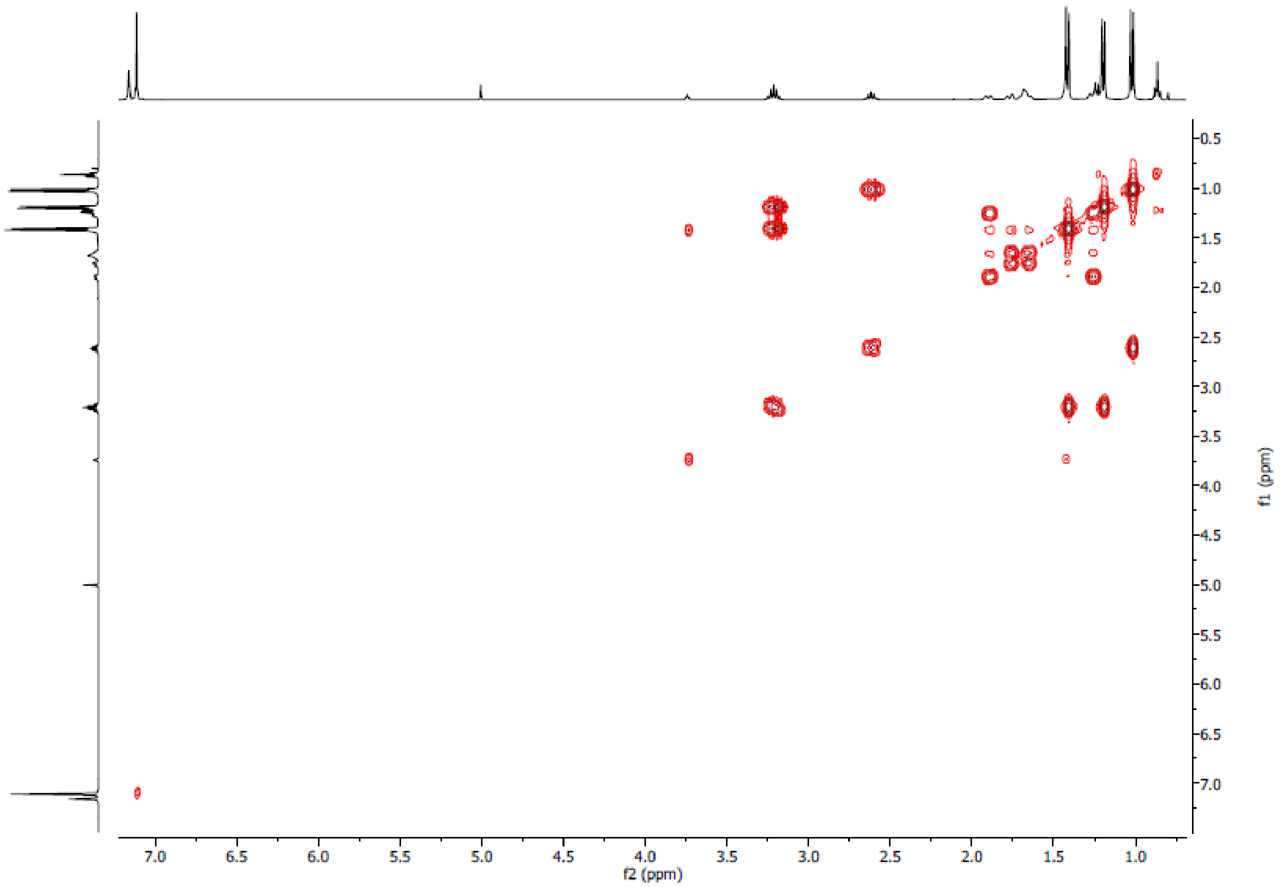

Figure S60. ^{1}H-^{1}H COSY NMR spectrum of [(^{iPrDip}nacnac)Mg(OAdH)] **8**.

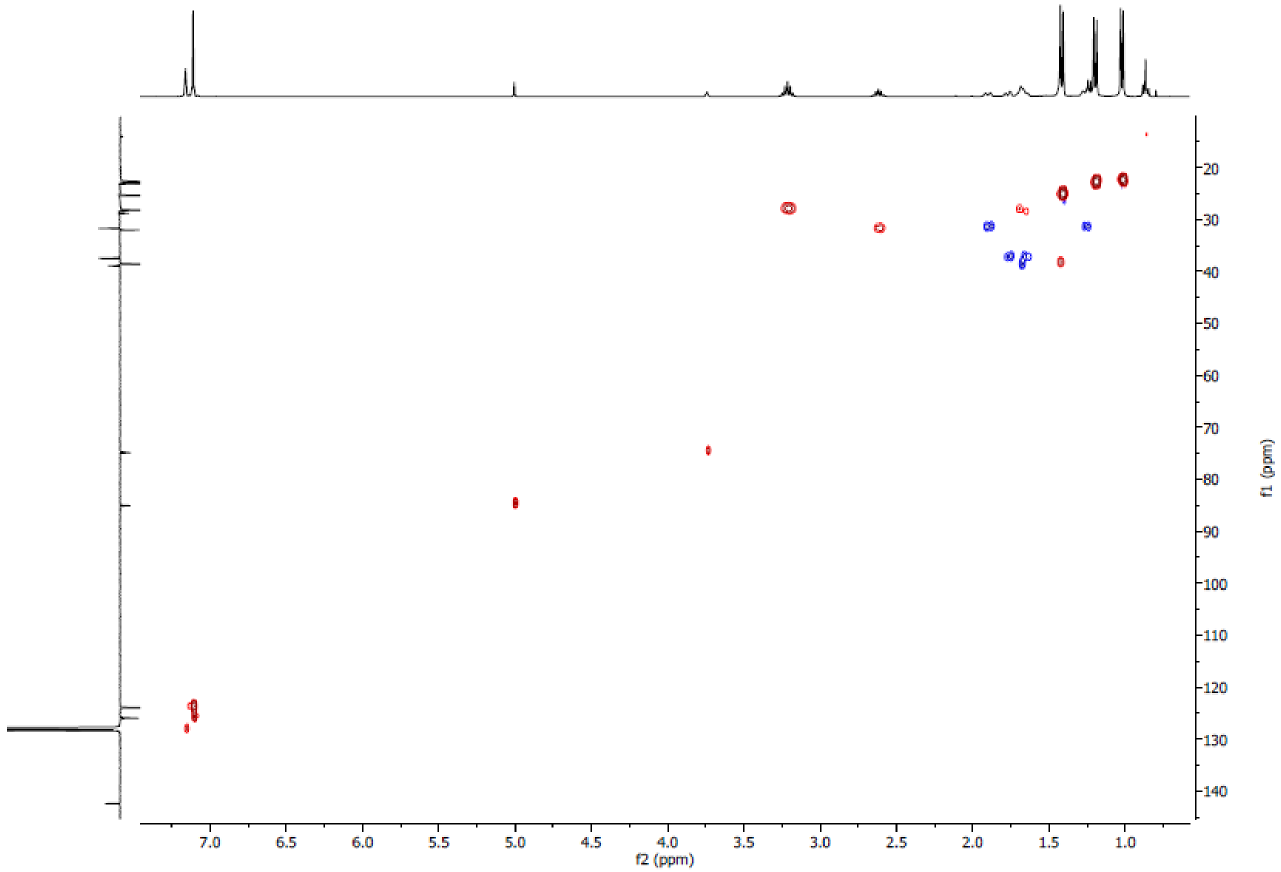

Figure S61. ^{1}H-^{13}C HSQC NMR spectrum of [(^{iPrDip}nacnac)Mg(OAdH)] **8**.

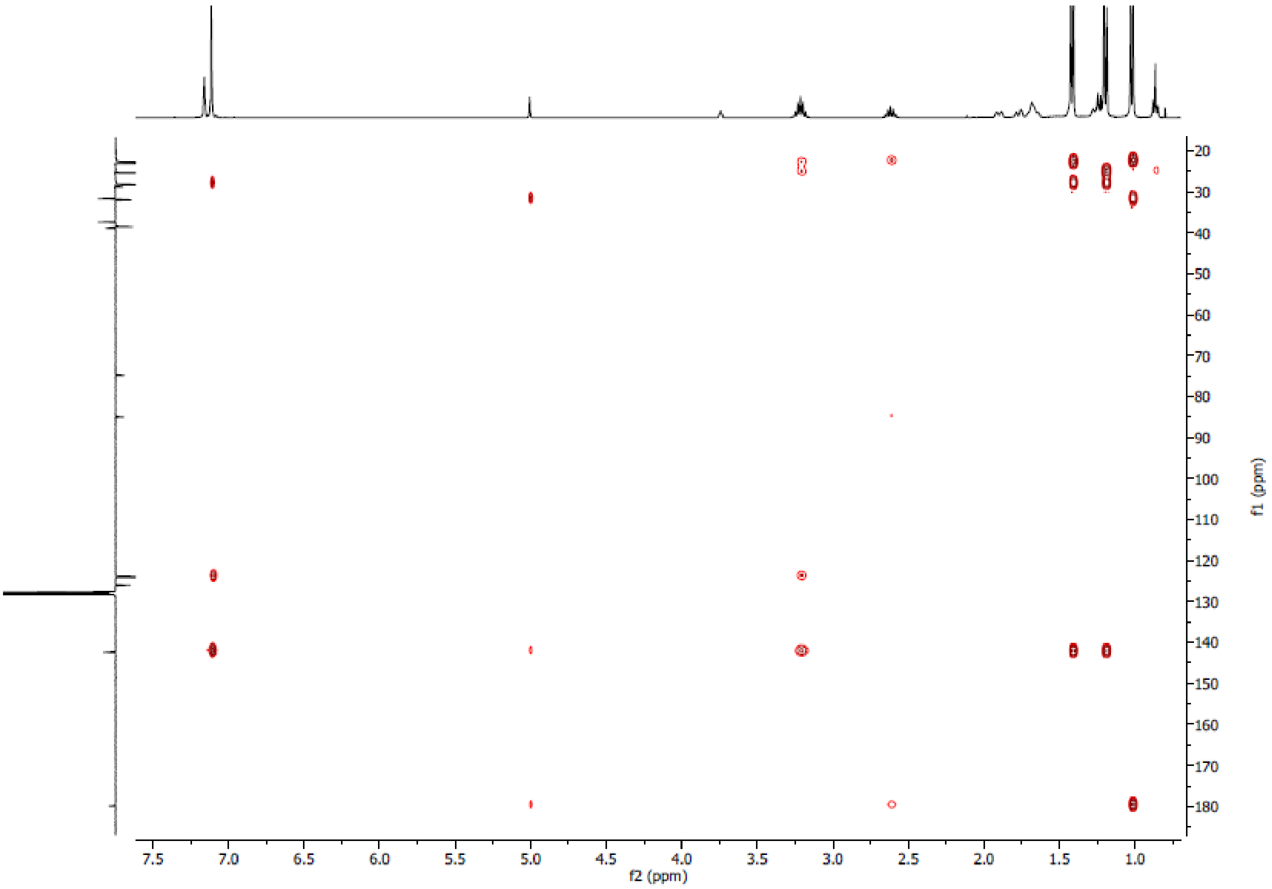

Figure S57. ^{1}H-^{13}C HMBC NMR spectrum of [(^{iPrDip}nacnac)Mg(OAdH)] **8**.

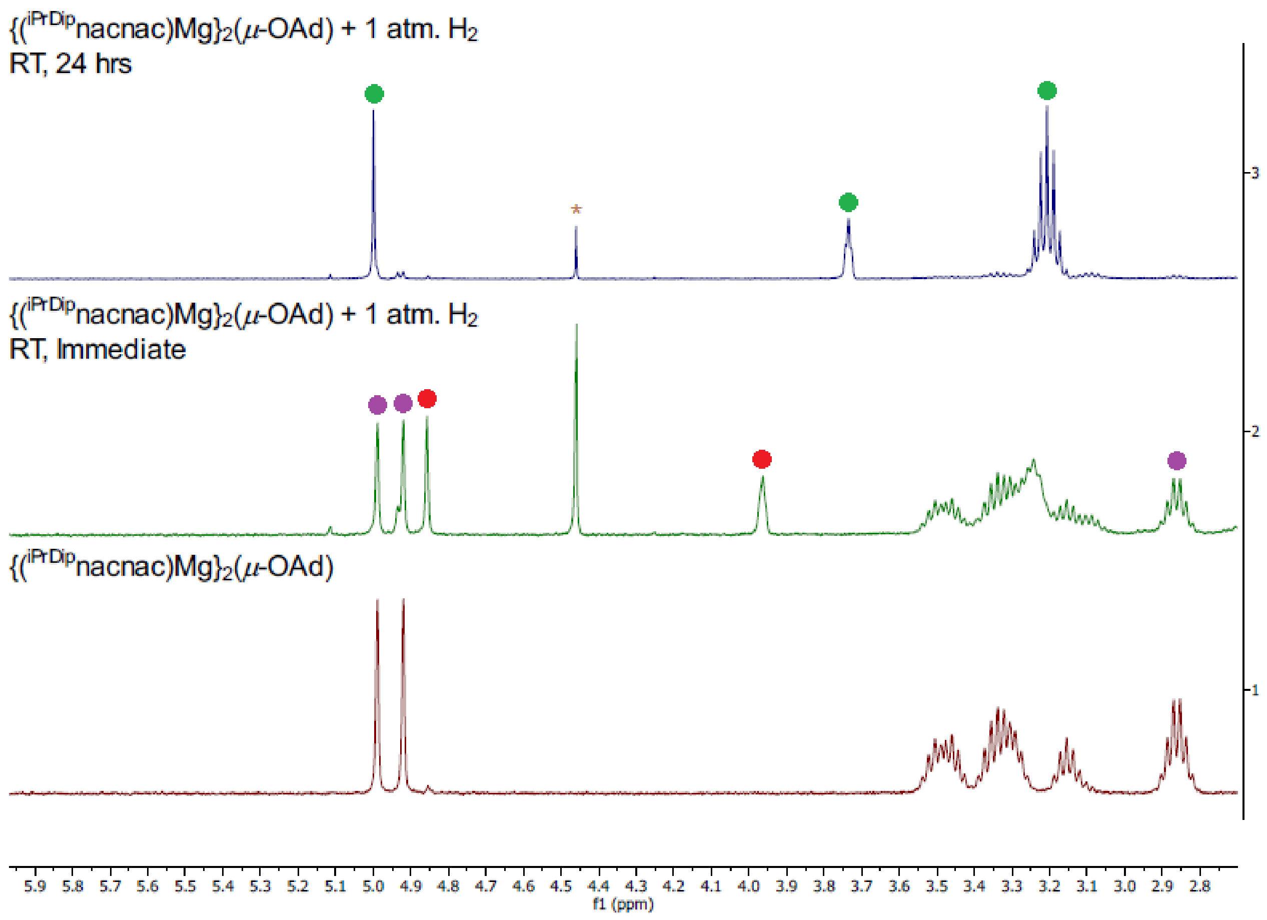

Figure S58. ^{1}H NMR spectroscopic time plots monitoring the room temperature reaction [{(^{iPrDip}nacnac)Mg}$_2$(μ-OAd)] **6** and *ca.* 1 atm. H_2. Green dots: [(^{iPrDip}nacnac)Mg(OAdH)] **8**; red dots: intermediate to **8**; purple dots: [{(^{iPrDip}nacnac)Mg}$_2$(μ-OAd)] **6**. The brown asterisk denotes residual H_2. An additional peak that could be a possible Mg-H resonance at *ca.* 4.45-4.47 ppm masked by residual H_2, see Figure S64.

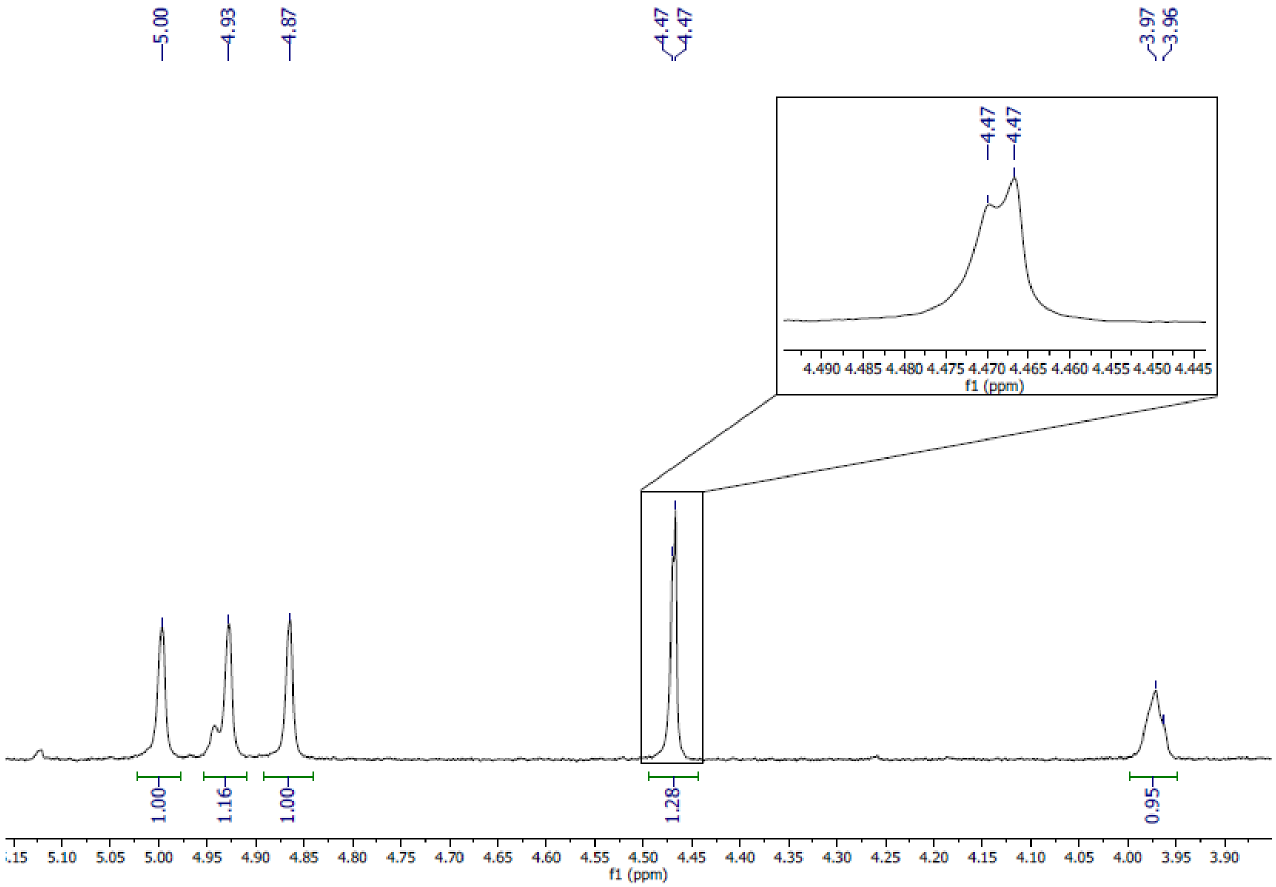

Figure S59. ^{1}H NMR spectrum (400.1 MHz, C_6D_6, 298 K) of intermediate complex in reaction of [{(^{iPrDip}nacnac)Mg}$_2$(μ-OAd)] **6** and H_2 (chemical shift range 3.85-5.15). The zoom shows the additional resonance at 4.47 ppm masked by residual H_2 which is absent at the end of the reaction. Note: a repeat experiment again provided complex **8** as one of the main end products and an additional as yet unidentified and unisolated product in the mixture, but again not [{(^{iPrDip}nacnac)Mg(μ-H)}$_2$] **5**. The additional peak at *ca.* 4.45-4.47 ppm in an intermediate was again observed and showed no cross-peaks in 2D NMR spectra of COSY, HSQC or HMBC experiments.

2.11 NMR spectra of [(^{iPrDip}nacnac)Mg(OCH*i*Pr$_2$)] 9 and [(^{iPrDip}nacnac)Mg{OC(=CMe$_2$)*i*Pr}] 10

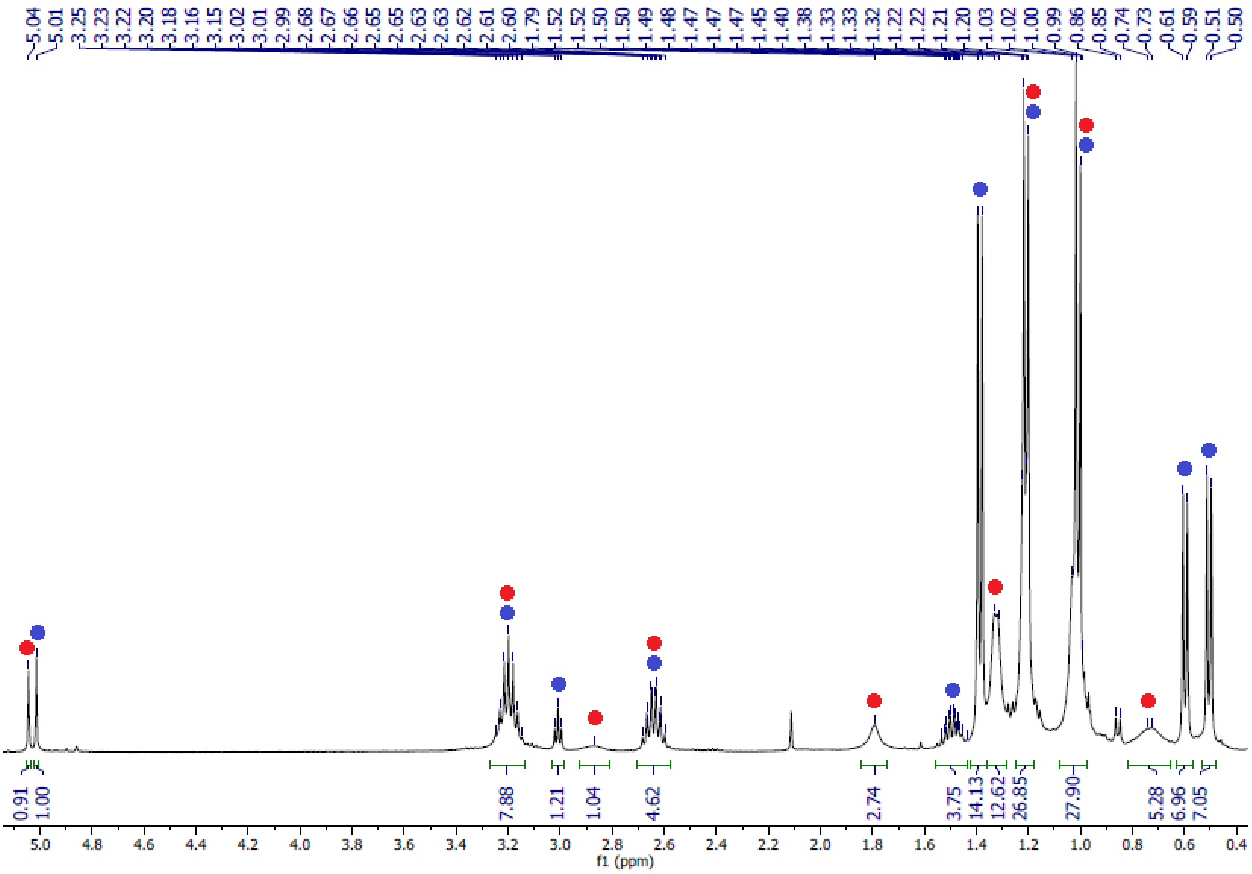

Figure S60. ^{1}H NMR spectrum (400.1 MHz, C_6D_6, 298 K) of [(^{iPrDip}nacnac)Mg(OCH*i*Pr$_2$)] **9** and [(^{iPrDip}nacnac)Mg{OC(=CMe$_2$)*i*Pr}] **10** (chemical shift range 0.4-5.1 ppm). Blue dots: [(^{iPrDip}nacnac)Mg(OCH*i*Pr$_2$)] **9**; red dots: [(^{iPrDip}nacnac)Mg{OC(=CMe$_2$)*i*Pr}] **10**.

2.12 NMR spectra of [(^{iPrDip}nacnac)Mg(OCH*i*Pr$_2$)] 9

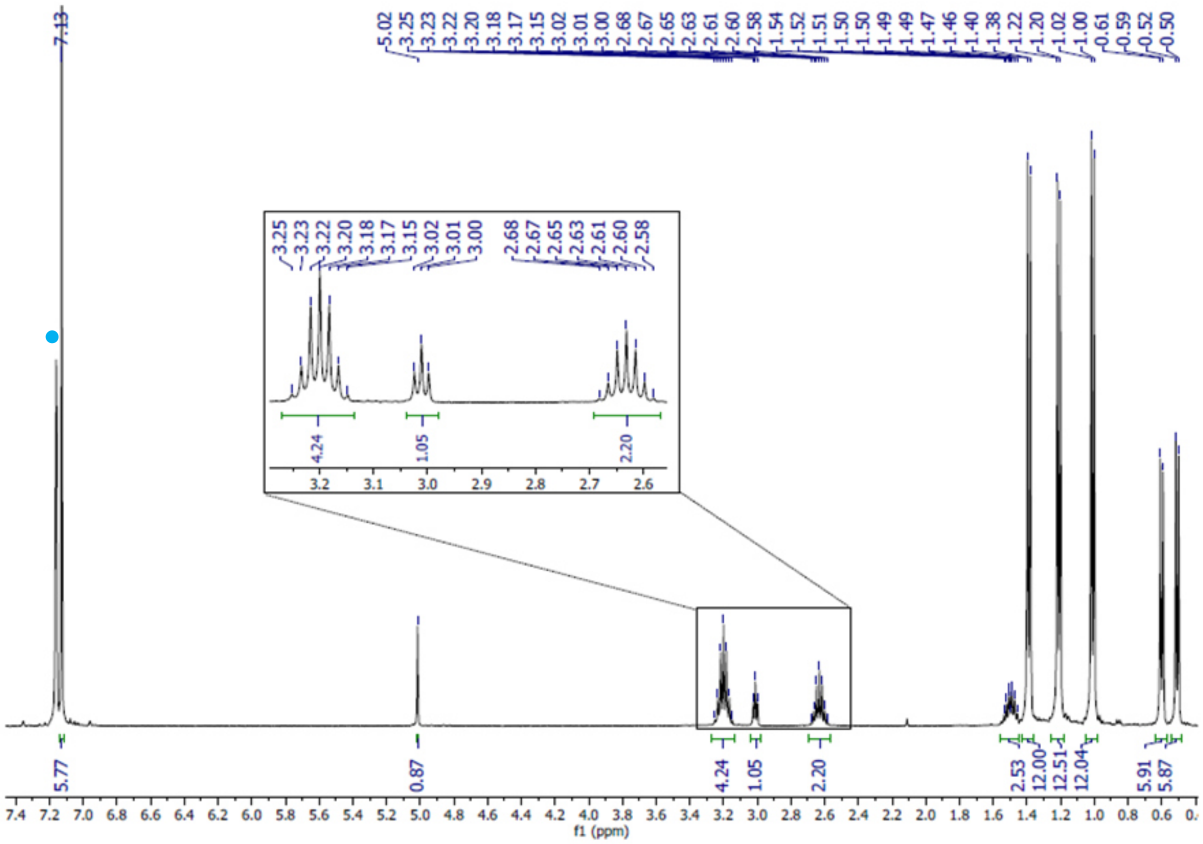

Figure S61. ^{1}H NMR spectrum (400.1 MHz, C_6D_6, 298 K) of [(^{iPrDip}nacnac)Mg(OCH*i*Pr$_2$)] **9**.

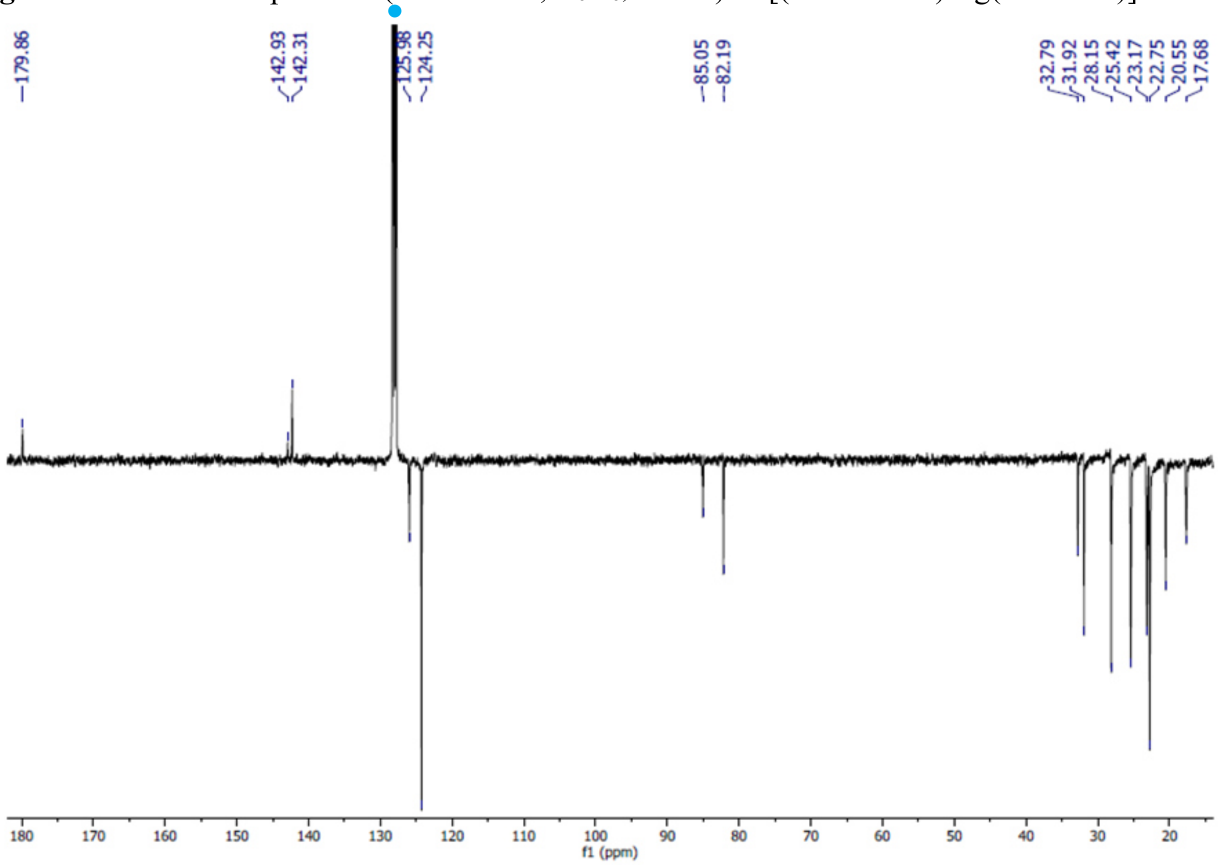

Figure S62. ^{13}C{^{1}H} (DEPT) NMR spectrum (100.5 MHz, C_6D_6, 298 K) of [(^{iPrDip}nacnac)Mg(OCH*i*Pr$_2$)] **9**.

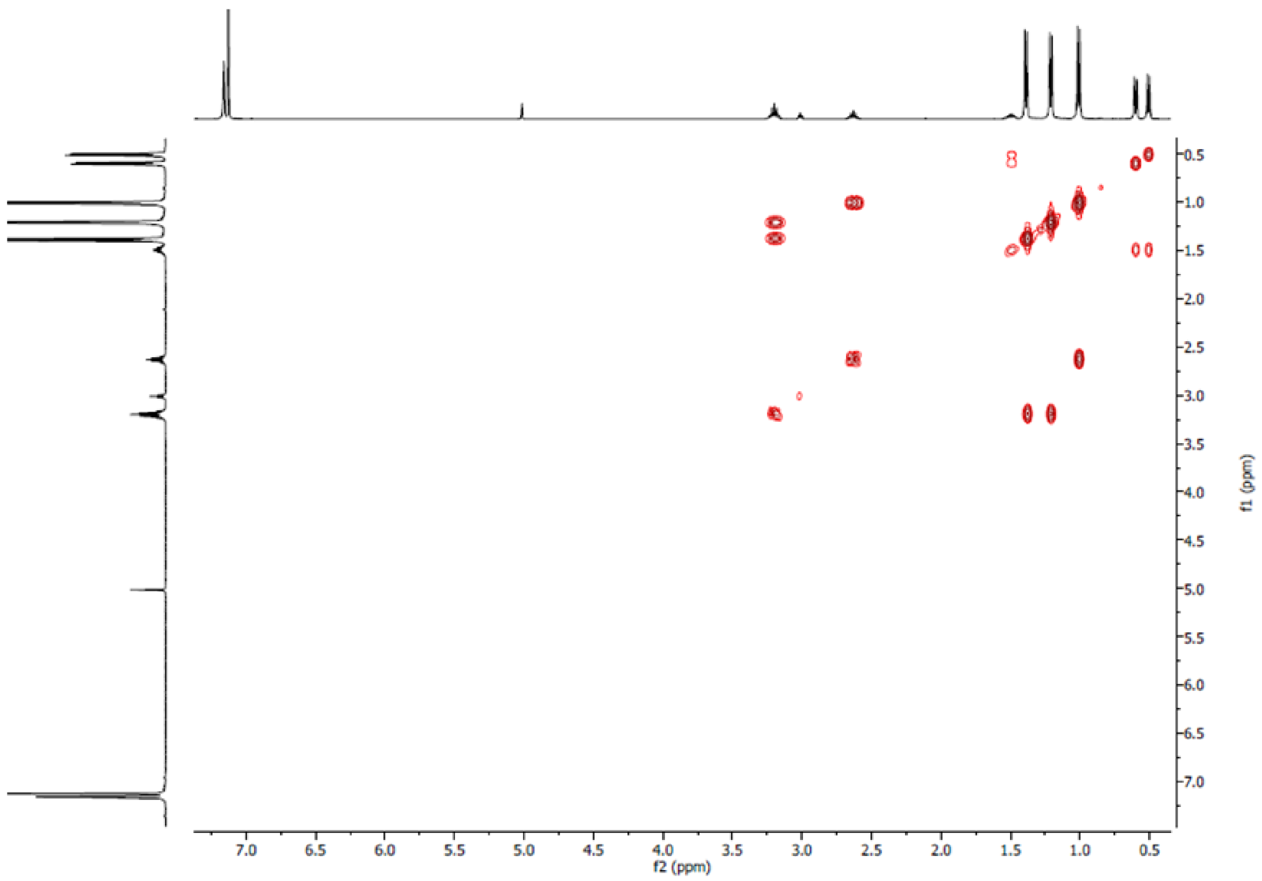

Figure S63. ^{1}H-^{1}H COSY NMR spectrum of [(^{iPrDip}nacnac)Mg(OCHiPr$_2$)] **9**.

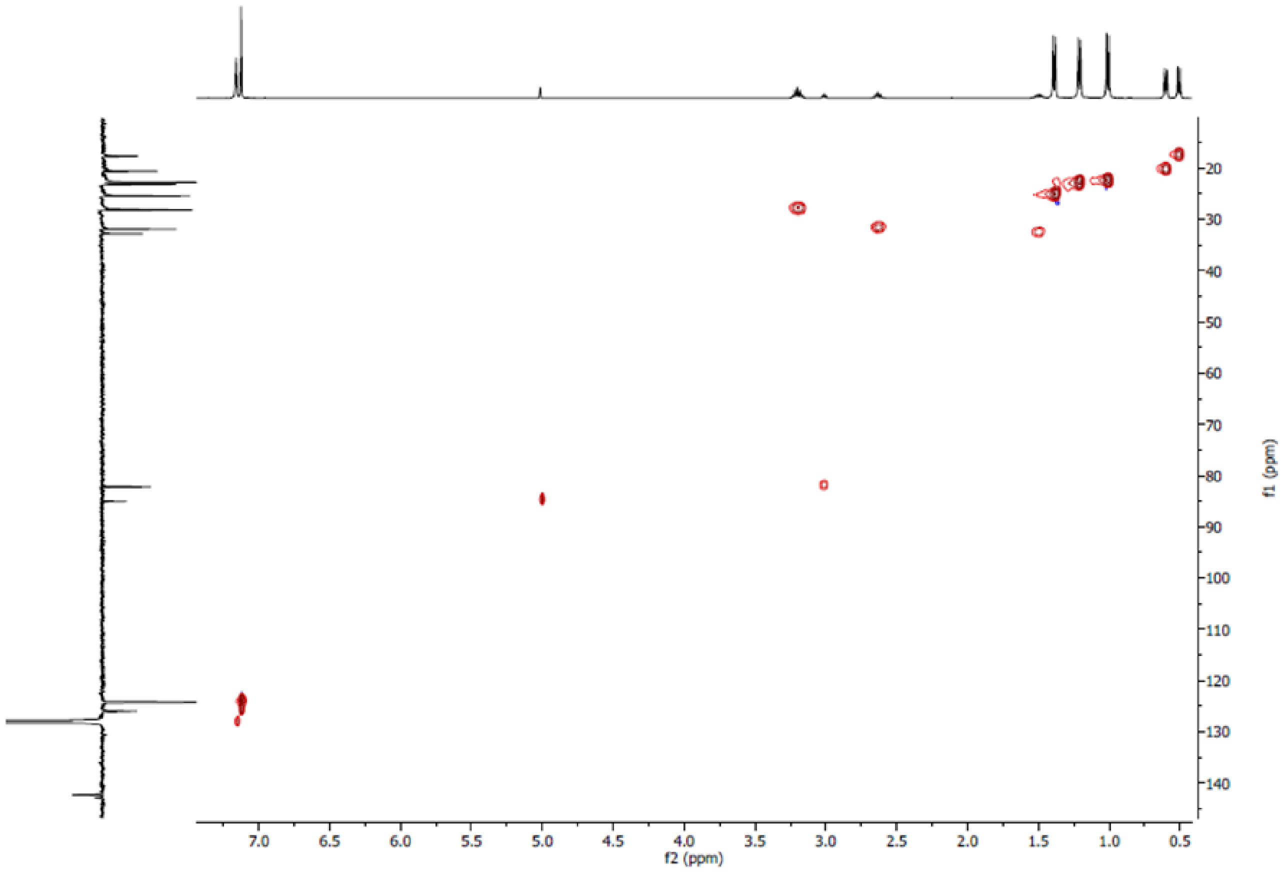

Figure S69. ^{1}H-^{13}C HSQC NMR spectrum of [(^{iPrDip}nacnac)Mg(OCHiPr$_2$)] **9**.

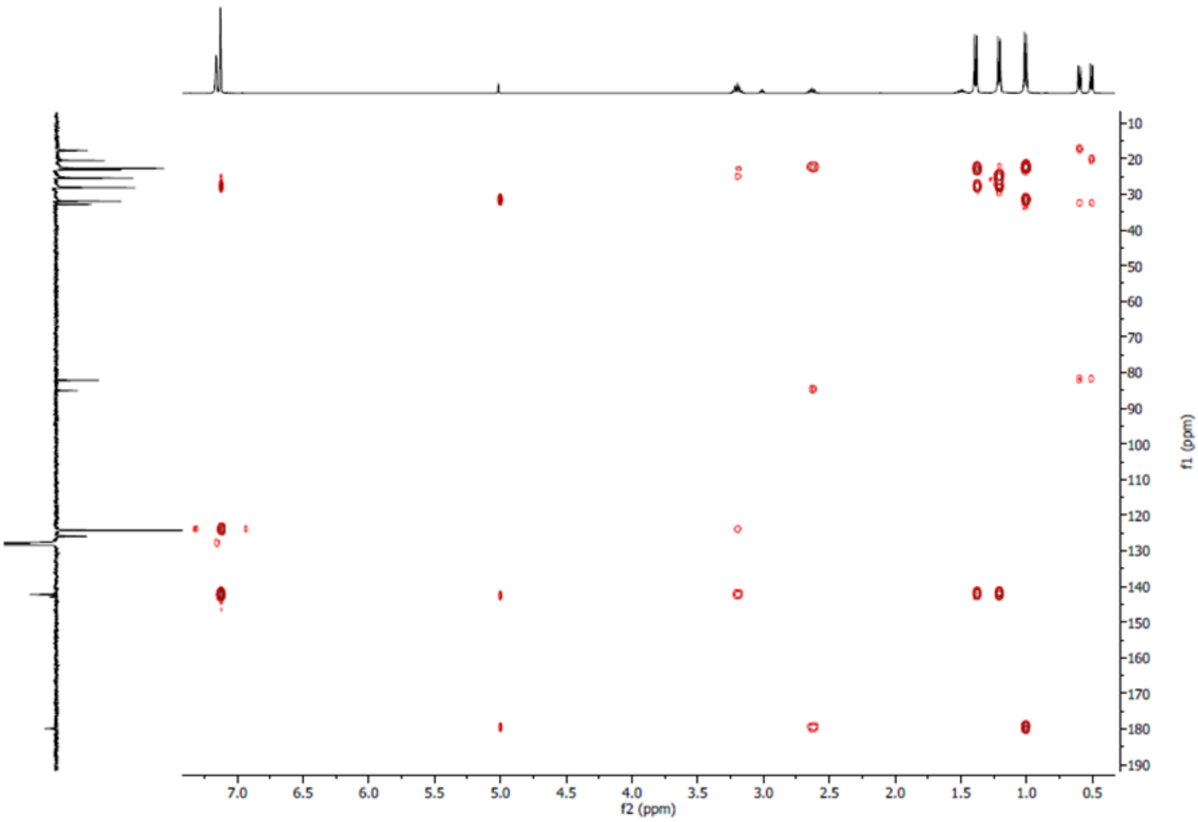

Figure S70. ^{1}H-^{13}C HMBC NMR spectrum of [(^{iPrDip}nacnac)Mg(OCH*i*Pr$_2$)] **9**.

2.13 NMR spectra of [(^{iPrDip}nacnac)Mg{OC(=CMe$_2$)*i*Pr}] 10

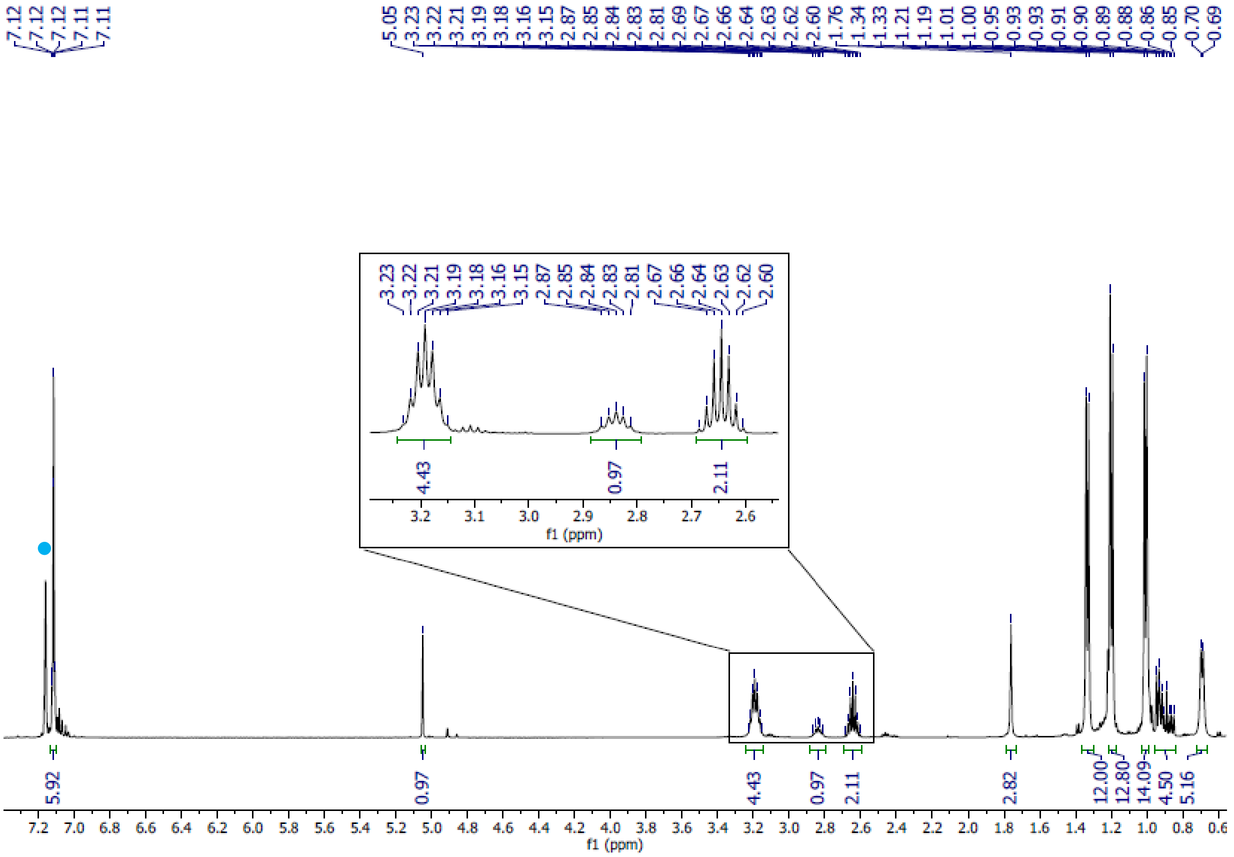

Figure S71. ^{1}H NMR spectrum (400.1 MHz, C_6D_6, 298 K) of [(^{iPrDip}nacnac)Mg{OC(=CMe$_2$)*i*Pr}] **10**.

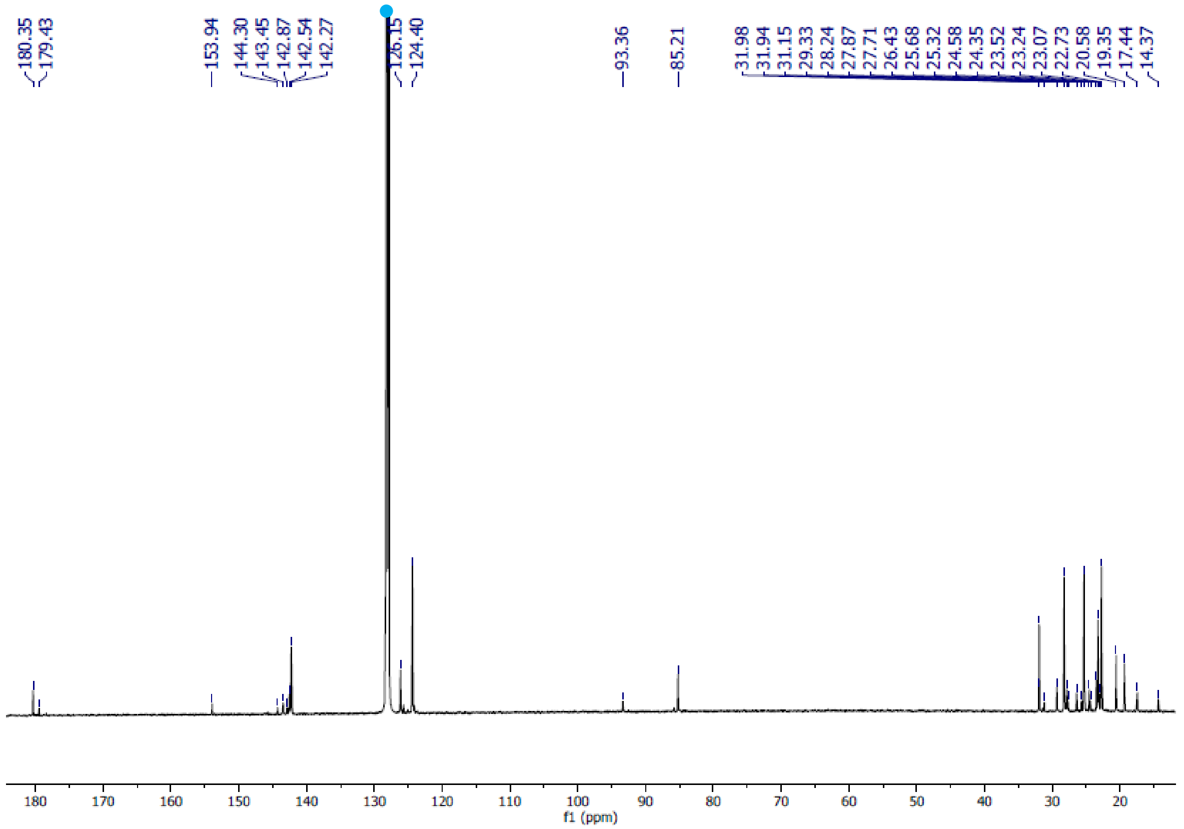

Figure S72. $^{13}C\{^1H\}$ NMR spectrum (100.5 MHz, C_6D_6, 298 K) of [(^{iPrDip}nacnac)Mg{OC(=CMe$_2$)*i*Pr}] **10**.

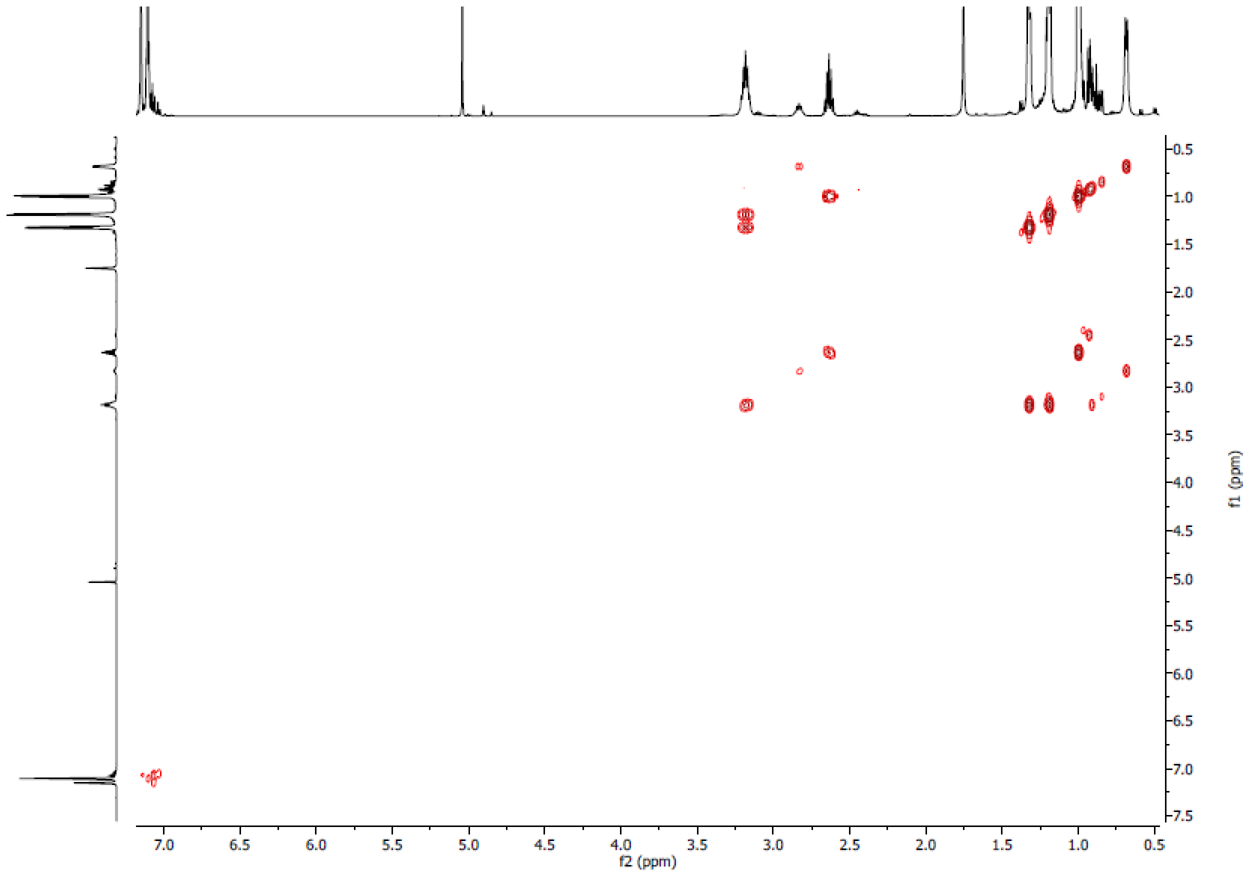

Figure S64. ^1H-^1H COSY NMR spectrum of [(^{iPrDip}nacnac)Mg{OC(=CMe$_2$)*i*Pr}] **10**.

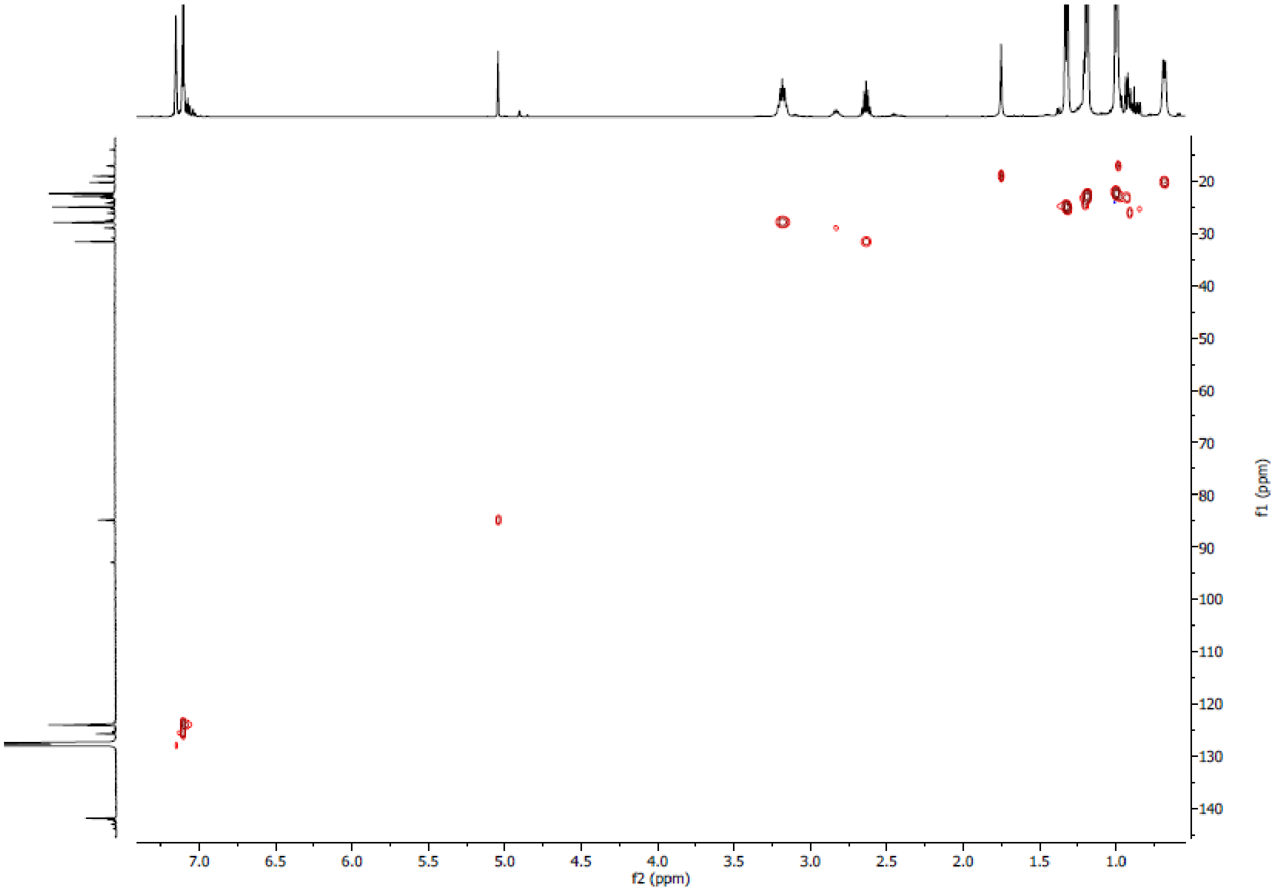

Figure S65. ^{1}H-^{13}C HSQC NMR spectrum of [(^{iPrDip}nacnac)Mg{OC(=CMe$_2$)*i*Pr}] **10**.

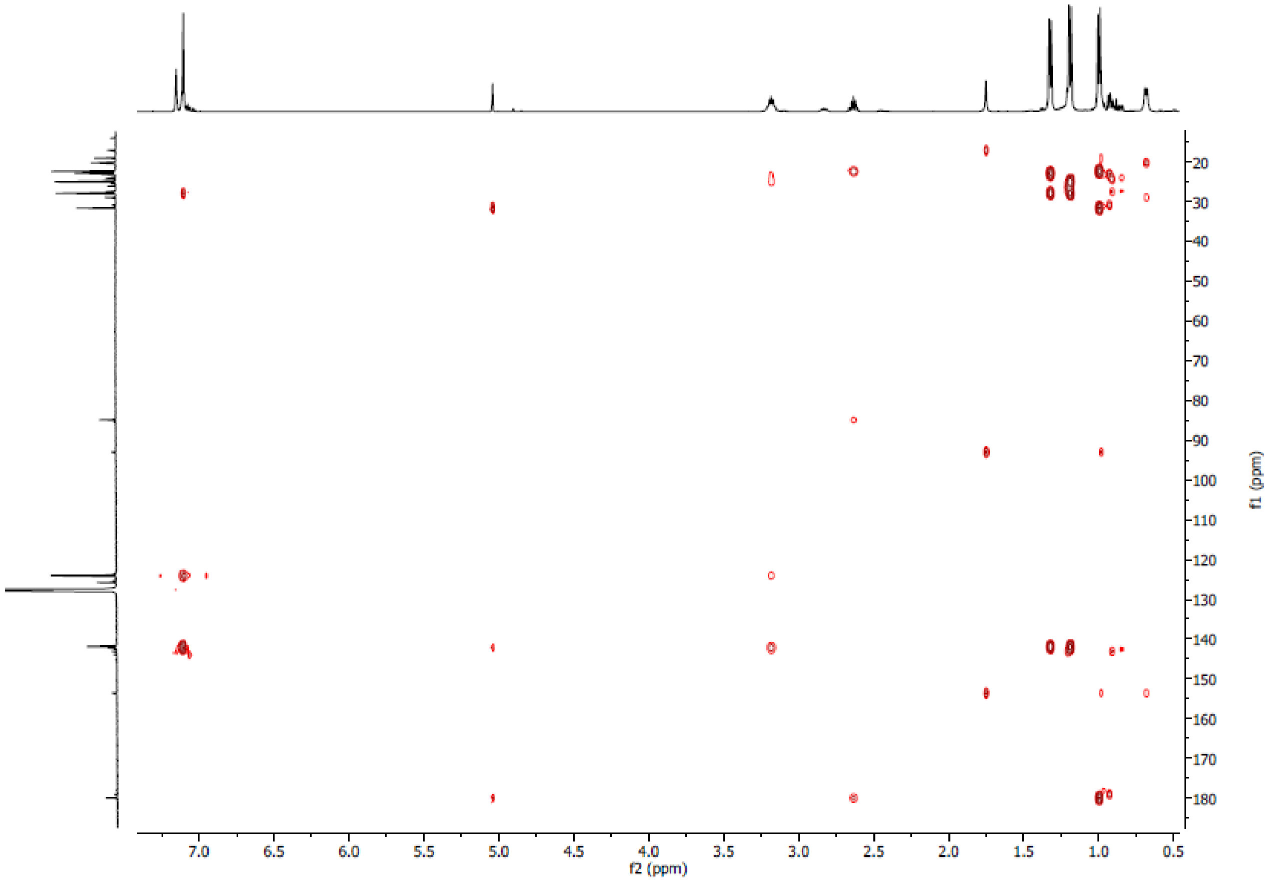

Figure S66. ^{1}H-^{13}C HMBC NMR spectrum of [(^{iPrDip}nacnac)Mg{OC(=CMe$_2$)*i*Pr}] **10**.

2.14 NMR spectra of [(^{iPrDip}nacnac)Mg(OCH*t*Bu$_2$)] 11

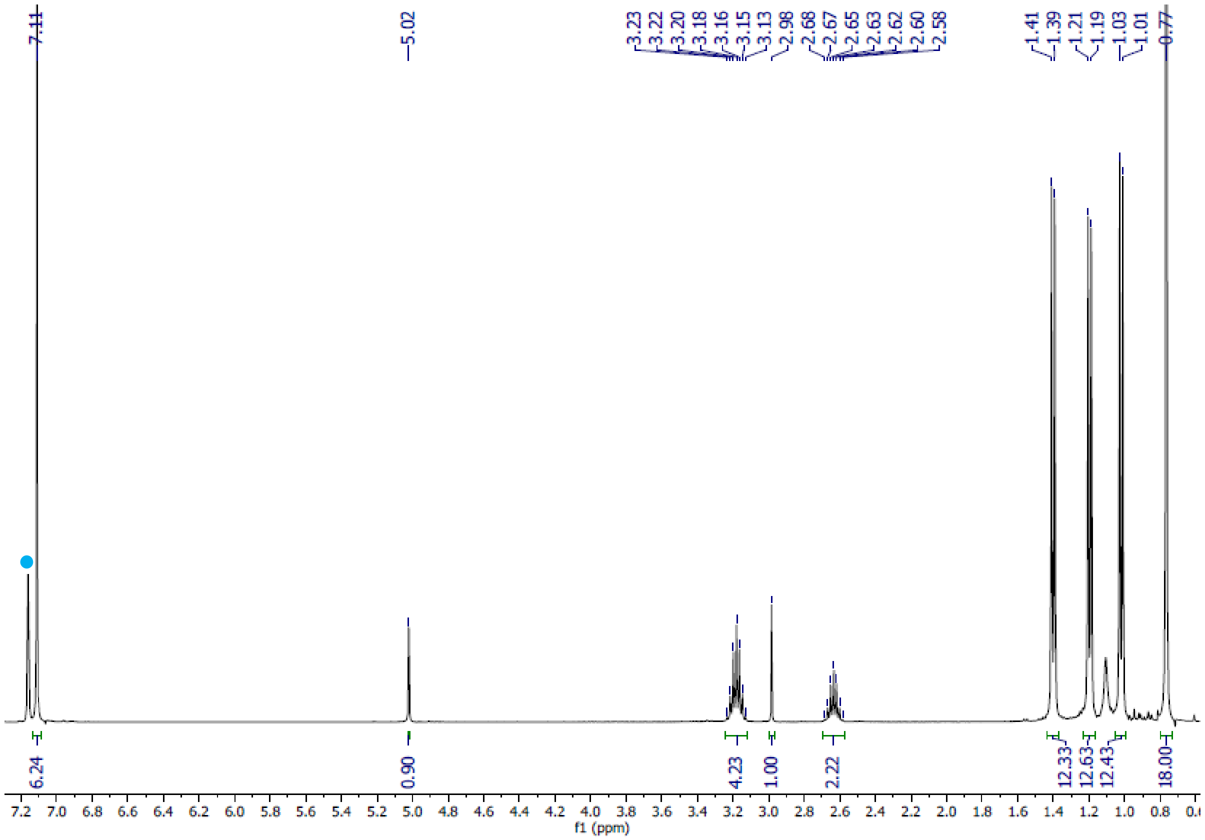

Figure S67. ^{1}H NMR spectrum (400.1 MHz, C_6D_6, 298 K) of [(^{iPrDip}nacnac)Mg(OCH*t*Bu$_2$)] **11**.

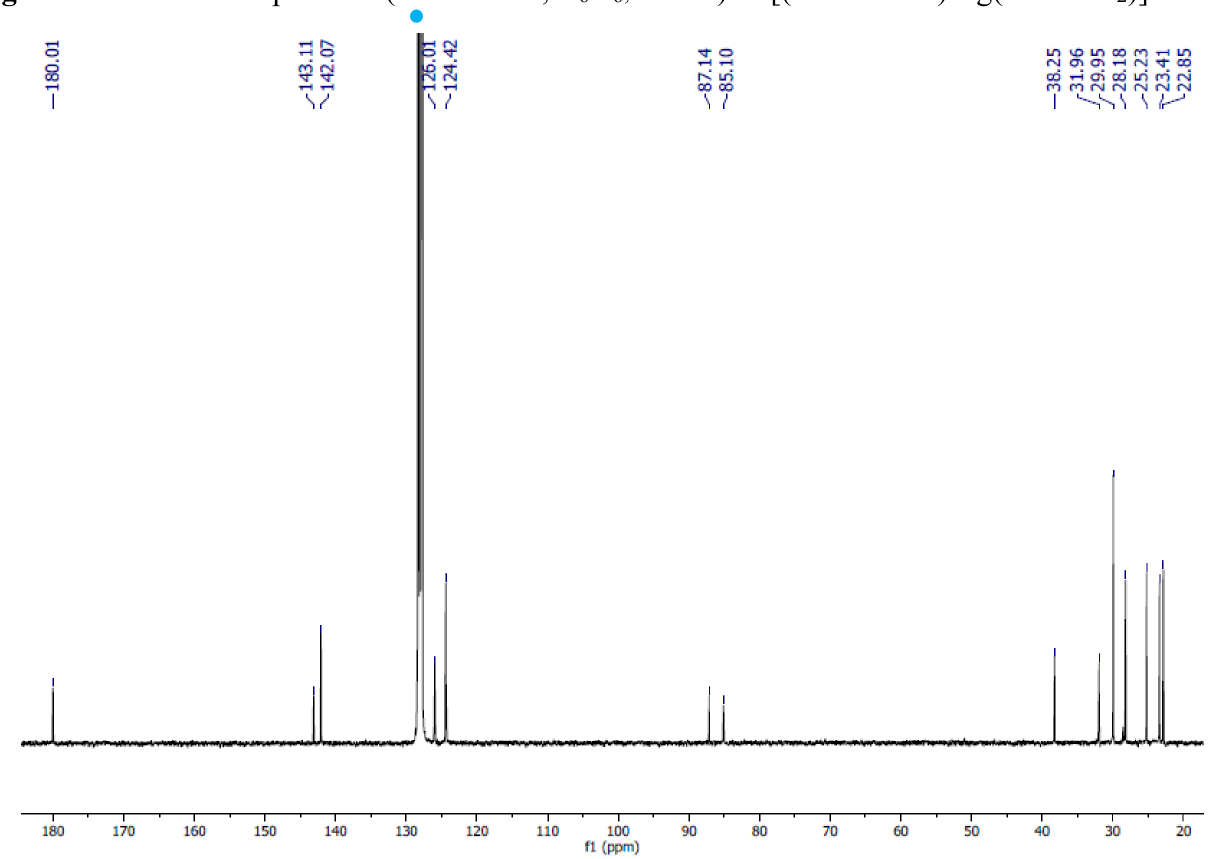

Figure S68. ^{13}C{^{1}H} NMR spectrum (100.5 MHz, C_6D_6, 298 K) of [(^{iPrDip}nacnac)Mg(OCH*t*Bu$_2$)] **11**.

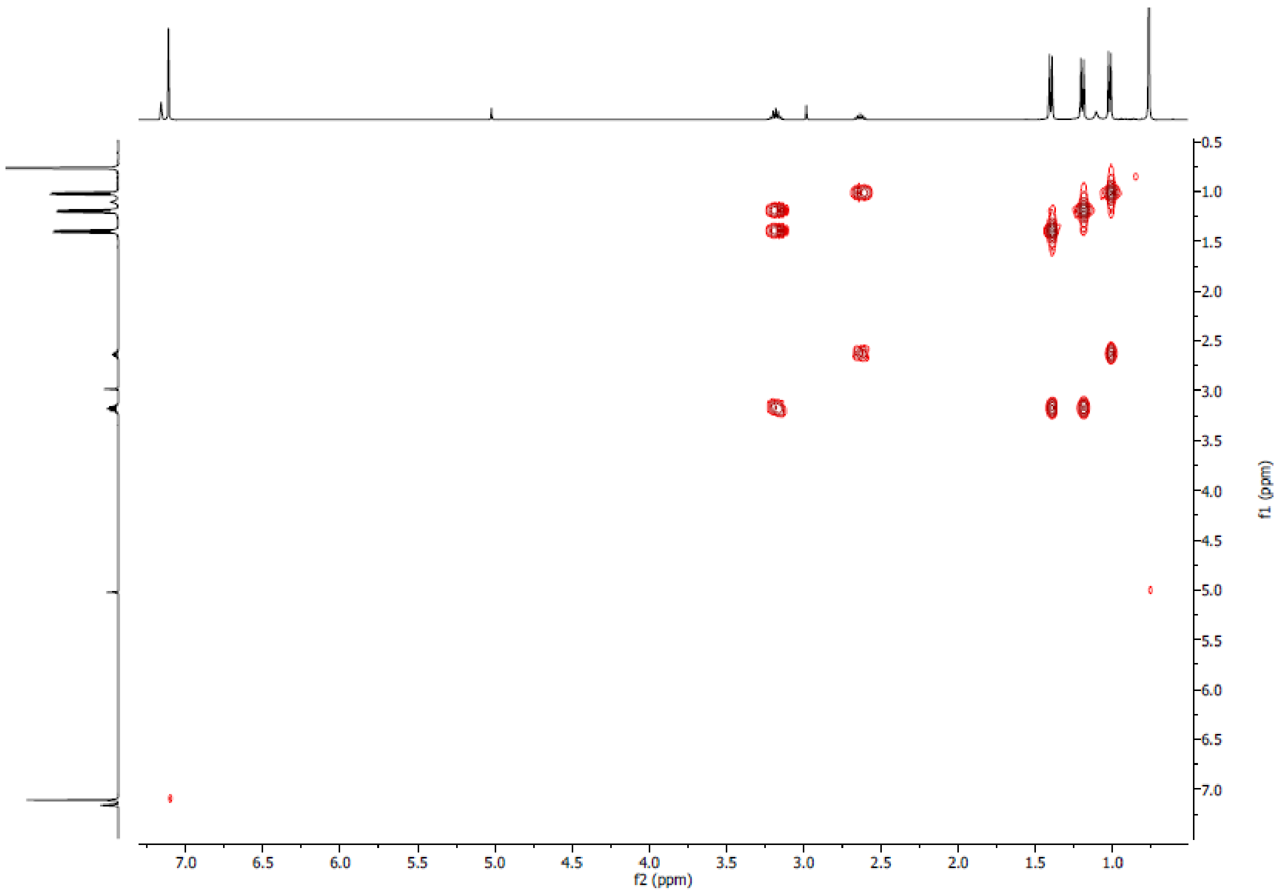

Figure S69. ^{1}H-^{1}H COSY NMR spectrum of [(^{iPrDip}nacnac)Mg(OCH*t*Bu$_2$)] **11**.

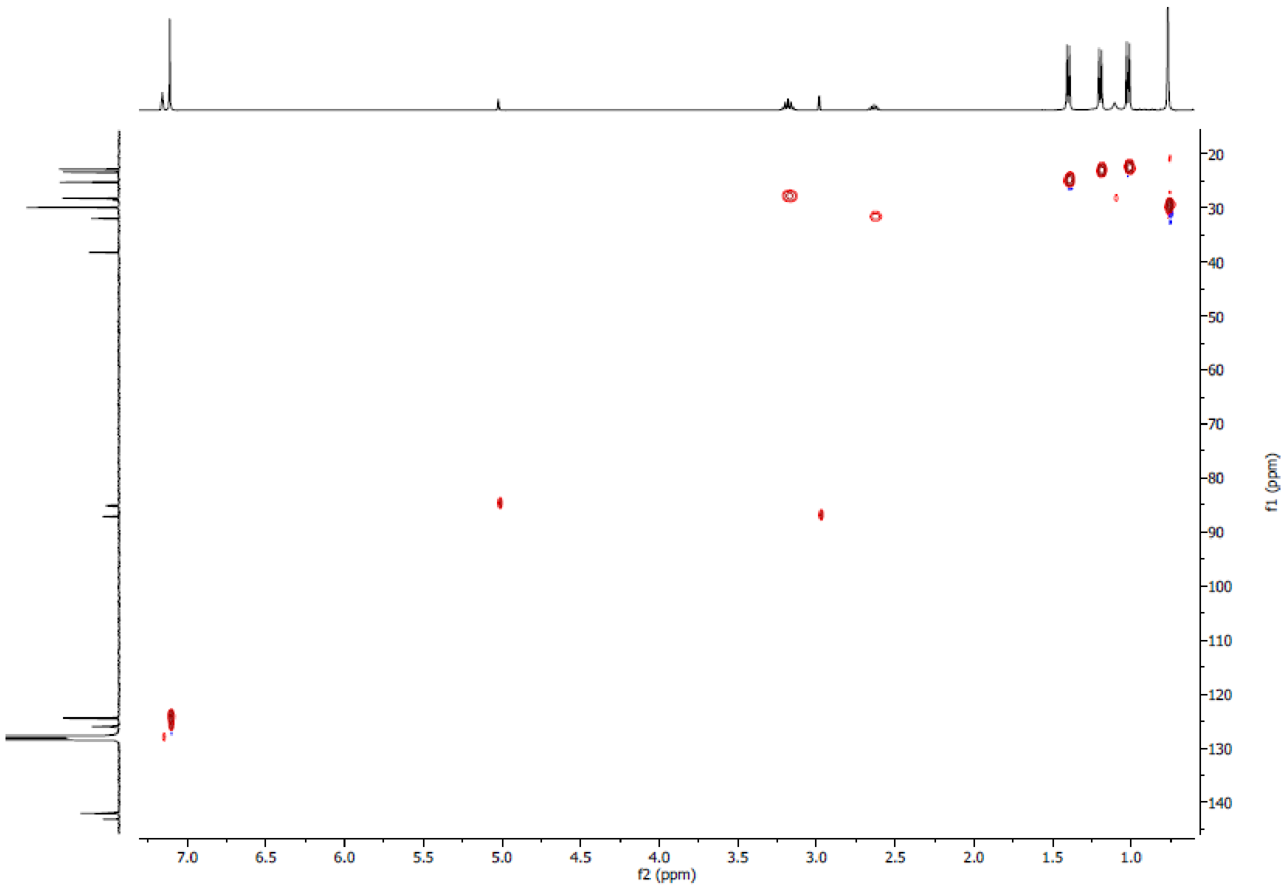

Figure S70. ^{1}H-^{13}C HSQC NMR spectrum of [(^{iPrDip}nacnac)Mg(OCH*t*Bu$_2$)] **11**.

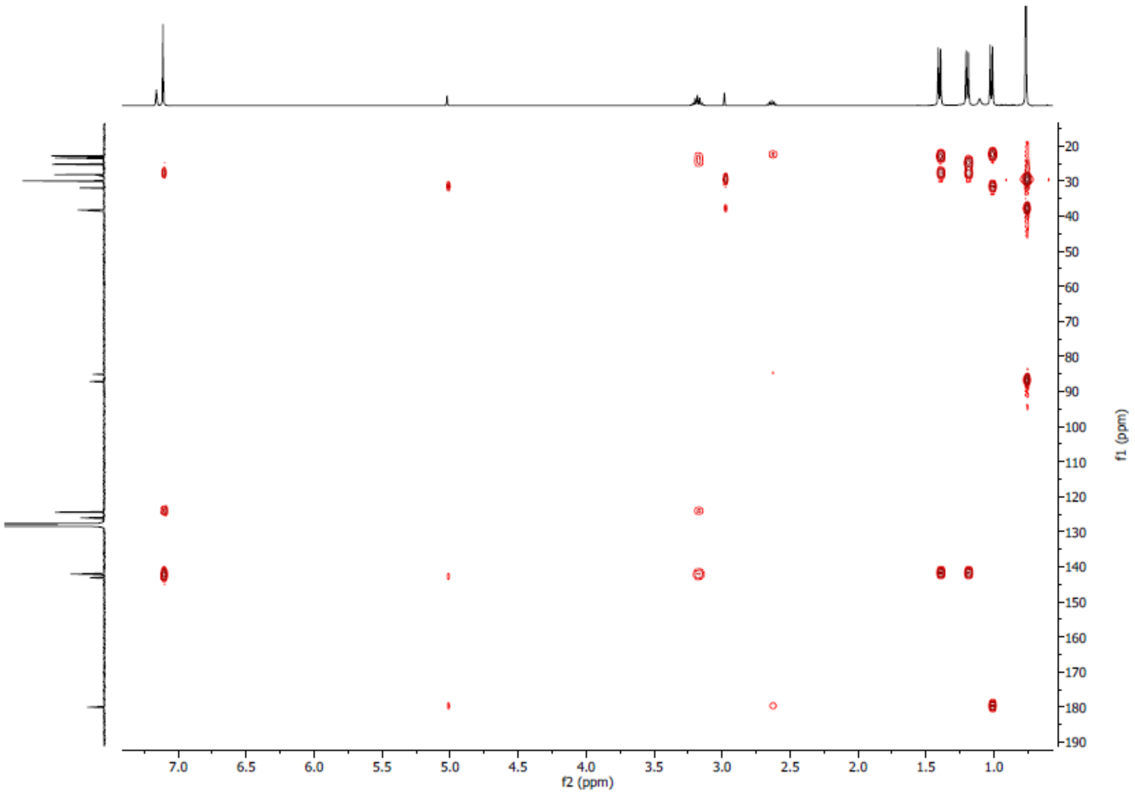

Figure S80. ^{1}H-^{13}C HMBC NMR spectrum of [(^{iPrDip}nacnac)Mg(OCH*t*Bu$_2$)] **11**.

2.15 NMR spectra of [{(^{MeMes}nacnac)Mg}(*μ*-OCH*t*Bu$_2$)(*μ*-CH$_2$-$^{MeMes-H}$nacnac)Mg] 12

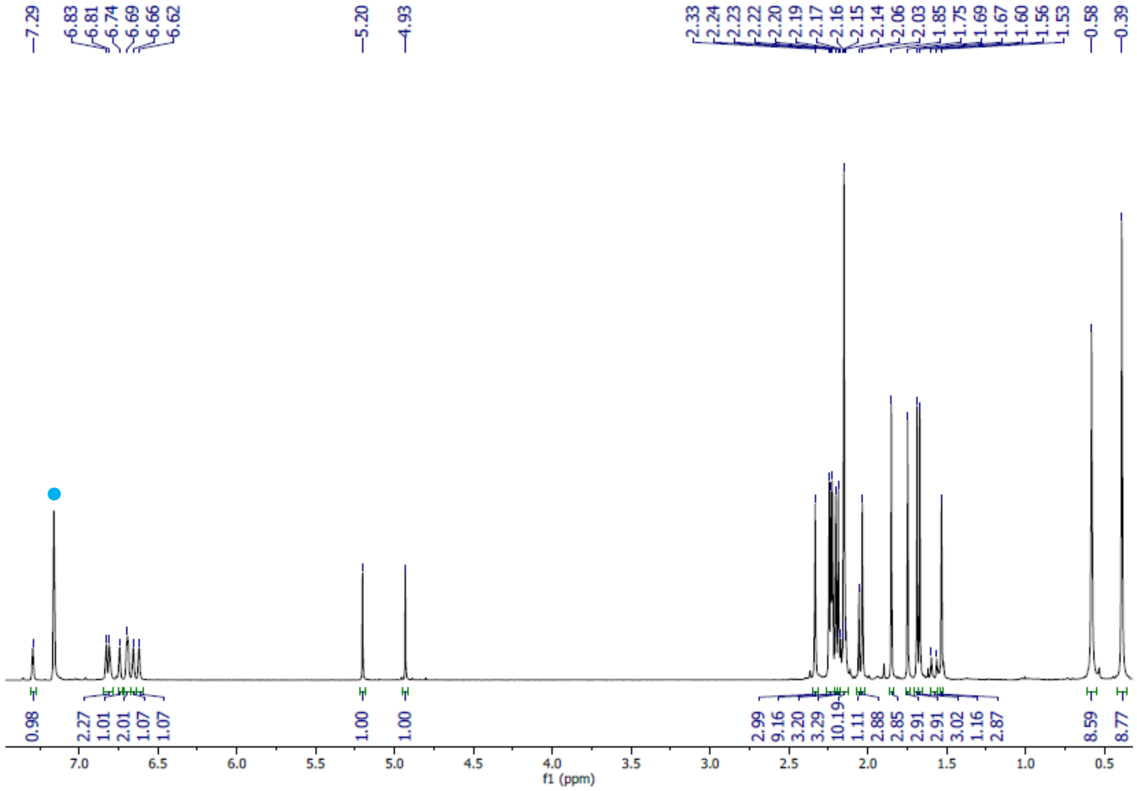

Figure S81. ^{1}H NMR spectrum (400.1 MHz, C_6D_6, 298 K) of [{(^{MeMes}nacnac)Mg}(*μ*-OCH*t*Bu$_2$)(*μ*-CH$_2$-$^{MeMes-H}$nacnac)Mg] **12**.

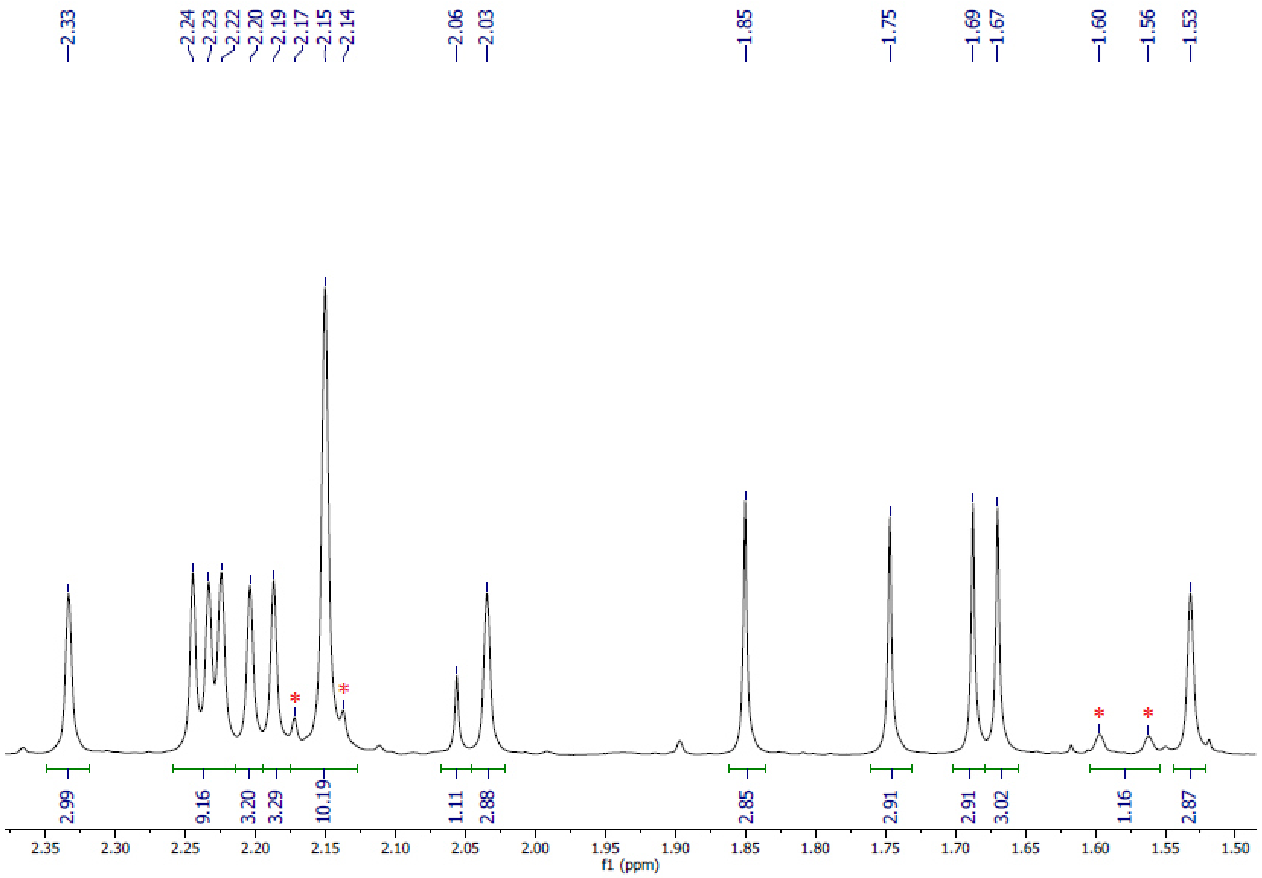

Figure S82. ^{1}H NMR spectrum (400.1 MHz, C_6D_6, 298 K) of [{(^{MeMes}nacnac)Mg}(*μ*-OCH*t*Bu_2)(*μ*-CH_2-$^{MeMes\text{-}H}$nacnac)Mg] **12** (chemical shift range 1.50-2.35 ppm). Red asterisks denote Ar-CH_2Mg chemical shifts.

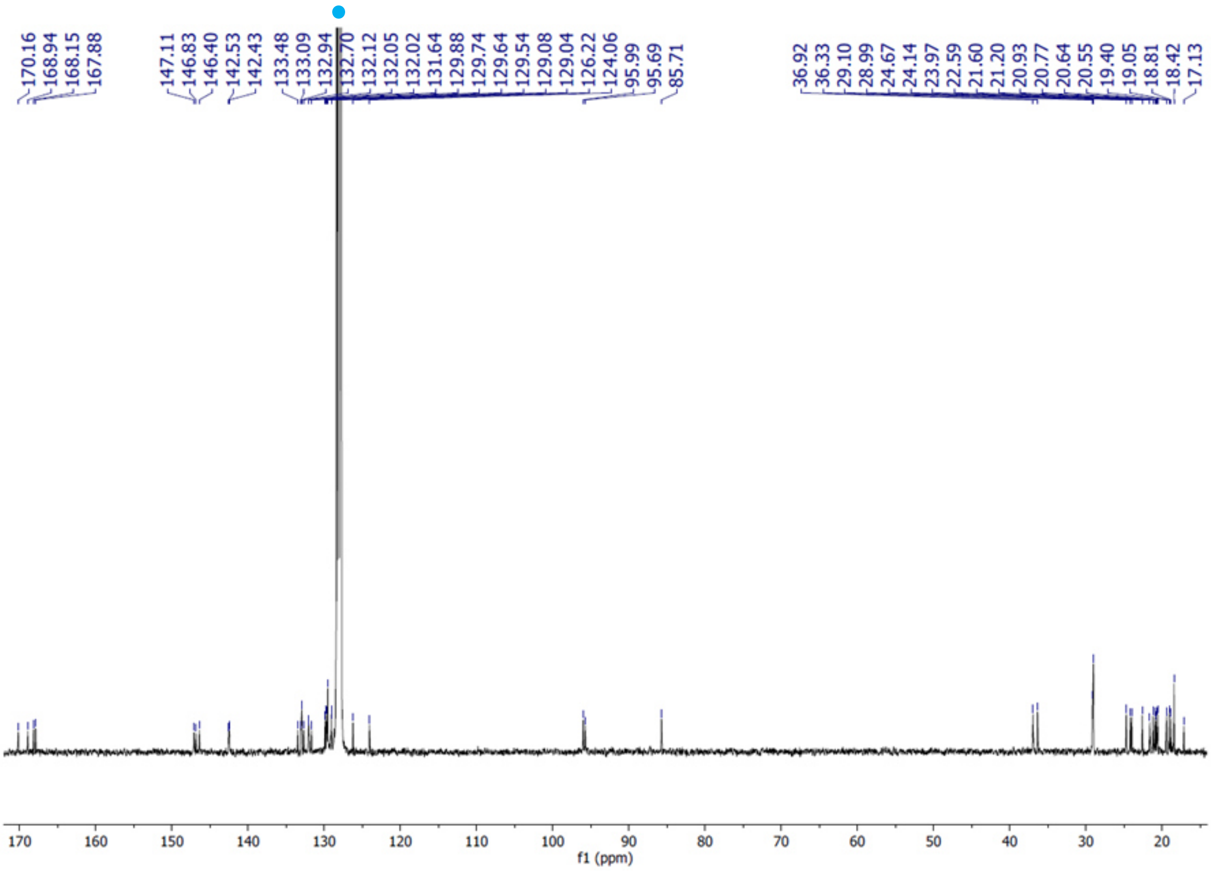

Figure S71. $^{13}C\{^{1}H\}$ NMR spectrum (100.5 MHz, C_6D_6, 298 K) of [{(^{MeMes}nacnac)Mg}(μ-OCHtBu$_2$)(μ-CH$_2$-$^{MeMes\text{-}H}$nacnac)Mg] **12**.

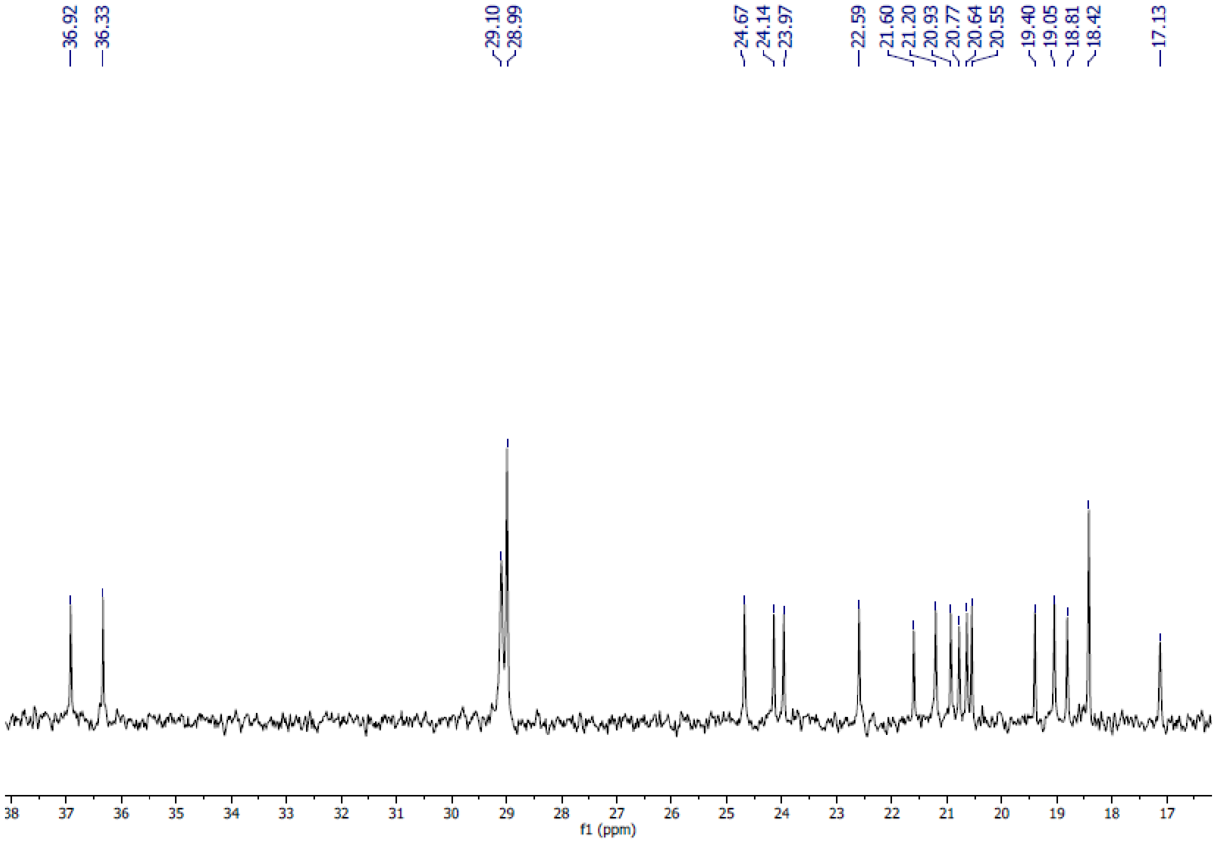

Figure S72. $^{13}C\{^{1}H\}$ NMR spectrum (100.5 MHz, C_6D_6, 298 K) of [{(^{MeMes}nacnac)Mg}(μ-OCHtBu$_2$)(μ-CH$_2$-$^{MeMes\text{-}H}$nacnac)Mg] **12** (chemical shift range 16-38 ppm).

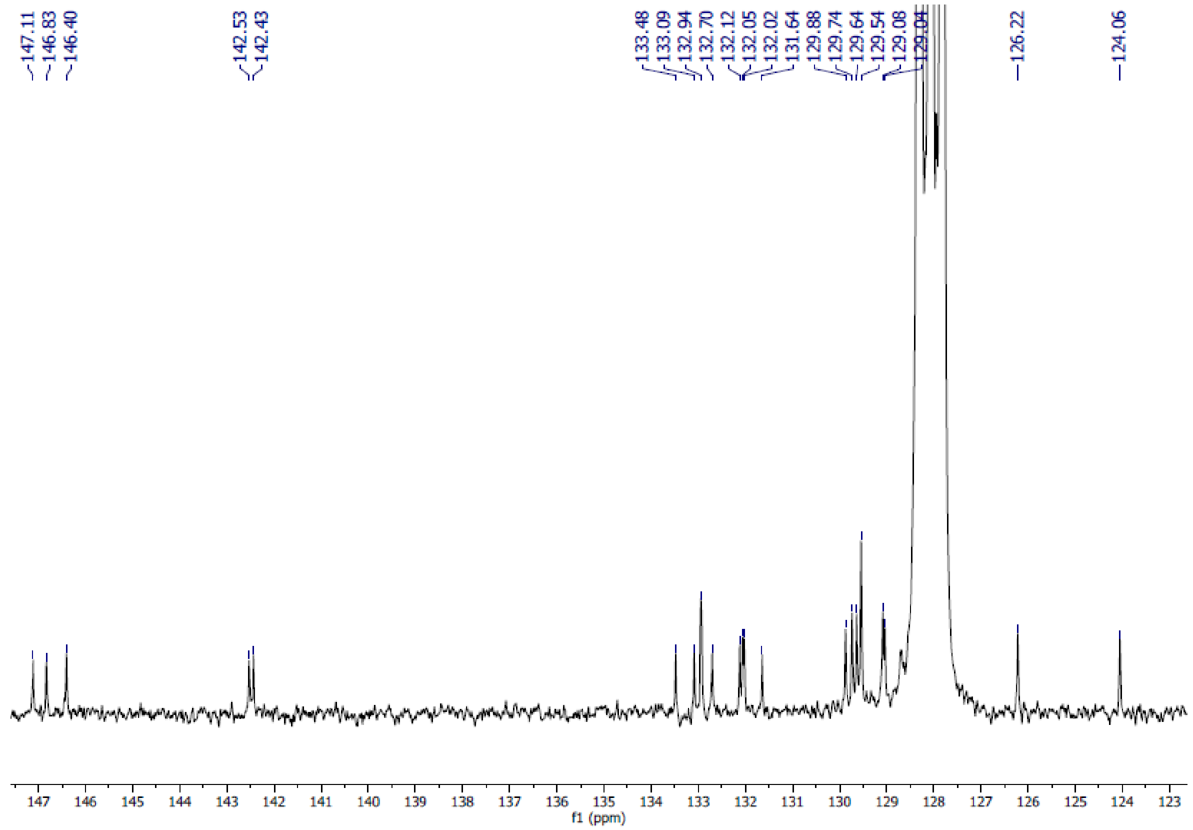

Figure S73. $^{13}C\{^{1}H\}$ NMR spectrum (100.5 MHz, C_6D_6, 298 K) of [{(^{MeMes}nacnac)Mg}(*μ*-OCH*t*Bu_2)(*μ*-CH_2-$^{MeMes-H}$nacnac)Mg] **12** (chemical shift range 123-148 ppm).

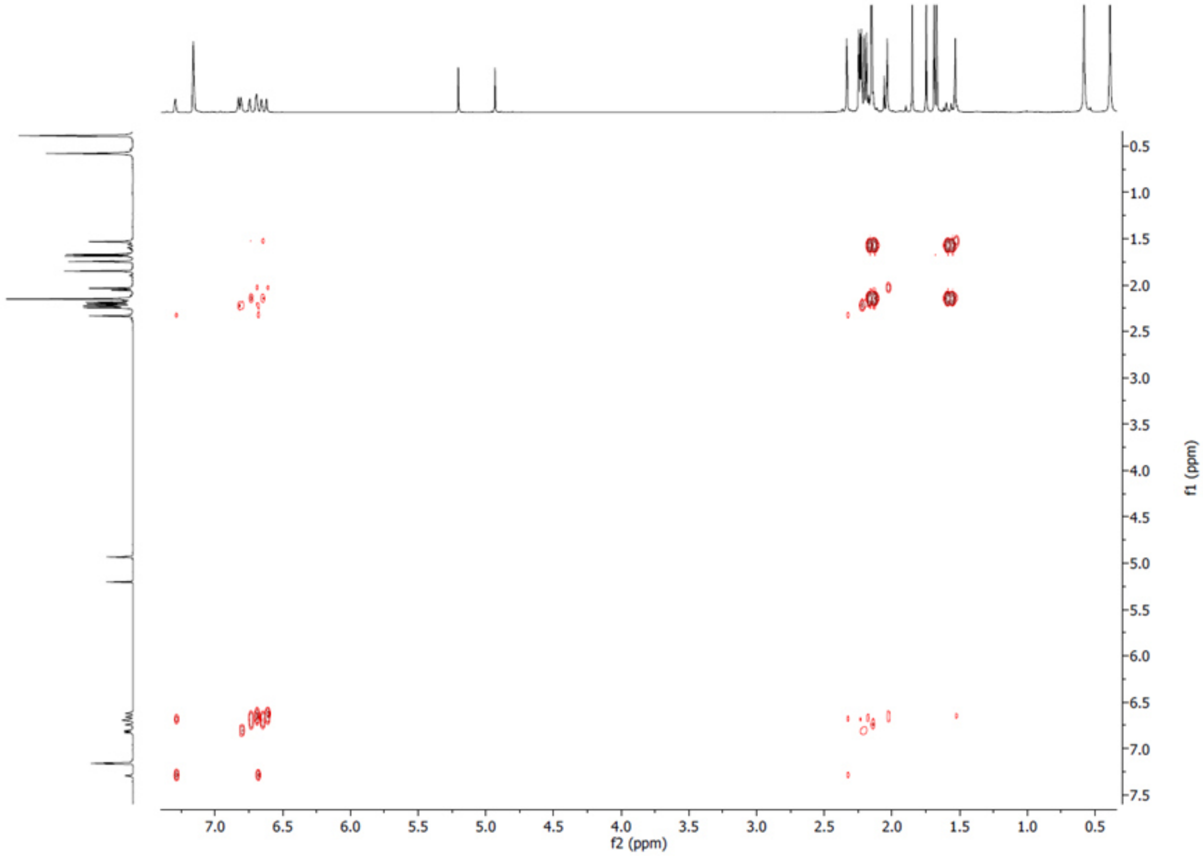

Figure S74. ^{1}H-^{1}H COSY NMR spectrum of [{(^{MeMes}nacnac)Mg}(*μ*-OCH*t*Bu_2)(*μ*-CH_2-$^{MeMes-H}$nacnac)Mg] **12**.

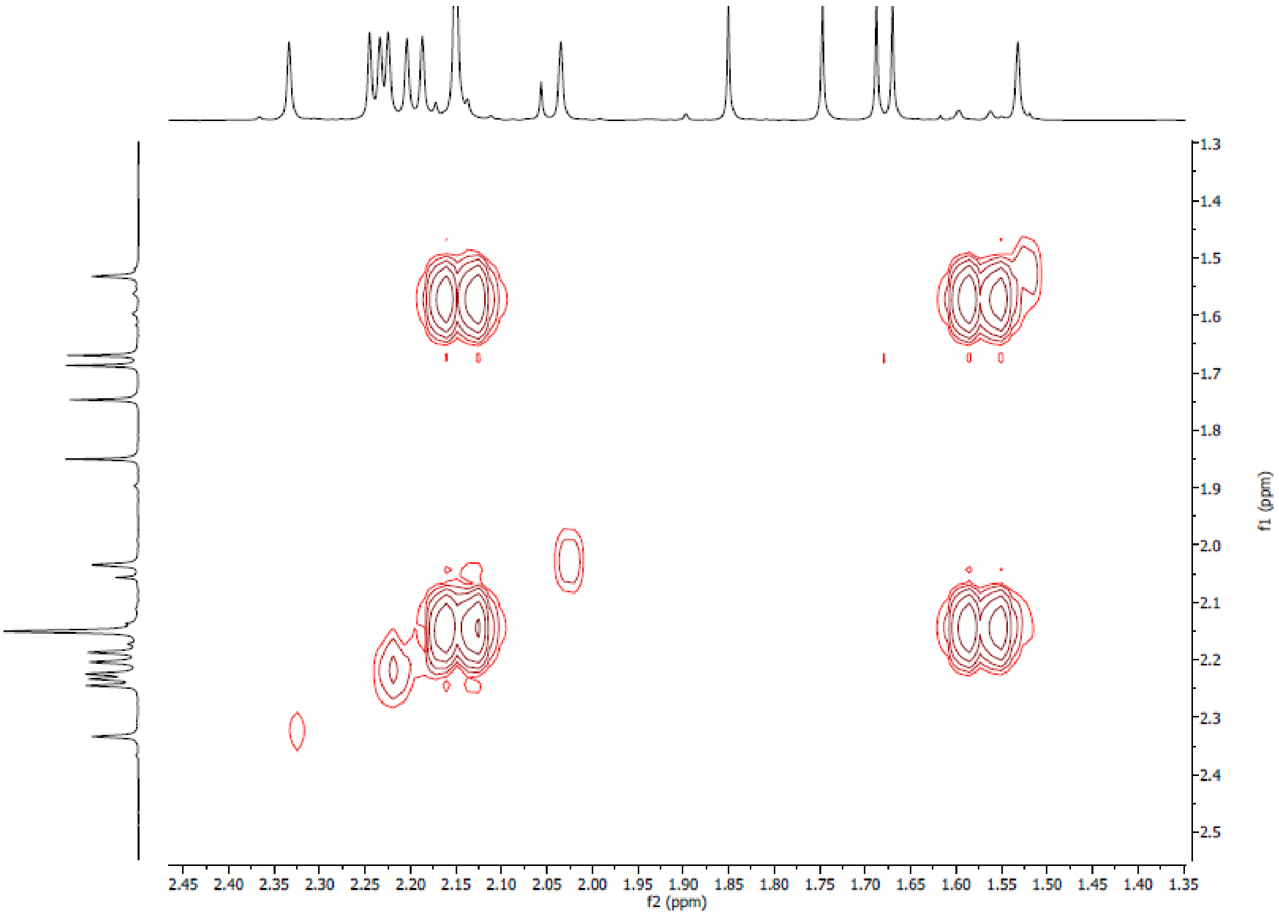

Figure S75. ^{1}H-^{1}H COSY NMR spectrum of [{(^{MeMes}nacnac)Mg}(μ-OCH*t*Bu$_2$)(μ-CH$_2$-$^{MeMes-H}$nacnac)Mg] **12** showing coupling of Ar-C*H*$_2$Mg protons (horizontal chemical shift range 1.35-2.45 ppm; vertical chemical shift range 1.3-2.5 ppm).

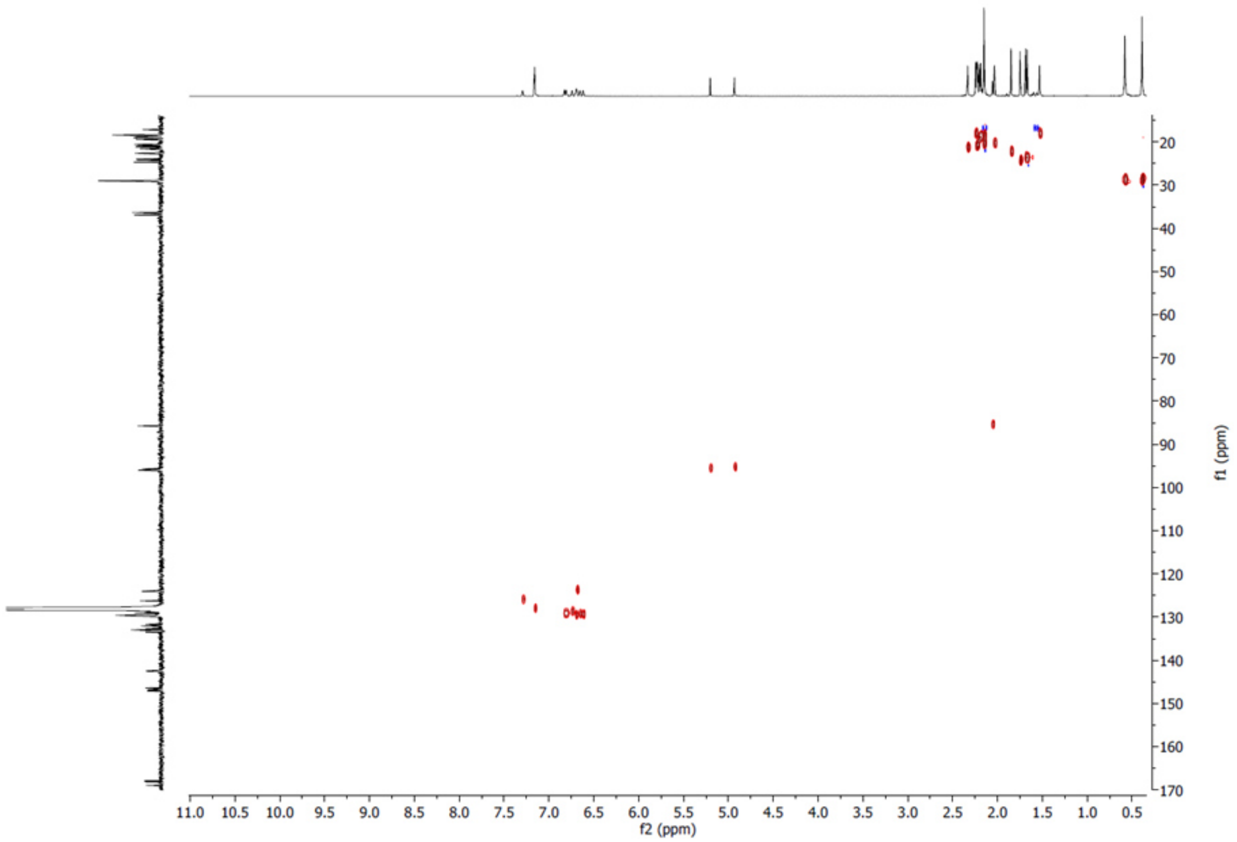

Figure S76. ^{1}H-^{13}C HSQC NMR spectrum of [{(^{MeMes}nacnac)Mg}(μ-OCHtBu$_2$)(μ-CH$_2$-$^{MeMes-H}$nacnac)Mg] **12**.

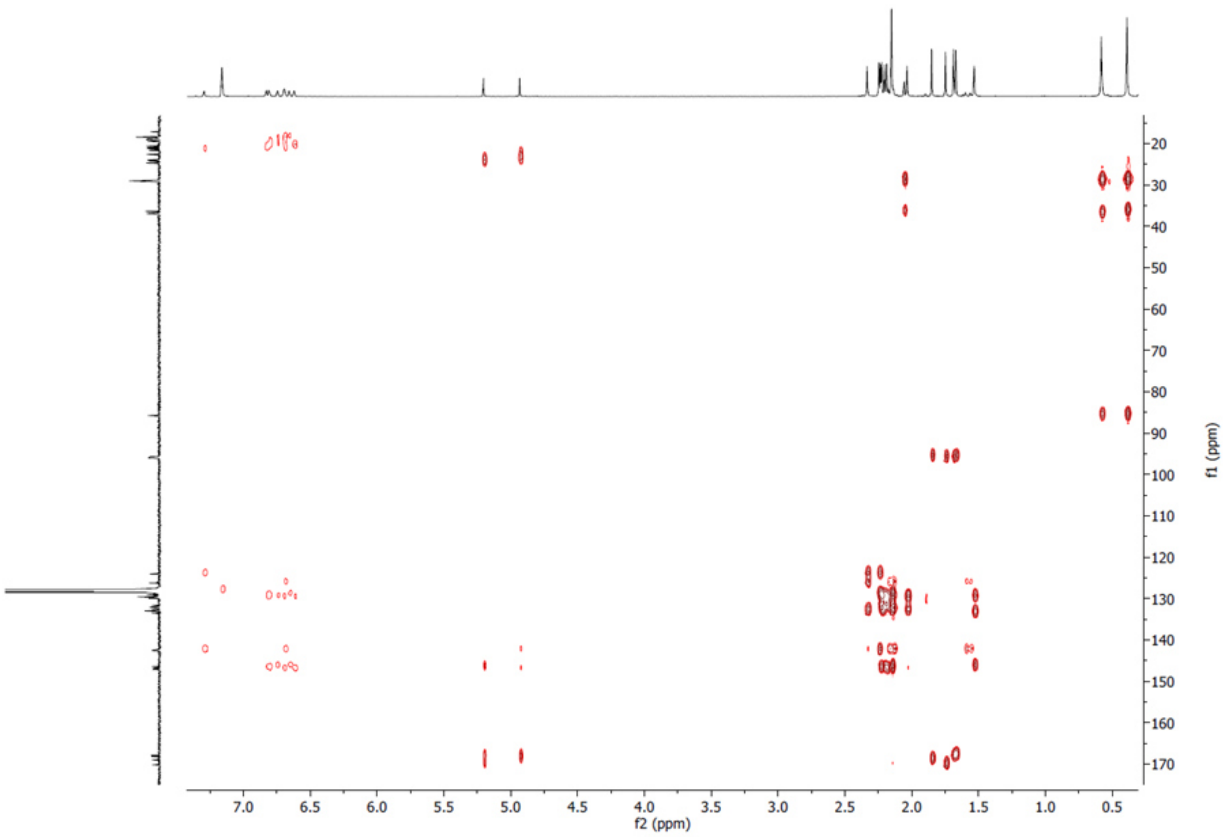

Figure S89. ^{1}H-^{13}C HMBC NMR spectrum of [{(^{MeMes}nacnac)Mg}(μ-OCHtBu$_2$)(μ-CH$_2$-$^{MeMes-H}$nacnac)Mg] **12**.

2.16 NMR spectra of [{(^{MeMes}nacnac)Mg}(μ-OAd)(μ-O(Ad)CH$_2$-$^{MeMes\text{-}H}$nacnac)Mg] 13

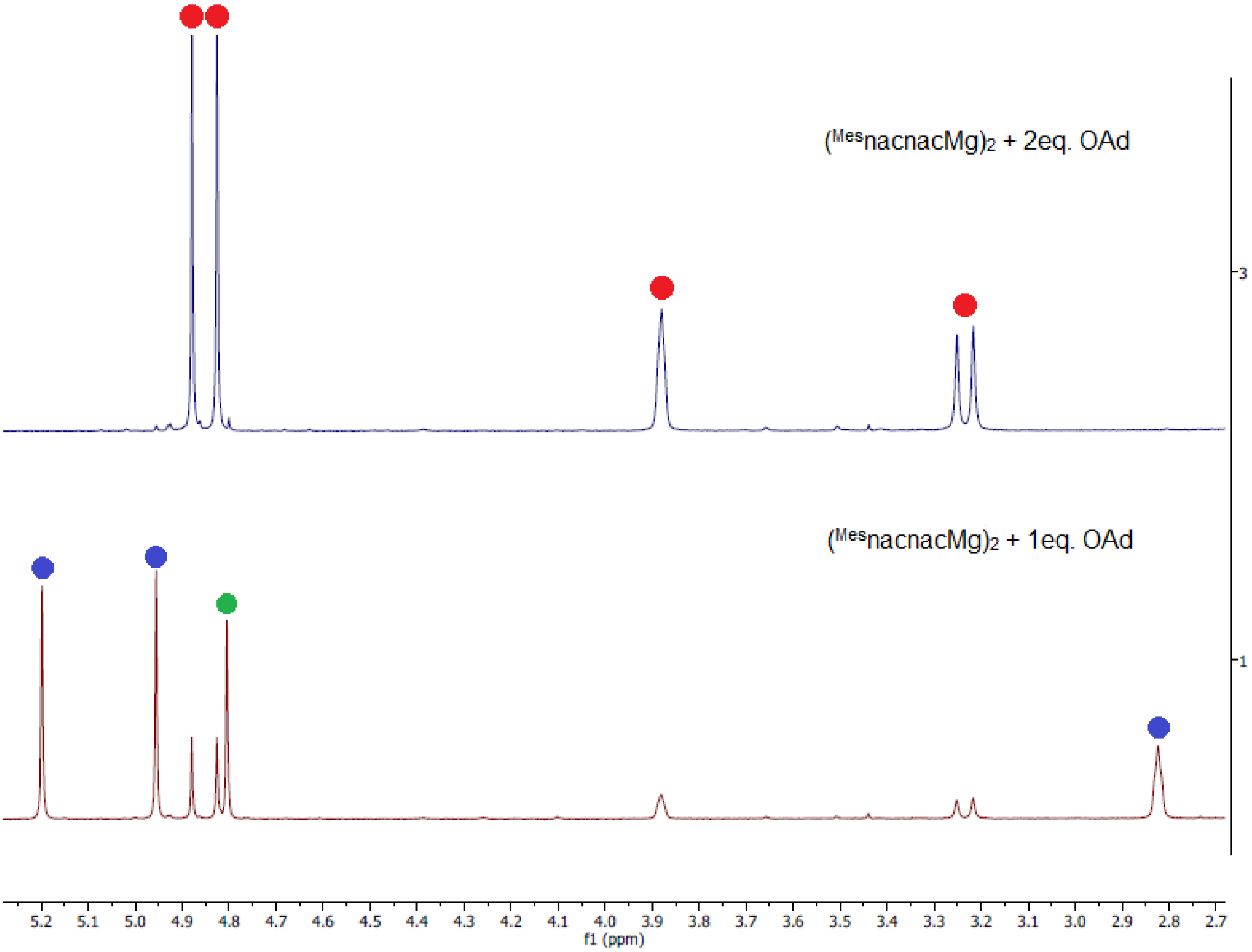

Figure S90. ^{1}H NMR spectrum (400.1 MHz, C_6D_6, 298 K) of [{(^{MeMes}nacnac)Mg}(μ-OAd)(μ-O(Ad)CH$_2$-$^{MeMes\text{-}H}$nacnac)Mg] **13** and [insert intermediate complex name] (chemical shift range 2.75-5.3 ppm). Red dots: [{(^{MeMes}nacnac)Mg}(μ-OAd)(μ-O(Ad)CH$_2$-$^{MeMes\text{-}H}$nacnac)Mg] **13**; blue dots: [{(^{MeMes}nacnac)Mg}(μ-OAdH)(μ-CH$_2$-$^{MeMes\text{-}H}$nacnac)Mg]; green dot: (^{Mes}nacnacMg)$_2$

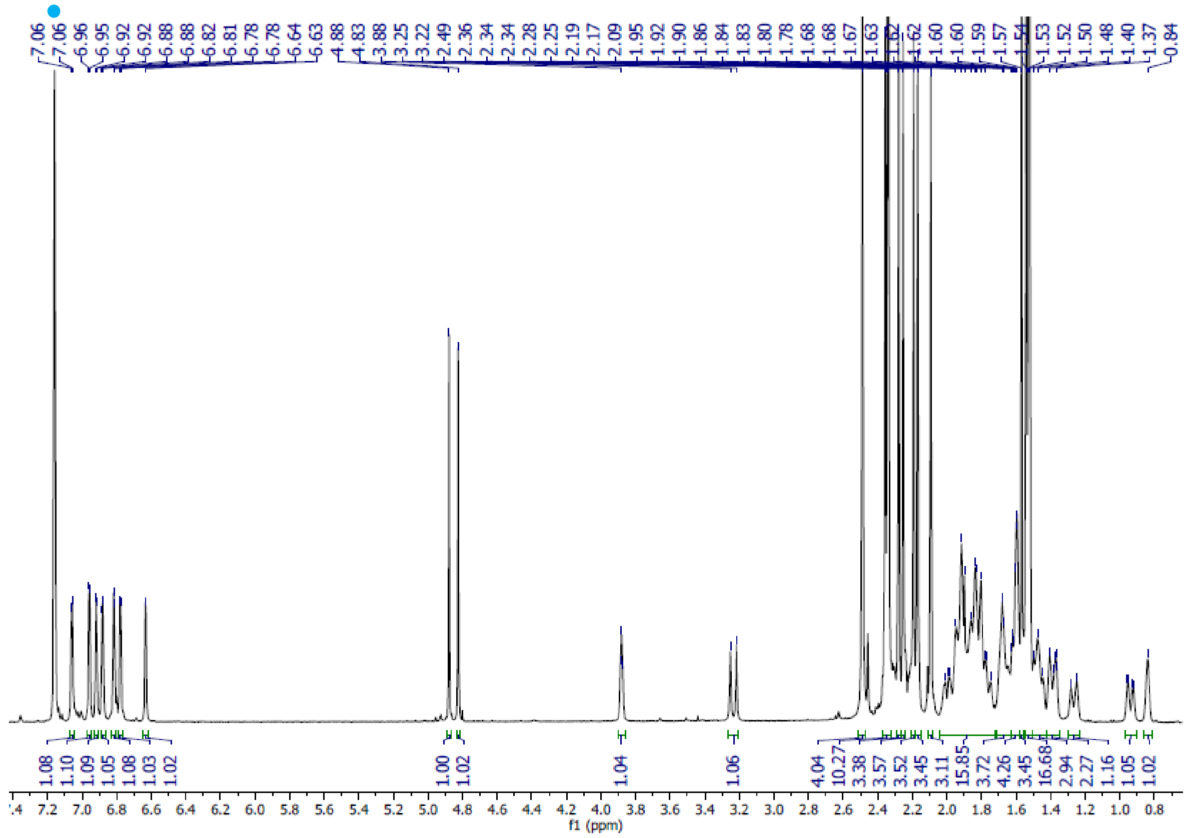

Figure S91. ^{1}H NMR spectrum (400.1 MHz, C_6D_6, 298 K) of [{(^{MeMes}nacnac)Mg}(μ-OAd)(μ-O(Ad)CH_2-$^{MeMes-H}$nacnac)Mg] **13**.

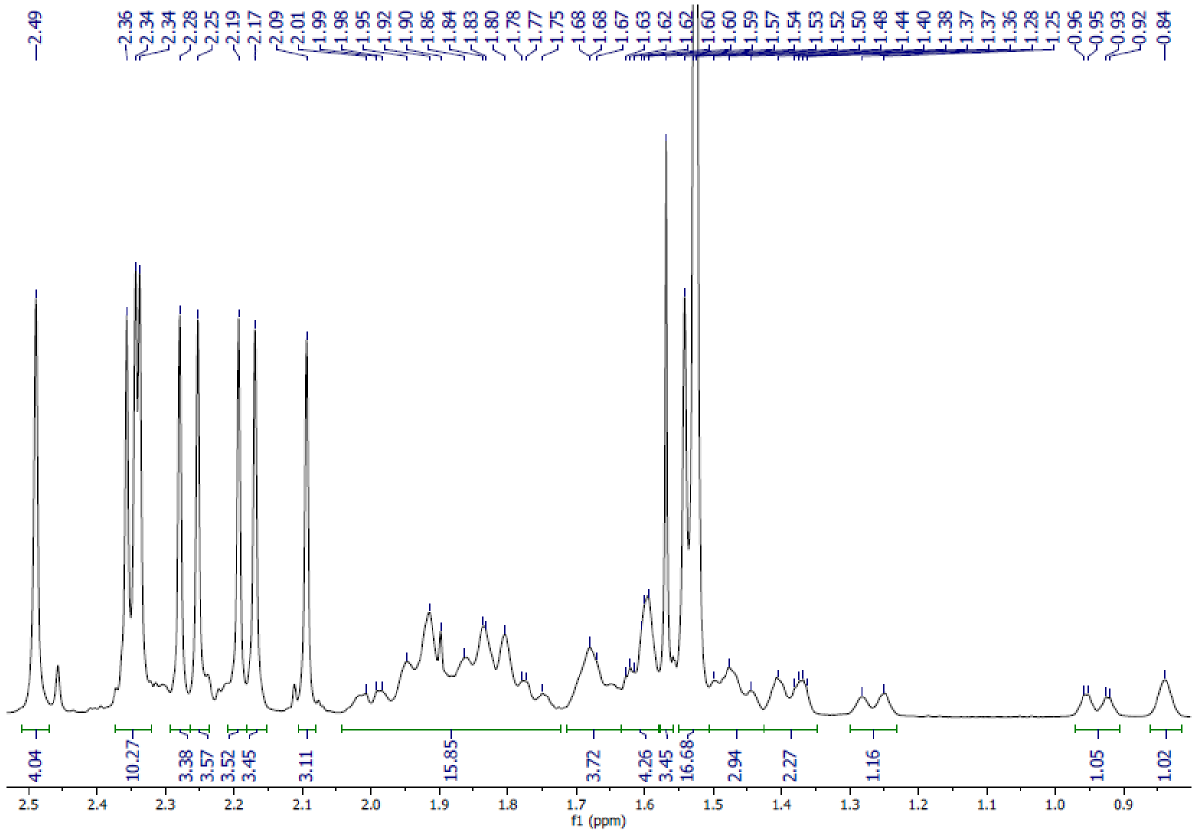

Figure S92. ^{1}H NMR spectrum (400.1 MHz, C_6D_6, 298 K) of [{(^{MeMes}nacnac)Mg}(μ-OAd)(μ-O(Ad)CH_2-$^{MeMes-H}$nacnac)Mg] **13** (chemical shift range 0.8-2.5 ppm).

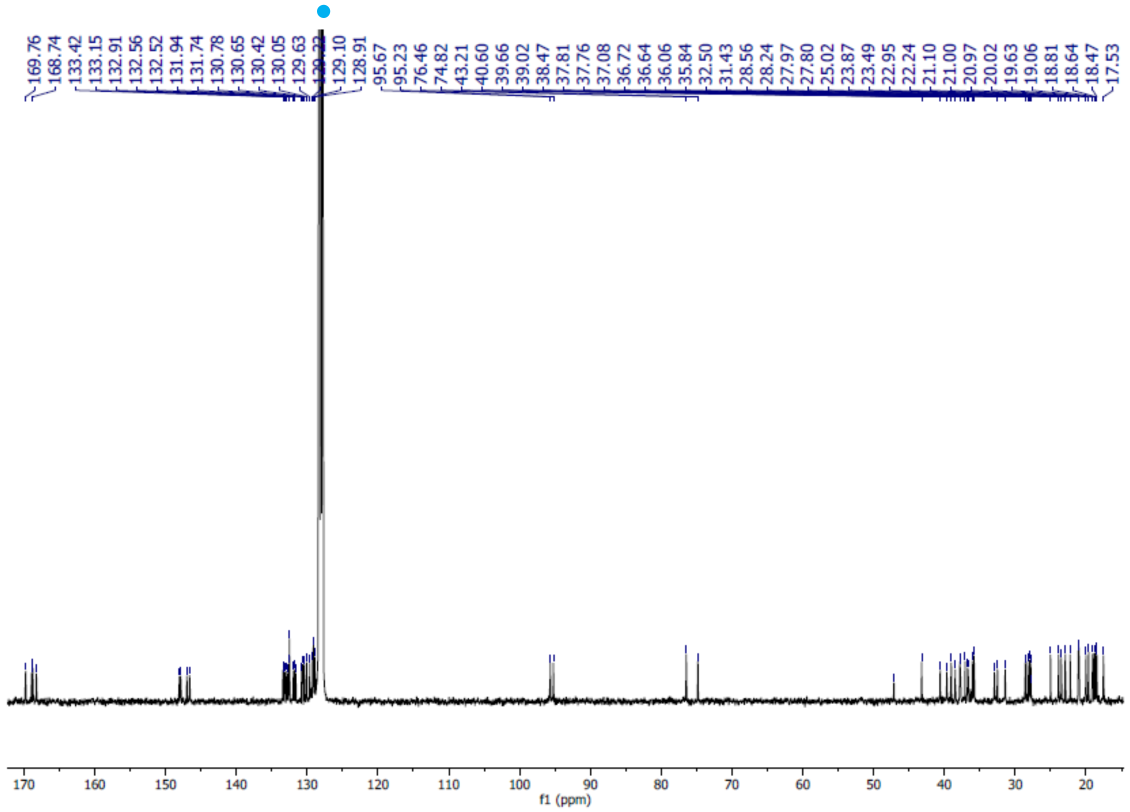

Figure S93. $^{13}C\{^{1}H\}$ NMR spectrum (100.5 MHz, C_6D_6, 298 K) of [{(^{MeMes}nacnac)Mg}(μ-OAd)(μ-O(Ad)CH_2-$^{MeMes-H}$nacnac)Mg] **13**.

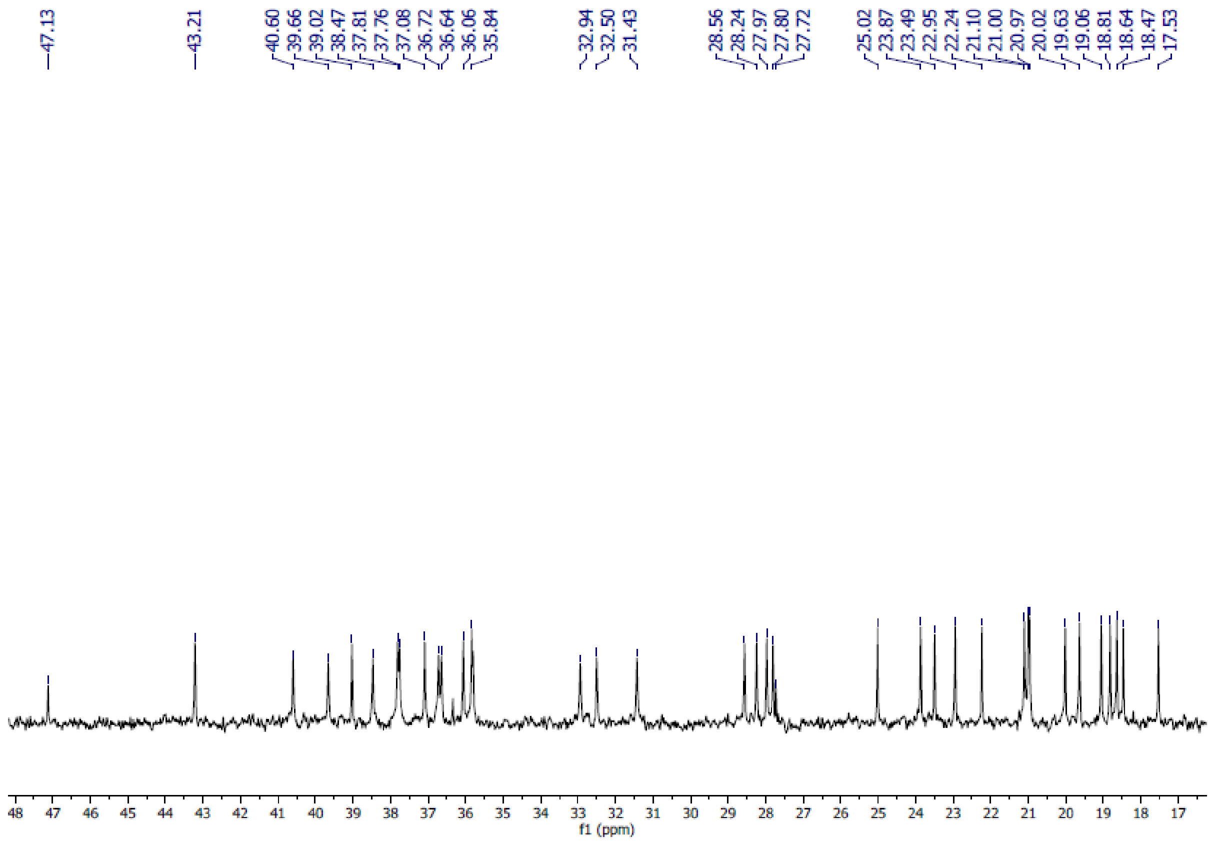

Figure S94. $^{13}C\{^{1}H\}$ NMR spectrum (100.5 MHz, C_6D_6, 298 K) of [{(^{MeMes}nacnac)Mg}(μ-OAd)(μ-O(Ad)CH_2-$^{MeMes-H}$nacnac)Mg] **13** (chemical shift range 17-48 ppm).

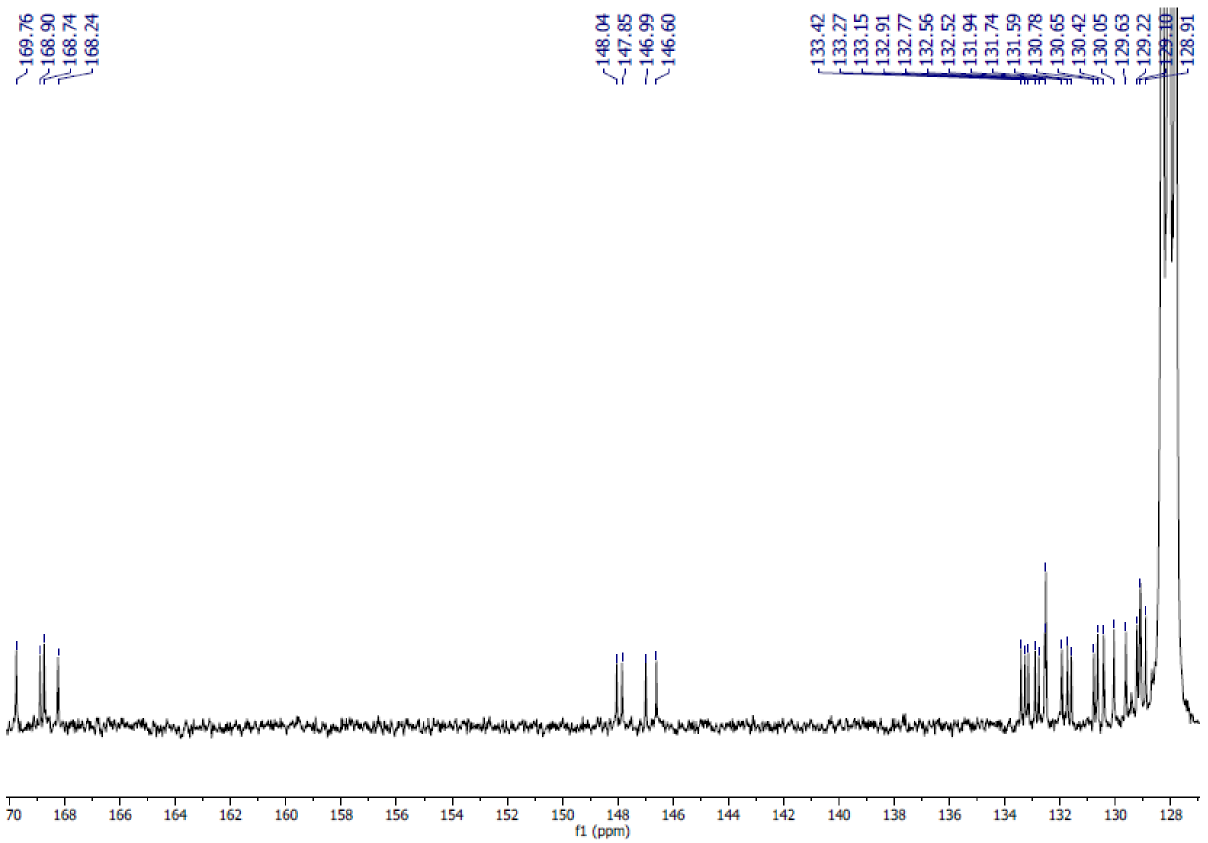

Figure S77. $^{13}C\{^{1}H\}$ NMR spectrum (100.5 MHz, C_6D_6, 298 K) of [{(^{MeMes}nacnac)Mg}(μ-OAd)(μ-O(Ad)CH_2-$^{MeMes\text{-}H}$nacnac)Mg] **13** (chemical shift range 128-170 ppm).

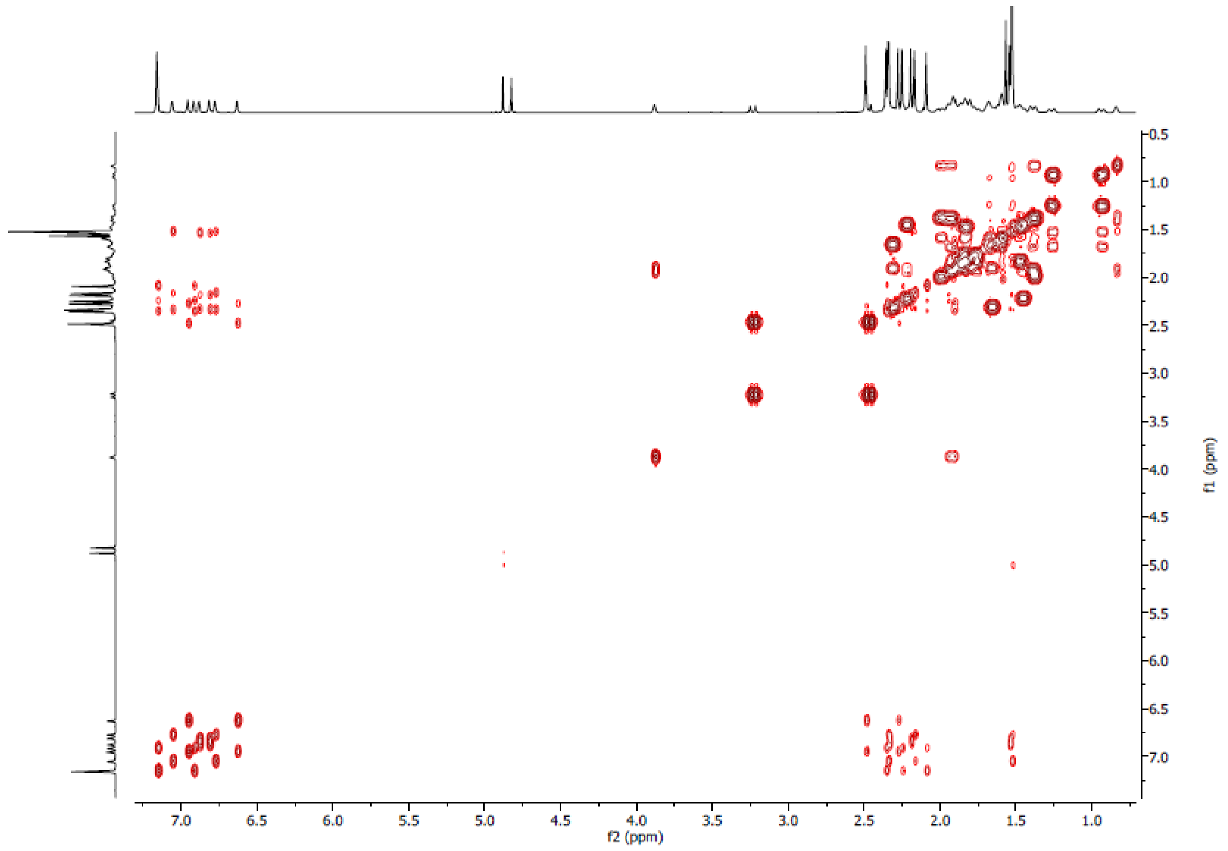

Figure S78. ^{1}H-^{1}H COSY NMR spectrum of [{(^{MeMes}nacnac)Mg}(μ-OAd)(μ-O(Ad)CH_2-$^{MeMes\text{-}H}$nacnac)Mg] **13**.

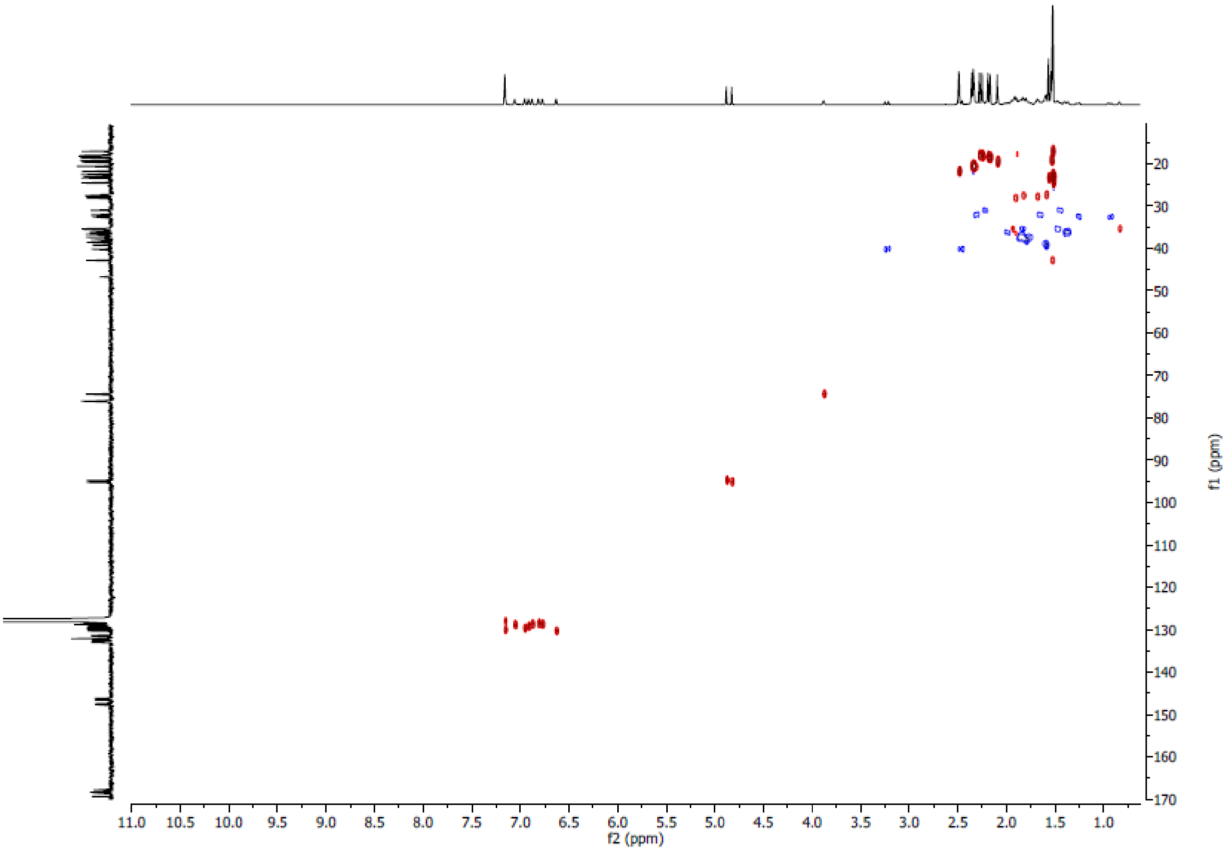

Figure S79. ^{1}H-^{13}C HSQC NMR spectrum of [{(^{MeMes}nacnac)Mg}(μ-OAd)(μ-O(Ad)CH$_2$-$^{MeMes-H}$nacnac)Mg] **13**.

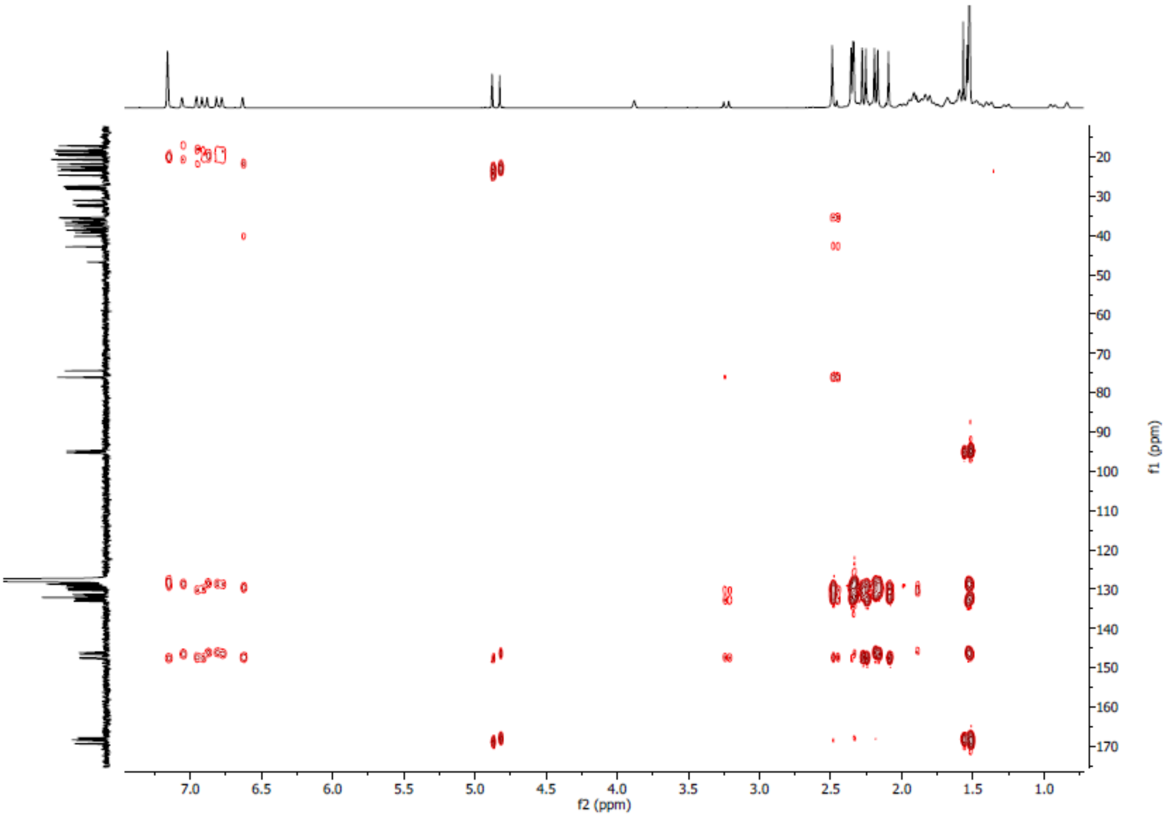

Figure S80. ^{1}H-^{13}C HMBC NMR spectrum of [{(^{MeMes}nacnac)Mg}(μ-OAd)(μ-O(Ad)CH$_2$-$^{MeMes-H}$nacnac)Mg] **13**.

3 X-ray crystallography

Suitable crystals were mounted in paratone oil and were measured using either a Rigaku FR-X Ultrahigh brilliance Microfocus RA generator/confocal optics with XtaLAB P200 diffractometer (Mo Kα radiation), a Rigaku SCX Mini (Mo Kα radiation), or a Rigaku MM-007HF High Brilliance RA generator/confocal optics with XtaLAB P200 diffractometer (Cu Kα radiation). Data for all compounds analysed were collected using CrystalClear.[3] Data were processed (including correction for Lorentz, polarization, and absorption) using either CrystalClear[3] or CrysAlisPro.[4] Structures were solved by a dual-space method (SHELXT-2018/2)[5] and refined by full-matrix least-squares against F^2 using SHELXL-2018/3.[6] All non-hydrogen atoms were refined anisotropically except in selected cases as described below. Hydrogen atoms were placed in calculated positions (riding model) except in selected cases as described below. All calculations were performed using CrystalStructure.[7] Details on individual crystal structure determinations and refinements are given below. Further experimental and refinement details are given in the CIF-files. CCDC 2158110-2158123 contains the supplementary crystallographic data for this paper. These data can be obtained free of charge via https://www.ccdc.cam.ac.uk/structures/.

Complex [{($^{i\text{PrDip}}$nacnac)Mg}$_2$] **4** could so far not be structurally characterised with sufficient quality, but the similar complexes [{(^{MeDip}nacnac)Mg}$_2$][8] and [{($^{t\text{BuDip}}$nacnac)Mg}$_2$][1] have been structurally characterised previously and are highly similar. Although complex [($^{i\text{PrDip}}$nacnac)Mg(OAdH)] **8** was obtained repeatedly, no molecular structure has so far been determined, but comparison with other species in here strongly suggest that the complex is monomeric.

Table S1 Crystallographic data.

Compound reference	**1**	**1'**	**2**·2 C_6H_6	LMgO*n*Bu	**3**·2 C_7H_8
Chemical formula	$C_{33}H_{50}N_2$	$C_{33}H_{50}N_2$	$C_{49}H_{70}MgN_2$	$C_{43}H_{72}MgN_2O$	$C_{80}H_{114}I_2Mg_2N_4$
Formula weight	474.77	474.77	711.38	657.36	1434.23
Temperature/K	173	173	173	93	173
Radiation type	Mo	Cu	Mo	Mo	Mo
Wavelength/Å	0.71075	1.54184	0.71075	0.71075	0.71075
Crystal system	orthorhombic	orthorhombic	orthorhombic	triclinic	monoclinic
Space group	*Fdd2*	*Fdd2*	*Pnma*	*P*-1	$P2_1/c$
a/Å	17.0179(12)	16.9852(5)	16.9412(19)	12.1464(14)	14.2924(19)
b/Å	42.405(3)	42.3795(13)	11.1598(10)	12.9556(12)	25.706(3)
c/Å	8.3891(6)	8.3707(2)	24.343(3)	14.5770(10)	10.8234(16)
α/°	90	90	90	66.436(7)	90
β/°	90	90	90	77.284(9)	103.041(4)
γ/°	90	90	90	87.734(10)	90
Unit cell volume/Å^3	6053.9(7)	6025.4(3)	4602.3(9)	2048.0(4)	3874.0(9)
No. of formula units per unit cell, Z	8	8	4	2	2
Density (calc)/ Mg/m^3	1.042	1.047	1.027	1.066	1.229
Absorption coefficient, μ/mm^{-1}	0.059	0.444	0.070	0.076	0.871
F(000)	2096	2096	1560	728	1504
Theta range/°	1.921 to 25.381	4.173 to 75.420	1.464 to 25.370	2.051 to 25.369	1.463 to 25.396
Reflections collected	32457	16598	67212	44855	51061
Independent reflections	2764	2737	4452	7508	7102
R_{int}	0.0308	0.0222	0.0209	0.0720	0.0886
Completeness (to theta)/%	99.3 (max)	98.1 (max)	99.8 (max)	99.7 (max)	99.6 (max)
Data / restraints / parameter	2764 / 2 / 169	2737 / 2 / 170	4452 / 6 / 293	7508 / 0 / 439	7102 / 0 / 410
Goodness of fit on F^2	1.046	1.055	1.056	1.056	0.803
Final R_1 values ($I > 2\sigma(I)$)	0.0341	0.0383	0.0394	0.0751	0.0446
Final $wR(F^2)$ values ($I > 2\sigma(I)$)	0.0951	0.0988	0.1126	0.2205	0.1083
Final R_1 values (all data)	0.0348	0.0384	0.0451	0.0859	0.0714
Final $wR(F^2)$ values (all data)	0.0957	0.0989	0.1172	0.2323	0.1330
Largest diff. peak and hole/e·Å^{-3}	0.12 and -0.14	0.18 and -0.16	0.262 and -0.191	0.86 and -0.52	0.87 and -0.473
Absolute structure parameter	0.0(5)	-0.20(18)	-	-	-
CCDC number	2158111	2158110	2158116	2158122	2158118

L denotes ^{iPrDip}nacnac

Table S1 continued Crystallographic data.

Compound reference	**3**·4 C_6H_6	LMgI(THF)	**5**	**6**·C_6H_6	$(LMgTHF)_2O\cdot C_6H_6$
Chemical formula	$C_{90}H_{122}I_2Mg_2N_4$	$C_{43}H_{63}IMgN_2O$	$C_{66}H_{100}Mg_2N_4$	$C_{82}H_{118}Mg_2N_4O$	$C_{80}H_{120}Mg_2N_4O_3$
Formula weight	1562.40	775.19	998.15	1224.42	1234.41
Temperature/K	173	173	125	173	125
Radiation type	Mo	Mo	Cu	Mo	Cu
Wavelength/Å	0.71075	0.71075	1.54184	0.71075	1.54184
Crystal system	monoclinic	monoclinic	orthorhombic	monoclinic	monoclinic
Space group	$P2_1/n$	$P2_1/c$	$C222_1$	$P2_1/c$	$P2_1/n$
a/Å	15.9100(15)	12.3655(8)	16.23500(17)	15.576(4)	12.74740(10)
b/Å	9.7639(5)	9.9726(7)	16.54720(17)	19.302(5)	14.20940(10)
c/Å	27.790(2)	34.742(2)	46.6005(5)	25.015(6)	20.81670(10)
α/°	90	90	90	90	90
β/°	100.771(3)	92.480(17)	90	95.386(5)	92.3860(10)
γ/°	90	90	90	90	90
Unit cell volume/Å^3	4240.9(5)	4280.2(5)	12518.9(2)	7488(3)	3767.32(4)
No. of formula units per unit cell, Z	2	4	8	4	2
Density (calc)/ Mg/m^3	1.223	1.203	1059	1.086	1.088
Absorption coefficient, μ/mm^{-1}	0.801	0.795	0.634	0.078	0.642
F(000)	1640	1632	4384	2680	1352
Theta range/°	1.492 to 25.351	1.648 to 27.533	3.814 to 75.506	1.335 to 25.243	3.767 to 75.458
Reflections collected	56541	33939	67884	181390	43320
Independent reflections	7753	9617	12538	13522	7599
R_{int}	0.0214	0.1033	0.0168	0.1440	0.0185
Completeness (to theta)/%	99.8 (max)	99.0 (25.242°)	98.3 (max)	99.9 (max)	99.7 (67.684°)
Data / restraints / parameter	7753 / 0 / 442	9617 / 0 / 433	12538 / 4 / 658	13522 / 42 / 940	7599 / 10 / 453
Goodness of fit on F^2	1.041	1.110	1.075	1.100	1.039
Final R_1 values ($I > 2\sigma(I)$)	0.0317	0.0676	0.0306	0.0909	0.0395
Final $wR(F^2)$ values ($I > 2\sigma(I)$)	0.0911	0.1297	0.0818	0.1607	0.1079
Final R_1 values (all data)	0.0335	0.1143	0.0307	0.1690	0.0409
Final $wR(F^2)$ values (all data)	0.0926	0.1483	0.0820	0.1963	0.1092
Largest diff. peak and hole/e·Å^{-3}	1.00 and -0.58	1.04 and -0.80	0.38 and -0.21	0.343 and -0.263	0.373 and -0.330
Absolute structure parameter	-	-	0.002(9)	-	-
CCDC number	2158117	2158121	2158119	2158120	2158123

L denotes ^{iPrDip}nacnac

Table S1 continued Crystallographic data.

Compound reference	**10**	**11**·0.5 C_6H_6	**12**	**13**
Chemical formula	$C_{40}H_{62}MgN_2O$	$C_{45}H_{71}MgN_2O$	$C_{55}H_{76}Mg_2N_4O$	$C_{66}H_{86}Mg_2N_4O_2$
Formula weight	611.25	680.37	857.81	1016.00
Temperature/K	93	93	173	125
Radiation type	Mo	Mo	Mo	Cu
Wavelength/Å	0.71075	0.71073	0.71075	1.54184
Crystal system	triclinic	triclinic	monoclinic	monoclinic
Space group	*P*-1	*P*-1	$P2_1/c$	$P2_1/c$
a/Å	10.617(4)	9.8100(2)	13.8663(16)	13.3421(3)
b/Å	12.201(5)	10.2482(2)	16.471(2)	16.3669(3)
c/Å	16.321(7)	21.6956(6)	22.411(3)	26.7682(5)
α/°	98.437(7)	81.568(2)	90	90
β/°	102.186(7)	88.7548(19)	93.277(4)	96.610(2)
γ/°	107.801(5)	85.5449(19)	90	90
Unit cell volume/$Å^3$	1916.3(14)	2150.95(9)	5110.1(11)	5806.5(2)
No. of formula units per unit cell, *Z*	2	2	4	4
Density (calc)/ Mg/m^3	1.059	1.050	1.114	1.162
Absorption coefficient, μ/mm^{-1}	0.077	0.074	0.088	0.725
F(000)	672	750	1860	2200
Theta range/°	2.622 to 25.355	2.015 to 28.600	1.820 to 25.387	3.171 to 76.559
Reflections collected	78471	24819	56826	59891
Independent reflections	6999	9124	9330	11680
R_{int}	0.0957	0.0326	0.0511	0.0605
Completeness (to theta)/%	99.6 (max)	99.3 (25.242°)	99.4 (max)	99.6 (67.684)
Data / restraints / parameter	6999 / 0 / 413	11680 / 12 / 724	9330 / 0 / 588	12172 / 4484 / 1353
Goodness of fit on F^2	0.989	1.158	1.088	1.260
Final R_1 values ($I > 2\sigma(I)$)	0.0441	0.0600	0.0485	0.1365
Final $wR(F^2)$ values ($I > 2\sigma(I)$)	0.1344	0.1770	0.1268	0.3731
Final R_1 values (all data)	0.0493	0.0660	0.0620	0.2673
Final $wR(F^2)$ values (all data)	0.1379	0.1931	0.1419	0.4485
Largest diff. peak and hole/e·$Å^{-3}$	0.31 and -0.22	0.353 and -0.390	0.309 and -0.225	0.993 and -0.835
Absolute structure parameter	-	-	-	-
CCDC number	2158112	2158113	2158114	2158115

L denotes ^{iPrDip}nacnac

Compound 1

The molecular structure of ^{iPrDip}nacnacH, **1**, see Figure S99, crystallised with half a molecule in the asymmetric unit and two similar datasets are provided. The NH hydrogen atom was found and freely refined.

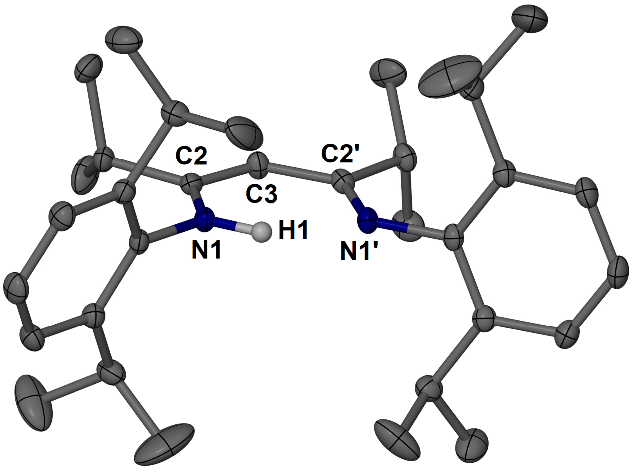

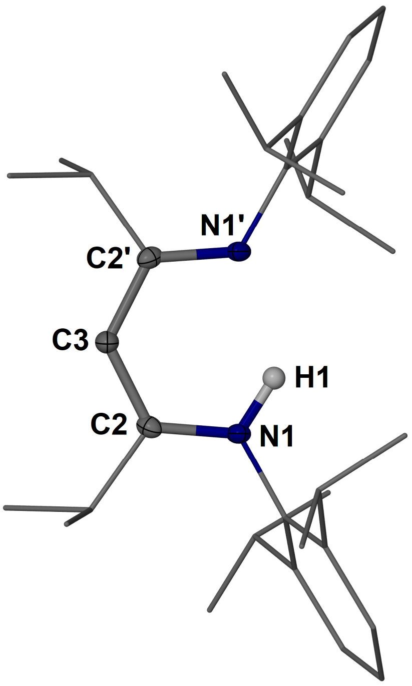

Figure S99. Molecular structure of **1 (Mo)** (30% thermal ellipsoids), top. Only one of the two 50% NH positions is shown. Selected bond lengths and angles: N1-C2 1.330(2), N1-C4 1.430(2), N1-H1 0.978(7), C2-C3 1.402(2); C2-C3-C2' 127.0(2), C2-N1-C4 123.54(15). Molecular structure of **1 (Cu)** (30% thermal ellipsoids), bottom. Only one of the two 50% NH positions is shown. Dip and *i*Pr groups are shown as wireframe. Selected bond lengths and angles: N1-C2 1.327(2), N1-C4 1.431(2), N1-H1 0.978(7), C2-C3 1.406(2); C2-C3-C2' 126.7(2), C2-N1-C4 123.46(15).

Complex 2

The molecular structure of [(^{iPrDip}nacnac)Mg*n*Bu]·2 C_6H_6, **2**·2 C_6H_6, see Figure S100, crystallised with half a molecule in the asymmetric unit having a mirror plane through the nacnacMg heterocycle. The outer three carbon atoms of the *n*Bu group are severely disordered and were modelled with two positions of these atoms in the asymmetric unit using geometry restraints. The independent hydrogen atom on C24 in the asymmetric unit was found and freely refined.

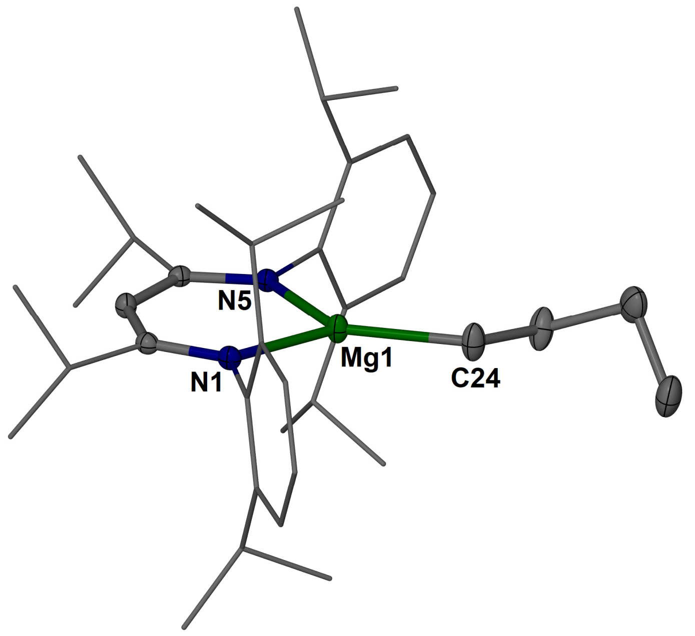

Figure S100. Molecular structure of **2**·2 C_6H_6 (30% thermal ellipsoids). Solvent molecules and hydrogen atoms not shown. Dip and *i*Pr groups shown as wireframe. Selected bond lengths and angles: Mg1-N5 2.0154(13), Mg1-N1 2.0272(13), Mg1-C24 2.1025(19), N1-C2 1.3349(19), N1-C6 1.4436(18), N5-C4 1.3316(19), N5-C17 1.4410(18), C2-C3 1.402(2), C3-C4 1.402(2); N5-Mg1-N1 93.57(5), N5-Mg1-C24 132.05(6), N1-Mg1-C24 134.39(6); sum of angles around Mg1: 360.01(17).

[{(^{iPrDip}nacnac)Mg(μ-OnBu)}$_2$]

Few crystals of complex [{(^{iPrDip}nacnac)Mg(μ-OnBu)}$_2$] were obtained in one instance during a synthesis of [(^{iPrDip}nacnac)MgnBu] **2**, presumably from a contamination of nBuMg(OnBu) in Mg(nBu)$_2$ due to exposure with traces of air. The molecular structure of [{(^{iPrDip}nacnac)Mg(μ-OnBu)}$_2$]·2 C_6H_{14}, see Figure S101, crystallized with half a molecule in the asymmetric unit.

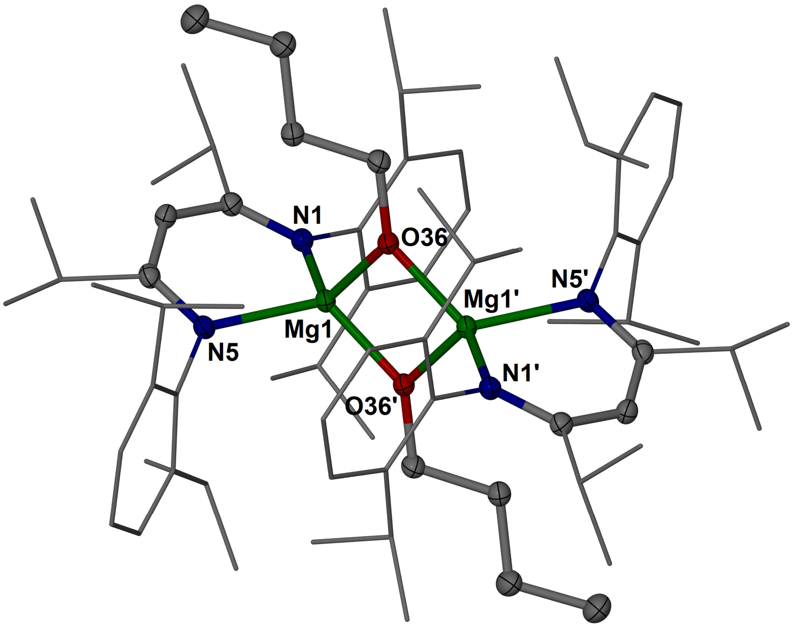

Figure S101. Molecular structure of [{(^{iPrDip}nacnac)Mg(μ-OnBu)}$_2$]·2 C_6H_{14} (30% thermal ellipsoids). Solvent molecule and hydrogen atoms not shown. Dip and iPr groups are shown as wireframe. Selected bond lengths and angles: Mg1-O36 1.9647(17), Mg1-O36' 1.9846(17), Mg1-N5 2.092(2), Mg1-N1 2.123(2); O36-Mg1-O36' 80.94(7), O36-Mg1-N5 117.51(8), O36-Mg1-N5' 120.78(8), O36-Mg1-N1 130.71(8), O36-Mg1-N1' 117.49(7), N5-Mg1-N1 92.74(8).

Complex 3

Two similar solvates of $[\{(^{iPrDip}\text{nacnac})\text{Mg}(\mu\text{-I})\}_2]$ **3**, Figure S102, were characterised; $[\{(^{iPrDip}\text{nacnac})\text{Mg}(\mu\text{-I})\}_2]\cdot 2\ C_7H_8$, **3**$\cdot 2\ C_7H_8$, and $[\{(^{iPrDip}\text{nacnac})\text{Mg}(\mu\text{-I})\}_2]\cdot 4\ C_6H_6$, **3**$\cdot 4\ C_6H_6$. Both crystallised with half a molecule in the asymmetric unit.

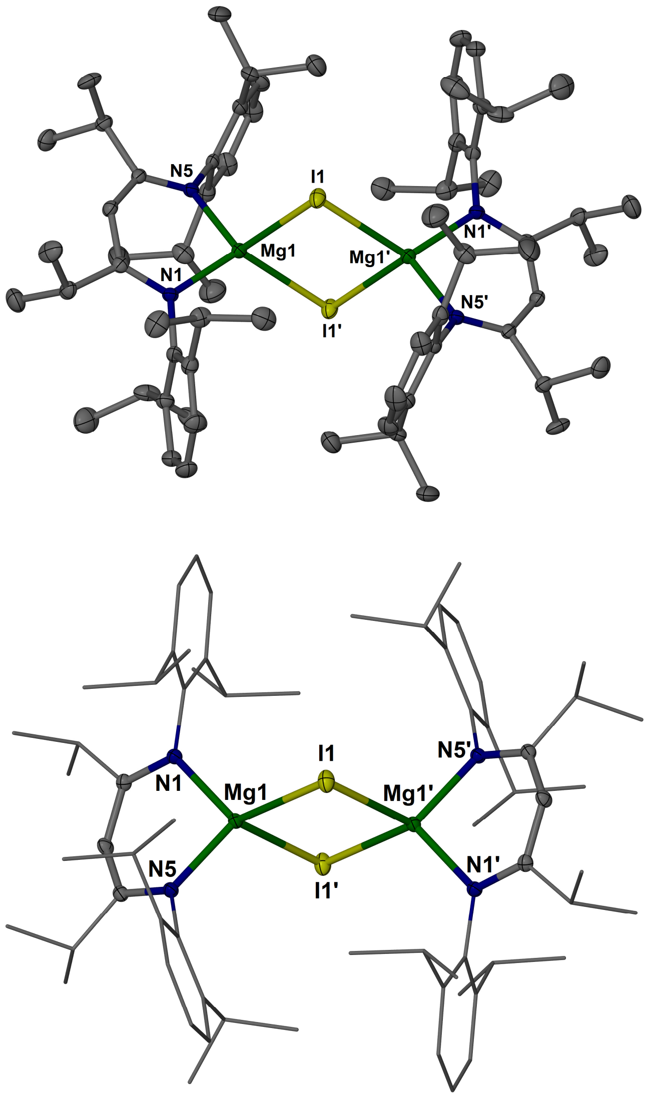

Figure S102. Molecular structure of **3**$\cdot 2\ C_7H_8$ (30% thermal ellipsoids), top. Solvent molecules and hydrogen atoms not shown. Selected bond lengths and angles: I1-Mg1 2.7796(14), I1-Mg1' 2.7928(14), Mg1-N1 2.038(4), Mg1-N5 2.040(3); Mg1-I1-Mg1' 88.02(4), N1-Mg1-N5 97.58(14), N1-Mg1-I1 121.66(11), N5-Mg1-I1 118.88(11), N1-Mg1-I1' 116.09(11), N5-Mg1-I1' 111.77(11), I1-Mg1-I1' 91.98(4). Molecular structure of **3**$\cdot 4\ C_6H_6$ (30% thermal ellipsoids), bottom. Solvent molecules and hydrogen atoms not shown. Dip and *i*Pr groups are shown as wireframe. Selected bond lengths and angles: I1-Mg1 2.7611(7), I1-Mg1' 2.7837(8), Mg1-N5 2.0281(19), Mg1-N1 2.0305(19); Mg1-I1-Mg1' 83.63(2), N5-Mg1-N1 99.01(8), N5-Mg1-I1 120.29(6), N1-Mg1-I1 119.93(6), N5-Mg1-I1' 111.59(6), N1-Mg1-I1' 109.87(6), I1-Mg1-I1' 96.38(2).

[(^{iPrDip}nacnac)Mg(THF)I]

Crystals of [(^{iPrDip}nacnac)Mg(THF)I] were obtained from **3** with the addition of THF. The molecular structure of [(^{iPrDip}nacnac)Mg(THF)I] is shown in Figure S103.

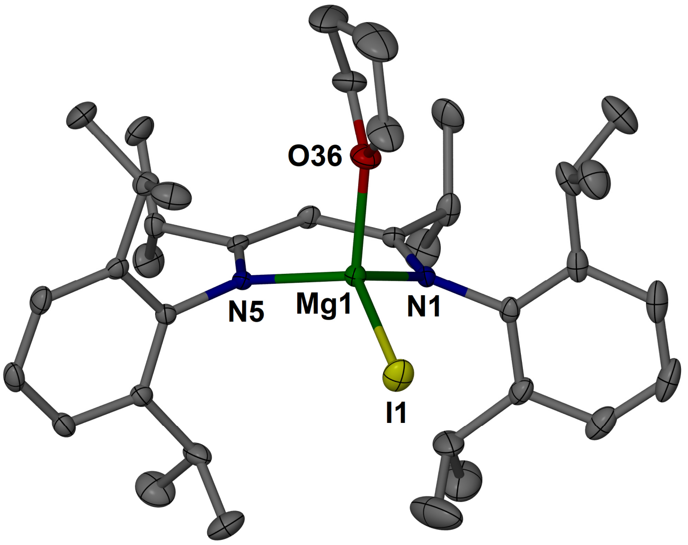

Figure S103. Molecular structure of [(^{iPrDip}nacnac)Mg(THF)I] (30% thermal ellipsoids). Selected bond lengths and angles: I1-Mg1 2.6512(14), Mg1-N1 2.035(4), Mg1-N5 2.037(4), Mg1-O36 2.040(4), Mg1-C2 2.869(4); N1-Mg1-N5 96.74(15), N1-Mg1-O36 104.89(15), N5-Mg1-O36 105.56(15), N1-Mg1-I1 118.47(11), N5-Mg1-I1 123.55(11), O36-Mg1-I1 105.80(12).

Complex 5

The molecular structure of [{(^{iPrDip}nacnac)Mg(μ-H)}$_2$] **5** crystallised with two independent half molecules in the asymmetric unit. Both molecules show a very similar molecular structure and metrical parameters; one is shown in Figure S104. The hydride units were found and refined using geometry restraints.

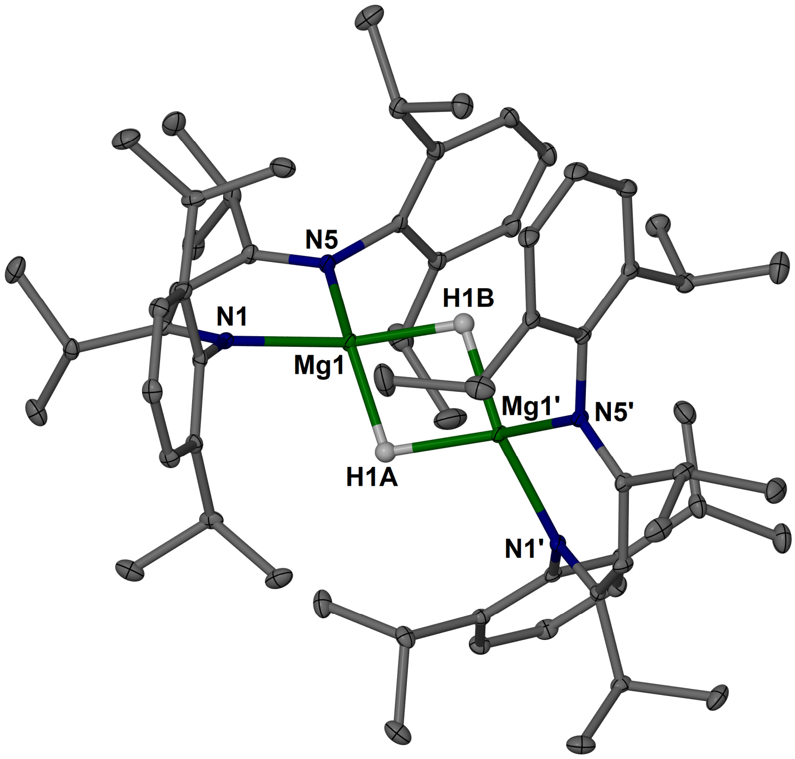

Figure S104. Molecular structure of **5** (30% thermal ellipsoids). Selected bond lengths and angles: Molecule 1: Mg1-N5 2.0543(14), Mg1-N1 2.0601(14), Mg1···Mg1' 2.8489(10), Mg1-H1A 1.849(7), Mg1-H1B 1.853(7); N5-Mg1-N1 93.90(5), N5-Mg1-H1A 139.67(6), N1-Mg1-H1A 104.2(2), N5-Mg1-H1B 102.6(2), N1-Mg1-H1B 149.58(6), H1A-Mg1-H1B 79.3(4); Molecule 2: Mg41-N45 2.0523(14), Mg41-N41 2.0562(14), Mg41···Mg41' 2.8482(10) 2, Mg41-H41B 1.852(7), Mg41-H41A 1.846(7); N45-Mg41-N41 93.63(6), N45-Mg41-H41B 102.5(2), N41-Mg41-H41B 140.93(7), N45-Mg41-H41A 146.35(6), N41-Mg41-H41A 106.1(2), H41B-Mg41-H41A 79.3(4).

Complex 6

The molecular structure of $[\{(^{iPrDip}nacnac)Mg\}_2(\mu\text{-OAd})]\cdot C_6H_6$, **6**·$C_6H_6$, see Figure S105, crystallised with a full molecule in the asymmetric unit. Some outer ligand substituents are relatively poorly ordered, and two Dip-isopropyl groups (C33-C35 and C52-C54) were modelled and refined with two positions for each atom using geometry restraints (see Figure S106). The benzene molecule was also disordered and was modelled with two positions for each atom using geometry restraints. Refinement of this structure afforded a relatively high *R* value (9.1%), but the inner Mg_2OAd unit is well ordered and shows no large residual electron density peaks. (Note: A dataset of a crystal of **6**·2 C_6H_{14} from *n*-hexane shows full site disorder of the OAd unit and an overall poor quality.)

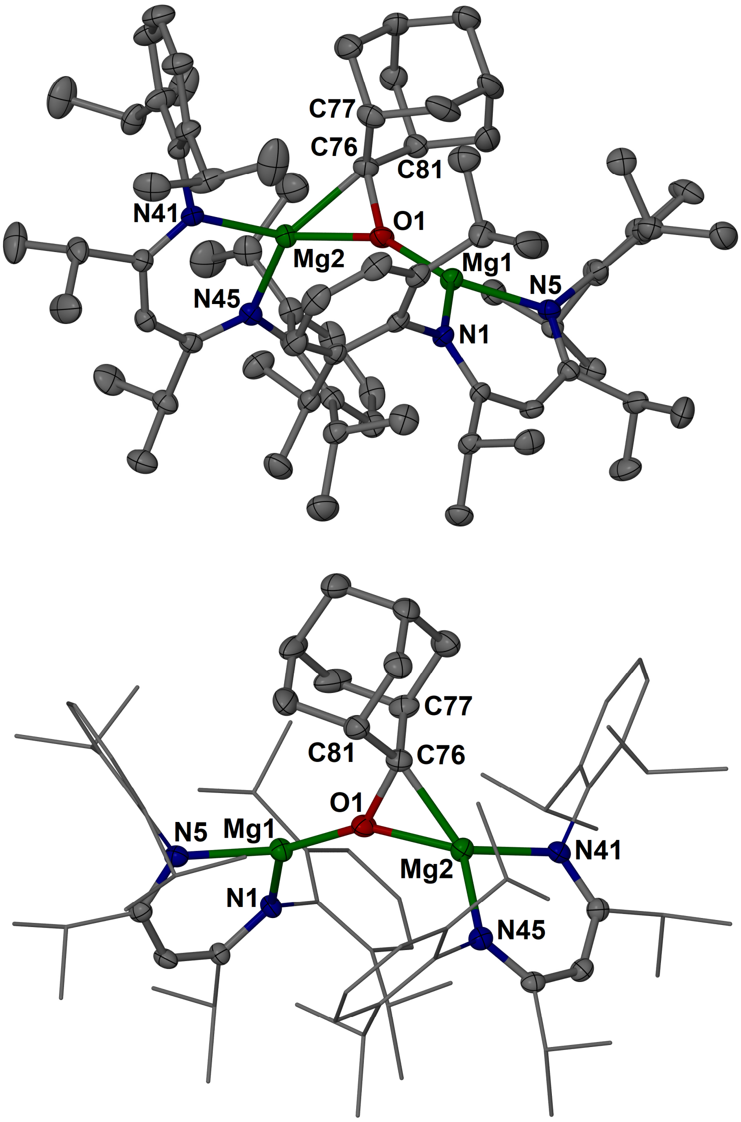

Figure S105. Molecular structure of **6**·C_6H_6 (30% thermal ellipsoids) in two views. Hydrogen atoms, solvent molecule and minor disordered parts omitted for clarity. Dip and *i*Pr groups are shown as wireframe (bottom). Selected bond lengths (Å) and angles (°): Mg1-O1 1.884(3), Mg1-N1 2.073(3), Mg1-N5 2.072(4), O1-C76 1.539(5), O1-Mg2 2.024(3), N1-C2 1.345(5), N1-C6 1.447(5),

Mg2-N41 2.077(4), Mg2-N45 2.077(4), Mg2-C76 2.106(5); O1-Mg1-N1 127.54(15), O1-Mg1-N5 136.04(15), N1-Mg1-N5 94.67(14), C76-O1-Mg1 138.1(2), C76-O1-Mg2 71.0(2), Mg1-O1-Mg2 150.09(16), C2-N1-C6 117.1(3), C2-N1-Mg1 117.7(3), C6-N1-Mg1 125.1(3), O1-Mg2-N41 135.44(15), O1-Mg2-N45 119.96(15), N41-Mg2-N45 94.06(15), O1-Mg2-C76 43.68(14), N41-Mg2-C76 121.90(17), N45-Mg2-C76 140.87(17), C77-C76-C81 107.3(4), C77-C76-O1 108.8(3), C81-C76-O1 111.8(4); sum of angles around C76 (excluding O1): 358.4(10)°; sum of angles around C76 (excluding Mg2): 327.9(11)°.

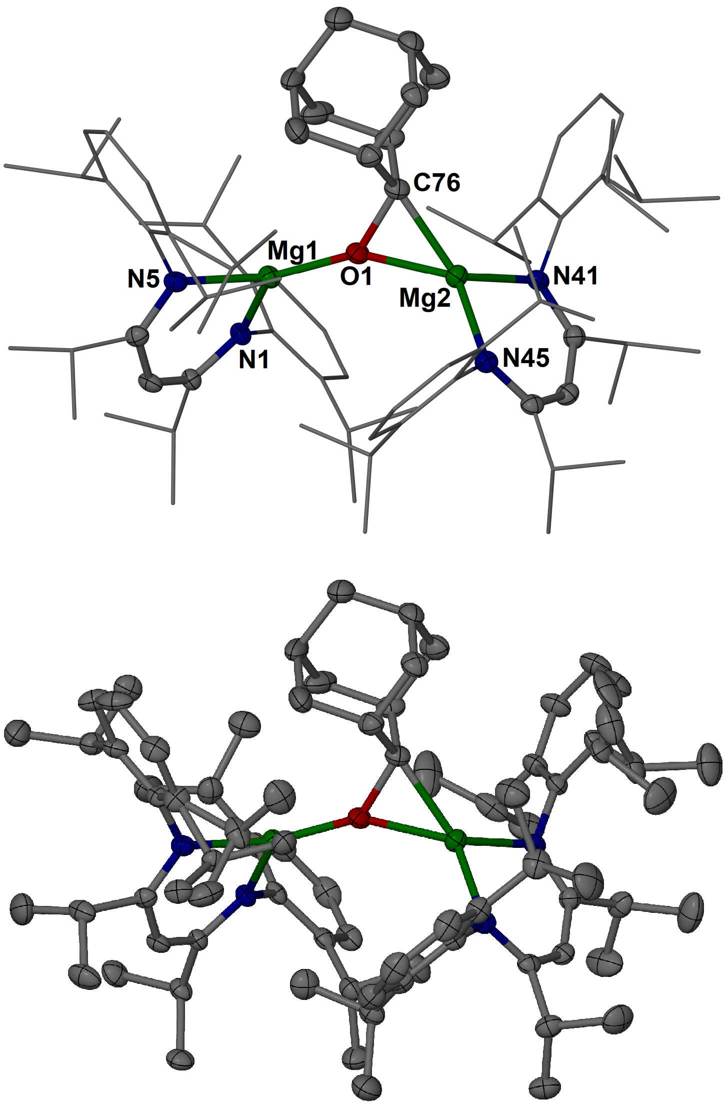

Figure S106. Molecular structure of $\mathbf{6}\cdot C_6H_6$ (30% thermal ellipsoids) in two views with the modelled *i*Pr groups included with both parts. Hydrogen atoms and solvent molecule omitted for clarity. Dip and *i*Pr groups are shown as wireframe in top image.

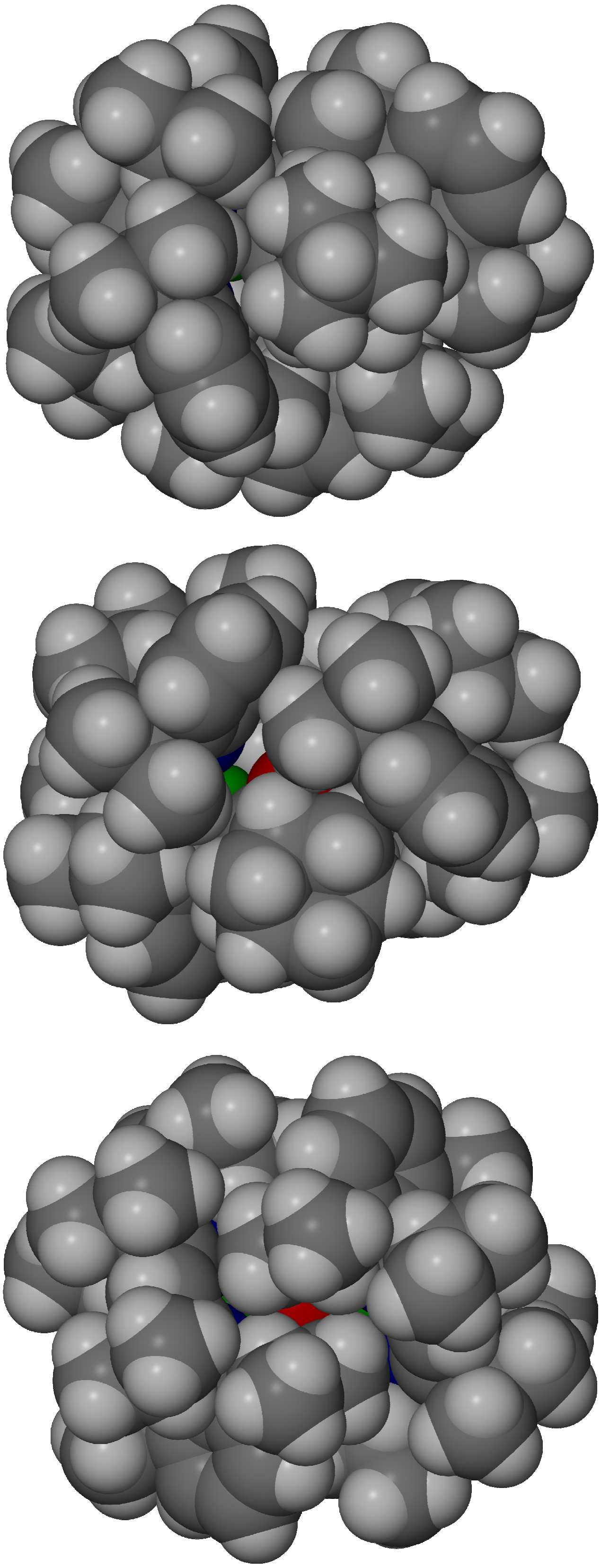

Figure S107. Space-filling model of **6** in three views. Minor disordered parts omitted. Mg: green, O: red, N: blue, C: grey, H: light grey. Top: view from "the top" along the C–O bond. Middle: view from the "more open" side onto the MgCOMg fragment. Bottom: view from "the bottom" onto the near-linear MgCOMg fragment.

Complex 7

No high-quality molecular structure of [{(^{iPrDip}nacnac)Mg}$_2$(μ-O)] **7** has so far been obtained, but a crystal from a high temperature decomposition of **6** included a well-ordered molecule of **7** (a quarter of the molecule in the asymmetric unit) alongside another molecule, possibly **7**, that showed severe full molecule disorder on a symmetry element, plus some disordered solvent. Removing the electron density of the disordered molecule and solvent molecules using PLATON/SQUEEZE[9] allowed **7** to be refined for an image but the data is not deposited, see Figure S108. Few provided bond lengths are given for guidance only. The related THF adduct [{(^{iPrDip}nacnac)Mg(THF)}$_2$(μ-O)] has been structurally characterised to higher quality (Figure S109).

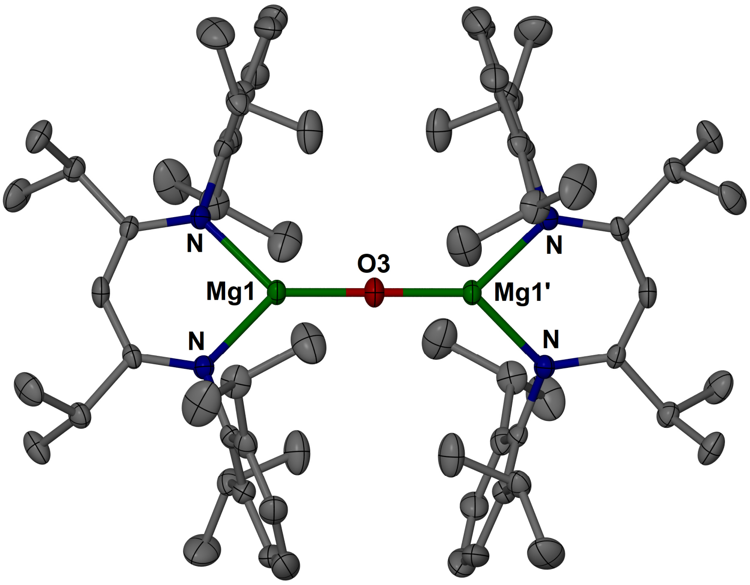

Figure S108. Image of the molecular structure of [{(^{iPrDip}nacnac)Mg}$_2$(μ-O)]. Bond lengths are provided as guidance only: Mg1-O3 1.8078(9), O3-Mg1' 1.8078(9), Mg1-N 2.0337(18), Mg1-N' 2.0338(17).

[{(^{iPrDip}nacnac)Mg(THF)}$_2$(μ-O)]

Complex [{(^{iPrDip}nacnac)Mg(THF)}$_2$(μ-O)]·C_6H_6 (see Figure S109) crystallised with half a molecule in the asymmetric unit. The coordinated THF ligand is disordered and was modelled and refined with two positions for the carbon atoms using geometry restraints.

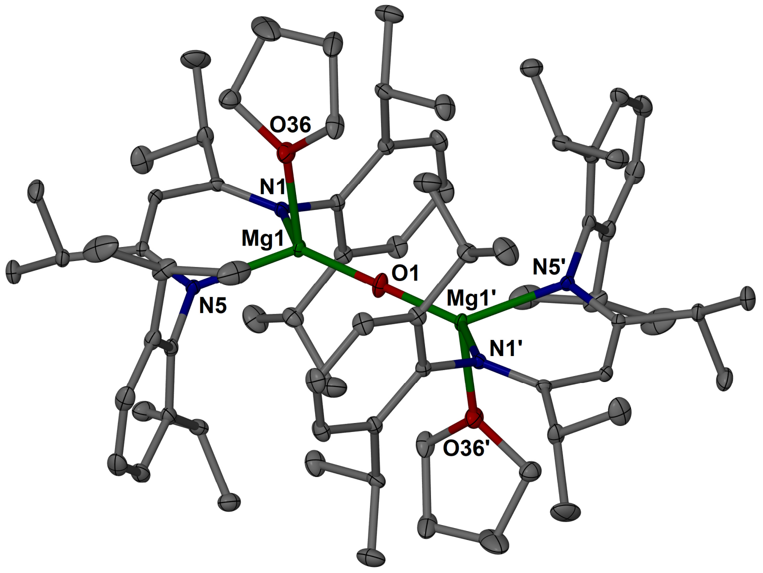

Figure S109. Molecular structure of [{(^{iPrDip}nacnac)Mg(THF)}$_2$(μ-O)]·C_6H_6 (30% thermal ellipsoids). Hydrogen atoms, solvent molecule and minor disordered parts omitted for clarity. Selected bond lengths and angles: Mg1-O1 1.8329(3), O1-Mg1’ 1.8330(3), Mg1-O36 2.0915(9), Mg1-N5 2.1125(9), Mg1-N1 2.1146(9); Mg1-O1-Mg1’ 180.00(2), O1-Mg1-O36 102.01(3), O1-Mg1-N5 128.88(3), O36 Mg1-N5 99.68(4), O1-Mg1-N1 130.00(3), O36-Mg1-N1 99.13(3), N5-Mg1-N1 90.58(3).

Complex 9

The molecular structure of [(^{iPrDip}nacnac)Mg(OCH*i*Pr$_2$)] **9**, see Figure S110, shows sever disorder and despite modelling the disorder using geometry restraints did not provide a high quality refined dataset and thus only an image is given. It does show the monomeric nature and similar overall structure to **10**, Figure S110.

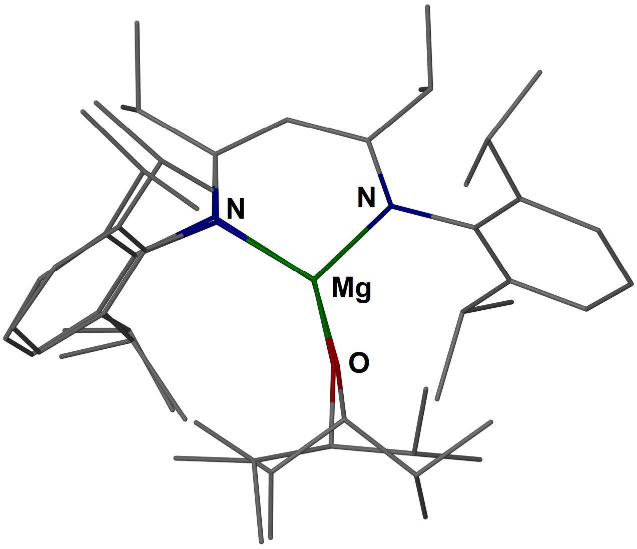

Figure S110. Disordered molecular structure of [(^{iPrDip}nacnac)Mg(OCH*i*Pr$_2$)] **9** shown in wireframe.

Complex 10

[($^{i\text{Pr}\text{Dip}}$nacnac)Mg{OC(=CMe$_2$)*i*Pr}] **10** crystallised with a full molecule in the asymmetric unit, see Figure S111.

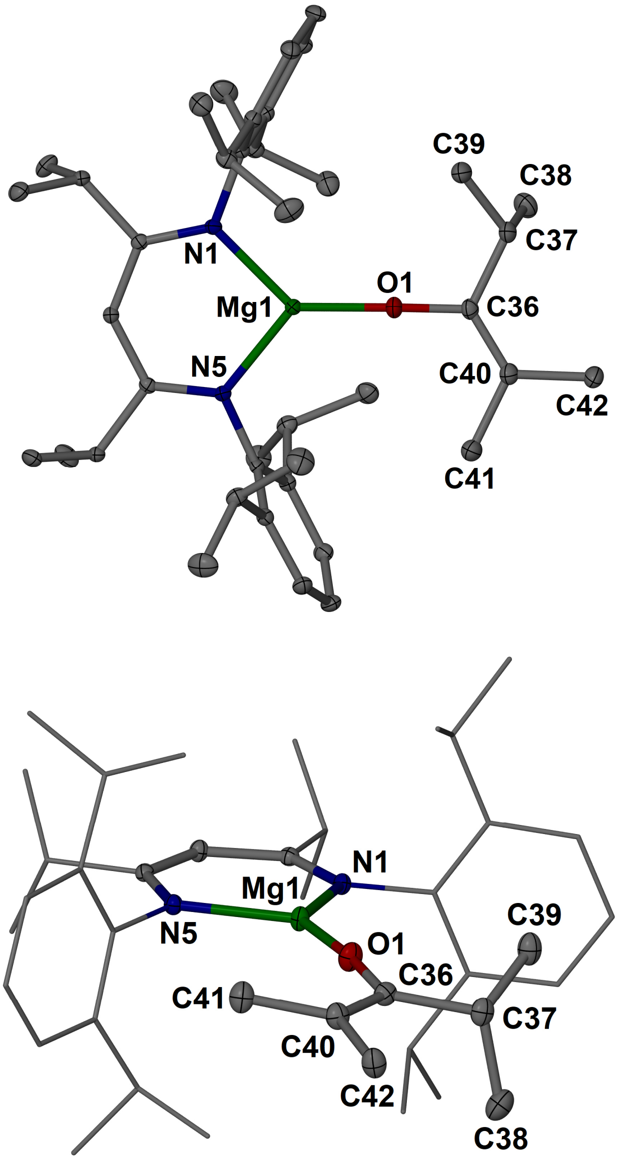

Figure S111. Molecular structure of **10** (30% thermal ellipsoids) in two views. Hydrogen atoms, solvent molecule and minor disordered parts omitted for clarity. Selected bond lengths and angles: Mg1-O1 1.7984(10), Mg1-N5 2.0123(13), Mg1-N1 2.0151(12), O1-C36 1.3477(15), C36-C40 1.3528(19), C36-C37 1.5279(19), C37-C39 1.531(2), C37-C38 1.532(2), C40-C41 1.510(2), C40-C42 1.5126(18); O1-Mg1-N5 128.92(5), O1-Mg1-N1 135.00(5), N5-Mg1-N1 95.98(4), C36-O1-Mg1 173.77(10), O1-C36-C40 122.44(12), O1-C36-C37 114.79(11), C40-C36-C37 122.72(11), C36-C37-C39 113.00(11), C36-C37-C38 111.38(12), C39-C37-C38 110.10(13).

Complex 11

The molecular structure of [(^{iPrDip}nacnac)Mg(OCH*t*Bu$_2$)] **11**·0.5 C_6H_6 is shown in Figure S112. The solvent molecule is poorly ordered and was refined using geometry restraints.

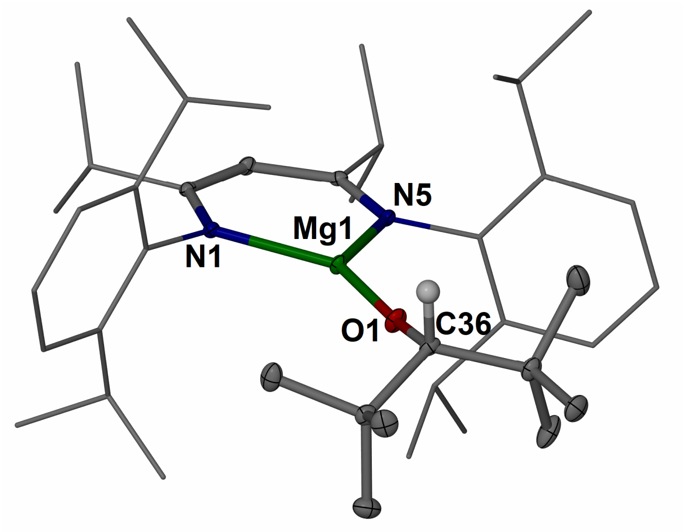

Figure S112. Molecular structure of **11**·0.5 C_6H_6 (30% thermal ellipsoids). Hydrogen atoms (except on C36), solvent molecule and minor disordered parts omitted for clarity. Dip and *i*Pr groups are shown as wireframe. Selected bond lengths and angles: Mg1-O1 1.8007(9), Mg1-N5 2.0190(10), Mg1-N1 2.0240(9), O1-C36 1.3998(13), C36-C41 1.5721(16), C36-C37 1.5740(16); O1-Mg1-N5 128.83(4), O1-Mg1-N1 136.55(4), N5-Mg1-N1 94.58(4), C36-O1-Mg1 164.95(7), O1-C36-C41 110.55(9), O1-C36-C37 108.70(9), C41-C36-C37 119.97(9).

Complex 12

Complex [{(^{MeMes}nacnac)Mg}(μ-OCH*t*Bu$_2$)(μ-CH$_2$-$^{MeMes\text{-}H}$nacnac)Mg] **12** is shown in Figure S113. The hydrogen atoms on C55 were found and freely refined.

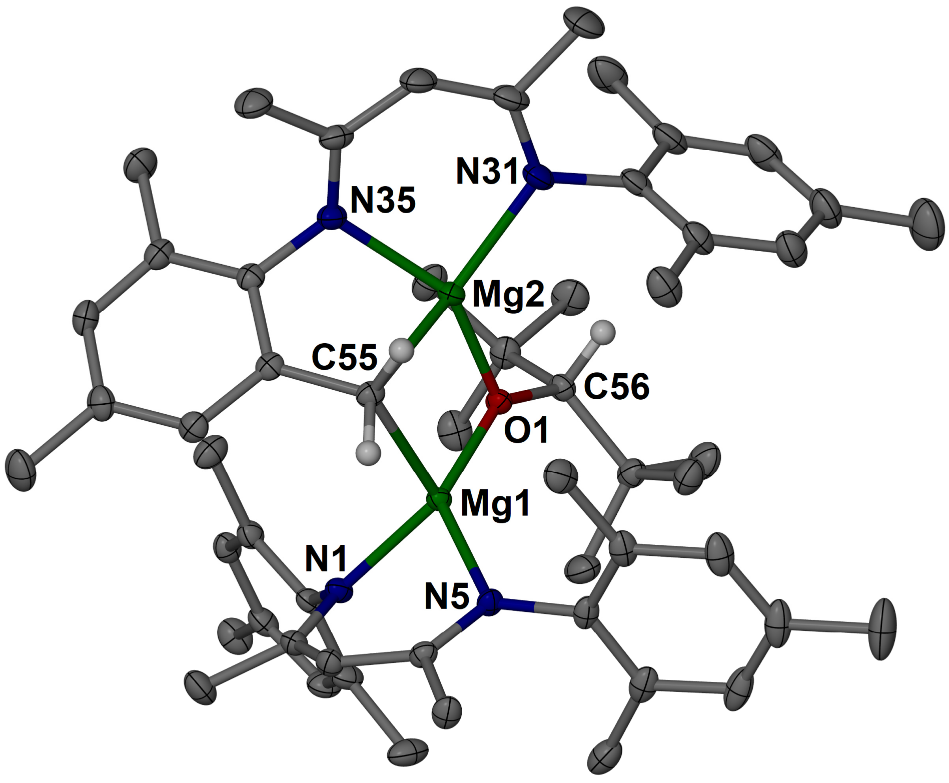

Figure S113. Molecular structure of **12** (30% thermal ellipsoids). Hydrogen atoms (except on C55 and C56) omitted for clarity. Selected bond lengths and angles: Mg1-O1 1.9491(13), Mg1-N1 2.0533(16), Mg1-N5 2.0907(15), Mg1-C55 2.293(2), Mg2-O1 1.9619(14), Mg2-N35 2.0588(17), Mg2-N31 2.0593(17), Mg2-C55 2.368(2), C52-C55 1.496(3), O1-C56 1.440(2), C56-C57 1.559(3), C56-C61 1.576(3); O1-Mg1-N1 122.09(6), O1-Mg1-N5 134.13(6), N1-Mg1-N5 92.14(6), O1-Mg1-C55 94.65(6), N1-Mg1-C55 110.60(7), N5-Mg1-C55 100.88(7), C52-C55-Mg1 116.73(12), C52-C55-Mg2 100.22(12), Mg1-C55-Mg2 77.21(6), O1-C56-C57 109.36(14), O1-C56-C61 110.35(14), C57-C56-C61 120.70(16).

Complex 13

Complex [{(^{MeMes}nacnac)Mg}(μ-OAd)(μ-O(Ad)CH$_2$-$^{MeMes-H}$nacnac)Mg] **13** is shown in Figure S114. A minor position (14% occupancy refined) of the adamantane cage on O2 was modelled using geometry restraints and the carbon atoms were refined isotropically.

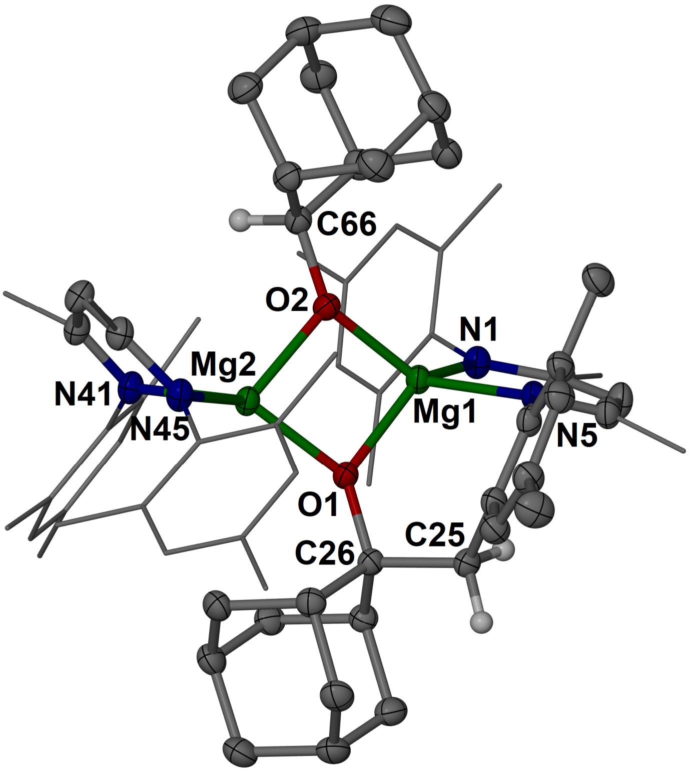

Figure S114. Molecular structure of **13** (30% thermal ellipsoids). Hydrogen atoms (except on C25 and C66), solvent molecule and minor disordered parts omitted for clarity. Dip and *i*Pr groups are shown as wireframe. Selected bond lengths and angles: Mg1-O2 1.9550(13), Mg1-O1 2.0136(13), Mg1-N5 2.0378(15), Mg1-N1 2.1184(16), Mg2-O1 1.9785(13), Mg2-O2 1.9844(13), Mg2-N45 2.0854(16), Mg2-N41 2.0970(15), O1-C26 1.427(2), O2-C66 1.417(2), C22-C25 1.513(3), C25-C26 1.563(2); O2-Mg1-O1 83.06(5), O2-Mg1-N5 122.90(6), O1-Mg1-N5 103.02(6), O2-Mg1-N1 125.05(6), O1-Mg1-N1 131.97(6), N5-Mg1-N1 92.67(6), O1-Mg2-O2 83.22(5), O1-Mg2-N45 129.53(6), O2-Mg2-N45 120.94(6), O1-Mg2-N41 126.31(6), O2-Mg2-N41 109.04(6), N45-Mg2-N41 89.51(6), C26-O1-Mg2 134.84(10), C26-O1-Mg1 128.17(10), Mg2-O1-Mg1 95.61(5), Mg1-O2-Mg2 97.31(6), C66-O2-Mg1 138.60(14), C66-O2-Mg2 121.47(14), C22-C25-C26 118.54(15), O1-C26-C33 109.93(13), O1-C26-C27 110.86(14), C33-C26-C27 107.37(15), O1-C26-C25 109.09(13), O2-C66-C73 112.84(19), O2-C66-C67 111.8(2).

4 Computational studies

Full complex **6** was optimised in the gas phase from the starting geometry obtained by X-ray diffraction using the M06 density functional[10] augmented with a D3 dispersion term,[11] coupled with the def2-TZVP basis set.[12] Natural Population Analysis (NPA) charges and Wiberg bond orders were calculated using Natural Bond Orbital (NBO) analysis.[13] The calculations were performed using Gaussian 16.[14] Quantum Theory of Atoms in Molecules (QTAIM) analysis was carried out using AIMAll.[15]

We estimated the dispersion contribution to the stability of complex **6** by calculating its electronic energy with and without Grimme's dispersion correction (GD3). This provides a value of 251 kJ/mol (60 kcal/mol). Note that this is likely an underestimate, as the M06 density functional is capable of recovering at least some dispersion energy. Thus, the dispersion contribution is sizeable.

Selected orbital energies of **6**: HOMO: -4.245 eV (see main text), LUMO: -1.069 eV (nacnac ligand based)

Selected Wiberg bond indices:

Mg1-N: 0.0202, Mg1-N: 0.0205, Mg1-O2: 0.0312

Mg4-N: 0.0364, Mg4-N: 0.0399, Mg4-C162: 0.1097, Mg4-O2: 0.0452

O2-C162: 0.8610

Atomic coordinates (XYZ) of complex 6

195
step 1 -----------
Mg 1.77577700 -0.23458800 0.08281800
O 0.04906400 -0.02007600 0.75542300
N 2.32428100 -1.49375600 -1.43218900
Mg -1.88018200 0.45087700 0.39044100
C 3.30053500 -1.14310900 -2.26580900
C 4.15665900 -0.06110600 -2.05279300
H 4.86619700 0.10616000 -2.85070100
C 4.36671600 0.74103800 -0.92639800
N 3.57211500 0.76802800 0.13319500
C 1.71155400 -2.76857500 -1.59485100
C 0.71687100 -2.99143400 -2.55731700
C 0.26669200 -4.28987600 -2.76206200
H -0.49198200 -4.47668200 -3.51521200
C 0.78257700 -5.34744100 -2.03997900
H 0.44372300 -6.35842800 -2.23439700
C 1.69522700 -5.10205900 -1.03198100
H 2.04981600 -5.92738800 -0.42528100
C 2.14461700 -3.81558700 -0.76489500
C 0.11335400 -1.85888600 -3.35367700
H 0.73909400 -0.97177900 -3.20906600
C -1.26624200 -1.53552200 -2.80509400
H -1.93471800 -2.39650900 -2.90782700
H -1.72802700 -0.69579000 -3.33235600
H -1.21560500 -1.28843000 -1.73890200
C 0.03303600 -2.13354300 -4.84743100
H 0.99849000 -2.43042400 -5.26326500
H -0.30212100 -1.23590800 -5.37532400
H -0.68560300 -2.92565800 -5.07221600
C 3.06801800 -3.55883800 0.40750200
H 2.86525800 -2.53874700 0.76584400
C 4.53902100 -3.60170500 0.01563500
H 4.77691100 -4.55784600 -0.46151600
H 5.17815700 -3.49848600 0.89697700
H 4.79857200 -2.79799300 -0.67770200
C 2.79729000 -4.48885700 1.57944500
H 3.14373300 -5.50588500 1.37771100
H 1.73176400 -4.53139800 1.81886000
H 3.32838100 -4.13631700 2.46665600
C 3.57124300 -1.91579200 -3.55488300
H 2.80735900 -2.68868200 -3.65382500
C 3.45567900 -1.01049200 -4.77687900
H 2.51245400 -0.45765600 -4.79138100
H 3.50090100 -1.61377300 -5.68711100
H 4.26812300 -0.28200400 -4.82632900
C 4.91595100 -2.62931600 -3.54156100
H 5.74227500 -1.93410000 -3.37036000
H 5.08375400 -3.12065600 -4.50299500
H 4.95482200 -3.39598100 -2.76628400
C 5.60896600 1.61478600 -0.99108300
H 5.72215300 2.10188200 -0.01824300
C 5.44315000 2.71113500 -2.03710800
H 5.37943600 2.29256700 -3.04482800
H 6.29944600 3.38953800 -2.01028400
H 4.54046200 3.30237500 -1.86556900
C 6.87265700 0.80391700 -1.24968200
H 6.92894700 -0.07513300 -0.60251100
H 7.75735600 1.41849600 -1.06601100
H 6.92489100 0.45363000 -2.28342900
C 3.96126700 1.48736200 1.30363800
C 4.74583500 0.84679300 2.27410600
C 4.99885700 1.50693400 3.46903200
H 5.59501200 1.01518300 4.23095900
C 4.50086700 2.77189800 3.70972600
H 4.69679800 3.26591700 4.65405800
C 3.76712200 3.40880200 2.72944100
H 3.39678400 4.41321300 2.90658600
C 3.49972600 2.79406700 1.51274800
C 5.32146200 -0.53526400 2.06404300
H 5.09799500 -0.84844800 1.03804700
C 6.83576100 -0.54640900 2.22583500
H 7.31027400 0.23474800 1.62722300
H 7.24338300 -1.51218400 1.91329800

H	7.12790400	-0.38794300	3.26733400
C	4.68376300	-1.54947300	3.00231800
H	4.79110100	-1.24200200	4.04638000
H	5.15571600	-2.52997700	2.89485100
H	3.61466700	-1.66557000	2.80668400
C	2.74793900	3.55638700	0.44827900
H	2.82446300	2.98747400	-0.48251200
C	3.35413700	4.92725500	0.18162200
H	2.88133100	5.37516500	-0.69792900
H	4.43076800	4.86704900	0.00135700
H	3.19353200	5.61056900	1.01959700
C	1.27193300	3.69012700	0.78411000
H	1.13572600	4.13848200	1.77328700
H	0.76975900	2.71927300	0.79248700
H	0.76544600	4.32280300	0.05395900
N	-3.52076300	-0.79223000	0.04153900
C	-4.24153400	-0.71396700	-1.06104900
C	-4.06912400	0.26900500	-2.04130600
H	-4.73854700	0.16856000	-2.88238800
C	-3.30711100	1.43727200	-2.05540900
N	-2.37982300	1.75690300	-1.15434500
C	-3.94679000	-1.64705300	1.09866500
C	-4.83879900	-1.13338200	2.05547900
C	-5.17880900	-1.92581600	3.14353000
H	-5.85484100	-1.53397500	3.89609700
C	-4.66581000	-3.19958500	3.29108700
H	-4.93538900	-3.80043800	4.15177100
C	-3.81919000	-3.70614800	2.32617600
H	-3.43469700	-4.71596500	2.43020700
C	-3.45317300	-2.95384200	1.21679400
C	-2.59307900	-3.59163400	0.14808100
H	-2.43659000	-2.85082800	-0.64162800
C	-3.30584100	-4.78681700	-0.47710500
H	-2.75062200	-5.13995500	-1.35202800
H	-4.32213100	-4.53974100	-0.79283000
H	-3.37176200	-5.61896200	0.22951200
C	-1.21961400	-4.02399600	0.64743900
H	-0.55446800	-3.17318000	0.80709900
H	-0.74426400	-4.67628000	-0.08594100
H	-1.28979400	-4.57456600	1.59082200
C	-5.37064700	-1.69767900	-1.33704900
H	-5.39985800	-2.40851200	-0.50675100
C	-6.72797900	-1.01050900	-1.39079000
H	-6.91661800	-0.42063400	-0.49112500
H	-7.52293300	-1.75504800	-1.47886900
H	-6.80460200	-0.33758100	-2.24872700
C	-5.11418000	-2.49410900	-2.60977000
H	-5.13441100	-1.85915800	-3.49899900
H	-5.88344500	-3.26137200	-2.73128500
H	-4.14426500	-2.99650300	-2.58060900
C	-3.68102700	2.40793900	-3.17149200
H	-2.87446800	3.13545500	-3.27956600
C	-4.92958000	3.18072200	-2.76330200
H	-5.77648600	2.50192200	-2.62756200
H	-5.19521200	3.91211100	-3.53062600
H	-4.77697800	3.71708500	-1.82394600
C	-3.87399600	1.75456400	-4.53299200
H	-3.06788600	1.05457600	-4.76968200
H	-3.89072800	2.52480000	-5.30836300
H	-4.81818700	1.20950400	-4.60411800
C	-1.74511500	3.02526300	-1.28430100
C	-0.70262900	3.20585600	-2.20773400
C	-0.18842300	4.47992800	-2.40802200
H	0.60223400	4.62577000	-3.13785000
C	-0.66508900	5.56283600	-1.69570600
H	-0.26481000	6.55446600	-1.87220900
C	-1.63263600	5.36280300	-0.73039600
H	-1.97345400	6.20493700	-0.13790100
C	-2.17833900	4.10587900	-0.49929400
C	-0.10521800	2.04436200	-2.96461100
H	-0.66281600	1.14435800	-2.68827100
C	-0.17590900	2.19157100	-4.47669700
H	0.44689400	3.01949900	-4.82650900
H	-1.19443100	2.37322200	-4.82435400
H	0.18981800	1.28017000	-4.96140100
C	1.33533200	1.84098200	-2.52875400
H	1.96185600	2.70114700	-2.78759200
H	1.78273700	0.96221500	-2.99708200
H	1.39294900	1.73636400	-1.43994000

C	-3.15335700	3.91454300	0.63920000
H	-3.69043500	2.97244200	0.47236900
C	-4.19268000	5.01734100	0.75790700
H	-3.74437300	5.96604300	1.06453100
H	-4.93152600	4.75256300	1.51820100
H	-4.72056600	5.18661100	-0.18347000
C	-2.36600500	3.79182800	1.94084000
H	-1.67431500	2.94211000	1.92071300
H	-3.03487500	3.65457800	2.79418300
H	-1.77663600	4.69785500	2.11272100
C	-0.66332200	0.16742400	2.06236600
C	-0.84928300	-1.19697800	2.72429600
H	-1.39273900	-1.85941700	2.04793800
C	-1.68551800	-1.03006500	3.99088600
H	-1.87072800	-2.01209300	4.44532600
H	-2.66190400	-0.60907200	3.73578400
C	-0.95576600	-0.12265500	4.97161500
H	-1.56544600	0.02189000	5.87112600
C	-0.69460900	1.22351000	4.30522700
H	-0.15635300	1.88491600	4.99606600
H	-1.64379300	1.71191800	4.06066300
C	0.13169600	1.02730400	3.03242900
H	0.33666500	2.01272100	2.60236300
C	0.47477800	-1.86057800	3.10722600
H	0.29038000	-2.85461100	3.53792700
H	1.09334800	-2.03379200	2.21112900
C	1.21743500	-0.96867100	4.09534600
H	2.17340900	-1.42670500	4.37587100
C	0.37288900	-0.76680000	5.34963000
H	0.19776600	-1.73095200	5.84220900
H	0.91439200	-0.13272400	6.06235900
C	1.46222900	0.37979500	3.42929100
H	2.10765500	0.24255500	2.55085400
H	2.03114500	1.04501400	4.09181400
C	-4.64263900	1.24117700	2.82111300
H	-5.03633400	2.25592200	2.70762100
H	-3.57591200	1.25376600	2.57669900
H	-4.72914500	0.96171800	3.87641700
C	-6.89562500	0.32360000	2.26704200
H	-7.46399700	-0.42572100	1.71080700
H	-7.29544100	1.30999100	2.01943600
H	-7.08328400	0.15826400	3.33117100
C	-5.41058300	0.26406900	1.94130000
H	-5.28956000	0.59753100	0.90403000

5 References

[1] S. J. Bonyhady, C. Jones, S. Nembenna, A. Stasch, A. J. Edwards, G. J. McIntyre, *Chem. Eur. J.* **2010**, *16*, 938–955.

[2] a) S. E. López, J. Restrepo, J. Salazar, *J. Chem. Res.* **2007**, *9*, 497–502; b) K. Yamamoto, H. Watnabe, *Chem. Lett.* **1982**, 1225–1228.

[3] *CrystalClear-SM Exper*t v2.1. Rigaku Americas, The Woodlands, Texas, USA, and Rigaku Corporation, Tokyo, Japan, 2015.

[4] *CrysAlisPro* v1.171.38.46. Rigaku Oxford Diffraction, Rigaku Corporation, Oxford, U.K. 2015.

[5] G. M. Sheldrick, *Acta Cryst.* **2015**, *A71*, 3–8.

[6] G. M. Sheldrick, *Acta Cryst.* **2015**, *C71*, 3–8.

[7] *CrystalStructure* v4.3.0. Rigaku Americas, The Woodlands, Texas, USA, and Rigaku Corporation, Tokyo, Japan, 2018.

[8] S. P. Green, C. Jones, A. Stasch, *Science* **2007**, *318*, 1754–1757.

[9] A. L. Spek, *Acta Cryst.* **2015**, *C71*, 9–18.

[10] Y. Zhao, D. G. Truhlar, *Theor. Chem. Acc.* **2008**, *120*, 215–241.

[11] S. Grimme, J. Antony, S. Ehrlich, H. Krieg, *J. Chem. Phys.* **2010**, *132*, 154104.

[12] F. Weigend, R. Ahlrichs, *Phys. Chem. Chem. Phys.* **2005**, *7*, 3297–3305.

[13] A. E. Reed, R. B. Weinstock, F. Weinhold, *J. Chem. Phys.* **1985**, *83*, 735–746.

[14] Gaussian 16, Revision C.01, M. J. Frisch, G. W. Trucks, H. B. Schlegel, G. E. Scuseria, M. A. Robb, J. R. Cheeseman, G. Scalmani, V. Barone, G. A. Petersson, H. Nakatsuji, X. Li, M. Caricato, A. V. Marenich, J. Bloino, B. G. Janesko, R. Gomperts, B. Mennucci, H. P. Hratchian, J. V. Ortiz, A. F. Izmaylov, J. L. Sonnenberg, D. Williams-Young, F. Ding, F. Lipparini, F. Egidi, J. Goings, B. Peng, A. Petrone, T. Henderson, D. Ranasinghe, V. G. Zakrzewski, J. Gao, N. Rega, G. Zheng, W. Liang, M. Hada, M. Ehara, K. Toyota, R. Fukuda, J. Hasegawa, M. Ishida, T. Nakajima, Y. Honda, O. Kitao, H. Nakai, T. Vreven, K. Throssell, J. A. Montgomery, Jr., J. E. Peralta, F. Ogliaro, M. J. Bearpark, J. J. Heyd, E. N. Brothers, K. N. Kudin, V. N. Staroverov, T. A. Keith, R. Kobayashi, J. Normand, K. Raghavachari, A. P. Rendell, J. C. Burant, S. S. Iyengar, J. Tomasi, M. Cossi, J. M. Millam, M. Klene, C. Adamo, R. Cammi, J. W. Ochterski, R. L. Martin, K. Morokuma, O. Farkas, J. B. Foresman, and D. J. Fox, Gaussian, Inc., Wallingford CT, 2016.

[15] AIMAll (Version 19.10.12), Todd A. Keith, TK Gristmill Software, Overland Park KS, USA, 2019 (aim.tkgristmill.com)
